# Spending on health and HIV/AIDS: domestic health spending and development assistance in 188 countries, 1995–2015

**DOI:** 10.1016/S0140-6736(18)30698-6

**Published:** 2018-05-05

**Authors:** Global Burden of Disease Health Financing Collaborator Network, Joseph L Dieleman, Annie Haakenstad, Angela Micah, Mark Moses, Cristiana Abbafati, Pawan Acharya, Tara Ballav Adhikari, Arsène Kouablan Adou, Aliasghar Ahmad Kiadaliri, Khurshid Alam, Reza Alizadeh-Navaei, Ala'a Alkerwi, Walid Ammar, Carl Abelardo T Antonio, Olatunde Aremu, Solomon Weldegebreal Asgedom, Tesfay Mehari Atey, Leticia Avila-Burgos, Ashish Awasthi, Rakesh Ayer, Hamid Badali, Maciej Banach, Amrit Banstola, Aleksandra Barac, Abate Bekele Belachew, Charles Birungi, Nicola L Bragazzi, Nicholas J K Breitborde, Lucero Cahuana-Hurtado, Josip Car, Ferrán Catalá-López, Abigail Chapin, Lalit Dandona, Rakhi Dandona, Ahmad Daryani, Samath D Dharmaratne, Manisha Dubey, Dumessa Edessa, Erika Eldrenkamp, Babak Eshrati, André Faro, Andrea B Feigl, Ama P Fenny, Florian Fischer, Nataliya Foigt, Kyle J Foreman, Nancy Fullman, Mamata Ghimire, Srinivas Goli, Alemayehu Desalegne Hailu, Samer Hamidi, Hilda L Harb, Simon I Hay, Delia Hendrie, Gloria Ikilezi, Mehdi Javanbakht, Denny John, Jost B Jonas, Alexander Kaldjian, Amir Kasaeian, Jennifer Kates, Ibrahim A Khalil, Young-Ho Khang, Jagdish Khubchandani, Yun Jin Kim, Jonas M Kinge, Soewarta Kosen, Kristopher J Krohn, G Anil Kumar, Hilton Lam, Stefan Listl, Hassan Magdy Abd El Razek, Mohammed Magdy Abd El Razek, Azeem Majeed, Reza Malekzadeh, Deborah Carvalho Malta, George A Mensah, Atte Meretoja, Ted R Miller, Erkin M Mirrakhimov, Fitsum Weldegebreal Mlashu, Ebrahim Mohammed, Shafiu Mohammed, Mohsen Naghavi, Vinay Nangia, Frida Namnyak Ngalesoni, Cuong Tat Nguyen, Trang Huyen Nguyen, Yirga Niriayo, Mehdi Noroozi, Mayowa O Owolabi, David M Pereira, Mostafa Qorbani, Anwar Rafay, Alireza Rafiei, Vafa Rahimi-Movaghar, Rajesh Kumar Rai, Usha Ram, Chhabi Lal Ranabhat, Sarah E Ray, Robert C Reiner, Nafis Sadat, Haniye Sadat Sajadi, João Vasco Santos, Abdur Razzaque Sarker, Benn Sartorius, Maheswar Satpathy, Miloje Savic, Matthew Schneider, Sadaf G Sepanlou, Masood Ali Shaikh, Mehdi Sharif, Jun She, Aziz Sheikh, Mekonnen Sisay, Samir Soneji, Moslem Soofi, Henok Tadesse, Tianchan Tao, Tara Templin, Azeb Gebresilassie Tesema, Subash Thapa, Alan J Thomson, Ruoyan Tobe-Gai, Roman Topor-Madry, Bach Xuan Tran, Khanh Bao Tran, Tung Thanh Tran, Eduardo A Undurraga, Tommi Vasankari, Francesco S Violante, Tissa Wijeratne, Gelin Xu, Naohiro Yonemoto, Mustafa Z Younis, Chuanhua Yu, Maysaa El Sayed Zaki, Lei Zhou, Bianca Zlavog, Christopher J L Murray

## Abstract

**Background:**

Comparable estimates of health spending are crucial for the assessment of health systems and to optimally deploy health resources. The methods used to track health spending continue to evolve, but little is known about the distribution of spending across diseases. We developed improved estimates of health spending by source, including development assistance for health, and, for the first time, estimated HIV/AIDS spending on prevention and treatment and by source of funding, for 188 countries.

**Methods:**

We collected published data on domestic health spending, from 1995 to 2015, from a diverse set of international agencies. We tracked development assistance for health from 1990 to 2017. We also extracted 5385 datapoints about HIV/AIDS spending, between 2000 and 2015, from online databases, country reports, and proposals submitted to multilateral organisations. We used spatiotemporal Gaussian process regression to generate complete and comparable estimates for health and HIV/AIDS spending. We report most estimates in 2017 purchasing-power parity-adjusted dollars and adjust all estimates for the effect of inflation.

**Findings:**

Between 1995 and 2015, global health spending per capita grew at an annualised rate of 3·1% (95% uncertainty interval [UI] 3·1 to 3·2), with growth being largest in upper-middle-income countries (5·4% per capita [UI 5·3–5·5]) and lower-middle-income countries (4·2% per capita [4·2–4·3]). In 2015, $9·7 trillion (9·7 trillion to 9·8 trillion) was spent on health worldwide. High-income countries spent $6·5 trillion (6·4 trillion to 6·5 trillion) or 66·3% (66·0 to 66·5) of the total in 2015, whereas low-income countries spent $70·3 billion (69·3 billion to 71·3 billion) or 0·7% (0·7 to 0·7). Between 1990 and 2017, development assistance for health increased by 394·7% ($29·9 billion), with an estimated $37·4 billion of development assistance being disbursed for health in 2017, of which $9·1 billion (24·2%) targeted HIV/AIDS. Between 2000 and 2015, $562·6 billion (531·1 billion to 621·9 billion) was spent on HIV/AIDS worldwide. Governments financed 57·6% (52·0 to 60·8) of that total. Global HIV/AIDS spending peaked at 49·7 billion (46·2–54·7) in 2013, decreasing to $48·9 billion (45·2 billion to 54·2 billion) in 2015. That year, low-income and lower-middle-income countries represented 74·6% of all HIV/AIDS disability-adjusted life-years, but just 36·6% (34·4 to 38·7) of total HIV/AIDS spending. In 2015, $9·3 billion (8·5 billion to 10·4 billion) or 19·0% (17·6 to 20·6) of HIV/AIDS financing was spent on prevention, and $27·3 billion (24·5 billion to 31·1 billion) or 55·8% (53·3 to 57·9) was dedicated to care and treatment.

**Interpretation:**

From 1995 to 2015, total health spending increased worldwide, with the fastest per capita growth in middle-income countries. While these national disparities are relatively well known, low-income countries spent less per person on health and HIV/AIDS than did high-income and middle-income countries. Furthermore, declines in development assistance for health continue, including for HIV/AIDS. Additional cuts to development assistance could hasten this decline, and risk slowing progress towards global and national goals.

**Funding:**

The Bill & Melinda Gates Foundation.

## Introduction

For more than 50 years, health spending has increased steadily, in many cases outpacing economic growth.^1^ As health spending grows as a share of the global economy, it is essential to know how spending on health is distributed among diseases and how national health spending differs. Assessing health spending, the source of the financing, and the distribution of the funds across different countries, populations, and diseases is crucial to ensure that funds for health are used optimally and equitably.

In many low-income countries, health spending amounts to less than $100 (2017 purchasing-power parity-adjusted dollars) per person annually, whereas in many high-income countries, health spending exceeds $5000 per person.^2^ While these national disparities are relatively well known, estimates of disease-specific spending around the world are scarce. Some National Health Accounts do exist for specific health areas, including maternal and child health, vaccines, malaria, tuberculosis, and HIV/AIDS.^3^ Disease-specific health accounting methodologies—such as National AIDS Spending Assessments—have also been developed and applied in many countries.^4^ The System of Health Accounts (2011) framework aims to fully allocate spending by disease area, including spending on cancers, diabetes, and other non-communicable diseases.^5^, ^6^ Finally, country-specific research on the USA, Norway, and other selected countries has quantified spending by disease in an even more detailed and comprehensive manner than before.^7^, ^8^, ^9^, ^10^ However, these previous cross-country comparisons have focused solely on either specific geographical regions or income groups over short time periods.^11^, ^12^ Despite this proliferation of country-level and regional estimates, very little is known about trends and the drivers of trends in spending by disease, because competing methodologies produce incomparable estimates. Major data gaps also limit comparisons over time and countries. With the exception of development assistance for health, disease-specific spending estimates have not been calculated in a way that permits cross-country comparison or comparison across time.

**Research in context**

**Evidence before this study**

Previous evidence about health financing, including global resource tracking efforts produced by WHO and the Global Burden of Disease (GBD) Health Financing Collaborator Network, has been published for diverse topics. In 2017, WHO updated estimates of health spending disaggregated by source, using System of Health Accounts (2011) classifications. Other research has captured health spending for subsets of countries and spending types. This study builds on those data and methods used previously by the GBD Health Financing Collaborator Network in 2017. The 2017 study used these data to illustrate health financing patterns and changes in health spending as countries develop. However, previous work by WHO and the Collaborator Network did not estimate uncertainty, did not estimate spending for all countries, and did not estimate disease-specific spending.

Previous evidence about HIV/AIDS spending includes National AIDS Spending Assessments, National Health Accounts, and other sources, which captured HIV/AIDS financing for short timespans and subsets of countries. To our knowledge, no study has collated these HIV/AIDS data and estimated spending for all countries across time. Furthermore, no comprehensive global estimates of total spending by disease or disaggregated by source or type of care exist to date for all countries, building from a broad dataset.

**Added value of this study**

This study improved estimates of health spending published in 2017, and expanded the scope to capture disease-specific spending. We added four more countries and estimated a complete series of health spending, disaggregated by source, for 188 countries, alongside 95% uncertainty intervals that quantify which estimates are most and least certain. We included recently published data from WHO that is based on the System of Health Accounts 2011, and built models favouring datapoints drawn from an identified source or clear estimation methods. We estimated development assistance for health disaggregated across health focus areas, expanded to include pandemic preparedness. Finally, we collected and harmonised 5385 datapoints to estimate HIV/AIDS spending across 188 countries. We report estimates of domestic government and private contributions to HIV/AIDS funding and spending on HIV/AIDS treatment and prevention.

**Implications of all the available evidence**

Total health spending continues to outpace economic growth in many contexts, but development assistance is levelling off. Global HIV/AIDS spending amounted to $562·6 billion from 2000 to 2015, although the amount of HIV/AIDS spending varies substantially across countries, income, and prevalence groups. The substantial share of spending sourced externally in countries with high HIV/AIDS prevalence highlights the risk posed by future reductions in development assistance for HIV/AIDS and the vigilance required to ensure that global goals, such as the UNAIDS Fast-Track Targets and Sustainable Development Goal target 3.3, are achieved. Comparable and comprehensive estimates of health spending and disease-specific spending are crucial for assessing the allocation of resources worldwide and pinpointing important gaps in spending. Paired with GBD estimates, these data make possible a diverse set of comparisons, including analyses across different periods, countries, income groups, regions, and funding sources.

Our study begins to fill this gap by tracking health spending on HIV/AIDS for 188 countries from 2000 to 2015. Quantifying HIV/AIDS spending is an initial step in conducting global disease-specific spending assessments and is a crucial priority for the international community and countries. The position of HIV/AIDS as a top global health priority was solidified in Millennium Development Goal 6 and Sustainable Development Goal (SDG) 3.^13^, ^14^ This attention has created an unprecedented level of HIV/AIDS financing data that have largely remained disparate, incomparable, and untapped. Collating and synthesising this information to produce comparable results has the potential to reveal the impact of looming declines in international HIV/AIDS financing.^15^

We aimed to investigate three features of global health spending to characterise the variation in health spending across health systems and to characterise how international and domestic partners have responded to recent global health crises, such as Ebola and HIV/AIDS. First, we estimated health spending worldwide, tracking how much was spent on health in 188 countries. Second, we tracked development assistance for health, tracing disbursements from their source, through intermediary channels, to recipients in low-income and middle-income countries. We aimed to quantify how much of that financing focuses on core health focus areas, including HIV/AIDS, pandemic preparedness, and other primary areas of health financing. Finally, we tracked international and domestic spending on HIV/AIDS, with funds disaggregated across four financing sources and broken down by spending on prevention and treatment. This evidence will be particularly important to both donors and countries as they coordinate responses to future global health challenges, move to adopt sustainable health financing polices that reduce reliance on development assistance for health, and chart courses towards the achievement of the SDGs.

## Methods

### Overview

Each health financing component we tracked required unique input data and, consequently, estimation focused on different time periods. We tracked health spending by source from 1995 to 2015, development assistance for health from 1990 to 2017, and HIV/AIDS spending from 2000 through to the end of 2015. Most spending estimates reported in this paper are reported using 2017 purchasing-power parity-adjusted dollars to adjust for inflation and to reflect the country-specific purchasing power of the resources. Development assistance for health estimates that are stratified by source, channel, or health focus area are tracked using 2017 US$ to reflect the quantity of development assistance for health provided by donors, using an internationally recognisable currency (ie, US$). Development assistance for health estimates stratified by recipient country are converted into 2017 purchasing-power parity-adjusted dollars based on the country to which the resources were provided.

### Estimating domestic health spending, government, prepaid private, and out-of-pocket spending

We extracted data about transfers from government domestic revenue (allocated to health purposes), social insurance contributions, compulsory prepayment, voluntary prepayment, other domestic revenue from households, corporations, and non-profit institutions serving the household, and gross domestic product (GDP), each measured in local currency, from the WHO Global Health Expenditure Database.^3^ We divided each health spending variable by GDP, also reported by WHO. To estimate domestic government spending on health, we added the value of transfers from government domestic revenue (allocated to health purposes), social insurance contributions, and compulsory prepayment. To estimate domestic prepaid private health spending, we added the values of voluntary prepayment, other domestic revenues from corporations, and other domestic revenues from non-profit institutions serving the household. Out-of-pocket spending is comprised of payments by households. Our tracking of domestic health spending focuses on current health spending and excludes major investment, such as building hospitals and research and development. We multiplied all health financing fractions by the GDP per capita series, measured in 2017 purchasing-power parity-adjusted dollars, to estimate spending per person in that currency.^16^

Many of the extracted data are not tied to an underlying data source and are estimated. Although more information is available in recent iterations,^6^, ^17^ the documentation of these tracking, estimation, and imputation methods remains, in some cases, poorly defined and inconsistent, or simply unreported. Furthermore, for a given country, these data vary substantially across time. To estimate health spending across time, country, and spending category, we used a spatiotemporal Gaussian process regression model.^18^ This model was developed for the Global Burden of Disease (GBD) Study to identify patterns across time and geographies.^18^ A further description of spatiotemporal Gaussian process regression model can be found in the appendix, along with out-of-sample statistics. To prevent data with unclear methods or proper data source identification from influencing our spatiotemporal Gaussian process regression model estimation, we developed a data weighting procedure. Each datapoint was assessed and assigned a weight between one and five on the basis of the point-specific metadata provided in the Global Health Expenditure Database. We based weights upon metadata completeness, documented source information, and documented methods for estimation. Our guidelines for assessing the metadata are included in the appendix.

### Tracking development assistance for health

Development assistance for health includes the financial and in-kind resources provided by development agencies to low-income and middle-income countries, with the primary objective of maintaining or improving health. We estimated development assistance for health using project records, annual reports, budgets, and financial statements from international organisations. We relied on commitment and budget data to generate estimates for the most recent years when disbursement data were not available.

Our estimates of development assistance for health tracked disbursements from the originating source through the disbursing agency, called the channel, to the recipient country and targeted health focus area or programme area. We used disbursement and income data to remove resources that were passed between development agencies before being disbursed to prevent double counting. We also accounted for the administrative expenses incurred by estimating in-kind expenses.

We disaggregated development assistance for health disbursements into nine health focus areas: HIV/AIDS, tuberculosis, malaria, maternal health, newborn and child health, other infectious diseases, non-communicable diseases, sector-wide approaches and health system strengthening, and other. The other category captured all projects that did not align with any of the other health focus areas. We further disaggregated these health focus areas by programme area, which are spending categories that represent programmatic aims or implementation approaches within the broader health focus areas. For example, we disaggregated development assistance for HIV/AIDS into treatment, diagnosis, care and support, counselling and testing, orphan and vulnerable children, prevention of mother-to-child transmission, and HIV/AIDS system support. Additionally, we tracked development assistance for pandemic preparedness as a programme area within sector-wide approaches and health system strengthening, and treatment and diagnosis as separate programme areas under tuberculosis. We used keywords from project titles, descriptions, and budgets to determine the targeted health focus and programme areas for projects.

We report development assistance for health estimates in 2017 US$, but converted disbursements from 2017 US$ to 2017 purchasing-power parity-adjusted dollars to add them to domestic spending estimates. We did this by first deflating disbursements to current US$, exchanging disbursements to the current national currency units of the recipient country, deflating to constant 2017 local currency, and then exchanging to 2017 purchasing-power parity-adjusted dollars. Detailed explanations of the methods used to track development assistance for health, including how disbursements for cross-cutting areas are allocated, are included in the appendix.

### Estimating HIV/AIDS spending

We estimated HIV/AIDS spending measures by financing source (government spending, out-of-pocket, and prepaid private spending) and three HIV/AIDS spending categories (prevention, care and treatment, and all other spending).

We extracted HIV/AIDS spending data from five data sources. First, we used the spending data in the AIDSinfo database.^19^ This UNAIDS-curated database collates countries' annual reports on progress towards global HIV/AIDS goals, which capture HIV/AIDS spending by governments and the private sector. Second, we used the public and private spending data reported by countries in proposals and concept notes submitted to the Global Fund to Fight AIDS, Tuberculosis and Malaria. We included only current and past spending data reported in these submissions. Third, we extracted data from all National Health Accounts that capture HIV/AIDS spending, including sub-accounts and data produced under the updated System of Health Accounts (2011) approach. Fourth, we extracted data from all National AIDS Spending Assessments, including spending on prevention and care and treatment.^4^, ^20^ Finally, we downloaded data for the Asia–Pacific region from the AIDS data hub. We converted all reported spending measures to 2017 purchasing power parity.

We aimed to adhere to the definition and boundaries of health spending as defined by the System of Health Accounts 2011 framework. This approach required us to harmonise the extracted data to correct for known definitional differences between data sources and observed biases within the data. The National AIDS Spending Assessment's definition of HIV/AIDS spending included spending on non-health related categories such as spending on orphan and vulnerable children, enabling environment, and social protection. To correct for this, we extracted data from these three non-health-related spending categories and subtracted their values from all National AIDS Spending Assessment-reported spending by financing source. This correction probably accounted for most definitional biases between National AIDS Spending Assessments and National Health Accounts, but the granularity with which the data were reported limited further efforts to harmonise these two data sources. Similarly, we removed orphan and vulnerable children disbursements from our development assistance for health data.

Not all data sources reported spending as granularly as we required. For example, some data sources only reported total domestic spending (sum of government, out-of-pocket, and prepaid private) or reported only private spending (sum of out-of-pocket and prepaid private). Although these spending measures did not match our measures of interest, they still provided valuable information. To use all available data, we estimated a total of five HIV/AIDS financing by source models (domestic, private, government, out-of-pocket, and prepaid private). To ensure internal consistency across all models, we developed a sophisticated aggregating procedure that included information about the number of underlying datapoints each series had, and how the estimated series related to each other. More information is provided in the appendix.

We used a spatiotemporal Gaussian process regression model to model each HIV/AIDS financing source and spending category model. For all HIV/AIDS spending variables, the model consisted of a mixed-effect model with random effects on GBD super-region, region, and country, as well as covariates ranging from antiretroviral therapy coverage to the natural log of lag distributed GDP per capita, natural log of HIV prevalence, natural log of HIV incidence, natural log of HIV mortality rate, and, the natural log of antiretroviral therapy prices. We determined the exact specifications of each model through out-of-sample prediction tests (appendix). We sourced all covariate estimates from the GBD Study 2016.^21^ To detect and reduce the influence of outlier datapoints, we used our previous model to measure the Cook's distance for each datapoint and excluded the datapoint if Cook's distance, D, was greater than 4/*n* where *n* is the number of extracted datapoints.

### Aggregating health and HIV/AIDS spending by income groups, regions, and HIV/AIDS severity

We reported health spending for each country, income group, and geographical region. We used 2017 World Bank income groups and GBD Study 2016 regions to categorise all years of data.^21^, ^22^ We aggregated rates by calculating total spending for the income group or region relative to the total income, number of prevalent cases, or health spending for the group or region. These measures reflect the income group or region as a whole, rather than reflecting the average of the nations that make up the group or region. We also grouped countries into three HIV/AIDS prevalence categories: low prevalence (<1% prevalence), high prevalence (1–5% prevalence), and extremely high prevalence (>5% prevalence). For these HIV/AIDS disease severity groups we extracted data from the GBD Study 2016.^23^ Categories were informed by cutoffs developed by UNAIDS.^24^ Finally, to compare health spending to health burden, we extracted country-specific disability-adjusted life-year estimates from the GBD Study 2016.^23^ We did this analysis using R (version 3.4.0), Stata (version 13), and Python (version 3.6).

### Role of the funding source

The funder of this study had no role in study design, data collection, data analysis, data interpretation, or writing of the report. All authors had full access to all the data in the study, and JLD and CJLM had final responsibility for the decision to submit for publication.

## Results

### Total health spending

In 2015, $9·7 trillion (95% uncertainty interval [UI] 9·7 trillion to 9·8 trillion) was spent globally on health. Spending per capita varied widely across countries, spanning from less than $100 per capita per year on health (Bangladesh, Benin, Burkina Faso, Burundi, Central African Republic, Democratic Republic of the Congo, Eritrea, Ethiopia, Madagascar, Mozambique, Niger, Somalia, South Sudan, and Togo) to more than $5000 per capita (Andorra, Austria, Denmark, Germany, Ireland, Luxembourg, the Netherlands, Norway, Sweden, Switzerland, and the USA; table 1). In 2015, high-income countries spent $5551 (5503 to 5605) per person on health, whereas upper-middle-income countries spent $949 (942 to 959) per capita. Lower-middle-income countries spent $266 (263 to 268) per capita and low-income countries spent $110 (108 to 111) per capita on health.

**Table 1:** Total health spending and spending by source, 2015

		**Total health spending per capita ($)**	**Total health spending as a share of gross domestic product (%)**	**Government health spending as a share of total health spending (%)**	**Prepaid private spending as a share of total health spending (%)**	**Out-of-pocket spending as a share of total health spending (%)**	**Development assistance for health as a share of total health spending (%)**	**Annualised rate of change in total health spending per capita, 1995–2015 (%)**
**Global**								
Total		1332 (1325 to 1343)	8·2% (8·1 to 8·2)	59·7% (59·2 to 60·0)	17·6% (17·3 to 17·9)	22·3% (22·1 to 22·4)	0·5% (0·5 to 0·5)	3·1% (3·1 to 3·2)
**Income group**								
	High-income countries	5551 (5503 to 5605)	11·7% (11·6 to 11·8)	64·2% (63·5 to 64·6)	21·7% (21·3 to 22·3)	14·1% (14·0 to 14·3)	0·0% (0·0 to 0·0)	3·0% (3·0 to 3·1)
	Upper-middle-income countries	949 (942 to 959)	5·7% (5·6 to 5·7)	57·7% (57·2 to 58·1)	10·1% (9·8 to 10·5)	32·0% (31·6 to 32·3)	0·2% (0·2 to 0·2)	5·4% (5·3 to 5·5)
	Lower-middle-income countries	266 (263 to 268)	3·9% (3·9 to 4·0)	31·9% (31·5 to 32·4)	7·4% (7·2 to 7·6)	57·7% (57·1 to 58·1)	3·1% (3·1 to 3·1)	4·2% (4·2 to 4·3)
	Low-income countries	110 (108 to 111)	6·3% (6·2 to 6·4)	21·6% (20·7 to 22·4)	7·1% (6·6 to 7·5)	39·0% (38·1 to 39·8)	32·3% (31·9 to 32·8)	1·9% (1·7 to 2·0)
**Global Burden of Disease super-region**								
Central Europe, eastern Europe, and central Asia		1288 (1273 to 1300)	6·1% (6·0 to 6·2)	61·9% (61·1 to 62·5)	3·0% (2·7 to 3·4)	34·8% (34·3 to 35·4)	0·3% (0·3 to 0·3)	3·5% (3·4 to 3·6)
Global Burden of Disease high income		5839 (5785 to 5897)	12·4% (12·3 to 12·5)	63·9% (63·2 to 64·3)	22·4% (21·9 to 23·0)	13·7% (13·6 to 13·9)	0·0% (0·0 to 0·0)	3·0% (2·9 to 3·1)
Latin America and Caribbean		1065 (1051 to 1077)	7·2% (7·1 to 7·3)	49·7% (49·2 to 50·3)	17·5% (17·0 to 18·0)	32·1% (31·6 to 32·6)	0·7% (0·7 to 0·7)	2·8% (2·7 to 2·8)
North Africa and Middle East		888 (872 to 905)	5·1% (5·0 to 5·2)	59·7% (58·7 to 60·6)	7·1% (6·7 to 7·5)	32·8% (31·8 to 33·8)	0·5% (0·4 to 0·5)	4·0% (3·8 to 4·2)
South Asia		210 (207 to 212)	3·6% (3·5 to 3·6)	25·6% (24·9 to 26·2)	7·8% (7·5 to 8·0)	64·6% (64·0 to 65·3)	2·0% (2·0 to 2·0)	4·6% (4·5 to 4·7)
Southeast Asia, east Asia, and Oceania		672 (663 to 682)	4·8% (4·8 to 4·9)	56·5% (55·9 to 57·1)	8·5% (8·0 to 9·0)	34·7% (34·1 to 35·2)	0·4% (0·3 to 0·4)	8·2% (8·1 to 8·4)
Sub-Saharan Africa		202 (199 to 206)	5·2% (5·1 to 5·3)	34·4% (33·5 to 35·2)	15·5% (15·0 to 16·0)	33·7% (32·5 to 35·1)	16·4% (16·1 to 16·6)	2·0% (1·9 to 2·2)
**Country**								
Afghanistan		168 (160 to 174)	10·2% (9·7 to 10·6)	5·6% (5·5 to 5·9)	0·7% (0·5 to 1·0)	76·6% (75·3 to 77·5)	17·1% (16·4 to 17·9)	2·5% (0·9 to 3·7)
Albania		848 (796 to 908)	7·1% (6·6 to 7·6)	41·6% (40·3 to 43·0)	2·7% (0·9 to 6·2)	54·8% (52·9 to 56·7)	0·8% (0·8 to 0·9)	4·4% (3·7 to 5·2)
Algeria		1026 (998 to 1055)	6·9% (6·7 to 7·1)	71·2% (70·4 to 71·8)	1·3% (1·3 to 1·3)	27·5% (27·2 to 28·0)	0·0% (0·0 to 0·0)	5·5% (5·3 to 5·7)
Andorra		9203 (8659 to 9745)	11·9% (11·2 to 12·6)	56·5% (54·2 to 58·6)	7·5% (7·5 to 7·6)	35·9% (35·6 to 36·5)	0·0% (0·0 to 0·0)	2·1% (1·6 to 2·6)
Angola		197 (177 to 216)	2·6% (2·3 to 2·8)	59·3% (56·4 to 63·1)	4·5% (3·0 to 6·9)	32·1% (29·6 to 33·3)	4·0% (3·6 to 4·4)	1·1% (0·5 to 1·7)
Antigua and Barbuda		1198 (1149 to 1251)	5·1% (4·9 to 5·3)	68·2% (66·5 to 69·0)	8·7% (8·4 to 9·1)	23·1% (22·8 to 23·2)	0·0% (0·0 to 0·0)	2·6% (2·3 to 3·0)
Argentina		1457 (1393 to 1528)	6·7% (6·4 to 7·0)	70·9% (70·4 to 71·4)	10·3% (8·9 to 12·0)	18·1% (16·3 to 20·0)	0·6% (0·6 to 0·6)	1·9% (1·6 to 2·3)
Armenia		849 (766 to 932)	9·2% (8·3 to 10·2)	17·4% (16·5 to 18·6)	1·4% (1·1 to 1·8)	79·8% (77·4 to 81·8)	1·3% (1·2 to 1·4)	9·8% (8·9 to 10·9)
Australia		4400 (4263 to 4559)	9·0% (8·8 to 9·4)	67·7% (66·3 to 68·6)	12·9% (12·3 to 13·5)	19·4% (17·8 to 22·2)	0·0% (0·0 to 0·0)	3·1% (2·9 to 3·2)
Austria		5183 (5116 to 5236)	10·3% (10·2 to 10·4)	75·6% (75·4 to 75·8)	6·5% (6·3 to 6·8)	17·9% (17·8 to 18·0)	0·0% (0·0 to 0·0)	2·2% (2·0 to 2·3)
Azerbaijan		1221 (1132 to 1322)	6·7% (6·2 to 7·2)	20·2% (19·8 to 20·6)	0·6% (0·6 to 0·7)	78·8% (77·5 to 80·3)	0·2% (0·2 to 0·3)	9·8% (8·0 to 12·1)
Bahrain		2470 (2363 to 2572)	5·0% (4·8 to 5·2)	66·6% (65·9 to 67·8)	8·9% (8·0 to 9·7)	24·5% (23·2 to 26·1)	0·0% (0·0 to 0·0)	2·6% (2·2 to 3·0)
Bangladesh		90 (86 to 94)	2·5% (2·4 to 2·7)	15·2% (13·5 to 16·8)	2·6% (2·4 to 2·9)	74·2% (73·7 to 74·8)	7·9% (7·6 to 8·3)	3·1% (2·6 to 3·5)
Barbados		1237 (1175 to 1293)	7·4% (7·0 to 7·7)	47·4% (45·5 to 48·6)	7·2% (7·2 to 7·3)	45·4% (45·2 to 45·8)	0·0% (0·0 to 0·0)	1·5% (1·1 to 1·9)
Belarus		1232 (1184 to 1275)	6·1% (5·8 to 6·3)	61·8% (61·6 to 62·4)	2·8% (2·7 to 3·0)	34·7% (33·0 to 37·1)	0·6% (0·6 to 0·7)	5·3% (4·8 to 5·7)
Belgium		4939 (4782 to 5095)	10·5% (10·2 to 10·9)	82·0% (81·1 to 83·1)	0·0% (0·0 to 0·0)	18·0% (15·4 to 19·5)	0·0%0·0 to 0·0)	3·1% (2·8 to 3·3)
Belize		544 (519 to 572)	6·1% (5·8 to 6·4)	65·9% (64·7 to 67·1)	5·7% (5·5 to 5·8)	23·1% (22·7 to 23·4)	5·2% (5·0 to 5·5)	3·2% (2·8 to 3·7)
Benin		82 (79 to 85)	3·8% (3·7 to 4·0)	21·1% (19·4 to 23·5)	5·4% (5·3 to 5·4)	42·9% (42·5 to 43·1)	30·6% (29·4 to 31·5)	1·0% (0·7 to 1·3)
Bhutan		285 (272 to 298)	3·5% (3·3 to 3·6)	71·8% (70·8 to 73·2)	1·1% (0·9 to 1·3)	19·7% (18·4 to 20·7)	7·4% (7·0 to 7·7)	2·9% (2·5 to 3·4)
Bolivia		450 (432 to 464)	6·3% (6·1 to 6·5)	68·1% (67·5 to 68·9)	3·0% (2·9 to 3·1)	26·3% (25·0 to 27·8)	2·6% (2·6 to 2·7)	5·3% (4·9 to 5·7)
Bosnia and Herzegovina		1076 (999 to 1174)	9·4% (8·7 to 10·2)	69·0% (65·8 to 71·0)	1·2% (0·5 to 2·9)	29·2% (25·1 to 34·7)	0·6% (0·5 to 0·6)	7·5% (6·1 to 8·8)
Botswana		1019 (946 to 1127)	5·9% (5·5 to 6·6)	55·2% (52·2 to 59·2)	31·3% (30·0 to 32·1)	5·3% (5·1 to 5·4)	8·2% (7·4 to 8·8)	2·2% (1·6 to 2·9)
Brazil		1431 (1407 to 1453)	8·9% (8·8 to 9·0)	43·2% (42·7 to 43·9)	28·3% (27·8 to 28·9)	28·4% (28·3 to 28·8)	0·0% (0·0 to 0·0)	2·4% (2·2 to 2·5)
Brunei		2092 (1942 to 2276)	2·6% (2·4 to 2·9)	88·9% (87·7 to 90·3)	4·9% (4·8 to 4·9)	6·1% (5·3 to 6·9)	0·0% (0·0 to 0·0)	−0·4% (−0·9 to 0·2)
Bulgaria		1620 (1566 to 1672)	8·3% (8·0 to 8·5)	51·5% (50·9 to 52·4)	1·2% (1·1 to 1·4)	47·2% (46·3 to 47·9)	0·1% (0·1 to 0·1)	6·4% (6·1 to 7·0)
Burkina Faso		94 (91 to 97)	5·4% (5·2 to 5·6)	29·9% (28·9 to 30·4)	6·2% (5·6 to 6·9)	36·1% (34·1 to 37·4)	27·8% (26·9 to 28·7)	3·1% (2·8 to 3·5)
Burundi		67 (63 to 71)	8·4% (8·0 to 8·9)	31·3% (28·6 to 34·1)	2·1% (1·5 to 2·8)	20·1% (17·1 to 23·0)	46·5% (43·8 to 48·9)	1·3% (0·8 to 1·9)
Cambodia		213 (199 to 229)	6·0% (5·6 to 6·4)	21·2% (19·0 to 24·2)	0·4% (0·3 to 0·5)	61·6% (60·3 to 62·8)	16·6% (15·5 to 17·9)	3·2% (2·5 to 3·7)
Cameroon		156 (148 to 163)	4·9% (4·6 to 5·1)	15·0% (13·9 to 16·1)	3·3% (1·5 to 6·5)	69·2% (68·7 to 69·9)	12·5% (12·0 to 13·2)	1·5% (1·0 to 1·9)
Canada		4921 (4835 to 5031)	10·4% (10·2 to 10·6)	73·7% (73·3 to 74·3)	11·9% (11·8 to 11·9)	14·4% (14·3 to 14·5)	0·0% (0·0 to 0·0)	2·5% (2·3 to 2·7)
Cape Verde		356 (340 to 372)	5·3% (5·1 to 5·6)	61·8% (60·5 to 63·3)	2·4% (2·1 to 2·6)	21·8% (21·4 to 22·4)	14·0% (13·4 to 14·7)	3·5% (3·0 to 4·1)
Central African Republic		28 (27 to 30)	4·3% (4·1 to 4·6)	14·2% (13·8 to 14·7)	4·7% (3·6 to 5·7)	45·1% (41·4 to 48·7)	35·9% (33·6 to 38·3)	−2·0% (−2·3 to −1·6)
Chad		103 (97 to 110)	4·3% (4·0 to 4·6)	27·7% (23·1 to 31·1)	5·7% (5·4 to 5·9)	58·6% (57·4 to 60·4)	8·0% (7·5 to 8·6)	0·2% (−0·2 to 0·6)
Chile		1950 (1921 to 1984)	8·0% (7·8 to 8·1)	60·7% (60·5 to 60·9)	6·7% (6·4 to 7·1)	32·6% (31·9 to 33·1)	0·0% (0·0 to 0·0)	4·5% (4·3 to 4·7)
China		779 (765 to 794)	5·3% (5·2 to 5·4)	59·1% (58·6 to 59·8)	7·9% (7·3 to 8·4)	33·0% (32·7 to 33·2)	0·0% (0·0 to 0·0)	10·1% (9·9 to 10·3)
Colombia		861 (806 to 914)	6·0% (5·6 to 6·4)	70·1% (68·6 to 71·6)	11·1% (10·8 to 11·5)	18·6% (17·7 to 19·2)	0·1% (0·1 to 0·1)	1·7% (1·2 to 2·2)
Comoros		131 (123 to 138)	8·3% (7·8 to 8·7)	13·0% (11·3 to 14·4)	3·7% (3·7 to 3·8)	73·4% (72·2 to 74·4)	9·9% (9·4 to 10·6)	−2·5% (−3·0 to −2·2)
Congo (Brazzaville)		181 (171 to 194)	2·9% (2·8 to 3·1)	48·0% (46·7 to 50·0)	2·0% (1·8 to 2·3)	45·0% (43·3 to 46·3)	4·9% (4·5 to 5·1)	2·7% (2·2 to 3·3)
Costa Rica		1339 (1300 to 1375)	8·2% (8·0 to 8·4)	75·5% (74·9 to 76·2)	2·4% (2·3 to 2·6)	22·0% (21·9 to 22·2)	0·1% (0·1 to 0·1)	4·2% (3·9 to 4·5)
Côte d'Ivoire		131 (108 to 162)	3·5% (2·9 to 4·4)	34·2% (25·7 to 44·8)	3·2% (1·4 to 6·2)	47·5% (43·1 to 50·9)	14·4% (11·5 to 17·3)	0·1% (−1·2 to 1·5)
Croatia		1736 (1660 to 1813)	7·4% (7·1 to 7·8)	77·8% (77·0 to 78·4)	7·2% (4·9 to 9·8)	14·9% (14·5 to 15·4)	0·0% (0·0 to 0·0)	3·2% (2·8 to 3·5)
Cuba		977 (870 to 1083)	10·4% (9·3 to 11·6)	93·2% (92·2 to 94·4)	1·8% (1·2 to 3·1)	4·6% (3·8 to 5·5)	0·3% (0·3 to 0·3)	7·2% (6·5 to 8·1)
Cyprus		2821 (2504 to 3127)	8·4% (7·5 to 9·3)	73·6% (72·8 to 74·9)	4·5% (4·4 to 4·6)	21·7% (17·6 to 25·7)	0·0% (0·0 to 0·0)	2·7% (1·9 to 3·6)
Czech Republic		2534 (2092 to 2924)	7·3% (6·0 to 8·4)	72·7% (70·6 to 75·4)	2·6% (1·4 to 6·3)	24·3% (18·0 to 30·6)	0·0% (0·0 to 0·0)	2·9% (1·8 to 4·1)
Democratic Republic of the Congo		44 (42 to 47)	4·4% (4·2 to 4·7)	15·6% (14·0 to 17·4)	7·0% (5·0 to 9·3)	36·7% (34·3 to 39·8)	40·6% (37·9 to 42·6)	2·6% (1·9 to 3·1)
Denmark		5144 (5049 to 5264)	10·3% (10·1 to 10·6)	84·1% (83·9 to 84·5)	2·1% (2·1 to 2·2)	13·8% (13·7 to 13·8)	0·0% (0·0 to 0·0)	2·9% (2·7 to 3·1)
Djibouti		147 (140 to 156)	4·2% (4·0 to 4·5)	57·8% (55·4 to 59·6)	1·6% (1·5 to 1·6)	21·7% (21·4 to 22·0)	19·0% (17·9 to 20·0)	0·6% (0·2 to 0·9)
Dominica		606 (591 to 620)	5·4% (5·3 to 5·5)	67·3% (67·0 to 68·0)	1·4% (0·7 to 2·5)	29·4% (29·3 to 29·8)	1·9% (1·8 to 1·9)	1·4% (1·2 to 1·6)
Dominican Republic		932 (905 to 968)	6·2% (6·0 to 6·5)	40·4% (39·3 to 41·9)	8·4% (8·0 to 8·8)	43·7% (42·6 to 44·1)	7·5% (7·3 to 7·8)	4·9% (4·5 to 5·3)
Ecuador		1028 (992 to 1077)	8·6% (8·3 to 9·1)	50·2% (49·4 to 51·0)	6·1% (5·7 to 6·6)	43·5% (42·6 to 44·1)	0·2% (0·2 to 0·2)	6·3% (5·8 to 6·7)
Egypt		484 (460 to 505)	4·2% (4·0 to 4·4)	30·1% (28·9 to 31·4)	7·7% (6·9 to 8·5)	61·9% (60·9 to 62·8)	0·4% (0·4 to 0·4)	2·0% (1·7 to 2·4)
El Salvador		598 (570 to 623)	6·9% (6·5 to 7·1)	64·2% (63·7 to 65·0)	5·8% (5·6 to 6·0)	28·1% (26·4 to 30·4)	1·8% (1·7 to 1·9)	3·1% (2·7 to 3·6)
Equatorial Guinea		1089 (988 to 1192)	2·9% (2·6 to 3·1)	21·9% (20·8 to 23·1)	9·4% (5·9 to 14·1)	67·8% (66·6 to 68·8)	0·8% (0·8 to 0·9)	9·5% (7·2 to 12·0)
Eritrea		41 (37 to 45)	3·2% (2·9 to 3·5)	23·8% (20·1 to 30·2)	4·3% (4·0 to 4·5)	55·2% (51·8 to 58·3)	16·6% (14·9 to 18·1)	−3·3% (−4·0 to −2·7)
Estonia		1946 (1922 to 1969)	6·4% (6·3 to 6·4)	75·1% (74·9 to 75·4)	1·8% (1·6 to 1·9)	23·2% (22·3 to 23·9)	0·0% (0·0 to 0·0)	4·5% (4·4 to 4·7)
Ethiopia		81 (77 to 85)	4·7% (4·5 to 4·9)	21·1% (19·0 to 23·4)	15·6% (14·2 to 16·7)	32·9% (31·9 to 34·0)	30·3% (28·9 to 31·8)	5·9% (5·4 to 6·4)
Federated States of Micronesia		239 (230 to 247)	7·4% (7·1 to 7·7)	44·6% (42·5 to 46·6)	0·3% (0·2 to 0·4)	4·2% (4·2 to 4·3)	50·9% (49·1 to 52·8)	4·9% (4·5 to 5·3)
Fiji		342 (328 to 358)	3·6% (3·5 to 3·8)	61·4% (60·4 to 63·3)	12·6% (11·4 to 14·1)	20·5% (19·9 to 20·8)	5·5% (5·2 to 5·7)	2·3% (1·8 to 2·6)
Finland		4101 (4035 to 4163)	9·4% (9·3 to 9·6)	77·5% (77·1 to 78·0)	2·8% (2·6 to 2·9)	19·7% (19·4 to 20·1)	0·0% (0·0 to 0·0)	3·3% (3·2 to 3·5)
France		4741 (4677 to 4799)	11·1% (10·9 to 11·2)	78·9% (78·7 to 79·1)	14·3% (14·1 to 14·4)	6·8% (6·7 to 6·9)	0·0% (0·0 to 0·0)	1·9% (1·8 to 2·0)
Gabon		487 (448 to 524)	2·7% (2·5 to 2·9)	58·9% (58·2 to 59·6)	13·6% (12·6 to 14·6)	26·2% (22·4 to 30·3)	1·2% (1·1 to 1·3)	−1·0% (−1·5 to −0·6)
Georgia		803 (754 to 860)	7·9% (7·4 to 8·5)	38·1% (36·3 to 39·6)	1·8% (0·6 to 3·6)	57·2% (55·4 to 59·8)	2·9% (2·7 to 3·1)	8·8% (7·0 to 10·5)
Germany		5532 (5366 to 5764)	11·1% (10·8 to 11·6)	84·2% (83·8 to 84·5)	3·3% (1·7 to 6·3)	12·5% (12·4 to 12·6)	0·0% (0·0 to 0·0)	1·8% (1·5 to 2·1)
Ghana		242 (234 to 250)	5·7% (5·5 to 5·9)	38·3% (36·2 to 40·5)	3·8% (3·5 to 4·1)	40·8% (40·0 to 41·6)	17·1% (16·6 to 17·7)	3·3% (3·0 to 3·6)
Greece		2352 (2181 to 2515)	8·5% (7·9 to 9·1)	62·8% (61·3 to 63·6)	3·5% (3·4 to 3·5)	33·7% (29·1 to 38·0)	0·0% (0·0 to 0·0)	1·5% (0·7 to 2·1)
Grenada		715 (671 to 773)	5·2% (4·9 to 5·6)	38·5% (36·5 to 39·9)	5·1% (2·4 to 8·2)	54·9% (54·1 to 55·3)	1·5% (1·4 to 1·6)	1·0% (0·5 to 1·5)
Guatemala		487 (459 to 514)	6·1% (5·8 to 6·5)	31·9% (31·3 to 32·6)	6·2% (5·9 to 6·5)	52·3% (50·2 to 54·0)	9·6% (9·1 to 10·2)	4·0% (3·5 to 4·6)
Guinea		102 (99 to 104)	6·5% (6·4 to 6·7)	11·7% (9·7 to 13·2)	2·2% (2·0 to 2·6)	41·0% (40·8 to 41·3)	45·0% (44·1 to 46·2)	2·9% (2·6 to 3·0)
Guinea-Bissau		121 (117 to 129)	7·9% (7·6 to 8·4)	24·6% (21·8 to 28·8)	1·8% (0·9 to 3·7)	32·4% (31·5 to 32·7)	41·1% (38·6 to 42·7)	0·5% (0·2 to 0·8)
Guyana		318 (298 to 335)	4·6% (4·3 to 4·8)	53·2% (50·8 to 56·0)	0·1% (0·1 to 0·1)	39·5% (39·3 to 40·0)	7·2% (6·8 to 7·7)	2·8% (2·3 to 3·5)
Haiti		135 (130 to 140)	7·6% (7·4 to 7·9)	9·9% (8·8 to 11·4)	4·2% (2·6 to 5·9)	33·7% (32·5 to 35·3)	52·2% (50·2 to 54·0)	−0·5% (−0·7 to −0·2)
Honduras		370 (351 to 397)	7·4% (7·0 to 7·9)	40·2% (38·4 to 41·9)	5·1% (4·9 to 5·2)	50·6% (49·1 to 51·6)	4·0% (3·7 to 4·2)	3·9% (3·4 to 4·4)
Hungary		2031 (1969 to 2100)	7·2% (7·0 to 7·5)	66·7% (66·3 to 67·3)	4·3% (3·9 to 4·8)	28·9% (28·3 to 29·4)	0·0% (0·0 to 0·0)	2·4% (2·2 to 2·7)
Iceland		4205 (4085 to 4323)	8·8% (8·5 to 9·0)	79·9% (79·5 to 80·4)	3·4% (3·2 to 3·6)	16·7% (16·2 to 17·1)	0·0% (0·0 to 0·0)	2·1% (1·8 to 2·4)
India		236 (233 to 239)	3·7% (3·7 to 3·8)	26·1% (25·3 to 26·7)	8·5% (8·3 to 8·7)	64·4% (64·2 to 64·5)	1·1% (1·1 to 1·1)	5·0% (4·9 to 5·1)
Indonesia		383 (365 to 398)	3·4% (3·2 to 3·5)	38·2% (38·0 to 38·4)	12·8% (12·6 to 13·2)	48·2% (46·0 to 49·9)	0·8% (0·7 to 0·8)	5·6% (5·2 to 6·1)
Iran		1232 (1171 to 1295)	7·1% (6·7 to 7·4)	48·7% (47·7 to 49·7)	7·6% (7·0 to 8·4)	43·7% (40·3 to 46·1)	0·0% (0·0 to 0·0)	5·1% (4·5 to 5·7)
Iraq		562 (502 to 644)	3·7% (3·3 to 4·2)	40·7% (38·0 to 41·9)	0·0% (0·0 to 0·0)	58·9% (54·5 to 63·4)	0·3% (0·2 to 0·3)	5·5% (4·2 to 6·7)
Ireland		5371 (5146 to 5576)	8·0% (7·6 to 8·3)	72·4% (71·8 to 73·5)	12·9% (12·3 to 13·2)	14·7% (14·0 to 15·7)	0·0% (0·0 to 0·0)	2·2% (1·9 to 2·6)
Israel		2560 (2417 to 2745)	7·1% (6·7 to 7·6)	65·3% (64·0 to 66·0)	11·3% (11·2 to 11·5)	23·3% (19·6 to 26·4)	0·0% (0·0 to 0·0)	1·4% (1·1 to 1·8)
Italy		3445 (3357 to 3526)	9·0% (8·8 to 9·2)	75·0% (74·7 to 75·5)	2·2% (2·0 to 2·4)	22·8% (22·5 to 23·1)	0·0% (0·0 to 0·0)	1·9% (1·7 to 2·1)
Jamaica		510 (479 to 542)	5·8% (5·5 to 6·2)	57·4% (55·1 to 58·8)	15·8% (15·7 to 15·9)	25·0% (23·5 to 26·9)	1·7% (1·6 to 1·8)	1·4% (0·9 to 1·8)
Japan		4286 (4163 to 4465)	10·4% (10·1 to 10·9)	86·8% (86·3 to 87·4)	0·0% (0·0 to 0·0)	13·2% (12·8 to 13·4)	0·0% (0·0 to 0·0)	3·7% (3·3 to 4·0)
Jordan		730 (687 to 774)	6·5% (6·1 to 6·9)	64·4% (63·6 to 64·9)	10·7% (9·1 to 12·2)	24·0% (22·0 to 26·7)	0·9% (0·8 to 0·9)	1·7% (1·2 to 2·2)
Kazakhstan		1017 (997 to 1040)	3·9% (3·8 to 4·0)	61·7% (61·4 to 62·0)	0·7% (0·6 to 0·8)	37·3% (36·9 to 38·1)	0·3% (0·3 to 0·3)	4·2% (4·1 to 4·4)
Kenya		187 (185 to 190)	5·8% (5·7 to 5·9)	30·6% (30·1 to 31·2)	12·9% (12·8 to 13·0)	30·0% (29·7 to 30·3)	26·4% (26·1 to 26·7)	2·1% (2·0 to 2·2)
Kiribati		189 (171 to 212)	10·1% (9·2 to 11·3)	76·3% (73·7 to 78·8)	0·0% (0·0 to 0·0)	4·7% (3·1 to 6·7)	18·9% (16·9 to 20·9)	−0·5% (−1·2 to 0·3)
Kuwait		2640 (2425 to 2869)	3·6% (3·3 to 3·9)	83·0% (82·1 to 84·0)	1·7% (1·5 to 1·8)	15·3% (14·4 to 16·4)	0·0% (0·0 to 0·0)	0·2% (−0·4 to 0·7)
Kyrgyzstan		308 (293 to 331)	8·6% (8·1 to 9·2)	43·4% (42·0 to 44·4)	1·7% (0·1 to 6·4)	46·8% (45·8 to 47·5)	8·1% (7·5 to 8·5)	3·1% (2·6 to 3·8)
Laos		178 (167 to 195)	2·8% (2·6 to 3·1)	34·2% (30·1 to 37·4)	2·8% (1·7 to 5·0)	44·7% (43·9 to 45·1)	18·2% (16·6 to 19·5)	3·3% (2·7 to 4·0)
Latvia		1683 (1593 to 1771)	6·5% (6·2 to 6·9)	61·5% (60·4 to 62·6)	1·0% (0·4 to 1·9)	37·5% (35·0 to 40·5)	0·0% (0·0 to 0·0)	4·1% (3·5 to 4·7)
Lebanon		1207 (1102 to 1312)	7·4% (6·7 to 8·0)	51·1% (50·2 to 52·9)	16·3% (16·1 to 16·5)	32·0% (28·9 to 35·1)	0·4% (0·4 to 0·5)	−0·6% (−1·2 to 0·0)
Lesotho		262 (254 to 270)	8·2% (8·0 to 8·5)	53·3% (51·7 to 55·2)	2·2% (1·8 to 2·7)	17·0% (16·9 to 17·1)	27·5% (26·6 to 28·3)	4·0% (3·7 to 4·3)
Liberia		481 (474 to 488)	53·9% (53·1 to 54·6)	2·3% (1·7 to 3·1)	0·3% (0·1 to 0·6)	5·6% (4·6 to 6·7)	91·8% (90·5 to 93·0)	15·4% (14·0 to 16·8)
Libya		502 (435 to 582)	8·0% (7·0 to 9·3)	51·0% (47·8 to 53·4)	9·5% (7·1 to 13·0)	39·1% (31·1 to 46·0)	0·2% (0·1 to 0·2)	−2·5% (−3·3 to −1·6)
Lithuania		1941 (1872 to 2010)	6·4% (6·2 to 6·6)	66·7% (65·9 to 67·6)	1·0% (0·9 to 1·1)	32·4% (32·2 to 32·8)	0·0% (0·0 to 0·0)	6·8% (6·3 to 7·3)
Luxembourg		6530 (6288 to 6784)	6·2% (5·9 to 6·4)	83·6% (82·8 to 84·1)	5·7% (5·5 to 6·0)	10·6% (9·6 to 12·2)	0·0% (0·0 to 0·0)	3·9% (3·6 to 4·2)
Macedonia		921 (758 to 1196)	6·3% (5·2 to 8·2)	61·2% (53·1 to 69·6)	2·7% (1·0 to 4·9)	35·1% (29·4 to 38·9)	0·8% (0·6 to 0·9)	4·1% (2·5 to 5·7)
Madagascar		78 (74 to 81)	5·3% (5·1 to 5·5)	42·9% (40·0 to 46·1)	6·5% (6·4 to 6·6)	22·5% (22·3 to 22·8)	28·1% (26·8 to 29·3)	0·6% (0·3 to 0·9)
Malawi		135 (132 to 138)	11·8% (11·5 to 12·0)	19·5% (19·1 to 20·3)	4·7% (3·6 to 6·0)	8·3% (7·2 to 9·6)	67·4% (66·2 to 69·0)	6·0% (5·7 to 6·3)
Malaysia		1072 (1041 to 1105)	4·0% (3·8 to 4·1)	52·6% (52·0 to 53·4)	10·8% (10·7 to 10·8)	36·6% (36·2 to 36·9)	0·0% (0·0 to 0·0)	5·8% (5·6 to 6·2)
Maldives		1850 (1719 to 1990)	11·6% (10·8 to 12·5)	80·1% (78·1 to 81·4)	1·7% (1·2 to 2·5)	18·0% (15·9 to 20·4)	0·2% (0·2 to 0·2)	6·2% (5·6 to 6·9)
Mali		110 (105 to 115)	5·6% (5·4 to 5·9)	15·9% (14·4 to 17·8)	4·7% (4·1 to 5·5)	47·4% (45·5 to 48·4)	32·0% (30·4 to 33·4)	2·1% (1·6 to 2·6)
Malta		3642 (3494 to 3766)	9·6% (9·2 to 9·9)	60·9% (60·2 to 62·0)	2·1% (2·0 to 2·2)	37·0% (34·6 to 38·7)	0·0% (0·0 to 0·0)	5·1% (4·7 to 5·5)
Marshall Islands		604 (565 to 646)	18·0% (16·9 to 19·1)	65·7% (63·7 to 68·3)	3·3% (2·9 to 3·7)	13·0% (12·8 to 13·1)	18·1% (16·8 to 19·3)	1·0% (0·5 to 1·5)
Mauritania		184 (174 to 194)	4·5% (4·3 to 4·8)	38·9% (36·3 to 41·5)	4·2% (3·7 to 5·1)	48·5% (47·5 to 50·1)	8·4% (7·9 to 8·9)	0·6% (0·2 to 1·0)
Mauritius		1094 (1047 to 1137)	5·3% (5·1 to 5·5)	46·3% (46·1 to 46·7)	0·9% (0·7 to 1·0)	52·7% (51·6 to 53·9)	0·1% (0·1 to 0·1)	6·5% (5·9 to 6·9)
Mexico		1081 (1050 to 1112)	5·9% (5·7 to 6·0)	52·0% (51·7 to 52·4)	6·5% (6·2 to 6·7)	41·3% (40·4 to 42·5)	0·2% (0·2 to 0·2)	3·6% (3·4 to 3·9)
Moldova		543 (516 to 574)	10·3% (9·8 to 10·9)	46·3% (43·8 to 47·7)	0·9% (0·9 to 1·0)	45·3% (43·8 to 46·4)	7·4% (7·0 to 7·8)	3·6% (3·1 to 4·3)
Mongolia		496 (475 to 522)	3·9% (3·7 to 4·1)	51·7% (50·2 to 53·2)	3·2% (2·9 to 3·6)	38·9% (37·4 to 40·4)	6·2% (5·9 to 6·5)	5·5% (4·8 to 6·1)
Montenegro		985 (954 to 1017)	5·9% (5·7 to 6·1)	66·5% (66·2 to 67·3)	0·5% (0·3 to 0·8)	32·5% (30·9 to 33·6)	0·5% (0·5 to 0·5)	2·5% (2·2 to 2·7)
Morocco		454 (438 to 472)	5·5% (5·4 to 5·8)	43·0% (41·5 to 44·7)	3·0% (2·5 to 3·6)	53·0% (52·2 to 53·5)	1·0% (1·0 to 1·0)	6·2% (5·6 to 6·8)
Mozambique		72 (71 to 74)	5·7% (5·6 to 5·9)	14·8% (13·1 to 16·6)	3·7% (3·5 to 3·8)	6·5% (6·0 to 7·0)	75·1% (73·1 to 76·5)	5·5% (5·3 to 5·8)
Myanmar		301 (270 to 339)	5·2% (4·6 to 5·8)	21·9% (21·0 to 22·4)	1·5% (1·3 to 1·7)	71·3% (68·3 to 74·5)	5·3% (4·7 to 5·9)	13·9% (12·0 to 15·9)
Namibia		1033 (991 to 1084)	8·8% (8·5 to 9·3)	63·0% (61·9 to 63·9)	20·4% (18·2 to 22·4)	8·5% (7·8 to 9·4)	8·1% (7·7 to 8·4)	1·8% (1·4 to 2·3)
Nepal		160 (153 to 167)	6·4% (6·1 to 6·7)	17·1% (16·9 to 17·4)	10·8% (9·9 to 11·7)	57·6% (56·1 to 59·4)	14·5% (13·9 to 15·1)	4·9% (4·3 to 5·6)
Netherlands		5579 (5360 to 5835)	10·7% (10·3 to 11·2)	80·8% (80·2 to 81·4)	7·1% (6·8 to 7·5)	12·1% (11·6 to 12·7)	0·0% (0·0 to 0·0)	3·0% (2·6 to 3·3)
New Zealand		3648 (3481 to 3856)	9·5% (9·1 to 10·1)	80·0% (79·0 to 81·2)	7·4% (6·8 to 8·0)	12·6% (12·2 to 12·8)	0·0% (0·0 to 0·0)	2·8% (2·5 to 3·2)
Nicaragua		432 (413 to 454)	8·1% (7·7 to 8·5)	54·4% (52·6 to 56·2)	2·2% (1·9 to 2·6)	34·5% (34·1 to 35·0)	8·9% (8·4 to 9·3)	2·9% (2·4 to 3·4)
Niger		67 (65 to 69)	6·5% (6·4 to 6·7)	25·3% (24·3 to 26·2)	1·4% (1·3 to 1·5)	54·6% (53·7 to 55·4)	18·7% (18·2 to 19·2)	1·0% (0·8 to 1·2)
Nigeria		216 (201 to 234)	3·5% (3·2 to 3·7)	16·1% (14·7 to 16·8)	1·8% (1·7 to 1·8)	73·5% (71·0 to 75·6)	8·6% (7·9 to 9·2)	6·2% (5·1 to 7·2)
North Korea		134 (128 to 139)	7·2% (6·9 to 7·5)	38·3% (36·8 to 40·1)	5·7% (4·2 to 8·0)	55·3% (54·8 to 55·9)	0·7% (0·7 to 0·7)	−1·7% (−2·1 to −1·3)
Norway		7024 (6810 to 7268)	9·9% (9·6 to 10·2)	85·3% (84·8 to 86·0)	0·4% (0·4 to 0·4)	14·3% (13·9 to 14·5)	0·0% (0·0 to 0·0)	3·8% (3·5 to 4·2)
Oman		1684 (1555 to 1799)	3·7% (3·4 to 4·0)	88·4% (87·5 to 89·6)	5·2% (5·1 to 5·3)	6·4% (6·0 to 6·8)	0·0% (0·0 to 0·0)	1·9% (1·3 to 2·5)
Pakistan		142 (136 to 150)	2·7% (2·6 to 2·9)	26·5% (24·7 to 28·1)	2·3% (2·2 to 2·3)	64·1% (63·7 to 64·6)	7·2% (6·8 to 7·5)	1·6% (1·1 to 2·1)
Palestine		390 (345 to 435)	9·7% (8·6 to 10·9)	39·8% (38·5 to 41·9)	19·2% (16·3 to 22·1)	40·2% (36·4 to 43·3)	0·6% (0·5 to 0·7)	2·4% (1·4 to 3·3)
Panama		1588 (1535 to 1649)	7·0% (6·7 to 7·2)	61·8% (60·6 to 63·3)	6·9% (6·5 to 7·1)	30·6% (30·3 to 30·8)	0·7% (0·7 to 0·7)	3·9% (3·4 to 4·3)
Papua New Guinea		121 (114 to 131)	3·8% (3·6 to 4·1)	74·5% (72·9 to 76·5)	0·0% (0·0 to 0·0)	5·6% (5·2 to 6·3)	19·9% (18·4 to 21·2)	3·0% (2·5 to 3·5)
Paraguay		738 (706 to 777)	7·8% (7·5 to 8·3)	53·4% (52·2 to 54·7)	9·7% (9·1 to 10·1)	36·3% (35·7 to 36·6)	0·6% (0·6 to 0·6)	4·6% (4·2 to 5·0)
Peru		683 (669 to 698)	5·4% (5·3 to 5·5)	59·5% (59·0 to 60·1)	6·7% (6·6 to 6·8)	31·0% (30·6 to 31·4)	2·8% (2·7 to 2·8)	4·5% (4·3 to 4·6)
Philippines		333 (324 to 347)	4·4% (4·3 to 4·6)	29·5% (29·1 to 29·6)	14·6% (14·4 to 14·8)	53·6% (52·8 to 54·9)	2·3% (2·2 to 2·4)	4·0% (3·7 to 4·2)
Poland		1757 (1671 to 1837)	6·2% (5·9 to 6·5)	71·2% (70·3 to 72·4)	5·0% (3·6 to 7·1)	23·8% (23·7 to 24·1)	0·0% (0·0 to 0·0)	2·8% (2·4 to 3·1)
Portugal		2712 (2621 to 2819)	9·0% (8·7 to 9·3)	66·2% (65·3 to 67·0)	6·2% (5·6 to 6·8)	27·6% (27·2 to 28·0)	0·0% (0·0 to 0·0)	3·0% (2·8 to 3·3)
Qatar		3251 (3050 to 3450)	2·7% (2·5 to 2·8)	84·1% (83·4 to 84·9)	8·7% (7·5 to 10·0)	7·1% (5·2 to 9·7)	0·0% (0·0 to 0·0)	1·9% (1·5 to 2·3)
Romania		1128 (1051 to 1198)	4·9% (4·6 to 5·3)	78·1% (77·0 to 79·1)	0·7% (0·6 to 0·8)	21·1% (20·5 to 21·5)	0·1% (0·1 to 0·1)	5·9% (5·2 to 6·8)
Russia		1544 (1523 to 1564)	5·7% (5·6 to 5·8)	61·6% (61·2 to 62·1)	2·7% (2·6 to 2·9)	35·7% (34·9 to 36·4)	0·0% (0·0 to 0·0)	3·3% (3·1 to 3·4)
Rwanda		149 (143 to 155)	7·9% (7·6 to 8·3)	24·4% (21·7 to 27·1)	8·6% (7·7 to 9·8)	26·0% (25·4 to 26·3)	40·9% (39·2 to 42·5)	6·4% (5·9 to 6·9)
Saint Lucia		714 (658 to 793)	5·9% (5·4 to 6·5)	39·9% (39·2 to 40·5)	4·5% (4·2 to 4·7)	51·2% (48·1 to 55·0)	4·4% (3·9 to 4·8)	1·1% (0·4 to 1·8)
Saint Vincent and the Grenadines		523 (506 to 537)	4·7% (4·5 to 4·8)	65·9% (65·5 to 66·6)	2·2% (1·8 to 2·5)	19·2% (17·5 to 20·8)	12·7% (12·3 to 13·1)	1·7% (1·4 to 2·0)
Samoa		342 (319 to 364)	6·5% (6·0 to 6·9)	67·7% (65·4 to 69·7)	0·8% (0·7 to 0·9)	10·3% (10·1 to 10·6)	21·1% (19·9 to 22·7)	2·5% (1·9 to 3·2)
São Tomé and Príncipe		216 (206 to 225)	6·5% (6·2 to 6·7)	47·5% (45·2 to 48·8)	2·0% (1·3 to 2·9)	17·7% (17·1 to 18·2)	32·8% (31·5 to 34·3)	0·8% (0·5 to 1·0)
Saudi Arabia		3138 (2975 to 3318)	5·6% (5·3 to 6·0)	71·8% (70·2 to 73·3)	13·3% (13·2 to 13·4)	14·9% (14·7 to 15·0)	0·0% (0·0 to 0·0)	5·1% (4·5 to 5·7)
Senegal		119 (113 to 123)	4·7% (4·4 to 4·9)	26·9% (26·2 to 27·9)	10·6% (9·7 to 11·9)	36·8% (34·4 to 39·2)	25·6% (24·6 to 26·9)	0·8% (0·4 to 1·1)
Serbia		1398 (1349 to 1459)	9·5% (9·2 to 10·0)	58·2% (57·7 to 58·5)	1·4% (0·9 to 2·2)	40·2% (38·9 to 42·2)	0·1% (0·1 to 0·1)	5·7% (5·2 to 6·3)
Seychelles		957 (870 to 1057)	3·4% (3·1 to 3·8)	97·1% (96·2 to 97·6)	0·1% (0·1 to 0·1)	2·6% (1·3 to 4·1)	0·2% (0·2 to 0·2)	−0·6% (−1·1 to 0·0)
Sierra Leone		248 (232 to 260)	16·4% (15·3 to 17·2)	9·3% (8·3 to 10·9)	4·5% (3·9 to 5·2)	47·3% (44·8 to 49·4)	38·8% (36·9 to 41·4)	3·4% (2·8 to 3·9)
Singapore		3657 (3529 to 3810)	4·2% (4·1 to 4·4)	51·6% (49·8 to 52·9)	16·7% (16·2 to 17·0)	31·7% (31·5 to 31·8)	0·0% (0·0 to 0·0)	3·8% (3·4 to 4·2)
Slovakia		2216 (2085 to 2350)	7·0% (6·6 to 7·4)	79·1% (77·9 to 80·1)	2·3% (0·8 to 4·8)	18·6% (16·2 to 21·4)	0·0% (0·0 to 0·0)	4·2% (3·7 to 4·8)
Slovenia		2806 (2744 to 2884)	8·5% (8·3 to 8·8)	71·2% (70·7 to 71·8)	16·2% (15·3 to 16·8)	12·6% (12·3 to 12·8)	0·0% (0·0 to 0·0)	3·2% (2·9 to 3·5)
Solomon Islands		157 (144 to 166)	7·9% (7·2 to 8·3)	64·7% (61·8 to 66·4)	0·2% (0·2 to 0·3)	3·4% (3·2 to 3·6)	31·6% (29·9 to 34·4)	2·1% (1·5 to 2·7)
Somalia		42 (42 to 43)	6·7% (6·6 to 6·9)	12·2% (11·5 to 13·0)	2·7% (2·5 to 2·8)	38·0% (37·2 to 39·0)	47·1% (46·4 to 47·9)	2·4% (2·2 to 2·6)
South Africa		1109 (1091 to 1128)	8·1% (8·0 to 8·3)	53·6% (53·4 to 53·8)	36·4% (35·6 to 37·1)	7·8% (7·5 to 8·2)	2·3% (2·3 to 2·3)	1·8% (1·7 to 1·9)
South Korea		2835 (2785 to 2884)	7·4% (7·3 to 7·5)	56·4% (55·7 to 57·1)	6·8% (6·4 to 7·2)	36·8% (36·4 to 37·3)	0·0% (0·0 to 0·0)	7·4% (7·0 to 7·7)
South Sudan		81 (79 to 84)	2·6% (2·5 to 2·6)	27·6% (26·7 to 28·4)	4·3% (4·1 to 4·4)	57·5% (57·0 to 58·1)	10·6% (10·3 to 10·9)	0·9% (0·6 to 1·1)
Spain		3363 (3262 to 3450)	9·1% (8·9 to 9·4)	71·0% (70·6 to 71·3)	4·8% (4·7 to 5·0)	24·2% (23·9 to 24·6)	0·0% (0·0 to 0·0)	3·0% (2·7 to 3·2)
Sri Lanka		360 (348 to 370)	3·0% (2·9 to 3·1)	53·8% (52·4 to 55·0)	6·7% (6·4 to 7·1)	36·4% (36·1 to 36·9)	3·0% (3·0 to 3·2)	2·7% (2·5 to 3·0)
Sudan		282 (262 to 306)	6·2% (5·8 to 6·8)	30·0% (28·1 to 33·4)	3·2% (3·2 to 3·3)	63·8% (61·5 to 64·8)	2·9% (2·7 to 3·1)	4·9% (4·1 to 5·5)
Suriname		993 (904 to 1074)	6·0% (5·5 to 6·5)	51·6% (50·7 to 52·3)	34·9% (32·3 to 38·1)	11·2% (10·4 to 12·2)	2·2% (2·0 to 2·4)	0·9% (0·4 to 1·4)
Swaziland		693 (661 to 729)	7·4% (7·1 to 7·8)	61·6% (59·5 to 63·4)	8·7% (8·4 to 9·0)	10·7% (10·7 to 10·9)	19·0% (18·0 to 19·9)	3·6% (3·2 to 4·0)
Sweden		5550 (5346 to 5748)	11·0% (10·6 to 11·4)	83·6% (83·1 to 84·3)	1·1% (1·1 to 1·2)	15·2% (15·0 to 15·5)	0·0% (0·0 to 0·0)	4·1% (3·7 to 4·4)
Switzerland		7465 (7252 to 7662)	11·9% (11·5 to 12·2)	70·5% (69·3 to 71·1)	6·5% (6·4 to 6·7)	23·0% (22·5 to 23·9)	0·0% (0·0 to 0·0)	3·0% (2·7 to 3·3)
Syria		241 (207 to 284)	4·6% (4·0 to 5·5)	40·9% (37·5 to 43·7)	6·1% (4·4 to 7·7)	50·5% (43·0 to 57·6)	2·4% (2·0 to 2·8)	1·4% (0·5 to 2·2)
Taiwan (Province of China)		2535 (2513 to 2555)	5·5% (5·5 to 5·6)	60·0% (59·6 to 60·3)	12·7% (9·6 to 16·3)	27·3% (23·8 to 30·3)	0·0% (0·0 to 0·0)	4·4% (4·3 to 4·5)
Tajikistan		200 (192 to 209)	6·8% (6·5 to 7·0)	28·7% (28·3 to 29·1)	0·4% (0·1 to 0·9)	63·7% (62·4 to 64·7)	7·3% (7·0 to 7·6)	7·3% (6·2 to 8·1)
Tanzania		161 (147 to 176)	5·8% (5·3 to 6·3)	36·8% (33·2 to 40·4)	2·2% (2·1 to 2·3)	28·6% (26·6 to 30·5)	32·4% (29·5 to 35·4)	2·3% (1·3 to 3·0)
Thailand		614 (588 to 643)	3·7% (3·6 to 3·9)	78·0% (77·4 to 78·3)	9·4% (9·3 to 9·5)	12·2% (10·2 to 14·6)	0·3% (0·3 to 0·3)	3·1% (2·7 to 3·4)
The Bahamas		1818 (1713 to 1935)	7·3% (6·9 to 7·8)	47·1% (45·5 to 48·1)	23·6% (22·6 to 24·8)	29·2% (27·2 to 31·9)	0·0% (0·0 to 0·0)	2·2% (1·0 to 3·6)
The Gambia		141 (135 to 148)	8·1% (7·8 to 8·6)	34·9% (32·4 to 37·6)	4·7% (4·1 to 5·0)	16·6% (16·5 to 16·7)	43·8% (41·5 to 45·6)	3·9% (3·5 to 4·4)
Timor-Leste		103 (96 to 112)	2·7% (2·5 to 2·9)	55·4% (51·6 to 58·7)	2·0% (1·6 to 2·2)	10·4% (8·5 to 11·3)	32·2% (29·3 to 34·5)	5·5% (4·8 to 6·3)
Togo		96 (92 to 101)	6·2% (5·9 to 6·5)	28·4% (26·5 to 30·3)	6·2% (6·1 to 6·6)	54·4% (53·2 to 55·2)	11·0% (10·5 to 11·6)	2·6% (2·2 to 3·0)
Tonga		241 (229 to 255)	4·6% (4·4 to 4·9)	59·6% (58·6 to 60·4)	5·4% (3·1 to 8·5)	12·7% (12·4 to 12·8)	22·3% (21·1 to 23·5)	1·7% (1·3 to 2·1)
Trinidad and Tobago		2024 (1917 to 2158)	5·9% (5·6 to 6·3)	54·1% (52·8 to 55·4)	8·9% (8·6 to 9·3)	37·0% (36·1 to 38·0)	0·0% (0·0 to 0·0)	5·9% (5·4 to 6·5)
Tunisia		791 (770 to 817)	6·7% (6·6 to 7·0)	56·7% (56·4 to 57·0)	3·5% (2·4 to 5·3)	39·6% (38·7 to 40·0)	0·2% (0·2 to 0·3)	3·4% (3·1 to 3·7)
Turkey		1029 (989 to 1074)	4·2% (4·1 to 4·4)	77·9% (77·6 to 78·5)	4·9% (4·1 to 6·0)	17·1% (15·0 to 18·5)	0·1% (0·1 to 0·1)	4·5% (3·9 to 5·2)
Turkmenistan		1171 (1078 to 1281)	6·1% (5·7 to 6·7)	24·6% (23·9 to 25·1)	4·7% (4·3 to 5·4)	70·4% (68·0 to 72·7)	0·2% (0·1 to 0·2)	4·7% (3·5 to 5·9)
Uganda		159 (146 to 168)	7·5% (6·9 to 8·0)	13·6% (11·6 to 16·9)	11·9% (8·6 to 15·5)	39·5% (37·8 to 40·8)	34·9% (32·9 to 37·8)	3·3% (2·8 to 3·8)
Ukraine		598 (575 to 624)	6·4% (6·2 to 6·7)	48·3% (46·8 to 49·3)	3·5% (3·5 to 3·5)	46·8% (45·7 to 47·6)	1·4% (1·4 to 1·5)	2·2% (1·8 to 2·5)
United Arab Emirates		2489 (2354 to 2636)	3·5% (3·3 to 3·7)	73·5% (72·8 to 74·2)	8·4% (7·8 to 8·8)	18·0% (15·7 to 21·8)	0·0% (0·0 to 0·0)	−0·7% (−1·1 to −0·4)
UK		4285 (4160 to 4409)	9·8% (9·6 to 10·1)	80·5% (80·2 to 80·8)	4·9% (4·8 to 5·0)	14·6% (13·1 to 15·6)	0·0% (0·0 to 0·0)	4·7% (4·4 to 5·0)
USA		9839 (9677 to 9983)	16·8% (16·5 to 17·0)	50·4% (49·8 to 51·1)	38·4% (38·0 to 38·8)	11·1% (11·1 to 11·2)	0·0% (0·0 to 0·0)	3·0% (2·9 to 3·1)
Uruguay		2038 (1943 to 2116)	9·2% (8·8 to 9·6)	69·7% (68·8 to 70·4)	14·0% (12·9 to 15·0)	16·3% (15·8 to 17·3)	0·0% (0·0 to 0·0)	2·3% (1·9 to 2·5)
Uzbekistan		451 (439 to 463)	6·2% (6·1 to 6·4)	53·6% (53·0 to 54·2)	2·6% (2·6 to 2·6)	42·6% (42·4 to 43·1)	1·2% (1·1 to 1·2)	3·7% (3·5 to 3·9)
Vanuatu		147 (136 to 161)	5·2% (4·8 to 5·7)	54·7% (51·2 to 58·7)	2·1% (2·0 to 2·2)	6·1% (5·7 to 6·4)	37·1% (33·9 to 40·1)	6·1% (5·0 to 7·2)
Venezuela		590 (559 to 616)	3·6% (3·4 to 3·7)	47·4% (47·2 to 47·8)	5·0% (5·0 to 5·1)	47·5% (44·5 to 49·5)	0·0% (0·0 to 0·0)	−0·3% (−0·6 to 0·1)
Vietnam		320 (308 to 334)	5·1% (4·9 to 5·4)	46·1% (45·1 to 47·2)	3·3% (2·5 to 4·0)	47·8% (47·3 to 48·6)	2·8% (2·7 to 2·9)	6·7% (6·1 to 7·4)
Yemen		179 (157 to 199)	6·5% (5·7 to 7·2)	13·1% (11·8 to 14·6)	1·2% (1·0 to 1·3)	78·9% (76·1 to 81·3)	6·8% (6·1 to 7·8)	1·7% (0·7 to 2·5)
Zambia		241 (231 to 251)	6·0% (5·7 to 6·2)	31·6% (29·0 to 34·0)	10·5% (9·8 to 11·4)	25·3% (25·1 to 25·4)	32·5% (31·3 to 34·0)	3·6% (3·1 to 4·0)
Zimbabwe		191 (181 to 201)	8·8% (8·4 to 9·3)	25·4% (24·9 to 26·1)	17·5% (15·3 to 19·5)	29·1% (27·1 to 31·3)	28·0% (26·5 to 29·5)	−2·3% (−2·8 to −1·9)

Spending is reported in 2017 purchasing-power parity-adjusted dollars. 95% uncertainty intervals are shown in parentheses. Income groups are 2017 World Bank income groups.

Globally, health spending per capita grew at an annualised rate of 3·1% (95% UI 3·1–3·2) between 1995 and 2015. Growth was largest in upper-middle-income countries, where health spending per capita grew by 5·4% (5·3–5·5) and in lower-middle-income countries, where it grew by 4·2% (4·2–4·3). Spending in low-income countries increased at a rate of 1·9% (1·7–2·0) annually and was the lowest rate of growth observed among income groups, whereas in high-income countries, which generally aim to slow health spending growth, per capita growth was 3·0% (3·0–3·1).

**Figure 1:**
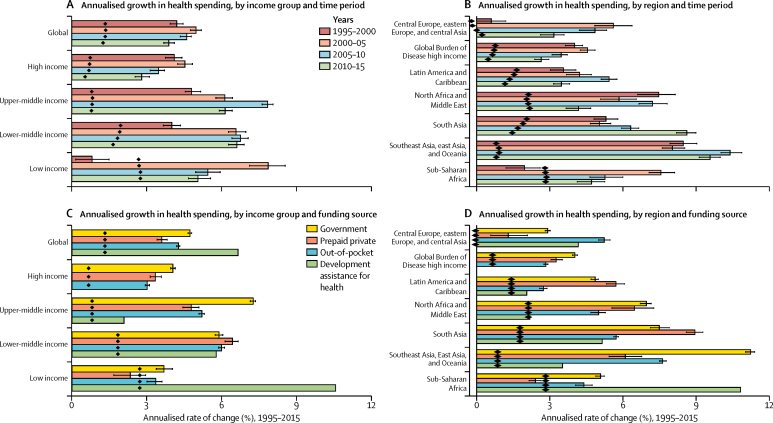
Annualised rate of change of health spending and population, 1995–2015 Annualised rate of change of health spending and population by income groups (A,B) and Global Burden of Disease super-regions (C,D). Income groups are 2017 World Bank income groups and held constant across time. Diamonds represent population growth rates. Error bars represent uncertainty intervals for rate of change of health spending.

Figure 1 shows annualised rates of change for health spending and population across income groups and regions. Between 1995 and 2000, spending in low-income countries increased by 0·8% (95% UI 0·2–1·5) annually. Subsequently, growth rose steeply to 7·8% (7·1–8·5) between 2000 and 2005, and then grew by 5·4% (5·0–5·9) from 2005 to 2010, and 5·0% (4·7–5·5) from 2010 to 2015 (figure 1). This finding contrasts with the other income groups: the highest rates of growth in health spending are observed from 2005 to 2010 for lower-middle-income and upper-middle-income countries, and from 2000 to 2005 for high-income countries (figure 1A). Additionally, the growth rates in population have declined for all other income groups except for low-income countries, for which annualised population growth rates remained at roughly 2·8% over the entire period. Because of these sustained populations, only marginal increases in per capita growth were observed in low-income countries. Across regions, health spending grew the most in southeast Asia, east Asia, and Oceania, which grew at an annualised rate of 9·1% (9·0–9·2) from 1995 to 2015, while health spending grew the slowest in central and eastern Europe and central Asia, at 3·5% (3·4–3·6) over the same period (figure 1B).

In addition to spending more on health, wealthy nations also tended to finance more health spending from public sources of funds. Panels C and D of figure 1 reflect the annualised rates of change in the source of funds for health spending and population, from 1995 to 2015, by income groups and regions. In low-income countries, health spending financed by the government grew by 3·7% (95% UI 3·4–4·0) annually between 1995 and 2015, compared with an annual growth of 10·5% in spending financed by development assistance for health over the same period. In lower-middle-income countries, similar levels of growth are observed in all the different sources of health spending over the same period (figure 1C). From 1995 to 2015, spending financed by government increased the most each year in upper-middle-income (7·2% [7·1–7·3]) and high-income countries (4·0% [3·9–4·1]), compared with other income groups. Across regions, southeast Asia, east Asia, and Oceania had the highest growth in spending financed by government sources: 11·2% (11·0–11·4) annually from 1995 to 2015. South Asia had the highest annualised growth rates in spending financed by prepaid private sources (8·9% [8·6–9·3]), whereas sub-Saharan Africa had the highest annualised growth rates in development assistance for health (10·8%) over the same period (figure 1D). Sub-Saharan African countries had the highest population growth rates (figure 1).

Despite clear patterns connecting total health spending and national income, country-level spending varied substantially, even within income groups and geographical regions (table 1). Across the low-income country group, health spending per capita, in 2015, spanned from $28 (95% UI 27–30) in the Central African Republic to $481 (474–488) in Liberia. Within the lower-middle-income group, health spending per capita spanned from $90 (86–94) in Bangladesh to $849 (766–932) in Armenia. Within the upper-middle-income group, health spending per capita ranged from $241 (229–255) in Tonga to $1850 (1719–1990) in the Maldives. Finally, in high-income countries, health spending per capita was lowest in the Seychelles at $957 (870–1057) and highest in the USA, at $9839 (9677–9983; table 1).

### Development assistance for health

**Figure 2:**
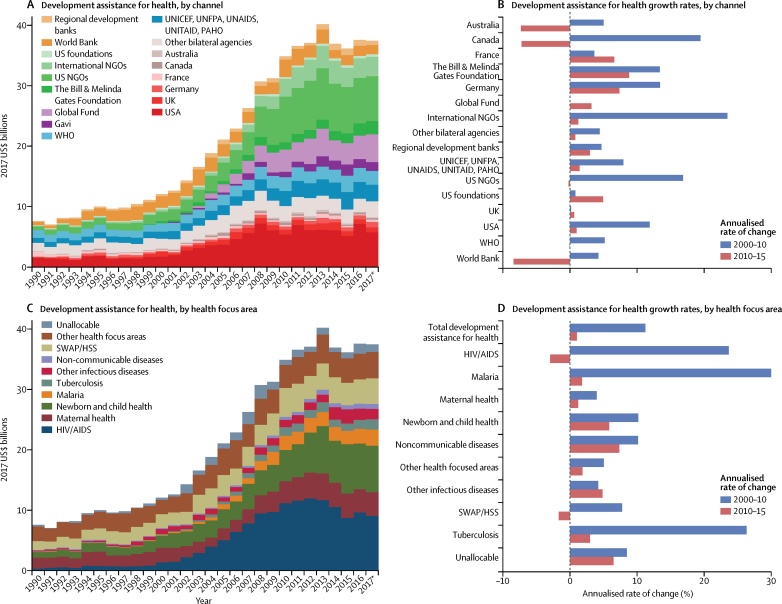
Development assistance for health by channel and health focus area, 1990–2017 Development assistance for health annual estimates and annualised growth rates, disaggregated by channel (A, B) and health focus area (C, D). Estimates are shown from 1990 to 2017, in billions of 2017 US$. Growth rates are shown for 2000–10, and 2010–17. Gavi channel annualised growth rates were excluded from panel B because of high annualised growth rates between 2000 and 2010, resulting from large increases in funding from the organisation's inception (in 2000) to 2010. World Bank includes the International Development Association and the International Bank for Reconstruction and Development; and regional development banks include the Inter-American Development Bank, the African Development Bank, and the Asian Development Bank. NGOs=non-governmental organisations. Global Fund=The Global Fund to Fight AIDS, Tuberculosis and Malaria. Gavi=Gavi, the Vaccine Alliance. UNFPA=United Nations Population Fund. UNAIDS=Joint United Nations Programme on HIV/AIDS. PAHO=Pan American Health Organization. SWAP/HSS=sector-wide approaches/health system strengthening. *Data for 2017 are preliminary estimates based on budget data and estimation.

Figure 2A shows that between 1990 and 2017, development assistance for health increased by 394·7% (from 7·6 billion to $37·4 billion), although this growth was not consistent throughout this period (figure 2B, 2D). From 1990 to 2000, the annualised growth rate was 4·8%, with development assistance for health reaching $12·0 billion in 2000. From 2000 to 2010, the annualised growth rate was 11·2%. Between 2010 and 2017, development assistance for health remained relatively constant (1·0% growth), peaking in 2013. We estimated the 2017 development assistance for health to be $37·4 billion (figure 2).

**Figure 3:**
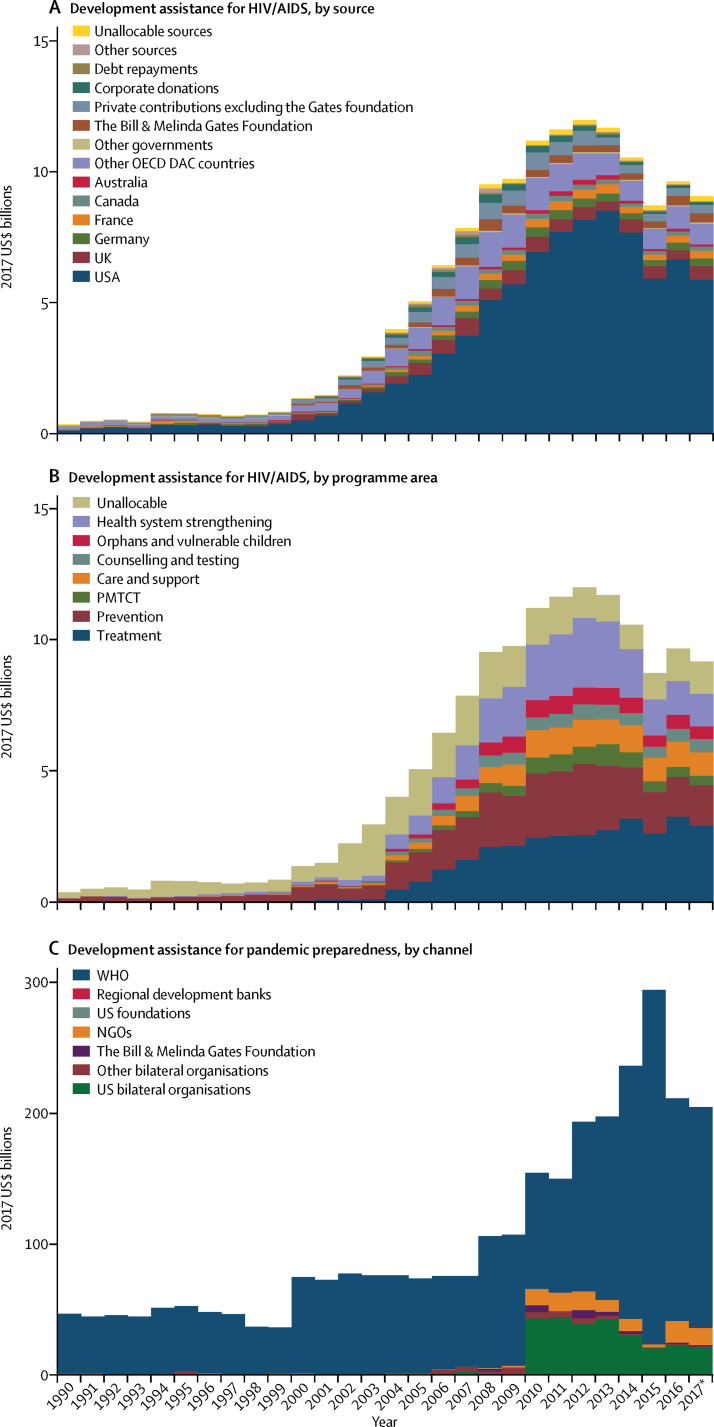
Development assistance for HIV/AIDS and pandemic preparedness, 1990–2017 Development assistance for HIV/AIDS by (A) source and (B) programme area, and for pandemic preparedness by channel (C). Spending is reported in 2017 US$. OECD=Organisation for Economic Co-operation and Development. DAC=Development Assistance Committee. PMTCT=prevention of mother-to-child transmission. NGOs=non-governmental organisations. *Data for 2017 are preliminary estimates based on budget data and estimation.

More development assistance for health was targeted at HIV/AIDS than at any other health focus area, with an estimated $9·1 billion spent in 2017 (figure 2C). This is a noteworthy increase (11·9% annualised growth rate) compared with spending on HIV/AIDS at the turn of the millennium and the onset of the Millennium Development Goals. Development assistance for HIV/AIDS reached its peak in 2012, at $12·0 billion, and has since declined by 24·3%. This finding stands in stark contrast with the growth observed between 2000 and 2012, which was 20·0% annually. The US Government was the largest source of development assistance for HIV/AIDS, providing more than 50% of this assistance each year since 2008 (figure 3A). Development assistance for HIV/AIDS is channelled through many international agencies, including international non-governmental organisations (7·3% in 2017) and the Global Fund (21·4% in 2017). In 2017, $2·9 billion (31·9%) of $9·1 billion of development assistance for HIV/AIDS was spent on treatment, and $1·5 billion (16·8%) was spent on prevention (excluding prevention of mother-to-child transmission of HIV/AIDS; figure 3B).

Development assistance for health that targeted other health focus areas was also substantial. We estimated that, in 2017, $7·7 billion of development assistance for health targeted newborn and child health, and $4·2 billion targeted sector-wide approaches and health system strengthening. There was substantially less development assistance targeting pandemic preparedness in 2017. We estimated this amount to be $204·2 million, with 82·6% ($168·7 million) of these funds channelled through WHO (figure 3C).

### Health spending on HIV/AIDS

We estimated that between 2000 and 2015, $562·6 billion (531·1 billion to 621·9 billion) was spent on HIV/AIDS. Global HIV/AIDS spending peaked at 49·7 billion (95% UI 46·2 billion to 54·7 billion) in 2013, but decreased slightly to $48·9 billion (45·2 billion to 54·2 billion) in 2015. Still, the 2015 total remains nearly three times that of spending in 2000 ($16·4 billion [14·6 billion to 19·3 billion]).

**Figure 4:**
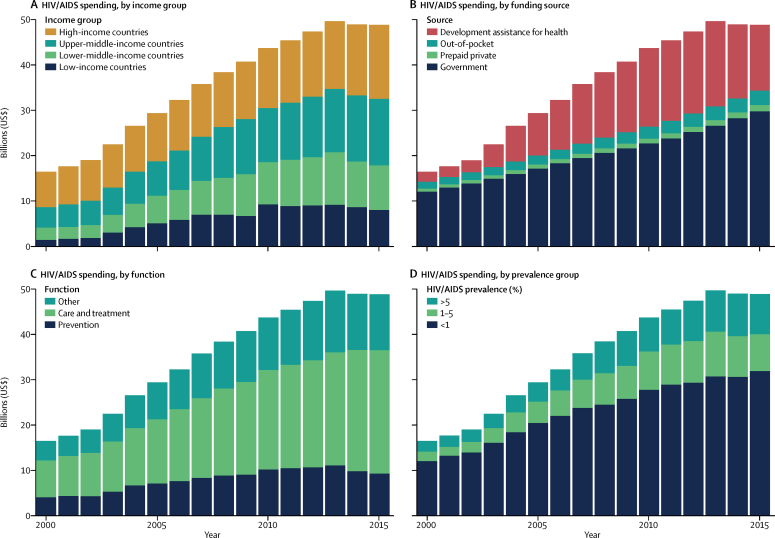
HIV/AIDS spending, 2000–15 HIV/AIDS spending by (A) income group, (B) source, (C) function, and (D) prevalence group. Spending is reported in 2017 purchasing-power parity-adjusted dollars. Income groups are based on 2017 World Bank income groups. Income group specifies where the resources were spent, not the source of the funds. HIV/AIDS prevalence data are from the Global Burden of Disease Study 2016.^21^

Most spending on HIV/AIDS occurs in high-income and upper-middle-income countries (figure 4A, table 2). In 2015, $16·3 billion (95% UI 14·5 billion to 18·4 billion) was spent on HIV/AIDS in high-income countries, and $14·7 billion (12·7 billion to 17·6 billion) was spent in upper-middle-income countries. Despite more people living with HIV/AIDS in lower-middle-income and low-income countries, these income groups have experienced reductions in HIV/AIDS spending between 2013 and 2015, whereas upper-middle-income and high-income countries' spending has continued to grow during these same years. By 2015, $9·8 billion (9·0 billion to 11·1 billion) was spent on HIV/AIDS in lower-middle-income countries, whereas $8·0 billion (7·8 billion to 8·6 billion) was spent in low-income countries.

**Table 2:** Health spending on HIV/AIDS, 2015

	**HIV/AIDS spending (millions of $)**	**HIV/AIDS spending per prevalent case ($)**	**Government HIV/AIDS spending as a share of total HIV/AIDS spending (%)**	**Prepaid private HIV/AIDS spending as a share of total HIV/AIDS spending (%)**	**Out-of-pocket HIV/AIDS spending as a share of total HIV/AIDS spending (%)**	**Development assistance for HIV/AIDS spending as a share of total HIV/AIDS spending (%)**	**Proportion of HIV/AIDS spending on curative care and treatment (%)**	**Proportion of HIV/AIDS spending on prevention (%)**
**Global**								
Total	48 863·9 (45 218·0 to 54 197·3)	1374·8 (1272·2 to 1524·8)	61·0% (55·1 to 65·1)	2·8% (0·9 to 6·9)	6·5% (3·5 to 10·5)	29·7% (26·7 to 32·0)	55·8% (53·3 to 57·9)	19·0% (17·6 to 20·6)
**World Bank income group**								
High-income countries	16 291·3 (14 538·9 to 18 382·7)	4869·9 (4346·1 to 5495·1)	86·3% (79·2 to 91·6)	3·6% (1·9 to 6·1)	10·1% (6·5 to 14·8)	0·0% (0·0 to 0·0)	62·4% (57·8 to 66·7)	16·0% (12·9 to 19·6)
Upper-middle-income countries	14 705·8 (12 657·5 to 17 569·0)	1509·6 (1299·3 to 1803·5)	79·8% (70·8 to 85·3)	3·5% (0·6 to 10·3)	4·5% (2·0 to 7·6)	12·1% (10·1 to 14·0)	60·3% (56·1 to 64·9)	16·7% (14·2 to 19·5)
Lower-middle-income countries	9841·3 (8972·5 to 11 159·1)	814·7 (742·8 to 923·8)	31·6% (26·5 to 37·9)	1·7% (0·3 to 5·6)	6·6% (2·6 to 12·7)	60·1% (52·8 to 65·7)	45·8% (43·0 to 49·4)	21·1% (19·2 to 23·3)
Low-income countries	8025·5 (7763·2 to 8557·2)	773·5 (748·2 to 824·7)	11·0% (9·4 to 13·0)	1·1% (0·1 to 4·1)	2·9% (1·4 to 5·3)	85·0% (79·7 to 87·8)	46·0% (45·0 to 47·1)	27·0% (25·9 to 28·0)
**Global Burden of Disease super-region**								
Central Europe, eastern Europe, and central Asia	2514·1 (2046·0 to 3383·9)	4106·0 (3341·5 to 5526·5)	74·0% (66·7 to 81·1)	0·7% (0·0 to 3·9)	2·0% (0·4 to 6·7)	23·4% (17·1 to 28·3)	42·8% (36·1 to 52·0)	28·8% (22·2 to 35·1)
Global Burden of Disease high income	16 131·5 (14 341·3 to 18 247·7)	4672·0 (4153·5 to 5284·8)	86·2% (79·1 to 91·5)	3·6% (2·0 to 6·1)	10·2% (6·5 to 14·9)	0·0% (0·0 to 0·0)	63·2% (58·6 to 67·5)	14·9% (11·9 to 18·4)
Latin America and Caribbean	5171·9 (4327·1 to 6483·8)	3991·1 (3339·2 to 5003·5)	69·2% (55·5 to 79·0)	6·7% (1·5 to 16·9)	11·6% (6·0 to 17·3)	12·5% (9·9 to 14·8)	62·3% (53·9 to 69·3)	18·4% (14·8 to 22·9)
North Africa and Middle East	1120·3 (982·2 to 1282·8)	10 152·5 (8901·0 to 11 625·0)	89·2% (85·2 to 91·5)	0·4% (0·0 to 2·1)	2·7% (1·1 to 5·2)	7·6% (6·6 to 8·7)	42·5% (35·1 to 50·0)	35·9% (27·6 to 44·8)
South Asia	2219·9 (1783·9 to 2931·2)	946·1 (760·3 to 1249·2)	43·5% (31·6 to 58·2)	3·7% (1·0 to 8·7)	7·6% (3·1 to 13·5)	45·2% (33·7 to 55·4)	33·3% (22·4 to 47·0)	18·3% (13·4 to 25·1)
Southeast Asia, east Asia, and Oceania	3730·1 (3376·1 to 4198·6)	1753·6 (1587·2 to 1973·8)	77·5% (75·1 to 80·0)	0·2% (0·0 to 0·5)	2·2% (1·2 to 3·7)	20·2% (17·9 to 22·2)	48·6% (38·8 to 59·2)	17·0% (12·8 to 23·1)
Sub-Saharan Africa	17 976·0 (16 297·9 to 20 523·7)	702·2 (636·7 to 801·8)	31·0% (24·6 to 39·0)	1·7% (0·2 to 6·3)	3·4% (1·4 to 6·6)	63·9% (55·7 to 70·2)	53·8% (51·7 to 56·5)	21·1% (19·3 to 22·6)
**Country**								
Afghanistan	33·8 (31·7 to 36·9)	10 743·2 (10 083·8 to 11 732·4)	5·2% (3·0 to 7·3)‖	0·4% (0·0 to 1·6)*	8·2% (3·2 to 14·5)*	86·2% (78·8 to 91·7)**	23·2% (21·3 to 25·7)*	40·1% (37·8 to 42·2)†
Albania	3·7 (2·6 to 5·3)	101 783·5 (71 342·1 to 147 039·3)	97·6% (93·5 to 99·4)§	0·2% (0·0 to 0·7)†	2·1% (0·5 to 5·7)†	0·1% (0·0 to 0·1)**	26·7% (10·7 to 50·1)*	52·1% (27·1 to 71·4)*
Algeria	53·2 (36·1 to 80·1)	8148·4 (5529·4 to 12 257·5)	99·0% (98·2 to 99·5)**	0·0% (0·0 to 0·1)†	0·6% (0·3 to 1·2)†	0·4% (0·2 to 0·5)**	83·5% (71·7 to 91·1)‡	12·4% (5·7 to 22·8)‡
Andorra	2·5 (1·9 to 3·3)	19 132·8 (14 426·4 to 25 004·6)	87·0% (76·6 to 94·2)*	0·4% (0·1 to 1·1)*	12·6% (5·6 to 22·4)*	0·0% (0·0 to 0·0)**	66·4% (53·8 to 77·6)*	14·5% (6·8 to 24·8)*
Angola	156·3 (123·5 to 204·8)	598·8 (473·2 to 784·6)	66·6% (58·3 to 75·0)**	0·5% (0·0 to 1·8)‡	1·0% (0·4 to 1·9)‡	31·9% (23·9 to 39·7)**	44·3% (26·9 to 63·0)*	12·9% (6·7 to 23·6)‡
Antigua and Barbuda	0·7 (0·5 to 0·9)	6496·6 (4834·4 to 8557·2)	91·3% (85·6 to 94·6)¶	0·9% (0·1 to 3·2)†	2·3% (0·6 to 5·3)†	5·5% (4·1 to 7·3)**	48·5% (30·2 to 65·2)*	23·8% (10·5 to 41·2)*
Argentina	678·5 (481·9 to 972·8)	4539·5 (3224·2 to 6508·2)	98·4% (97·7 to 99·0)**	0·1% (0·0 to 0·1)†	0·8% (0·4 to 1·2)†	0·8% (0·5 to 1·1)**	69·0% (58·4 to 78·4)*	4·2% (2·2 to 7·0)‡
Armenia	9·5 (8·1 to 12·1)	14 952·1 (12 655·4 to 18 949·8)	52·4% (44·5 to 62·7)**	0·0% (0·0 to 0·0)‡	0·6% (0·3 to 1·2)‡	47·0% (36·7 to 54·9)**	26·7% (14·3 to 45·2)*	34·2% (17·1 to 50·1)*
Australia	365·0 (295·5 to 454·9)	10 868·4 (8797·8 to 13 543·4)	91·8% (85·4 to 96·2)†	1·1% (0·3 to 2·4)*	7·1% (3·4 to 12·1)*	0·0% (0·0 to 0·0)**	61·4% (48·5 to 73·4)*	17·7% (8·7 to 29·4)*
Austria	203·1 (157·3 to 266·8)	8656·8 (6704·2 to 11 372·3)	91·8% (83·6 to 96·5)*	0·6% (0·2 to 1·5)*	7·6% (3·3 to 15·0)*	0·0% (0·0 to 0·0)**	63·9% (51·1 to 75·1)*	15·1% (7·5 to 26·5)*
Azerbaijan	32·8 (24·8 to 44·9)	11 343·6 (8591·2 to 15 539·5)	56·9% (42·1 to 69·1)**	0·0% (0·0 to 0·1)*	7·9% (1·4 to 23·3)*	35·1% (25·1 to 45·3)**	27·8% (15·8 to 47·6)†	47·5% (24·5 to 58·3)†
Bahrain	6·3 (4·1 to 9·7)	24 476·1 (15 720·5 to 37 611·8)	98·0% (94·0 to 99·6)*	0·4% (0·0 to 1·9)*	1·6% (0·4 to 4·2)*	0·0% (0·0 to 0·0)**	56·1% (35·2 to 74·6)*	24·5% (10·1 to 44·6)*
Bangladesh	52·3 (48·1 to 59·3)	8681·1 (7981·8 to 9841·2)	26·1% (20·4 to 34·4)**	0·5% (0·1 to 1·3)†	1·9% (0·8 to 4·1)†	71·5% (62·9 to 77·6)**	19·4% (16·4 to 24·5)*	32·3% (26·4 to 37·5)*
Barbados	5·3 (3·4 to 8·0)	5058·6 (3262·7 to 7627·4)	89·6% (74·7 to 97·1)¶	1·2% (0·2 to 4·6)†	9·1% (2·7 to 21·0)†	0·0% (0·0 to 0·0)**	49·2% (36·3 to 61·4)§	25·8% (15·8 to 37·5)‖
Belarus	202·7 (163·6 to 257·0)	15 254·4 (12 315·6 to 19 341·4)	66·4% (58·9 to 73·7)**	0·6% (0·1 to 1·8)†	3·6% (1·9 to 6·0)†	29·4% (22·9 to 36·0)**	27·2% (18·8 to 38·7)¶	42·0% (26·1 to 54·8)‖
Belgium	239·1 (173·2 to 353·3)	10 581·3 (7665·3 to 15 633·2)	96·7% (93·2 to 98·6)‡	0·0% (0·0 to 0·0)*	3·3% (1·4 to 6·8)*	0·0% (0·0 to 0·0)**	62·4% (49·6 to 74·0)*	17·9% (9·0 to 29·5)*
Belize	9·9 (9·2 to 10·9)	4326·1 (3985·6 to 4763·6)	28·8% (23·5 to 34·5)¶	0·9% (0·1 to 3·2)†	2·6% (0·8 to 5·6)†	67·6% (61·3 to 73·3)**	20·1% (17·6 to 23·5)†	35·1% (30·6 to 38·3)‡
Benin	47·7 (42·2 to 56·9)	621·5 (550·0 to 741·6)	21·4% (12·3 to 34·8)**	0·0% (0·0 to 0·1)‖	0·9% (0·0 to 4·3)**	77·7% (64·7 to 87·3)**	34·1% (32·6 to 35·9)‡	32·7% (29·9 to 34·4)‡
Bhutan	6·8 (6·1 to 8·3)	12 174·0 (10 989·8 to 14 860·4)	30·9% (23·9 to 43·5)‡	0·1% (0·0 to 0·3)*	0·4% (0·1 to 1·1)*	68·7% (55·9 to 75·6)**	29·4% (24·4 to 37·7)*	19·4% (13·5 to 26·0)*
Bolivia	32·9 (25·7 to 44·0)	2677·2 (2089·1 to 3586·9)	50·5% (37·6 to 63·8)**	0·3% (0·1 to 1·1)§	6·4% (1·4 to 16·5)§	42·7% (31·3 to 53·7)**	26·4% (16·3 to 39·2)§	30·2% (20·0 to 41·1)§
Bosnia and Herzegovina	18·2 (13·8 to 25·2)	247 120·4 (187 132·5 to 342 581·9)	51·9% (38·0 to 66·2)§	0·0% (0·0 to 0·2)†	0·5% (0·2 to 1·3)†	47·6% (33·5 to 61·3)**	41·7% (35·8 to 51·3)*	38·6% (27·2 to 48·4)*
Botswana	580·7 (450·1 to 742·0)	1648·3 (1277·6 to 2106·2)	73·1% (61·6 to 79·9)**	2·6% (0·0 to 14·6)**	0·5% (0·0 to 1·9)‖	23·8% (18·3 to 30·2)**	52·6% (42·4 to 65·2)§	7·7% (6·4 to 9·7)§
Brazil	2161·2 (1513·4 to 3082·4)	4264·8 (2986·5 to 6082·9)	75·8% (53·9 to 91·6)‖	12·1% (2·4 to 29·6)*	11·8% (5·5 to 17·2)*	0·3% (0·2 to 0·4)**	72·0% (53·3 to 84·2)¶	17·7% (10·0 to 29·8)¶
Brunei	3·7 (3·1 to 4·5)	3149·3 (2668·3 to 3889·2)	96·9% (94·0 to 98·6)*	0·3% (0·1 to 0·8)*	2·8% (1·3 to 5·2)*	0·0% (0·0 to 0·0)**	71·1% (58·5 to 81·8)*	12·6% (6·1 to 22·6)*
Bulgaria	29·3 (21·9 to 40·1)	23 730·8 (17 696·9 to 32 441·6)	71·6% (62·8 to 79·5)**	0·0% (0·0 to 0·2)*	3·4% (0·9 to 8·6)*	24·9% (17·8 to 32·6)**	37·4% (22·2 to 56·9)*	42·9% (24·9 to 60·4)*
Burkina Faso	84·5 (68·8 to 107·9)	839·8 (684·1 to 1073·1)	31·5% (19·8 to 45·7)**	0·2% (0·0 to 1·4)**	11·0% (3·6 to 23·1)**	57·3% (44·2 to 69·4)**	33·8% (29·8 to 38·1)¶	30·3% (25·2 to 34·8)**
Burundi	74·8 (71·3 to 80·5)	865·2 (824·3 to 931·7)	7·7% (3·7 to 13·9)**	1·2% (0·1 to 4·8)¶	0·2% (0·1 to 0·3)¶	90·9% (84·4 to 95·3)**	20·5% (18·7 to 23·2)§	35·9% (33·9 to 37·6)‡
Cambodia	130·7 (123·3 to 142·1)	1938·7 (1829·2 to 2108·3)	14·1% (9·2 to 21·0)**	0·0% (0·0 to 0·0)‖	0·9% (0·1 to 3·1)‖	85·0% (78·1 to 90·0)**	26·8% (24·2 to 30·2)‖	32·5% (30·6 to 34·6)*
Cameroon	145·6 (130·4 to 171·3)	251·1 (224·8 to 295·4)	17·3% (9·0 to 29·4)**	1·0% (0·0 to 6·2)‖	1·0% (0·1 to 3·2)‖	80·7% (68·3 to 89·7)**	49·8% (48·1 to 51·2)†	21·0% (19·0 to 22·7)‡
Canada	686·8 (577·4 to 843·2)	6127·3 (5151·8 to 7522·9)	93·9% (90·6 to 96·3)*	1·2% (0·5 to 2·4)*	4·9% (3·2 to 7·1)*	0·0% (0·0 to 0·0)**	63·6% (51·1 to 75·9)*	15·0% (7·2 to 25·3)*
Cape Verde	5·8 (5·2 to 6·8)	1479·9 (1331·7 to 1743·6)	37·5% (30·8 to 47·3)**	0·1% (0·0 to 0·7)§	0·5% (0·0 to 1·9)§	61·9% (52·3 to 68·4)**	41·9% (33·7 to 51·4)*	23·4% (18·0 to 30·1)*
Central African Republic	13·4 (12·1 to 15·8)	106·6 (96·0 to 125·5)	13·9% (6·3 to 26·3) ‖	1·3% (0·0 to 8·1)‡	1·0% (0·1 to 3·9)‡	83·8% (70·8 to 92·6)**	14·3% (11·3 to 19·4)*	43·9% (41·0 to 46·7)*
Chad	39·8 (30·4 to 53·2)	193·9 (148·1 to 258·9)	48·5% (34·7 to 62·6)‖	0·6% (0·0 to 3·6)‡	1·4% (0·2 to 4·8)§	49·4% (36·2 to 63·4)**	38·6% (29·4 to 45·2)¶	35·2% (31·2 to 41·7)§
Chile	245·0 (176·2 to 341·7)	8323·2 (5986·5 to 11 605·1)	77·7% (67·7 to 85·6)¶	0·4% (0·2 to 0·8)‡	21·8% (14·2 to 31·5)‡	0·0% (0·0 to 0·0)**	66·8% (52·4 to 80·5)*	29·6% (15·5 to 44·3)*
China	1478·1 (1196·7 to 1860·5)	2214·4 (1792·9 to 2787·2)	98·4% (96·9 to 99·1)**	0·1% (0·0 to 0·5)‡	0·8% (0·2 to 2·0)‡	0·7% (0·6 to 0·9)**	54·6% (29·7 to 78·8)†	15·8% (5·7 to 31·8)‡
Colombia	303·0 (211·2 to 423·2)	4503·7 (3140·1 to 6290·6)	64·3% (46·1 to 79·7)**	10·6% (3·0 to 23·0)§	19·4% (11·3 to 27·2)§	5·6% (3·9 to 7·8)**	80·7% (71·0 to 87·8)‡	6·9% (4·1 to 11·1)¶
Comoros	1·3 (1·1 to 1·9)	3516·3 (2775·2 to 4870·8)	27·9% (14·1 to 44·2)**	1·1% (0·0 to 5·6)†	10·2% (1·9 to 25·1)†	60·8% (43·0 to 75·4)**	27·9% (20·9 to 39·0)*	30·8% (22·6 to 39·6)*
Congo (Brazzaville)	46·8 (35·6 to 63·0)	573·4 (436·2 to 772·4)	71·5% (62·6 to 79·5)‖	2·4% (0·6 to 6·3)†	1·7% (1·2 to 2·2)†	24·4% (17·7 to 31·4)**	39·9% (21·8 to 60·8)*	23·5% (13·4 to 38·6)*
Costa Rica	55·8 (41·6 to 73·7)	8887·3 (6627·6 to 11 743·9)	78·1% (62·6 to 88·7)‖	1·8% (0·4 to 5·0)‡	15·4% (6·3 to 28·4)‡	4·8% (3·5 to 6·2)**	55·5% (42·5 to 67·0)‡	28·8% (19·3 to 38·8)‡
Côte d'Ivoire	225·7 (208·8 to 252·6)	437·5 (404·9 to 489·7)	13·9% (8·5 to 20·8)**	1·8% (0·1 to 8·9)**	1·1% (0·2 to 2·6)**	83·2% (74·2 to 89·7)**	53·9% (49·8 to 58·3)‖	24·5% (20·0 to 29·8)§
Croatia	18·8 (12·4 to 27·3)	56 397·3 (36 996·8 to 81 800·2)	99·1% (97·5 to 99·8)¶	0·3% (0·0 to 1·2)*	0·5% (0·2 to 1·3)*	0·0% (0·0 to 0·0)**	38·8% (16·9 to 63·6)*	35·3% (14·7 to 59·5)*
Cuba	216·6 (167·9 to 283·1)	8583·1 (6651·2 to 11 214·5)	88·6% (85·5 to 91·5)**	0·2% (0·0 to 0·6)*	0·4% (0·1 to 0·8)*	10·9% (8·2 to 13·8)**	42·5% (28·1 to 59·1)*	30·1% (15·5 to 45·9)*
Cyprus	8·6 (6·6 to 11·5)	6622·7 (5076·9 to 8852·1)	90·7% (81·7 to 96·0)*	0·5% (0·1 to 1·3)*	8·8% (3·9 to 17·1)*	0·0% (0·0 to 0·0)**	58·9% (45·4 to 71·3)*	19·0% (9·4 to 31·7)*
Czech Republic	104·5 (63·7 to 170·6)	89 541·0 (54 589·6 to 146 222·7)	99·1% (98·2 to 99·6)‡	0·1% (0·0 to 0·2)†	0·8% (0·4 to 1·6)†	0·0% (0·0 to 0·0)**	42·9% (18·0 to 68·2)*	33·7% (12·2 to 60·6)*
Democratic Republic of the Congo	256·8 (236·6 to 286·9)	604·1 (556·7 to 675·0)	16·6% (10·3 to 25·4)**	0·8% (0·0 to 6·0)**	0·3% (0·0 to 1·5)¶	82·3% (73·4 to 89·0)**	49·2% (46·6 to 52·2)*	25·2% (22·4 to 28·6)*
Denmark	123·9 (97·8 to 166·0)	10 663·8 (8413·4 to 14 287·5)	95·6% (90·5 to 98·3)*	0·1% (0·0 to 0·4)*	4·3% (1·7 to 9·0)*	0·0% (0·0 to 0·0)**	63·2% (49·2 to 75·6)*	15·6% (7·4 to 27·9)*
Djibouti	13·3 (12·6 to 14·1)	1115·3 (1060·2 to 1186·0)	10·4% (6·5 to 14·8)¶	0·2% (0·0 to 0·9)†	1·6% (0·2 to 5·7)†	87·9% (82·6 to 92·4)**	73·7% (70·3 to 76·8)*	13·7% (11·8 to 17·0)†
Dominica	0·9 (0·8 to 1·1)	12 458·0 (11 086·7 to 14 557·6)	27·8% (19·7 to 38·0)§	0·1% (0·0 to 0·6)*	1·7% (0·4 to 4·7)*	70·4% (60·0 to 78·7)**	35·5% (30·6 to 42·1)*	31·9% (27·3 to 37·4)*
Dominican Republic	153·8 (118·2 to 198·4)	1686·5 (1296·7 to 2176·3)	30·4% (20·2 to 42·1)‖	5·0% (1·5 to 11·5)§	31·6% (21·3 to 40·4)‡	32·9% (25·1 to 42·1)**	38·6% (26·7 to 50·9)†	25·4% (19·3 to 32·4)†
Ecuador	37·8 (27·6 to 51·8)	1566·9 (1143·8 to 2146·7)	66·5% (48·1 to 78·3)**	0·1% (0·0 to 0·5)‡	15·4% (4·3 to 36·5)†	17·9% (12·7 to 23·9)**	47·7% (36·3 to 62·5)‡	13·8% (9·8 to 18·6)‡
Egypt	59·4 (48·7 to 72·7)	13 693·4 (11 235·3 to 16 753·1)	83·8% (73·2 to 89·0)§	1·3% (0·0 to 6·5)‡	5·3% (1·4 to 12·0)†	9·5% (7·7 to 11·5)**	44·9% (27·3 to 62·7)†	27·5% (12·2 to 45·9)†
El Salvador	118·8 (95·1 to 148·2)	9152·8 (7329·9 to 11 421·1)	76·9% (69·1 to 82·7)**	0·5% (0·1 to 1·8)‖	5·0% (1·7 to 11·5)**	17·5% (13·9 to 21·6)**	55·7% (45·7 to 63·6)‖	29·2% (23·9 to 36·5)‖
Equatorial Guinea	10·0 (5·4 to 16·8)	328·7 (176·7 to 551·6)	90·2% (75·3 to 96·5)§	1·1% (0·1 to 5·5)‡	5·8% (1·4 to 15·6)‡	2·9% (1·6 to 5·0)**	58·2% (27·1 to 83·8)*	11·3% (4·8 to 25·7)*
Eritrea	12·1 (10·4 to 14·6)	473·5 (407·9 to 571·2)	33·6% (24·7 to 42·9)¶	0·8% (0·0 to 5·1)†	4·6% (0·7 to 13·1)†	60·9% (50·2 to 70·2)**	27·2% (19·9 to 37·9)*	37·4% (29·1 to 45·4)*
Estonia	25·8 (17·6 to 37·1)	20 784·5 (14 171·9 to 29 902·1)	99·7% (98·8 to 100·0)†	0·0% (0·0 to 0·2)*	0·3% (0·0 to 1·0)*	0·0% (0·0 to 0·0)**	49·6% (28·1 to 69·5)*	23·1% (7·1 to 43·7)*
Ethiopia	870·2 (823·5 to 944·3)	1318·9 (1248·1 to 1431·2)	9·9% (4·9 to 16·9)**	0·1% (0·0 to 0·6)¶	0·4% (0·1 to 1·2)§	89·6% (82·5 to 94·6)**	46·7% (45·2 to 49·2)†	25·0% (23·3 to 27·0)*
Federated States of Micronesia	0·4 (0·3 to 0·5)	931·3 (735·6 to 1245·7)	42·7% (30·5 to 55·1)*	4·8% (0·5 to 10·9)*	7·1% (1·1 to 19·2)*	45·4% (33·4 to 56·5)**	46·2% (41·7 to 51·0)§	20·0% (13·1 to 27·3)*
Fiji	1·6 (0·7 to 3·1)	3775·5 (1750·5 to 7576·3)	48·5% (19·3 to 81·0)¶	45·6% (14·1 to 73·7)†	4·1% (1·8 to 5·9)*	1·8% (0·8 to 3·3)**	37·0% (22·1 to 52·7)§	26·3% (10·6 to 43·7)*
Finland	48·2 (36·7 to 65·2)	17 746·3 (13 510·7 to 23 992·1)	94·4% (88·5 to 97·9)*	0·2% (0·0 to 0·6)*	5·4% (2·1 to 10·9)*	0·0% (0·0 to 0·0)**	57·4% (44·3 to 71·0)*	22·4% (11·2 to 36·8)*
France	919·6 (720·3 to 1205·8)	16 370·3 (12 822·3 to 21 466·4)	94·4% (88·8 to 97·7)*	2·0% (0·6 to 4·9)*	3·5% (1·7 to 6·3)*	0·0% (0·0 to 0·0)**	58·7% (43·8 to 71·3)*	19·4% (9·9 to 32·4)*
Gabon	21·5 (13·1 to 34·8)	509·8 (310·7 to 823·8)	87·8% (63·4 to 97·1)**	6·3% (0·3 to 24·9)¶	4·3% (1·0 to 10·0)¶	1·7% (1·0 to 2·6)**	33·0% (10·9 to 60·8)‡	31·6% (13·6 to 57·0)§
Georgia	47·9 (41·9 to 56·8)	44 330·7 (38 741·0 to 52 586·8)	36·0% (27·3 to 45·6)**	0·1% (0·0 to 0·5)‖	3·9% (0·5 to 13·8)‖	60·0% (50·3 to 68·3)**	38·8% (29·4 to 52·6)¶	36·6% (23·7 to 50·3)§
Germany	1345·4 (1007·4 to 1897·9)	8722·1 (6531·3 to 12 304·2)	94·2% (87·7 to 97·8)*	0·2% (0·1 to 0·7)*	5·6% (2·2 to 11·6)*	0·0% (0·0 to 0·0)**	62·9% (48·7 to 74·7)*	19·4% (9·9 to 31·9)*
Ghana	372·3 (247·2 to 619·9)	1188·9 (789·5 to 1979·5)	19·4% (7·8 to 34·7)**	3·7% (0·1 to 16·5)**	31·4% (11·8 to 47·7)‖	45·4% (25·8 to 64·8)**	51·3% (38·4 to 65·4)‡	22·5% (15·4 to 29·9)‡
Greece	96·3 (64·0 to 148·4)	11 023·3 (7325·0 to 16 983·0)	95·3% (90·8 to 97·9)†	0·1% (0·0 to 0·4)*	4·5% (2·1 to 8·8)*	0 0·0% (0·0 to 0·0)**	55·0% (41·3 to 68·3)*	22·7% (12·0 to 37·0)*
Grenada	1·0 (0·9 to 1·2)	8627·8 (7630·3 to 9912·5)	34·9% (28·1 to 41·8)‡	0·7% (0·1 to 2·5)*	5·1% (1·3 to 12·5)*	59·3% (51·4 to 66·8)**	36·7% (29·4 to 44·1)*	31·6% (25·7 to 39·3)*
Guatemala	107·1 (96·5 to 119·4)	4269·0 (3845·6 to 4758·9)	64·1% (59·7 to 68·2)**	1·8% (0·7 to 3·8)**	5·9% (4·1 to 8·1)**	28·2% (25·2 to 31·2)**	43·9% (33·1 to 54·4)**	30·3% (20·3 to 40·9)**
Guinea	44·3 (39·7 to 52·9)	377·7 (338·5 to 451·2)	16·6% (9·5 to 25·8)**	2·2% (0·1 to 10·6)¶	2·2% (0·2 to 8·4)¶	79·0% (65·8 to 87·7)**	33·6% (30·3 to 37·6)†	38·1% (34·9 to 41·3)‡
Guinea-Bissau	15·5 (14·8 to 16·8)	373·0 (355·8 to 403·4)	9·5% (5·4 to 16·4)**	0·1% (0·0 to 0·9)‡	0·4% (0·0 to 2·0)‡	89·9% (83·0 to 94·2)**	35·6% (34·3 to 37·7)*	39·3% (37·3 to 41·0)†
Guyana	18·6 (15·7 to 23·1)	1222·3 (1030·0 to 1518·2)	29·2% (17·9 to 42·3)§	0·0% (0·0 to 0·1)‡	5·8% (1·2 to 17·5)‡	65·0% (51·8 to 76·4)**	41·6% (36·0 to 47·6)*	19·6% (15·2 to 26·0)*
Haiti	371·4 (367·8 to 376·1)	2598·6 (2573·5 to 2631·4)	2·8% (1·9 to 4·1)**	0·0% (0·0 to 0·1)†	0·1% (0·0 to 0·2)†	97·1% (95·9 to 98·0)**	56·6% (56·2 to 57·0)*	14·4% (14·0 to 15·0)†
Honduras	58·3 (48·2 to 70·0)	3239·7 (2674·3 to 3885·4)	51·1% (41·5 to 59·8)**	3·1% (1·2 to 6·2)**	13·9% (9·9 to 18·2)**	31·9% (26·3 to 38·2)**	34·8% (27·2 to 43·7)§	39·9% (32·1 to 48·0)‡
Hungary	27·9 (18·6 to 41·2)	25 407·7 (16 909·4 to 37 581·3)	89·4% (70·1 to 97·9)‡	1·3% (0·1 to 5·5)*	9·3% (2·0 to 24·5)*	0·0% (0·0 to 0·0)**	40·6% (17·9 to 65·4)*	35·2% (13·7 to 62·5)*
Iceland	5·2 (4·1 to 7·3)	9092·5 (7103·8 to 12 671·9)	93·9% (87·5 to 97·5)*	0·3% (0·1 to 0·7)*	5·8% (2·5 to 11·8)*	0·0% (0·0 to 0·0)**	61·6% (47·9 to 74·1)*	16·6% (8·2 to 29·1)*
India	1946·7 (1515·0 to 2659·9)	859·1 (668·6 to 1173·9)	46·4% (33·0 to 62·0)**	3·9% (1·0 to 9·4)‡	8·3% (3·3 to 14·9)‡	41·3% (29·7 to 52·1)**	34·8% (22·3 to 50·0)*	15·6% (10·7 to 23·1)‡
Indonesia	380·7 (339·0 to 423·0)	1946·3 (1733·3 to 2162·6)	56·5% (51·4 to 61·0)**	0·0% (0·0 to 0·0)‡	0·1% (0·1 to 0·1)‡	43·4% (38·9 to 48·5)**	40·3% (36·1 to 45·0)¶	17·5% (14·0 to 22·9)†
Iran	292·2 (222·0 to 374·1)	22 162·8 (16 842·0 to 28 377·8)	90·8% (87·6 to 93·2)‖	0·2% (0·0 to 0·9)‡	1·0% (0·2 to 2·4)‡	8·0% (6·2 to 10·4)**	20·7% (9·5 to 37·3)‡	51·8% (31·4 to 69·4)†
Iraq	24·2 (16·0 to 37·3)	5913·1 (3910·6 to 9098·0)	91·2% (75·8 to 98·0)*	0·0% (0·0 to 0·0)*	8·6% (1·9 to 24·0)*	0·2% (0·1 to 0·2)**	42·4% (24·5 to 61·4)*	36·3% (18·6 to 57·0)*
Ireland	73·1 (55·6 to 102·3)	14 117·0 (10 737·7 to 19 751·9)	91·7% (83·3 to 96·7)*	1·3% (0·3 to 3·5)*	6·9% (3·0 to 13·0)*	0·0% (0·0 to 0·0)**	61·9% (47·5 to 75·4)*	17·7% (8·8 to 31·2)*
Israel	59·1 (42·9 to 81·7)	4966·2 (3605·3 to 6872·0)	85·9% (74·5 to 93·6)*	1·3% (0·4 to 3·3)*	12·8% (6·0 to 22·3)*	0·0% (0·0 to 0·0)**	58·9% (45·6 to 71·6)*	21·0% (11·2 to 34·6)*
Italy	1169·6 (912·8 to 1607·1)	4601·9 (35 91·7 to 6323·7)	91·0% (82·8 to 96·1)†	0·2% (0·1 to 0·6)*	8·7% (3·8 to 16·6)*	0·0% (0·0 to 0·0)**	62·5% (49·3 to 74·5)*	14·7% (7·3 to 25·4)*
Jamaica	35·2 (28·4 to 45·2)	2977·7 (2405·7 to 3822·7)	36·3% (24·8 to 48·6)**	4·1% (0·4 to 14·0)§	4·8% (1·3 to 10·3)§	54·8% (42·1 to 66·9)**	27·5% (25·0 to 30·9)†	48·3% (43·5 to 52·5)‡
Japan	713·7 (596·8 to 837·3)	17 479·9 (14 616·5 to 20 506·5)	83·8% (72·4 to 92·0)‡	0·0% (0·0 to 0·0)*	16·2% (8·0 to 27·6)*	0·0% (0·0 to 0·0)**	40·4% (25·6 to 56·3)‡	19·9% (10·1 to 33·6)‡
Jordan	4·8 (3·9 to 6·3)	44 042·1 (36 428·4 to 57 850·4)	43·2% (32·7 to 57·4)‖	0·6% (0·0 to 2·9)*	1·2% (0·3 to 2·6)*	55·1% (41·3 to 65·6)**	23·4% (16·1 to 34·1)*	54·3% (44·6 to 61·5)*
Kazakhstan	72·6 (51·8 to 107·1)	6100·8 (4357·8 to 9004·6)	74·9% (65·8 to 83·5)**	0·0% (0·0 to 0·0)†	0·7% (0·3 to 1·2)†	24·4% (16·0 to 33·0)**	42·5% (21·1 to 67·1)*	35·3% (11·3 to 61·1)*
Kenya	1911·0 (1687·5 to 2257·1)	1165·0 (1028·7 to 1376·0)	15·6% (8·3 to 25·5)**	2·0% (0·3 to 6·4)‖	10·4% (4·4 to 17·7)¶	72·0% (60·6 to 81·0)**	59·6% (52·2 to 67·3)¶	18·6% (14·4 to 26·4)§
Kiribati	0·2 (0·1 to 0·2)	24 346·4 (18 305·7 to 34 215·6)	55·1% (40·9 to 69·3)†	0·3% (0·0 to 1·4)*	2·1% (0·2 to 8·1)*	42·5% (29·4 to 55·0)**	18·4% (13·2 to 27·1)†	50·0% (40·3 to 56·8)†
Kuwait	14·4 (7·0 to 27·7)	196 260·6 (94 715·9 to 376 046·5)	99·8% (99·4 to 100·0)¶	0·0% (0·0 to 0·1)*	0·2% (0·0 to 0·5)*	0·0% (0·0 to 0·0)**	52·0% (30·2 to 72·9)*	32·3% (13·1 to 54·7)*
Kyrgyzstan	51·3 (46·1 to 58·6)	10 026·7 (9022·5 to 11 463·4)	29·7% (23·0 to 37·8)**	0·1% (0·0 to 0·6)†	2·6% (0·3 to 9·4)†	67·6% (58·9 to 74·9)**	20·3% (16·2 to 30·1)†	41·7% (30·5 to 50·3)*
Laos	10·9 (10·2 to 12·3)	1329·4 (1247·5 to 1497·2)	13·5% (8·0 to 23·3)**	0·0% (0·0 to 0·0)†	0·3% (0·2 to 0·5)†	86·2% (76·4 to 91·7)**	24·6% (22·5 to 27·8)†	37·1% (36·0 to 38·2)†
Latvia	9·7 (6·2 to 14·3)	5063·6 (3243·9 to 7478·9)	99·5% (98·8 to 99·9)¶	0·0% (0·0 to 0·1)†	0·4% (0·1 to 1·1)†	0·0% (0·0 to 0·0)**	48·2% (26·9 to 68·0)*	24·7% (8·3 to 46·3)*
Lebanon	13·4 (9·2 to 19·4)	11 941·0 (8221·3 to 17 309·9)	89·7% (77·4 to 95·0)§	2·4% (0·1 to 11·6)*	3·1% (0·9 to 6·3)*	4·8% (3·2 to 6·8)**	47·1% (29·9 to 65·1)*	27·4% (12·6 to 46·9)*
Lesotho	190·1 (162·5 to 227·6)	618·4 (528·7 to 740·3)	29·8% (18·6 to 41·8)**	0·0% (0·0 to 0·1)§	0·1% (0·1 to 0·2)§	70·0% (58·1 to 81·3)**	52·3% (47·5 to 57·3)‡	21·9% (19·9 to 24·1)‡
Liberia	70·0 (64·7 to 77·3)	1990·5 (1840·6 to 2199·1)	2·6% (0·8 to 5·2)‖	2·8% (0·1 to 8·8)‡	7·3% (3·4 to 10·1)†	87·3% (78·8 to 94·2)**	35·1% (33·1 to 37·3)*	29·4% (27·3 to 31·3)*
Libya	4·9 (3·3 to 7·4)	5827·6 (3905·4 to 8845·8)	90·1% (79·1 to 95·3)*	1·3% (0·0 to 6·8)*	3·8% (0·9 to 9·0)*	4·8% (3·0 to 6·8)**	42·4% (24·8 to 60·4)*	33·3% (15·9 to 52·5)*
Lithuania	7·2 (4·9 to 10·1)	7580·4 (5110·7 to 10 663·7)	92·8% (73·5 to 99·4)†	0·5% (0·0 to 2·7)*	6·7% (0·6 to 23·8)*	0·0% (0·0 to 0·0)**	45·5% (25·1 to 66·1)*	29·0% (9·9 to 52·1)*
Luxembourg	15·4 (12·1 to 21·2)	11 596·6 (9111·6 to 15 943·7)	96·3% (92·3 to 98·5)*	0·3% (0·1 to 0·7)*	3·4% (1·4 to 7·0)*	0·0% (0·0 to 0·0)**	70·5% (58·1 to 81·0)*	11·9% (5·6 to 21·2)*
Macedonia	13·7 (12·0 to 16·7)	312 938·9 (273 776·1 to 381 145·2)	28·9% (19·2 to 42·2)§	0·1% (0·0 to 0·2)†	0·5% (0·2 to 1·1)†	70·5% (57·5 to 80·1)**	35·5% (31·2 to 43·2)*	33·8% (27·0 to 39·9)*
Madagascar	30·7 (22·8 to 42·9)	825·9 (613·3 to 1154·8)	60·3% (47·6 to 72·3)**	0·6% (0·0 to 3·6)§	0·8% (0·1 to 2·7)§	38·4% (26·7 to 50·3)**	17·8% (10·1 to 32·0)†	41·4% (28·2 to 58·5)†
Malawi	837·4 (777·3 to 929·2)	691·0 (641·5 to 766·8)	11·6% (7·1 to 16·8)**	1·9% (0·1 to 7·0)‖	2·7% (0·5 to 6·3)**	83·8% (75·4 to 90·1)**	44·1% (42·1 to 47·0)‡	26·5% (24·2 to 28·9)*
Malaysia	127·2 (101·2 to 160·4)	3267·6 (2601·3 to 4122·4)	95·0% (93·5 to 96·1)**	0·1% (0·0 to 0·2)§	1·1% (0·6 to 1·9)§	3·8% (3·0 to 4·8)**	56·9% (45·3 to 67·5)‖	20·2% (14·4 to 27·1)‖
Maldives	1·6 (1·2 to 2·2)	96482·6 (71 059·1 to 137 089·5)	60·2% (47·7 to 72·9)†	0·0% (0·0 to 0·0)*	0·2% (0·0 to 0·6)*	39·6% (27·0 to 52·2)**	35·7% (25·1 to 49·5)*	25·6% (16·5 to 36·2)*
Mali	71·0 (65·5 to 78·5)	598·3 (551·2 to 661·4)	21·6% (15·2 to 28·9)**	0·4% (0·0 to 1·8)‖	0·7% (0·1 to 2·5)‖	77·4% (69·8 to 83·8)**	22·1% (18·9 to 25·5)¶	26·5% (24·4 to 29·3)¶
Malta	5·8 (4·3 to 7·9)	7445·0 (5511·4 to 10 111·5)	85·1% (72·8 to 93·6)*	0·2% (0·1 to 0·5)*	14·7% (6·3 to 26·6)*	0·0% (0·0 to 0·0)**	59·4% (46·4 to 72·0)*	18·2% (9·0 to 30·0)*
Marshall Islands	0·3 (0·2 to 0·5)	7684·5 (4789·2 to 13 541·2)	51·0% (24·9 to 71·3)¶	20·7% (3·5 to 47·2)*	7·7% (1·9 to 14·9)*	20·6% (10·9 to 30·8)**	25·8% (18·1 to 34·7)¶	43·8% (34·0 to 52·3)†
Mauritania	9·0 (6·3 to 14·1)	967·2 (675·3 to 1505·5)	45·4% (27·6 to 63·9)**	1·2% (0·0 to 7·4)§	10·2% (0·9 to 32·3)§	43·3% (26·6 to 59·4)**	35·9% (30·3 to 44·1)‡	27·6% (19·3 to 35·3)*
Mauritius	13·5 (8·9 to 20·6)	8996·3 (5915·2 to 13701·1)	89·7% (83·5 to 93·8)¶	0·0% (0·0 to 0·0)†	2·6% (1·0 to 6·3)†	7·7% (4·8 to 11·2)**	38·2% (27·0 to 49·7)†	43·8% (32·1 to 55·9)†
Mexico	965·0 (750·3 to 1228·9)	5266·5 (4094·7 to 6706·7)	83·2% (72·5 to 91·5)**	1·9% (0·5 to 4·4)¶	14·4% (7·5 to 22·6) ¶	0·5% (0·4 to 0·7)**	51·9% (40·5 to 70·3)**	15·6% (10·9 to 22·2)**
Moldova	12·0 (8·9 to 16·3)	2296·2 (1691·0 to 3116·6)	69·0% (59·2 to 78·1)**	0·2% (0·0 to 1·0)§	1·7% (0·5 to 4·4)§	29·1% (20·8 to 38·4)**	31·8% (20·4 to 43·4)‖	34·6% (25·2 to 44·9)†
Mongolia	18·5 (16·8 to 21·4)	116 549·7 (105 647·4 to 134 353·7)	23·9% (16·9 to 33·5)**	0·1% (0·0 to 0·4)¶	3·2% (0·5 to 10·6)§	72·7% (62·8 to 79·9)**	15·8% (11·7 to 22·4)‡	51·0% (40·7 to 58·3)*
Montenegro	3·5 (2·2 to 5·5)	137 124·4 (85 645·0 to 216 721·2)	98·9% (97·8 to 99·5)‡	0·0% (0·0 to 0·1)†	1·0% (0·5 to 2·1)†	0·0% (0·0 to 0·0)**	32·4% (13·1 to 55·5)*	44·3% (19·9 to 67·0)*
Morocco	27·0 (22·6 to 33·0)	2457·0 (2049·5 to 2998·8)	75·1% (68·0 to 80·8)**	0·5% (0·1 to 2·2)§	7·5% (4·1 to 12·1)§	16·9% (13·7 to 20·0)**	37·7% (29·0 to 47·1)§	42·4% (32·0 to 52·1)§
Mozambique	861·2 (842·2 to 890·8)	507·6 (496·4 to 525·1)	5·7% (3·7 to 8·8)**	0·2% (0·0 to 0·9)**	0·2% (0·0 to 0·5)¶	94·0% (90·8 to 96·1)**	54·6% (53·9 to 55·6)§	22·5% (21·4 to 23·9)§
Myanmar	162·2 (151·7 to 180·0)	664·4 (621·2 to 737·4)	11·5% (5·7 to 19·7)**	0·1% (0·0 to 0·1)†	4·1% (2·8 to 5·8)†	84·4% (75·9 to 90·1)**	26·4% (23·7 to 30·7)*	29·4% (27·2 to 31·6)*
Namibia	397·8 (279·0 to 585·3)	1658·6 (1163·4 to 2440·4)	58·6% (43·0 to 72·8)**	2·6% (0·1 to 11·3)**	0·6% (0·2 to 1·2)‖	38·3% (25·1 to 52·6)**	58·3% (43·1 to 73·4)¶	21·1% (14·5 to 30·6)§
Nepal	95·6 (91·6 to 101·0)	3335·8 (3197·3 to 3526·4)	3·3% (1·9 to 5·3)‖	6·1% (3·5 to 9·6)‡	4·2% (3·4 to 4·9)‡	86·4% (81·7 to 90·1)**	26·3% (24·8 to 28·7)§	31·1% (27·5 to 34·1)†
Netherlands	370·6 (296·8 to 507·1)	14 067·4 (11 267·4 to 19 249·5)	94·7% (89·1 to 97·8)*	0·7% (0·2 to 1·8)*	4·6% (2·0 to 9·1)*	0·0% (0·0 to 0·0)**	62·2% (48·9 to 74·8)*	15·8% (7·9 to 26·7)*
New Zealand	55·4 (44·4 to 72·3)	11 331·2 (9083·9 to 14 771·6)	95·8% (92·0 to 98·1)*	0·6% (0·2 to 1·4)*	3·6% (1·7 to 6·6)*	0·0% (0·0 to 0·0)**	58·2% (44·7 to 71·3)*	20·0% (10·4 to 32·4)*
Nicaragua	75·3 (64·7 to 87·6)	12 893·5 (11 064·1 to 14 997·7)	57·0% (49·9 to 63·5)**	1·2% (0·2 to 3·3)§	3·9% (1·5 to 7·5)§	37·8% (32·3 to 43·8)**	32·4% (26·6 to 41·2)†	35·8% (27·4 to 44·8)‡
Niger	39·3 (36·8 to 43·1)	773·5 (723·8 to 848·9)	12·0% (6·2 to 19·5)**	0·2% (0·0 to 1·0)**	0·8% (0·1 to 2·9)**	87·0% (79·1 to 92·8)**	20·6% (18·1 to 24·3)†	29·4% (28·1 to 30·5)†
Nigeria	1082·6 (991·3 to 1236·8)	329·2 (301·4 to 376·1)	19·2% (12·3 to 29·2)**	0·1% (0·0 to 0·9)**	1·1% (0·1 to 3·9)§	79·6% (69·4 to 86·6)**	48·7% (46·2 to 54·0)¶	19·5% (16·8 to 22·8)¶
North Korea	8·9 (7·6 to 10·7)	734·1 (628·7 to 882·5)	97·0% (90·7 to 99·4)*	0·8% (0·1 to 2·9)*	2·2% (0·5 to 6·4)*	0·0% (0·0 to 0·0)**	33·6% (18·1 to 52·0)*	32·3% (16·9 to 50·0)*
Norway	110·9 (87·2 to 149·7)	19 229·4 (15 121·1 to 25 970·6)	96·7% (93·3 to 98·6)*	0·0% (0·0 to 0·0)*	3·3% (1·4 to 6·6)*	0·0% (0·0 to 0·0)**	64·5% (51·3 to 76·7)*	15·9% (7·9 to 26·7)*
Oman	13·4 (9·2 to 19·3)	8844·0 (6073·6 to 12 774·1)	98·7% (95·6 to 99·8)§	0·4% (0·0 to 2·3)*	0·8% (0·2 to 2·2)*	0·0% (0·0 to 0·0)**	30·7% (15·9 to 48·1)†	9·2% (2·9 to 19·1)†
Pakistan	118·5 (108·0 to 135·0)	2619·8 (2388·0 to 2983·7)	36·3% (30·5 to 44·2)**	0·1% (0·0 to 0·2)†	0·4% (0·1 to 1·2)†	63·2% (55·3 to 69·1)**	19·6% (16·6 to 24·7)‡	45·5% (37·7 to 52·8)‡
Palestine	2·1 (1·7 to 2·5)	8042·5 (6680·6 to 9794·0)	67·9% (55·7 to 75·3)†	5·7% (0·5 to 18·0)*	4·8% (2·0 to 7·4)*	21·6% (17·6 to 25·7)**	27·2% (16·2 to 39·7)*	45·0% (31·7 to 57·3)*
Panama	70·4 (50·8 to 94·9)	4214·8 (3043·3 to 5681·2)	71·9% (54·2 to 84·3)**	8·5% (2·2 to 20·2)‖	12·4% (6·0 to 19·8)**	7·2% (5·2 to 9·7)**	51·6% (38·4 to 63·7)‡	30·1% (19·7 to 41·1)‡
Papua New Guinea	76·9 (67·7 to 90·0)	2893·2 (2548·8 to 3385·0)	30·3% (22·1 to 40·8)**	0·0% (0·0 to 0·0)§	3·6% (0·6 to 10·5)§	66·1% (56·2 to 74·6)**	20·9% (17·5 to 25·3)‡	49·0% (42·6 to 54·4)*
Paraguay	25·7 (18·8 to 35·6)	3931·2 (2878·2 to 5448·0)	51·1% (35·7 to 65·9)**	5·2% (0·9 to 14·2)§	15·1% (6·2 to 25·8)§	28·6% (20·1 to 38·0)**	46·7% (32·4 to 59·9)*	31·3% (22·8 to 41·3)*
Peru	101·3 (60·6 to 165·7)	3403·7 (2035·1 to 5567·4)	83·5% (66·3 to 93·6)**	1·5% (0·3 to 4·0)**	12·9% (4·4 to 27·5)**	2·1% (1·2 to 3·4)**	51·5% (36·2 to 70·0)¶	23·6% (7·6 to 40·4)**
Philippines	34·3 (28·3 to 41·9)	221·3 (182·7 to 270·6)	51·4% (41·8 to 61·0)**	1·0% (0·2 to 2·9)‖	6·2% (2·0 to 13·4)¶	41·5% (33·6 to 49·7)**	23·6% (18·6 to 30·0)**	47·8% (38·1 to 57·1)‖
Poland	208·6 (135·7 to 309·2)	19 960·0 (12 985·0 to 29 588·8)	98·9% (97·4 to 99·6)¶	0·2% (0·0 to 0·6)†	1·0% (0·4 to 2·0)†	0·0% (0·0 to 0·0)**	43·8% (20·8 to 68·1)*	30·9% (10·9 to 55·9)*
Portugal	332·6 (232·1 to 459·2)	1425·6 (995·0 to 1968·4)	80·4% (65·8 to 91·5)§	1·0% (0·3 to 2·3)*	18·6% (8·2 to 32·0)*	0·0% (0·0 to 0·0)**	64·4% (52·4 to 75·4)*	10·7% (4·9 to 18·4)*
Qatar	6·4 (3·6 to 11·2)	108 976·6 (61 735·9 to 192 075·2)	99·7% (99·1 to 99·9)*	0·1% (0·0 to 0·5)*	0·2% (0·1 to 0·4)*	0·0% (0·0 to 0·0)**	58·4% (35·6 to 77·4)*	27·1% (11·4 to 48·7)*
Romania	158·2 (101·1 to 252·1)	21 473·4 (13 717·6 to 34 207·1)	99·8% (99·4 to 99·9)‖	0·0% (0·0 to 0·0)†	0·2% (0·1 to 0·5)†	0·0% (0·0 to 0·0)**	43·1% (20·1 to 66·3)*	30·8% (11·2 to 55·1)*
Russian Federation	636·5 (280·9 to 1320·8)	1641·6 (724·5 to 3406·4)	96·9% (92·9 to 98·8)¶	0·1% (0·0 to 0·5)†	0·7% (0·1 to 2·8)†	2·3% (1·0 to 4·5)**	52·4% (32·1 to 72·0)*	22·0% (6·9 to 43·3)*
Rwanda	419·5 (389·7 to 466·3)	2125·0 (1974·0 to 2362·6)	16·8% (10·7 to 25·1)**	0·3% (0·0 to 0·8)†	0·5% (0·3 to 0·7)¶	82·4% (74·0 to 88·6)**	36·9% (34·2 to 41·4)§	30·0% (26·6 to 34·6)†
Saint Lucia	1·2 (1·0 to 1·4)	9907·4 (8353·1 to 12 088·9)	41·9% (32·2 to 52·3)†	0·7% (0·1 to 2·6)*	5·3% (1·4 to 13·1)*	52·1% (42·4 to 61·3)**	36·3% (27·8 to 46·0)*	32·9% (24·8 to 42·4)*
Saint Vincent and the Grenadines	1·7 (1·2 to 2·4)	6331·3 (4678·9 to 9151·9)	55·0% (40·7 to 69·7)§	0·3% (0·1 to 0·9)†	2·4% (1·3 to 3·9)†	42·3% (28·4 to 55·6)**	20·1% (17·4 to 25·9)†	16·5% (11·8 to 21·1)†
Samoa	0·6 (0·4 to 0·8)	5488·5 (3665·4 to 8280·8)	67·4% (43·0 to 82·8)§	7·5% (1·0 to 20·7)†	11·8% (2·5 to 26·2)†	13·3% (8·4 to 19·0)**	14·0% (9·6 to 21·5)‡	14·6% (5·5 to 30·1)†
São Tomé and Príncipe	0·9 (0·8 to 1·1)	1533·5 (1398·7 to 1773·0)	18·6% (11·7 to 28·3)**	0·9% (0·0 to 6·2)†	1·5% (0·2 to 4·6)†	79·0% (68·1 to 86·3)**	31·8% (28·2 to 37·5)*	35·9% (31·5 to 39·5)*
Saudi Arabia	157·4 (96·9 to 261·4)	35 953·1 (22 141·1 to 59 707·1)	98·5% (94·9 to 99·7)†	0·5% (0·0 to 2·8)*	0·9% (0·3 to 2·3)*	0·0% (0·0 to 0·0)**	62·2% (42·9 to 79·1)†	27·0% (12·7 to 47·7)†
Senegal	61·7 (52·5 to 77·7)	1199·1 (1021·8 to 1511·3)	14·7% (5·6 to 28·0)‖	4·0% (0·2 to 14·8)†	5·7% (2·0 to 9·6)†	75·5% (59·4 to 87·8)**	32·8% (28·7 to 40·1)*	34·0% (28·8 to 38·2)*
Serbia	24·2 (15·8 to 35·9)	26 408·9 (17 202·9 to 39 197·6)	99·3% (98·0 to 99·8)‡	0·0% (0·0 to 0·1)†	0·6% (0·1 to 1·8)†	0·2% (0·1 to 0·2)**	36·2% (15·9 to 59·4)*	39·9% (17·2 to 62·5)*
Seychelles	3·8 (1·8 to 6·8)	29 503·4 (14 220·2 to 53 593·9)	99·9% (99·7 to 100·0)‖	0·0% (0·0 to 0·0)†	0·1% (0·0 to 0·2)†	0·1% (0·0 to 0·1)**	58·1% (43·0 to 74·4)†	8·0% (3·5 to 16·4)‡
Sierra Leone	19·9 (19·0 to 21·4)	379·5 (362·1 to 406·7)	10·9% (6·8 to 16·9)**	0·5% (0·0 to 1·8)§	0·8% (0·2 to 1·6)†	87·9% (81·9 to 92·0)**	26·4% (24·3 to 29·5)*	44·7% (42·2 to 46·7)‡
Singapore	76·5 (60·9 to 96·5)	6389·5 (50 91·4 to 8062·2)	92·7% (86·9 to 96·3)¶	0·5% (0·2 to 1·2)*	6·8% (3·5 to 11·8)*	0·0% (0·0 to 0·0)**	68·1% (57·3 to 77·7)§	23·1% (13·7 to 34·4)§
Slovakia	23·5 (14·2 to 38·2)	112 793·2 (68 146·7 to 183 377·4)	99·5% (98·5 to 99·9)*	0·1% (0·0 to 0·2)*	0·5% (0·1 to 1·3)*	0·0% (0·0 to 0·0)**	37·5% (16·0 to 61·9)*	40·4% (16·4 to 65·6)*
Slovenia	13·3 (7·9 to 21·6)	100 865·3 (60 066·3 to 164 007·8)	99·1% (97·4 to 99·8)*	0·4% (0·1 to 1·6)*	0·4% (0·1 to 1·0)*	0·0% (0·0 to 0·0)**	40·6% (18·6 to 65·6)*	37·3% (14·1 to 61·0)*
Solomon Islands	0·5 (0·4 to 0·6)	1619·4 (1312·0 to 2160·5)	75·2% (55·0 to 84·8)‡	6·4% (0·8 to 19·8)*	5·8% (1·0 to 15·8)*	12·6% (9·3 to 15·3)**	19·2% (8·8 to 34·5)†	49·4% (33·0 to 65·2)*
Somalia	28·4 (27·8 to 30·1)	823·0 (804·3 to 872·2)	0·8% (0·5 to 1·2)†	0·5% (0·0 to 2·5)*	2·1% (0·5 to 5·8)*	96·5% (91·0 to 98·7)**	26·2% (25·4 to 27·2)*	40·3% (39·4 to 41·1)*
South Africa	4243·7 (2853·7 to 6666·9)	679·0 (456·6 to 1066·7)	69·3% (55·4 to 81·4)**	3·4% (0·4 to 11·4)§	0·6% (0·4 to 0·9)§	26·7% (16·3 to 38·0)**	67·0% (62·4 to 71·8)‡	13·8% (10·1 to 17·9)†
South Korea	230·4 (169·0 to 293·5)	6207·1 (4551·6 to 7906·8)	52·0% (37·9 to 65·5)‡	1·7% (0·8 to 3·0)*	46·3% (33·6 to 59·2)*	0·0% (0·0 to 0·0)**	37·7% (24·7 to 52·1)‡	26·0% (14·7 to 39·4)‡
South Sudan	19·2 (16·7 to 26·3)	122·1 (106·5 to 167·3)	3·3% (2·0 to 5·0)‡	1·1% (0·0 to 5·6)*	12·5% (2·5 to 32·5)*	83·1% (59·7 to 93·8)**	36·3% (31·9 to 42·3)*	27·9% (23·2 to 31·8)*
Spain	1382·0 (1059·6 to 1888·1)	4195·4 (3216·5 to 5731·5)	94·4% (90·0 to 97·4)¶	0·2% (0·1 to 0·6)*	5·4% (2·5 to 9·4)*	0·0% (0·0 to 0·0)**	63·4% (50·1 to 74·9)*	13·2% (6·1 to 22·8)*
Sri Lanka	15·2 (12·8 to 18·4)	8526·2 (7179·2 to 10 285·7)	57·7% (50·2 to 65·6)**	0·1% (0·0 to 0·3)‡	3·9% (1·9 to 7·0)‡	38·3% (31·4 to 45·0)**	16·9% (12·7 to 23·6)‡	45·8% (33·1 to 59·4)*
Sudan	24·5 (20·3 to 31·0)	570·0 (472·6 to 723·2)	29·9% (17·0 to 45·7)**	1·1% (0·2 to 3·5)‡	9·7% (7·1 to 12·2)‡	59·3% (46·2 to 70·6)**	30·8% (24·1 to 40·0)*	37·0% (29·9 to 44·0)*
Suriname	8·0 (6·7 to 9·8)	2526·6 (2105·3 to 3102·1)	52·7% (43·6 to 62·3)¶	3·5% (0·7 to 10·3)‡	1·3% (0·6 to 2·2)‡	42·5% (34·3 to 50·5)**	53·5% (47·9 to 59·8)†	28·0% (20·7 to 34·6)†
Swaziland	247·0 (195·2 to 321·4)	1063·4 (840·5 to 1383·6)	43·0% (29·2 to 56·8)**	0·9% (0·1 to 3·8)¶	1·8% (0·3 to 6·2)§	54·3% (41·1 to 67·6)**	45·3% (39·6 to 50·4)‖	14·6% (11·9 to 18·4)§
Sweden	156·4 (122·0 to 206·2)	17 261·2 (13 462·9 to 22 767·3)	95·7% (91·1 to 98·3)*	0·1% (0·0 to 0·2)*	4·2% (1·7 to 8·7)*	0·0% (0·0 to 0·0)**	60·4% (46·8 to 73·2)*	19·2% (9·5 to 31·3)*
Switzerland	186·2 (134·1 to 238·0)	8017·8 (5774·7 to 10 248·0)	32·9% (19·8 to 46·9)¶	2·6% (1·4 to 4·0)*	64·5% (51·5 to 76·6)*	0·0% (0·0 to 0·0)**	66·7% (54·1 to 77·9)*	14·9% (7·3 to 26·6)*
Syria	14·6 (10·6 to 19·9)	9613·0 (6999·7 to 13 120·7)	89·9% (86·6 to 92·9)§	0·5% (0·1 to 1·6)†	1·9% (1·2 to 2·7)†	7·7% (5·5 to 10·3)**	34·0% (19·2 to 50·3)*	40·0% (23·4 to 56·2)*
Taiwan (Province of China)	121·0 (98·6 to 153·2)	22 289·5 (18 154·6 to 28 216·7)	99·7% (99·1 to 100·0)*	0·1% (0·0 to 0·2)*	0·2% (0·0 to 0·7)*	0·0% (0·0 to 0·0)**	58·5% (34·7 to 80·6)*	17·5% (5·9 to 34·7)*
Tajikistan	36·2 (32·9 to 40·8)	11 280·9 (10 248·4 to 12 723·4)	29·3% (22·7 to 37·5)**	0·0% (0·0 to 0·1)§	2·1% (0·6 to 4·9)‖	68·6% (60·6 to 75·3)**	29·1% (26·5 to 32·9)§	36·7% (33·3 to 40·0)¶
Tanzania	1411·4 (1364·9 to 1480·9)	889·0 (859·7 to 932·7)	9·2% (6·6 to 12·1)**	0·2% (0·0 to 1·2)‖	1·0% (0·1 to 3·7)‖	89·6% (85·4 to 92·6)**	49·3% (47·4 to 51·7)‡	32·3% (29·1 to 35·4)‡
Thailand	866·1 (686·4 to 1073·5)	1973·7 (1564·3 to 2446·3)	92·1% (90·2 to 93·8)**	0·1% (0·0 to 0·2)§	0·2% (0·0 to 0·5)§	7·7% (6·1 to 9·6)**	51·9% (45·7 to 62·7)**	6·0% (3·0 to 10·9)**
The Bahamas	8·5 (5·9 to 11·9)	26 80·7 (1863·9 to 3722·6)	99·0% (96·6 to 99·8)‡	0·4% (0·0 to 1·8)†	0·6% (0·1 to 1·6)†	0·0% (0·0 to 0·0)**	55·9% (37·9 to 72·3)*	15·1% (4·8 to 31·6)*
The Gambia	19·4 (18·7 to 20·5)	939·7 (905·2 to 991·4)	14·5% (11·3 to 19·0)¶	0·1% (0·0 to 0·4)†	0·1% (0·0 to 0·3)†	85·4% (80·9 to 88·6)**	29·5% (27·1 to 32·5)*	32·8% (30·5 to 35·2)*
Timor-Leste	5·3 (4·6 to 6·1)	2847·2 (2492·5 to 3314·9)	34·3% (25·4 to 43·9)§	0·0% (0·0 to 0·1)†	0·1% (0·0 to 0·3)†	65·6% (56·0 to 74·5)**	29·7% (24·3 to 37·0)*	31·8% (27·2 to 37·3)*
Togo	43·7 (34·3 to 63·7)	384·6 (301·8 to 560·8)	26·0% (14·0 to 39·6)**	9·0% (1·0 to 28·8)**	6·1% (1·5 to 13·5)**	58·9% (39·4 to 73·3)**	34·8% (26·6 to 45·0)¶	31·3% (24·0 to 39·6)§
Tonga	0·2 (0·2 to 0·3)	3755·0 (2731·6 to 5726·6)	35·2% (21·7 to 46·9)§	23·8% (7·6 to 44·5) ‡	7·1% (2·9 to 11·0)‡	33·9% (21·4 to 45·0)**	38·8% (28·9 to 50·0)*	21·3% (10·4 to 33·4)†
Trinidad and Tobago	33·0 (23·2 to 44·9)	4695·1 (3302·8 to 6388·0)	97·3% (93·9 to 98·9)‡	0·3% (0·1 to 1·1)†	2·4% (1·0 to 5·2)†	0·0% (0·0 to 0·0)**	58·1% (40·0 to 74·6)*	17·1% (6·0 to 34·0)*
Tunisia	20·2 (14·4 to 26·8)	9931·6 (7109·9 to 13 178·9)	61·9% (46·9 to 75·4)§	2·5% (0·3 to 8·7)†	26·0% (16·0 to 36·4)†	9·7% (7·1 to 13·2)**	44·7% (35·7 to 55·3)§	36·4% (25·1 to 46·3)§
Turkey	292·7 (211·4 to 399·0)	40 332·0 (29 124·7 to 54 968·0)	98·5% (95·6 to 99·6)‡	0·3% (0·0 to 1·7)†	1·2% (0·3 to 2·8)†	0·0% (0·0 to 0·0)**	46·1% (26·6 to 66·4)*	32·6% (16·2 to 53·1)*
Turkmenistan	29·0 (18·7 to 45·5)	8056·0 (5183·6 to 12 652·4)	81·5% (54·7 to 93·5)*	0·5% (0·0 to 1·9)*	13·4% (1·8 to 39·4)*	4·7% (2·8 to 6·9)**	45·3% (19·5 to 72·7)*	33·0% (4·9 to 67·8)*
Uganda	1454·6 (1332·9 to 1609·9)	945·6 (866·5 to 1046·6)	12·3% (6·3 to 20·2)**	1·2% (0·2 to 3·8)†	9·7% (6·2 to 13·2)‡	76·8% (69·3 to 83·6)**	52·9% (49·5 to 57·5)‡	22·1% (18·7 to 26·8)*
Ukraine	615·4 (419·8 to 1192·9)	4353·5 (2969·8 to 8438·5)	38·3% (17·5 to 70·1)**	2·1% (0·1 to 13·5)‖	2·5% (0·2 to 11·9)‖	57·0% (27·2 to 77·2)**	40·9% (36·2 to 46·8)†	20·6% (17·2 to 27·1)†
United Arab Emirates	47·8 (38·7 to 58·8)	30 606·9 (24 805·8 to 37 676·4)	99·1% (97·3 to 99·8)‡	0·2% (0·0 to 0·9)*	0·7% (0·2 to 1·8)*	0·0% (0·0 to 0·0)**	58·9% (38·0 to 77·7)*	22·9% (9·1 to 42·3)*
UK	999·0 (787·6 to 1308·5)	10 340·0 (8151·7 to 13 543·9)	93·8% (87·9 to 97·4)‡	0·4% (0·1 to 1·0)*	5·8% (2·4 to 11·0)*	0·0% (0·0 to 0·0)**	60·8% (47·5 to 73·1)*	17·9% (8·9 to 30·4)*
USA	5174·0 (3954·7 to 6539·9)	2969·3 (2269·5 to 3753·2)	76·2% (66·6 to 84·5)**	10·1% (5·6 to 16·2)**	13·7% (9·9 to 17·8)**	0·0% (0·0 to 0·0)**	67·3% (54·1 to 78·4)*	11·9% (5·7 to 21·7)*
Uruguay	50·0 (37·4 to 68·4)	4468·4 (3348·1 to 6122·2)	92·1% (81·6 to 97·7)§	0·6% (0·1 to 1·9)‡	7·2% (2·2 to 16·7)‡	0·0% (0·0 to 0·0)**	35·2% (29·4 to 41·5)§	32·0% (23·8 to 39·5)§
Uzbekistan	59·7 (45·4 to 80·6)	6712·5 (5103·5 to 9066·9)	63·0% (51·0 to 73·4)**	0·4% (0·0 to 1·4)*	5·2% (0·8 to 16·2)*	31·4% (22·8 to 40·5)**	32·0% (17·6 to 51·8)*	37·7% (15·0 to 57·0)*
Vanuatu	3·0 (2·9 to 3·1)	21 049·1 (20 437·3 to 22 005·6)	5·2% (3·3 to 7·7)§	1·3% (0·1 to 4·5)*	0·3% (0·0 to 0·9)*	93·3% (89·2 to 96·0)**	14·1% (13·2 to 15·7)†	37·0% (35·6 to 38·2)*
Venezuela	193·5 (146·6 to 252·5)	2504·8 (1898·1 to 3269·8)	80·8% (62·9 to 92·6)‖	1·6% (0·3 to 4·4)*	17·6% (7·1 to 32·8)*	0·0% (0·0 to 0·1)**	93·3% (80·4 to 97·5)‖	4·6% (1·9 to 8·8)‖
Vietnam	287·3 (251·6 to 333·8)	1101·6 (964·8 to 1280·0)	22·3% (13·5 to 32·7)**	0·7% (0·3 to 1·4)†	18·0% (12·6 to 24·1)‡	59·0% (50·5 to 67·0)**	45·7% (41·5 to 50·6)‡	23·5% (19·8 to 27·5)‡
Yemen	7·6 (5·2 to 11·1)	1829·8 (1254·8 to 2681·3)	40·7% (25·5 to 56·6)‡	1·4% (0·1 to 4·7)*	36·9% (15·1 to 53·8)*	21·0% (13·8 to 29·5)**	30·2% (18·1 to 44·4)*	39·7% (26·3 to 52·4)*
Zambia	800·2 (746·0 to 896·4)	641·3 (597·9 to 718·4)	11·7% (5·7 to 21·1)**	0·1% (0·0 to 0·2)§	1·6% (0·8 to 3·1)‡	86·6% (77·2 to 92·7)**	57·3% (54·8 to 60·9)*	18·6% (16·7 to 21·5)†
Zimbabwe	668·0 (623·4 to 773·4)	470·0 (438·6 to 544·2)	8·6% (5·7 to 11·7)**	3·6% (0·1 to 14·2)‡	1·5% (0·3 to 3·6)‡	86·3% (74·3 to 92·2)**	35·3% (33·1 to 38·3)*	33·0% (30·3 to 35·1)*

Spending reported in 2017 purchasing-power parity-adjusted dollars. Income groups are 2017 World Bank income groups. Data for number of prevalent cases are sourced from the Global Burden of Disease 2016 Study.^21^ 95% uncertainty intervals are shown in parentheses. We added the count of private HIV/AIDS spending data points to the count of out-of-pocket and prepaid private data points. For categories presented as a proportion of total spending, footnotes refer to number of underlying data points for the numerator only.

* No datapoints.

† 1–2 datapoints.

‡ 3–4 datapoints.

§ 5–6 datapoints.

¶ 7–8 datapoints.

‖ 9–10 datapoints.

** More than 10 datapoints.

Figure 4B shows that, globally, governments were the largest source of spending on HIV/AIDS, contributing a total of $29·8 billion (95% UI 27·5 billion to 32·8 billion) or 61·0% (55·1 to 65·1) of total HIV/AIDS spending in 2015 (figure 4B). Prepaid private spending was the smallest, making up only $1·4 billion (0·4 billion to 3·8 billion) or 2·8% (0·9 to 6·9) of the total in 2015. The development assistance for health share of HIV/AIDS spending is larger than is the development assistance for health portion of total health spending: whereas development assistance for health made up $51·8 billion or 0·5% (0·5 to 0·5) of total health spending globally in 2015, development assistance for health comprised $14·5 billion or 29·7% (26·7 to 32·0) of all HIV/AIDS spending in 2015.

Figure 4C highlights the evolution in the focus of HIV/AIDS resources over time. Spending on care and treatment of HIV/AIDS has grown substantially. In 2000, $8·1 billion (95% UI 6·9 billion to 9·8 billion) in HIV/AIDS resources was expended on care and treatment services, including inpatient and outpatient care and antiretroviral therapy delivered in these settings. By 2015, $27·3 billion (24·5 billion to 31·1 billion) of all HIV/AIDS spending, or 55·8% (53·3 to 57·9), was disbursed for these same services. Spending on prevention—including general public health programmes and projects focused on slowing transmission in at-risk groups—amounted to $4·0 billion (3·6 billion to 4·6 billion) in 2000. In 2015, spending on prevention reached $9·3 billion (8·5 billion to 10·4 billion) or 19·0% (17·6 to 20·6) of all HIV/AIDS spending.

Figure 4D shows that, in 2015, countries with a low (<1%) prevalence of HIV/AIDS, which collectively had 11·4 million people living with HIV/AIDS, spent the most on HIV/AIDS ($31·9 billion [95% UI 29·2 billion to 35·7 billion]), and had the highest spending per prevalent case ($2788 [2556 to 3118]). In 2015, HIV/AIDS spending in high (1–5%) prevalence countries was $8·8 billion (7·6 billion to 9·1 billion) and in extremely high (>5%) prevalence countries it was $8·8 (7·4 billion to 11·3 billion). HIV/AIDS spending per prevalent case in 2015 was generally lower in these countries, constituting $731 (682 to 814) per prevalent case in high prevalence countries, and $681 (570 to 869) per prevalent case in extremely high prevalence countries, the lowest across prevalence groups. Although spending per prevalent case in extremely high prevalence countries increased between 2010 and 2015 (1·2% [0·5 to 2·1] annually), this recent growth was much slower than the annual per prevalent case growth seen between 2000 and 2010 (10·2% [7·5 to 12·7]).

**Figure 5:**
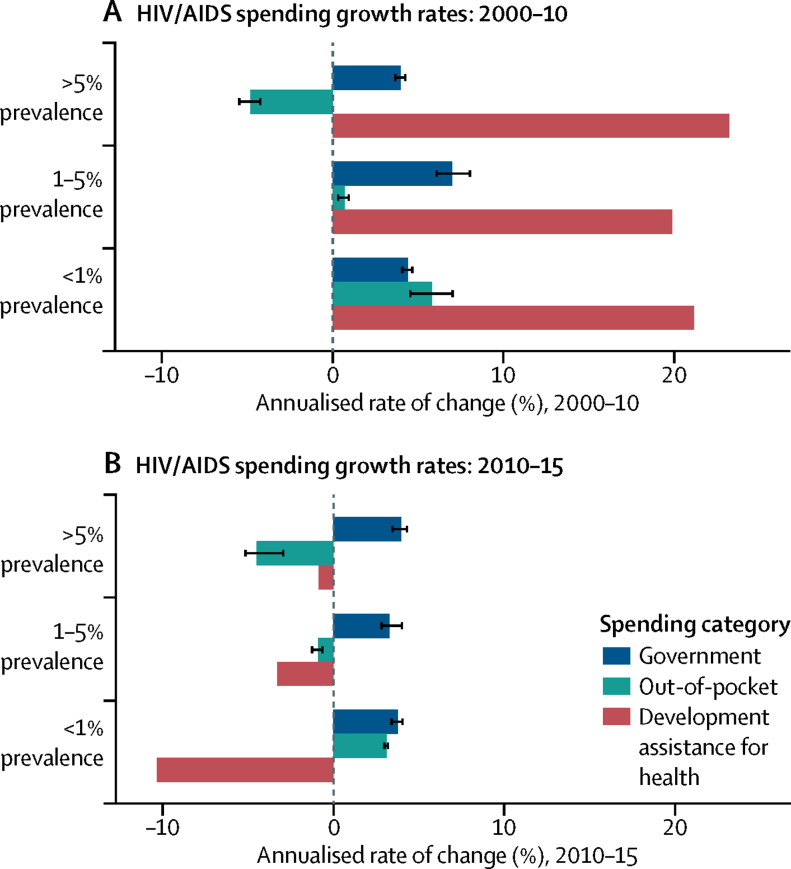
Annualised rate of change of HIV/AIDS spending per prevalent case, 2000–10 (A), and 2010–15 (B), by source and prevalence group Annualised rate of change of government and out-of-pocket spending on HIV/AIDS, and development assistance for HIV/AIDS. Number of prevalent cases date to 2015 and are sourced from the Global Burden of Disease Study 2016.^21^ Error bars represent 95% uncertainty intervals.

Figure 5 highlights the financing sources that have contributed to HIV/AIDS spending per prevalent case growth. There have been major increases in development assistance per prevalent case of HIV/AIDS—annualised rates of change for all prevalence groups increased by 19·9% from 2000 to 2010 (figure 5). However, between 2010 and 2015, annual declines in development assistance for HIV/AIDS per prevalent case were observed in all HIV/AIDS prevalence groups (figure 5). Growth in government spending per prevalent case was also substantial in the 10 years after the millennium, increasing more than 4·0% annually for all country groupings (figure 5). Alongside the decreases in development assistance for health for HIV/AIDS between 2010 and 2015, the increases in government spending on HIV/AIDS were largely sustained. Finally, trends in out-of-pocket spending per prevalent case are mixed across prevalence groups and time periods. In countries with extremely high prevalence, out-of-pocket spending per prevalent case decreased by 4·8% (95% UI 4·2–5·5) annually between 2000 and 2010, and by 4·5% (2·9–5·2) annually between 2010 and 2015. By contrast, out-of-pocket spending per prevalent case increased in both periods among low-prevalence countries (figure 5).

Table 2 reports total health spending for HIV/AIDS for each person living with HIV/AIDS in 2015. Some of the countries with the highest HIV/AIDS spending per prevalent case, such as Bosnia and Herzegovina and Macedonia, have few people living with HIV/AIDS (estimated to be fewer than 80 people in 2015, in both countries). Additionally, there was low HIV/AIDS spending per prevalent case in some high-burden countries, such as Haiti and Rwanda (table 2). These patterns exist generally across each prevalence group. In extremely high prevalence countries in 2015, 58·1% (95% UI 54·8–62·1) of HIV/AIDS spending was disbursed as care and treatment, whereas 17·9% (15·2–20·0) was spent on prevention. This finding contrasts with low-burden countries, where 18·5% (16·6–20·9) of all HIV/AIDS spending in 2015 was used to prevent the transmission of HIV. This distinction remains even when comparing prevention spending per capita: low-prevalence countries spent $516 (456–588) per person, whereas high prevalence countries spent just $121 (105–153), 76·4% (69·7–80·5) less than in areas where HIV prevalence is low.

**Figure 6:**
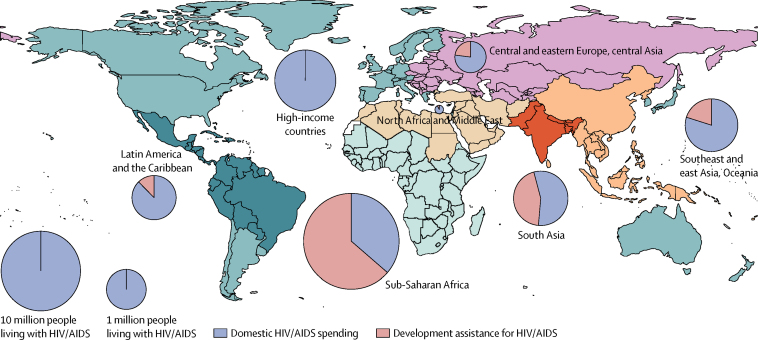
Domestic HIV/AIDS spending, development assistance for HIV/AIDS, and number of people living with HIV/AIDS, 2015 The size of each pie represents the number of people living with HIV/AIDS in 2015, in each specified Global Burden of Disease super-region. Domestic spending includes out-of-pocket, government, and prepaid private spending. Number of prevalent cases date to 2015, and are sourced from the Global Burden of Disease Study 2016.^21^

Figure 6 depicts the share of HIV/AIDS spending sourced externally, as development assistance for health, for each GBD region in 2015. The size of each pie represents the number of people living with HIV/AIDS. Not only does sub-Saharan Africa have the largest HIV-positive population (24·4 million in 2015), it also depends most substantially on development assistance for health, which constitutes 63·9% (95% UI 55·7–70·2) of HIV/AIDS spending in the region. South Asia also has a high level of dependence on donor financing, with development assistance for health comprising 45·2% (33·7–55·4) of spending on HIV/AIDS. Development assistance for health makes up more than 20% of spending on HIV/AIDS in southeast Asia, east Asia, Oceania, central Europe, eastern Europe, and central Asia. In high-income countries, Latin America, and the Caribbean, development assistance for health constitutes less than 13% of HIV/AIDS spending.

For each dollar of development assistance for health for HIV/AIDS, in countries that receive it, there is $2·1 (95% UI 1·9–2·3) in government health spending for HIV/AIDS. In 2015, low-income, lower-middle-income, and upper-middle-income countries with low (<1%) HIV/AIDS prevalence received just $4·1 billion or 26·0% (22·8–28·5) of their HIV/AIDS spending as development assistance for health, whereas high prevalence (1–5%) countries received $6·0 billion or 76·5% (68·7–81·5) of total HIV/AIDS spending as development for health, and extremely high prevalence countries (>5%) received $4·4 billion or 50·5% (39·1–59·6) of total HIV/AIDS spending as development assistance for health. In 2015, Haiti received 97·1% (95·9–98·0) and Guinea-Bissau received 89·9% (83·0–94·2) of their HIV/AIDS spending as development assistance for health; both are categorised as high prevalence countries. Similarly, of the extremely high prevalence countries, Mozambique received 94·0% (90·8–96·1) and Zambia received 86·6% (77·2–92·7) of their HIV/AIDS spending as development assistance for health.

Among low-income countries with high or extremely high prevalence of HIV/AIDS, $5·3 billion or 85·4% (79·8–88·6) of HIV/AIDS spending was sourced externally. However, during the past 5 years, the share of HIV/AIDS spending that is development assistance has been decreasing in countries of high and extremely high prevalence.

**Figure 7:**
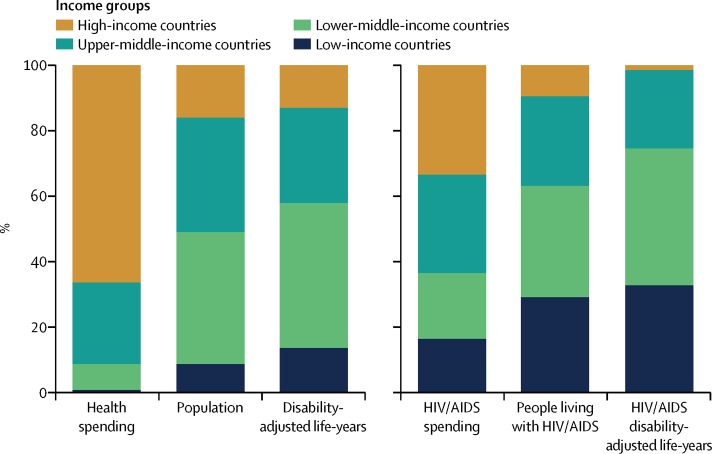
Spending, population size, and disability-adjusted life -years overall and for HIV/AIDS, by income group, 2015 Income groups are 2017 World Bank income groups.

Figure 7 depicts spending, population size, and disability-adjusted life-years overall and for HIV/AIDS, by income group in 2015. High-income countries account for 33·3% (95% UI 30·9–35·8) of total global HIV/AIDS spending but comprise 1·3% of the burden, as measured by disability-adjusted life-years, and 9·4% of the people living with HIV globally (figure 7). In low-income countries, where 32·8% of HIV/AIDS burden occurs and 29·2% of HIV-positive people live, spending on HIV/AIDS constitutes 16·4% (15·4–17·5) of global spending (figure 7). Compared with all-health spending, a higher proportion of HIV/AIDS spending occurs in low-income and middle-income countries (including lower-middle-income and upper-middle-income countries). High-income countries have 12·9% of the total health burden and spend 66·3% (66·0–66·5) of total health spending, whereas low-income countries constitute 0·7% (0·7–0·7) of total health spending and account for 13·7% of global health burden (figure 7).

The ratio of development assistance for health to government spending, in 2015, was 1·5 in low-income countries and 0·1 in lower-middle-income countries, although the ratio of development assistance for HIV/AIDS to government HIV/AIDS spending was 7·7 for low-income countries and 1·9 for lower-middle-income countries.

## Discussion

From 1995 to 2015, global per capita health spending increased substantially. Health spending is on the verge of surpassing $10 trillion and accounts for 10% of the world's total economy. The USA alone accounted for almost a third of the world's health spending in 2015—slightly less than what was spent by all low-income and middle-income countries combined. Between 1995 and 2015, per capita health spending grew the fastest in middle-income countries, despite the declines in development assistance for health that occurred from 2010 to 2015. More broadly, development assistance for health remained relatively flat since 2013, although disbursements to some health focus areas, such as HIV/AIDS, have declined.

In recognition of the growing threat of infectious disease outbreaks and epidemics in an increasingly interconnected world, we tracked development assistance for pandemic preparedness for the first time. We expect our estimates of development assistance targeted towards pandemic preparedness to improve over time as global initiatives that address these threats, such as the Global Health Security Agenda, become well established. According to the World Bank, the estimate of the economic and fiscal costs of the Ebola crises in the three affected countries—Guinea, Liberia, and Sierra Leone—was approximately $2·8 billion.^25^ This estimate signals that greater investment in pandemic preparedness could be warranted.

In 1995, 18 million people were living with HIV/AIDS globally and 3 million additional cases emerged each year. At its peak in 2005, nearly 2 million people died from HIV/AIDS each year. Since 2005, the number of new HIV/AIDS cases each year have decreased by 23%, the global HIV/AIDS death rate declined by 52%, and 20·9 million people are now on antiretroviral therapy. These historic gains were made partly because of the extraordinary amount of resources made available to fight HIV/AIDS. Between 2000 and 2015, $562·6 billion (531·1 billion to 621·9 billion) was spent to combat the disease, with annual growth in HIV/AIDS spending surpassing 10% for many years. International support played a key part in the escalation of funding.

Domestic governments also played an important role in the fight against HIV/AIDS, constituting the majority of worldwide spending on HIV/AIDS between 2000 and 2015. Government resources have continued to grow in all income groups while development assistance for health for HIV/AIDS declined. Governments played an integral part in the development and advancement of key prevention and treatment programmes. Still, in low-income countries and countries with extremely high prevalence, development assistance for health constituted most HIV/AIDS spending, even in 2015.

Unique to HIV/AIDS is the small share spent out-of-pocket. Domestic HIV/AIDS spending is drawn predominately from government financing rather than being out-of-pocket, the latter of which leaves people susceptible to financial instability and impoverishment. In 2015, out-of-pocket spending accounted for less than 10% of HIV spending, smaller than overall out-of-pocket spending, which comprised nearly a quarter of total health spending. Our analysis suggests international and domestic government efforts surrounding HIV/AIDS have a major role in mitigating the financial hardship associated with HIV/AIDS.

Despite the considerable domestic response to HIV/AIDS, many low-income and middle-income countries remain dependent on development assistance for health to fund HIV/AIDS programmes. Development assistance for health made up most of the total spending on HIV/AIDS in high prevalence (1–5%) countries in 2015. In extremely high prevalence (>5%) countries, development assistance for health comprises half of HIV/AIDS spending. Low-income countries make up half of high-prevalence countries and a third of extremely high-prevalence countries.^26^ In low-income and lower-middle-income countries, the ratio of development assistance for health to government spending, for HIV/AIDS, is nearly twenty times higher than the ratio of development assistance for health to government spending overall. Domestically sourced resources are crucial to the long-term sustainability of HIV/AIDS programmes, but governments in low-income countries often have constrained fiscal space, generally driven by low government revenue. Reliance on development assistance for health to fight HIV/AIDS in these countries leaves them susceptible to fluctuations in the external resources available for HIV/AIDS, and puts national HIV/AIDS programmes at risk of gaps in support and unrealised investment opportunities.

High-prevalence countries reliant on development assistance for health must plan strategically so that decreases in external financing do not alter trajectories towards ending the transmission of HIV and sustaining HIV-positive populations with antiretroviral therapy. Ageing HIV-positive populations will continue to need antiretroviral therapy to live healthy and productive lives. Potential ways to offset declines in external HIV/AIDS funding include reallocating more government resources to the health sector, reallocating more government health resources to HIV/AIDS, or reallocating government HIV/AIDS resources to focus on the most effective approaches to HIV/AIDS prevention or treatment.^27^, ^28^ Improving the efficiency of antiretroviral therapy service provision and integrating HIV/AIDS programmes into the health system are also potential strategies that complement the reductions in treatment costs, bolstered by heavily negotiated antiretroviral therapy prices, which have transpired in developing countries since 2000.^29^, ^30^ Although this set of options suggests there might be multiple means to preserve the gains made in curbing the HIV/AIDS epidemic, in many contexts these options might not be realistic. The government of an average low-income country spent less than $25 per person on health in 2015. Reallocating government resources to HIV/AIDS might not be possible in many cases or would be possible only by reducing spending on other health priorities.

Apart from the novel tracking of HIV/AIDS spending, our analysis provides further evidence of the wide variation in health spending, as well as the disconnect between health spending and health burden. Although these differences are stark, many factors affect how much is spent on health and which diseases are prioritised for spending in each country. Factors such as access to and price of health care, and efficiency of health systems, probably explain some variation in spending and prioritisation. Additionally, political and social preferences, and the availability of cost-effective interventions, govern how many resources are directed to the health sector, as well as to which patients and diseases. Although we would not expect spending levels or health system foci to be commensurate across income groups or to merely reflect health burden, we believe these discrepancies highlight potential gaps and places where more attention should be given to determine if health need is being met. We hope this exercise and future disease tracking spending studies could help parse out the factors associated with disease spending and identify how donors and governments can reduce financial barriers impeding progress towards important health-related goals.

This research takes an initial step towards global disease-specific resource tracking, which is essential for several reasons. Disease-specific spending estimates make a host of new, policy-relevant analyses possible, including decomposing the drivers of health spending growth,^31^ quantifying disease-specific spending gaps, and assessing the effects of health spending. These estimates enable researchers to assess how disease-specific funding complements or replaces other health spending. Existing evidence shows that development assistance for health provided to the government tends to replace domestic financing for health.^32^, ^33^ Finally, decision-makers can use estimates of this kind to inform the allocation of spending across diseases and other disease-specific policies. By combining disease-specific prevalence estimates, costing estimates, and spending estimates, more precise targets can be constructed and disease-specific spending gaps could be identified. This is important work that is only made possible by ongoing global disease-specific resource tracking.

### Limitations

Although increasingly granular tracking of health spending is advantageous for many reasons, it is not without challenges and limitations. The estimates for development assistance for health do not capture transfers of assistance among middle-income and low-income countries, largely because of the requirement that we capture a complete time series of disbursements for each agency we track, and that these data are comparable with all other data sources. Publicly available data that meet these requirements from low-income and middle-income countries are sparse, which is a gap we aim to fill in the future. Moreover, some of the input data used for parts of this study were not precise and required modelling. The input data for total health spending and HIV/AIDS spending were, in some cases, contradictory, had incomplete underlying documentation, and included many gaps. It is difficult in some cases for health accountants and financing experts to disentangle HIV/AIDS and tuberculosis funding, which could affect the underlying data used. A greater push and adherence to an agreed-upon set of spending definitions and methods to track resources would help produce more precise and comparable estimates of health spending. It is our hope that these estimates help demonstrate the use of disease-specific resource tracking studies, and can be a catalyst for more investment in global resource tracking for health. That approach includes necessary investment in low-income and middle-income countries, as well as in high-income countries, where the internationally consistent tracking for HIV/AIDS spending was weakest. The wide UIs surrounding our estimates should be a recurring reminder of the need for sustained investment in health systems capable of disease-specific resource tracking. In addition to tracking health spending with more rigour and precision, we urge that investments be made in tracking spending subnationally to assess, with more accuracy and consistency, within-country spending disparities across important socioeconomic and geographical stratifiers. Many country-specific studies have shown that within-country health spending varies as substantially as cross-country estimates, with equally as poignant conclusions.^21^ Measuring spending subnationally by disease would be valuable for assessing the connection between spending patterns and disease-specific health outcomes, including avertable mortality.

## Conclusion

Even as development assistance for health levels off, health spending continues to increase, outpacing economic growth in many contexts. With growth steady or accelerating, it is more important than ever to understand where resources for health go and how they align with health needs, particularly because major variation in spending persists across countries. Estimates of spending on HIV/AIDS are a step toward better understanding this variation. The noteworthy increases in spending on HIV/AIDS has mitigated, at least at present, a major global health crisis. Prevention efforts will remain essential in all contexts. However, the vulnerability of low-income and high-burden countries to reductions in development assistance for health is also a crucial finding, capturing the risk posed by future reductions in development assistance for health for HIV/AIDS and the vigilance required to ensure UNAIDS Fast-Track Targets and SDG target 3.3 are achieved.^16^, ^31^ Despite these advances in health resource tracking, we know little about how patterns in HIV/AIDS spending contrast with spending on other disease areas. Estimates for a wider set of diseases are needed to fully understand what is being purchased, with $9·7 trillion being spent on health in 2015. It is increasingly important—and possible—to track health spending with the precision and granularity to inform policy, investigate effectiveness, and identify areas where more investment could lead to improved health. Disease-specific resource tracking is an essential tool for understanding health markets and health policy, and for deploying that knowledge to improve health.

Correspondence to: Dr Joseph L Dieleman, Institute for Health Metrics and Evaluation, Seattle, WA 98121, USA **dieleman@uw.edu**
