# Supplementary material for: Spending on health and HIV/AIDS: domestic health spending and development assistance in 188 countries, 1995–2015
Source: Lancet. 2018 May 5;391(10132):1799–829. doi: 10.1016/S0140-6736(18)30698-6 (PMC5946845; doi:10.1016/S0140-6736(18)30698-6)
Supplement: Supplementary appendix [file mmc1.pdf]

THE LANCET

Supplementary appendix

This appendix formed part of the original submission and has been peer reviewed. We post it as supplied by the authors.

Supplement to: Global Burden of Disease Health Financing Collaborator Network. Spending on health and HIV/AIDS: domestic health spending and development assistance in 188 countries, 1995–2015. *Lancet* 2018; published online April 17. http://dx.doi.org/10.1016/S0140-6736(18)30698-6.

SUPPLEMENTARY METHODS ANNEX

Tracking spending on health and HIV/AIDS: 188 countries, 1995-2015

Version: April 13, 2018

Global Burden of Disease Health Financing Collaborator Network

SECTION 0: AUTHORS' CONTRIBUTIONS

Managing the estimation process

Abigail Chapin, Joseph Dieleman, Annie Haakenstad, Angela Micah, Mark Moses.

Writing the first draft of the manuscript

Joseph Dieleman, Annie Haakenstad, Angela Micah, Mark Moses.

Providing data or critical feedback on data sources

Cristiana Abbafati, Tara Ballav Adhikari, Kouablan Arsene Adou, Aliasghar Ahmad Kiadaliri, Khurshid Alam, Ala'a Alkerwi, Walid Ammar, Olatunde Aremu, Solomon W. Asgedom, Ashish Awasthi, Rakesh Ayer, Maciej Banach, Amrit Banstola, Aleksandra Barac, Charles Birungi, Nicola Luigi Bragazzi, Josip Car, Ferrán Catalá-López, Lalit Dandona, Rakhi Dandona, Ahmad Daryani, Samath Dharmaratne, Joseph Dieleman, Manisha Dubey, Maysaa El Sayed Zaki, Babak Eshrati, Andre Faro, Andrea Feigl, Ruoyan Gai, Mamata Ghimire, Alemayehu Hailu, Hilda Harb, Delia Hendrie, Trang Huyen Nguyen, Mehdi Javanbakht, Denny John, Jost B. Jonas, Amir Kasaeian, Ibrahim Khalil, Young-Ho Khang, Jagdish Khubchandani, Yun Jin Kim, Soewarta Kosen, Kristopher Krohn, G Anil Kumar, Hilton Lam, Hassan Magdy Abd El Razek, Muhammed Magdy Abd El Razek, Azeem Majeed, Reza Malekzadeh, Atte Meretoja, Ted Miller, Erkin Mirrakhimov, Shafiu Mohammed, Mohsen Naghavi, Frida Ngalesoni, Mayowa Owolabi, David Pereira, Mostafa Qorbani, Anwar Rafay, Alireza Rafiei, Vafa Rahimi-Movaghar, Rajesh Kumar Rai, Chhabi Lal Ranabhat, Maheswar Satpathy, Sadaf Sepanlou, Masood Ali Shaikh, Mehdi Sharif, Aziz Sheikh, Mekonnen Sisay, Cuong Tat Nguyen, Khanh Bao Tran, Bach Tran , Tung Tran Thanh, Eduardo Undurraga, Tommi Vasankari, Francesco S Violante, Tissa Wijeratne, Gelin Xu, Ebrahim M. Yimer, Naohiro Yonemoto, Mustafa Younis, Chuanhua Yu.

Developing methods or computational machinery

Catherine Chen, Joseph Dieleman, Erika Eldrenkamp, Kyle Foreman, Gloria Ikilezi, Angela Micah, Robert Reiner, Nafis Sadat, Matthew Schneider, Tianchhan Tao, Tara Templin, Bianca Zlavog.

Applying analytical methods to produce estimates

Khurshid Alam, Solomon W. Asgedom, Catherine Chen, Ahmad Daryani, Joseph Dieleman, Erika Eldrenkamp, Gloria Ikilezi, Ibrahim Khalil, Hassan Magdy Abd El Razek, Angela Micah, Shafiu Mohammed, Alireza Rafiei, Nafis Sadat, Abdur Razzaque Sarker, Matthew Schneider, Mehdi Sharif, Tianchhan Tao, Tissa Wijeratne, Ebrahim M. Yimer, Bianca Zlavog.

Providing critical feedback on methods or results

Cristiana Abbafati, Pawan Acharya, Tara Ballav Adhikari, Kouablan Arsene Adou, Aliasghar Ahmad Kiadaliri, Khurshid Alam, Reza Alizadeh-Navaei, Ala'a Alkerwi, Walid Ammar, Carl Abelardo Antonio, Olatunde Aremu, Solomon W. Asgedom, Tesfay Mehari Atey, Leticia Avila-Burgos, Ashish Awasthi, Hamid Badali, Maciej Banach, Amrit Banstola, Aleksandra Barac, Abate Bekele, Charles Birungi, Nicola Luigi Bragazzi, Nicholas Breitborde, Lucero Cahuana-Hurtado, Josip Car, Deborah Carvalho Malta, Ferrán Catalá-López, Lalit Dandona, Rakhi Dandona, Ahmad Daryani, Samath Dharmaratne, Manisha Dubey, Dumessa Edessa, Maysaa El Sayed Zaki, Babak Eshrati, Andre Faro, Andrea Feigl, Ama Fenny, Florian Fischer, Nataliya Foigt, Kyle Foreman, Nancy Fullman, Ruoyan Gai, Srinivas Goli, Alemayehu

Hailu, Samer Hamidi, Hilda Harb, Simon Hay, Delia Hendrie, Trang Huyen Nguyen, Mehdi Javanbakht, Denny John, Jost B. Jonas, Amir Kasaeian, Young-Ho Khang, Jagdish Khubchandani, Yun Jin Kim, Jonas Kinge, Kristopher Krohn, G Anil Kumar, Hilton Lam, Yirga Legesse, Hassan Magdy Abd El Razek, Muhammed Magdy Abd El Razek, Azeem Majeed, Reza Malekzadeh, George Mensah, Atte Meretoja, Ted Miller, Erkin Mirrakhimov, Shafiu Mohammed, Vinay Nangia, Frida Ngalesoni, Mehdi Noroozi, Mayowa Owolabi, David Pereira, Mostafa Qorbani, Anwar Rafay, Alireza Rafiei, Vafa Rahimi-Movaghar, Rajesh Kumar Rai, usha ram, Chhabi Lal Ranabhat, Sarah Ray, Haniye S Sajadi, João Vasco Santos, Abdur Razzaque Sarker, Benn Sartorius, Maheswar Satpathy, Miloje Savic, Sadaf Sepanlou, Masood Ali Shaikh, Mehdi Sharif, Jun She, Aziz Sheikh, Mekonnen Sisay, Samir Soneji, Moslem Soofi, Listl Stefan, Cuong Tat Nguyen, Tara Templin, Azeb Tesema, Subash Thapa, Alan Thomson, Roman Topor-Madry, Khanh Bao Tran, Bach Tran , Tung Tran Thanh, Eduardo Undurraga, Tommi Vasankari, Francesco S Violante, Fitsum Weldegebreal, Tissa Wijeratne, Gelin Xu, Ebrahim M. Yimer, Naohiro Yonemoto, Mustafa Younis, Chuanhua Yu, Lei Zhu.

Drafting the work or revising is critically for important intellectual content

Khurshid Alam, Solomon W. Asgedom, Ashish Awasthi, Hamid Badali, Amrit Banstola, Charles Birungi, Abigail Chapin, Ahmad Daryani, Joseph Dieleman, Manisha Dubey, Maysaa El Sayed Zaki, Andre Faro, Florian Fischer, Nataliya Foigt, Annie Haakenstad, Jennifer Kates, Kristopher Krohn, Hilton Lam, Hassan Magdy Abd El Razek, Muhammed Magdy Abd El Razek, Azeem Majeed, Angela Micah, Mark Moses, Christopher Murray, Frida Ngalesoni, Mayowa Owolabi, Anwar Rafay, Alireza Rafiei, Vafa Rahimi-Movaghar, Abdur Razzaque Sarker, Maheswar Satpathy, Mehdi Sharif, Bach Tran , Tung Tran Thanh, Ebrahim M. Yimer.

Extracting, cleaning, or cataloging data; designing or coding figures and tables

Catherine Chen, Erika Eldrenkamp, Gloria Ikilezi, Nafis Sadat, Matthew Schneider, Tianchan Tao, Bianca Zlavog.

Managing the overall research enterprise

Abigail Chapin, Joseph Dieleman, Christopher Murray.

Table of Contents

SECTION 0: AUTHORS' CONTRIBUTIONS 2

SECTION 1. INTRODUCTION 8

SECTION 2. TRACKING DEVELOPMENT ASSISTANCE FOR HEALTH 9

Overview 9

Tracking development assistance for health from bilateral aid agencies and the European Commission 27

Tracking development assistance for health from the development banks 38

Tracking contributions from GFATM and GAVI 44

Tracking expenditure by United Nations Agencies active in the health domain 47

Tracking development assistance for health from private foundations 50

Tracking non-governmental organizations 51

Calculating the technical assistance and program support component of development assistance for health from loan-and grant-making channels of assistance 56

SECTION 3. TRACKING TOTAL HEALTH SPENDING AND ITS COMPONENTS 59

Overview of GHED data cleaning process 59

Statistical model to fill missingness in health expenditure variables 79

SECTION 4. TRACKING GLOBAL HIV SPENDING 81

Overview of data sources and data cleaning process 81

Currency exchange and deflation 82

Currency exchange and deflation 82

Modeling HIV/AIDS spending with ST-GPR 83

REFERENCES 92

Abbreviations

ADB	Asian Development Bank and
AfDB	African Development Bank
BMGF	Bill & Melinda Gates Foundation
CRS	Creditor Reporting System
DAC	Development Assistance Committee
DAH	Development assistance for health
EC	European Commission
GDP	Gross domestic product
GFATM	Global Fund for AIDS, Tuberculosis and Malaria
GGHE	General government health expenditure
GHED	Global Health Expenditure Database
GHES	Government health spending (Aggregate of "Social insurance contributions", "Transfers from government domestic revenue (allocated to health purposes)", and "Compulsory prepayment (Other, and unspecified, than FS.3)" as defined in GHED)
HSS	Health System Strengthening
IBRD	International Bank for Reconstruction and Development
IDA	International Development Association
IDB	Inter-American Development Bank
LCU	Local currency units
NASA	National AIDS Spending Assessments
NGO	Non-governmental organizations
NHA	National Health Accounts
NPISH	Non-profit institutions serving households
ODA	Official development assistance
OECD	Organisation for Economic Co-operation and Development
OOP	Out-of-pocket (defined in GHED: “Other revenues from households n.e.c")
PAHO	Pan American Regional Office for WHO
PI	Private insurance
PPP	Pre-paid private (Aggregate of "Voluntary prepayment", "Other revenues from corporations n.e.c." and "Other revenues from NPISH n.e.c." as defined in GHED)
SHA 2011	System of Health Accounts 2011

SWAps	Sector Wide Approaches
TB	Tuberculosis
THE	Total health expenditure (defined in GHED: “Current health expenditure by revenues of health care financing schemes”)
UNICEF	United Nations Children’s Fund
UNAIDS	Joint United Nations Programme on HIV/AIDS
UNFPA	United Nations Population Fund
VolAg	Report of Voluntary Agencies
WHO	World Health Organization

List of Figures

eFigure 1 Comparing CRS commitments, CRS disbursements, and DAC commitments

eFigure 2 CRS disbursement to commitment ratio and cutoff points by donor agency

eFigure 3 One- to six-year disbursement schedules for bilateral channels

eFigure 4 DAH as a percentage of corresponding budget data by bilateral agency

eFigure 5 Malaria DAH to program areas as assigned by keyword search and PMI reports

eFigure 6 World Bank’s annual health sector commitments and disbursements

eFigure 7 Disbursements by the African Development Bank

eFigure 8 Commitments and disbursements by Asian Development Bank

eFigure 9 Commitments and disbursements by Inter-American Development Bank

eFigure 10 Contributions received by the Global Fund to Fight AIDS, Tuberculosis and Malaria

eFigure 11 The Global Fund to Fight AIDS, Tuberculosis and Malaria’s commitments and disbursements

eFigure 12 Gavi’s income and disbursements

eFigure 13 Total revenue received by non-governmental organizations

eFigure 14 Expenditure by non-governmental organizations

eFigure 15 In-kind contributions by loan- and grant-making DAH channels of assistance

eFigure 16 DAH by source as a percentage of GDP, 2016

eFigure 17. Comparison of GHED Extracted data and ST-GPR Output

List of Tables

eTable 1. Definitions of health spending sources
eTable 2. Summary of primary data sources and databases

eTable 3. Data sources received via personal correspondence

eTable 4. Additional data sources, databases and model choices used for preliminary estimates of DAH

eTable 5. Terms for keyword searches

eTable 6. Additional health focus area categorizations

eTable 7 World Bank's health sector and theme codes

eTable 8 Summary of data sources for the regional development banks

eTable 9 Summary of US non-governmental organizations in the study

eTable 10 Summary of data sources for calculating in-kind contributions

eTable 11. Rules for assigning level values to metadata

eTable 12. Supplemental table for data classification

eTable 13. Supplemental table: Gross Domestic Product

eTable 14. Supplemental table: Total expenditure on health

eTable 15. Supplemental table: General government expenditure on health

eTable 16. Supplemental table: Out of pocket expenditures

eTable 17. Supplemental table: Pre-paid private

eTable 18. Count of data sources, by financing sources and spending functions

eTable 19. Count of data sources, by GBD super region

Box 1. EXAMPLE - Australia's primary and additional data sources

Box 2. EXAMPLE. Post-keyword search weighting

Box 3. EXAMPLE. Australia's CRS disbursement to commitment ratio and cutoff year

Box 4. EXAMPLE. Australia's one- to six-year disbursement schedules

Box 5. EXAMPLE. Australia's DAH as a percentage of corresponding budget data

SECTION 1. INTRODUCTION

Reliable and complete data on global health spending is important for resource mobilization, planning, resource allocation and monitoring the health-related targets in the Sustainable Development Goals. The objective of this study is to describe global spending on health with a special and new focus on disease-specific spending. In addition, to presenting data on development assistance for health and total health spending globally and disaggregated by source, this study presents an assessment of spending on HIV/AIDS in 188 countries.

This appendix describes in detail the methodology used in each of the three analyses. It provides a detailed description of the sources of data and estimation techniques and assumptions. Section 2 details how funds for development assistance for health are tracked. Section 3 describes how total health spending and its component sources of funding are disaggregated. Section 4 describes the data and modeling techniques used to estimate HIV spending. eTable 1 below presents the definitions for the various health spending sources.

eTable 1. Definitions of health spending sources

Health Spending Type	Definition
Development assistance for health	Financial and in-kind contributions from global health channels that aim to improve or maintain health in low- or middle-income countries.
Government health expenditure as source	Government health expenditure as source only includes domestically-financed government expenditure on health.
Out-of-pocket spending	Payment by individuals for health services, and are considered catastrophic if exceeding 40% of a household’s annual income.
Pre-paid private health spending	Private risk pooling against catastrophic health expenditure, includes private insurance and non-governmental organizations.

SECTION 2. TRACKING DEVELOPMENT ASSISTANCE FOR HEALTH

Overview

Development assistance for health (DAH) estimates were obtained from the Institute for Health Metrics and Evaluation's development assistance for health database. We summarize the original methodology as well as updates for this year's estimates below. A more detailed description of the original methodology used to obtain the estimates in the database can be found in Dieleman et al.[1] All known, systematically reported, available data on health-related disbursements and expenditures were extracted, as well as income and revenue from existing project databases, annual reports, and audited financial statements. The channels included and the corresponding data sources are summarized in eTable 2. Data sources obtained via personal correspondence are summarized in eTable 3.

DAH for bilateral agencies included all health-related disbursements from bilateral donor agencies, excluding funds that they transferred to any of the other channels we tracked in order to avoid double-counting. This information was extracted from the Creditor Reporting System (CRS) and Development Assistance Committee (DAC) databases of the Development Assistance Committee of the Organisation for Economic Co-operation and Development (OECD-DAC). In some cases, donor agencies did not report disbursement data to the CRS. A method for predicting disbursements from commitment data was implemented to address this challenge. For detailed description of this method see Tracking Development Assistance for Health from Bilateral Aid Agencies and the European Commission section below as well as in Dieleman et al. [1]

For other grant- and loan-making institutions, annual disbursements on health grants and loans were similarly included, excluding transfers to any other channels and ignoring any repayments on outstanding debts. For a more detailed description of this process see Dieleman et al.[1] The annual disbursements for grant- and loan-making institutions only reflect the financial transfers made by these agencies. Therefore, in-kind transfers from these institutions in the form of staff time for providing technical assistance and the costs of managing programs were estimated separately.[1]

Estimates of DAH for the United Nations (UN) agencies included annual expenditures on health both from their core budgets and from voluntary contributions. Calculating DAH for the United Nations Children's Fund (UNICEF) involved estimating the fraction of its total expenditure spent on health prior to 2001.[1]

Non-governmental organizations' (NGOs) DAH estimates utilized data from US government sources and a survey of health expenditure for a sample of NGOs to estimate DAH from US-based and internationally based NGOs receiving support from the US government. We were unable to include other NGOs due to the lack of audited and comparable data.

The database also included an analysis of the composition of health funding by recipient country, as well as by health focus area. In this round of updates to the databases we have made several improvements. These improvements include the inclusion of UNITAID as a channel, addition of new program areas – treatment and diagnosis – under the Tuberculosis health focus area, addition of pandemic preparedness as a program area under Sector Wide Approaches and Health system strengthening (SWAps/HSS) and modifications to our health focus area key word search terms. All methodological updates made are detailed in their relevant channel sub-section below. The improvements to our health focus area keyword search terms are detailed in eTable 5 and in the section below titled Disaggregation by health focus area, respectively.

For many channels, reporting-time lags prevent primary disbursement data for the most recent year(s). For those years, the values of DAH were predicted, using channel-specific time trends. The methods employed to obtain these predictions are summarized in eTable 4. In general, these methods depend on data availability. The estimates are based on channel-specific budget, commitment, and appropriations data, and in many cases assume the most recent disbursement patterns persist. Due to the lack of more detailed disaggregated data, estimates for the most recent two years are not provided for recipient countries.

Specific methodological updates made this year include improvements to our ebola DAH estimation process, allocation process for SWAp/HSS and the reallocation of DAH estimates to newly created countries.

We predicted ebola funding in 2017 for bilateral sources and the European Commission by assuming that 2017 ebola funding was equal to 2016 ebola funding.

In addition, for SWAp/HSS funding, we allocated SWAP/HSS projects with multiple health focus areas identified by a proportional allocation based on the relative proportions of the project going to the various health focus areas.

For countries that only began existing in certain years, we backcasted DAH in years before their existence as follows. For countries that split off from parent countries, we calculated a three-year average ratio of child country DAH received to parent country DAH received. In years before the child country split off, DAH received by the parent country would have included DAH received in the region that would split off to become the child country. Therefore we reallocated funding from the parent country, in all years before the child country split off, adding this proportion of the parent country's DAH to the child country's DAH and subtracting out this value from the parent country's DAH. By this method, total annual DAH between the parent and child country do not change, but the allocation of funding between the parent country and child country change. For any country that ceased to exist (such as former Yugoslavia and former USSR) and that had observed DAH received in certain years, we split the funding equally among its new constituent countries.

Currency exchange and deflation

All results are presented in real 2017 US dollars. All disbursement sequences were converted into real 2017 US dollars by taking disbursements in nominal US dollars in the year of disbursement and adjusting these sequences into real 2017 US dollars using US gross domestic product (GDP) deflators. Analyses were conducted in Stata (version 13.1).

eTable 2. Summary of primary data sources and databases

Channel	Source
Bilateral agencies	OECD-DAC and CRS databases[2]
European Commission	OECD-DAC and CRS databases[3]
Joint United Nations Programme on HIV/AIDS (UNAIDS)	Financial reports and audited financial statements[4]
United Nations Children's Fund (UNICEF)	Financial reports and audited financial statements[5–7]
United Nations Population Fund (UNFPA)	Financial reports and audited financial statements[8]
UNITAID	Financial reports and audited financial statements[9]
Pan American Health Organization (PAHO)	Financial reports and audited financial statements[10]
World Health Organization (WHO)	Financial reports and audited financial statements[11]
World Bank	Online project database and correspondence[12,13]
Asian Development Bank (ADB)	Online project database[14]
African Development Bank (AfDB)	Online project database and compendium of statistics[15,16]
Inter-American Development Bank (IDB)	Online project database and correspondence[17,18]
Gavi, the Vaccine Alliance	Online project database, cash received database, International Finance Facility for Immunisation (IFFIm) annual reports, Advance Market Commitment for Pneumococcal Vaccines (AMC) annual reports, and annual reports[19–22]
The Global Fund to Fight AIDS, Tuberculosis and Malaria (GFATM)	Online grant database, contributions report and annual reports[23–25]
NGOs registered in the US	United States Agency for International Development (USAID) Report of Voluntary Agencies (VolAg), tax filings, annual reports, financial statements, RED BOOK Expanded Database, and WHO's Model List of Essential Medicines[26–29]
Bill & Melinda Gates Foundation (BMGF)	Online grant database, IRS 990 tax forms, and correspondence[30,31]
Other private US foundations	Foundation Center's grants database[32]

eTable 3. Data sources received via personal correspondence

Channel	Data received
World Bank	Health project-level disbursement data, 1990 – September 2016[13]
BMGF	Health disbursement data, 2015[31]
IDB	Health project-level loan disbursement data, 2016[18]
African Development Bank	Health project-level disbursement data, 2001 – October 2017[33]
UNITAID	Health project-level disbursement data, 2007- 2016[34]
UAE	UAE Foreign Assistance in Health 1990 - 2008[35]

eTable 4. Additional data sources, databases and model choices used for preliminary estimates of DAH

Channel	Data source	Variables used	Years of budget data used for modeling*	Years underlying DAH data not available; thus modeled*	Model used
**National agencies**					
Australia	Australia's International Development Assistance (2008-2016); Australia's Overseas Aid Program (1998-2008)[36,37]	Health official development assistance (ODA): International development assistance budget	1998-2017	2016-2017	Weighted average of actual DAH/budgeted DAH
Austria	Austria Federal Ministry of Finance budget[38]	General ODA: Federal ODA budget	2007-2017	2016-2017	Weighted average of DAH/budgeted ODA
Belgium	Project Budget General – general expenses[39]	General ODA: Foreign affairs, foreign trade development and cooperation	2000-2017	2016-2017	Weighted average of DAH/budgeted ODA
Canada	Canadian International Development Agency – Report on Plans and Priorities[40]	General ODA: Financial summary – planned spending	1996-2017	2016-2017	Weighted average of DAH/budgeted ODA
Denmark	Danish Ministry of Foreign Affairs Budget[41]	General ODA: Budgeted expenditures on overseas development assistance	2000-2017	2016-2017	Weighted average of DAH/budgeted ODA
European Commission	General budget[42]	Data not used as they were inconsistent with disbursements	–	2016-2017	Based on weighted average of trends in member countries
Finland	Document Assembly in budget years 1998-2016[43]	General ODA: Ministry of Foreign Affairs' administrative appropriations, international development	2002-2017	2016-2017	Weighted average of DAH/budgeted ODA
France	Budget and Financial documents[44,45]	General ODA: aggregated project data; Total ODA	2009-2017	2016-2017	Weighted average of DAH/budgeted ODA
Germany	Plan of the Federal Budget[46]	General ODA: Development expenditure	2001-2017	2016-2017	Weighted average of DAH/budgeted ODA

Channel	Data source	Variables used	Years of budget data used for modeling*	Years underlying DAH data not available; thus modeled*	Model used
Greece	Ministry of Finance Budget (2013-2016); OECD Data (1996-2012)[2,47,48]	General ODA; ODA commitments	1996-2014	2016-2017	Weighted average of DAH/budgeted ODA
Ireland	Department of Finance – budget 2000-2004; Estimates for Public Services and Summary Public Capital Programme, 2005-2016 [49]	General ODA: Summary of adjustments to gross current estimates – international co-operation	2002-2017	2016-2017	Weighted average of DAH/budgeted ODA
Italy	The Italian Agency for Development Cooperation[50]	General ODA: Net development corporation	2007-2017	2016-2017	Weighted average of DAH/budgeted ODA
Japan	Highlights of the Budget for FY1999-2016[51]	General ODA: Major budget expenditures	2003-2017	2016-2017	Weighted average of DAH/budgeted ODA
Korea, South	ODA Korea comprehensive implementation plan[52]	General ODA: Plan for international development cooperation	2008-2017	2016-2017	Weighted average of DAH/budgeted ODA
Luxembourg	State Budget[53]	General ODA: Ministry of Foreign Affairs – budgeted international development cooperation and humanitarian aid	2001-2017	2016-2017	Weighted average of DAH/budgeted ODA
Netherlands	Netherlands International Cooperation Budget (2001-2016)	General ODA: Total annual official development assistance expenditure	2001-2017	2016-2017	Weighted average of DAH/budgeted ODA
New Zealand	Vote Foreign Affairs and Trade (1998-2001); VOTE Official Development Assistance (2002-2016)[54]	General ODA: Total annual official development assistance expenditure	1998-2017	2016-2017	Weighted average of DAH/budgeted ODA

Channel	Data source	Variables used	Years of budget data used for modeling*	Years underlying DAH data not available; thus modeled*	Model used
Norway	Norwegian Ministry of Finance National Budget (2014-2016); Correspondence (2000-2013)[55,56]	General ODA: ODA budget	2000-2017	2016-2017	Weighted average of DAH/budgeted ODA
Portugal	Ministry of Finance and Public Administration State Budget 2003-2016[57]	General ODA: Integrated service expenditure – external cooperation budget	2003-2017	2016-2017	Weighted average of DAH/budgeted ODA
Spain	Annual Plans of Spanish International Cooperation[58]	General ODA: Spanish total development cooperation	2003-2017	2016-2017	Weighted average of DAH/budgeted ODA
Sweden	Correspondence (2000-2010); Ministry of Foreign Affairs Budget (2010-2016)[59]	General ODA: Ministry for Foreign Affairs budgets for expenditure – international development cooperation	2000-2017	2016-2017	Weighted average of DAH/budgeted ODA
Switzerland	Foreign Affairs (2000-2006); Budget – Further Explanations and Statistics (2007-2016)	General ODA: Direction of development and cooperation (2000-2006); foreign affairs – international cooperation, development aid (in the South and East) (2007-2016)	2000-2017	2016-2017	Weighted average of DAH/budgeted ODA
United Kingdom	IATA (Department for International Development (DFID))[60,61]	General ODA: assistance for international development; Sum (revised) - aggregated project data	1998-2017	2016-2017	Weighted average of DAH/budgeted ODA
United States	Foreign Assistance Dashboard (2006-2016); Budget of the US Government (2005-2016)[62,63]	Global health ODA: Planned foreign assistance for health; Department of Health and Human Services global health budget	2005-2017	2016-2017	Weighted average of actual DAH/budgeted DAH
UN agencies					
WHO	Programme budget[64]	DAH budget: Programme budget	2002-2017	2016- 2017	Weighted average of DAH/budget

Channel	Data source	Variables used	Years of budget data used for modeling*	Years underlying DAH data not available; thus modeled*	Model used
UNAIDS	Unified Budget and Workplan, bienniums 2002-2017 and 2018-2019[65,66]	DAH budget: Unified Budget and Workplan	2002-2017	2016- 2017	Weighted average of DAH/Core Budget
UNICEF	Financial report and audited financial statements; correspondence[7,67,68]	Total expenditure; Total health expenditure	2001-2016	2016-2017	Weighted average of DAH/budget
UNFPA	Audited Financial report and contributions report [69,70]	Total health expenditure	2002-2016	2016-2017	Weighted average of DAH/budget
PAHO	Proposed program budget[10]	Total regular budget, estimated voluntary contributions	2000-2017	2016-2017	Weighted average of DAH/budget
NGOs	VolAg (1990-2011), GuideStar (2014), sample of top NGOs (2011-2012) [26,27]	Revenue breakdowns for: US public, non-US public, private, in-kind, BMGF; total overseas expenditures	1990-2014	2014-2017	Regression on DAH, US GDP, and USAID and private voluntary organization (PVO) revenue

* Years of budget data used for modeling versus years underlying DAH data unavailable thus modeled: The data used to estimate DAH by channel vary across channels. eTable 2 reports our primary data used for each channel. Due to reporting lags there are some years we need to estimate disbursement using additional data sources. These additional data sources, the years in which the primary data is modeled, the years the additional data is available, and the methods for this estimating these modeled years are reported in eTable 4. Years of budget data used for modeling are the years of additional data available to us. We rely on historic trends to inform our estimates so we rely on many years of additional data despite only modeling a few years of primary data. Years underlying DAH data unavailable thus modeled are the years the primary data is incomplete and thus estimated using additional data. See example below for more details for Australia

Box 1. EXAMPLE - Australia's primary and additional data sources

Project-level data for health-related projects funded by Australia's bilateral aid agencies are available from the OECD's CRS database through 2015. This is the primary data source used to estimate DAH channeled by Australian aid agencies, as described in eTable 2. 2016-2017 are incomplete because of lags in reporting. To estimate DAH disbursed for 2016 and 2017, additional data are available from Australia's International Development Assistance budget (2008-2017) and Australia's Overseas Aid Program budget (1998-2008), as described in eTable 4. These sources provide health-specific official development assistance (ODA) budgeted by Australia, 1998-2017. We convert countries' budgeted ODA, as given in nominal local currency units, to nominal US dollars using the OECD's currency exchange rate series based on USD monthly averages. To estimate DAH disbursed 2016-2017, we calculated the ratio of disbursed DAH (from the CRS database) relative to budgeted DAH (from the International Development Assistance and Overseas Aid Program budgets) for 1998-2015. We combine the most recent three ratios into a single estimate by taking a weighted average, weighting substantially higher the most recent year. We multiply this ratio – the estimated disbursed DAH to budgeted DAH – by the 2016 and 2017 budgeted DAH to estimate disbursed DAH in those years. These methods are described more fully in Dieleman et al.[1]

DISAGGREGATING BY HEALTH FOCUS AREA

We improved our analysis of the disaggregation of health funding by health focus areas by augmenting our keyword search terms. In particular, we added new keywords to the Non-communicable diseases, SWAps/HSS for pandemic preparedness, and TB for treatment and diagnosis. Similar to our previous work, the analysis of health focus areas included assessments of development assistance for HIV/AIDS, tuberculosis (TB), malaria, maternal health, newborn and child health, other infectious diseases, non-communicable diseases, and SWAps and health system strengthening, using keyword searches within descriptive fields. These were chosen as the areas of focus because of their relevance to current policy debates about global health financing and data availability.

In effect, DAH was disaggregated into eight health focus areas: HIV/AIDS; tuberculosis; malaria; maternal, newborn and child health; non-communicable diseases; SWAps/health sector support; other infectious diseases; and other. For most data sources, project-level data were available only through 2015. Methods to estimate health focus area allocations for 2016 and 2017 are described in more detail below. Keyword searches were performed for a subset of global health channels that provide project-level data with project titles or descriptions. These sources include the bilateral development assistance agencies from 24 DAC member countries, one DAC participant country, GFATM, the World Bank, Asian Development Bank, African Development Bank, Inter-American Development Bank, Bill & Melinda Gates Foundations, Non-government Organizations, and US foundations. The keywords used are outlined in eTable 5 below. Descriptive fields were adjusted so that they were in all capitalized letters, and search terms with multiple words were put between quotation marks. All keywords were translated into nine major languages (English, Spanish, French, Portuguese, Italian, Dutch, German, Norwegian, and Swedish) used in the OECD CRS, checked for double meanings across all languages, and adjusted accordingly.

Total DAH was split across the health focus areas using weighted averages based on the number of keywords present in each project's descriptive variables. If, for example, three keywords suggested the project focused on HIV/AIDS and two keywords related to tuberculosis were also tagged, three-fifths of the project's total DAH was allocated to HIV/AIDS and two-fifths was allocated to tuberculosis. To account for the sensitivity of this method, several checks were implemented after the keyword searches to ensure the project was accurately categorized. First, projects that were tagged as child and newborn vaccines and other infectious diseases were categorized as child and newborn vaccines only. Second, projects that were tagged as one of the three major infectious diseases (HIV/AIDS, tuberculosis, or malaria) and other infectious diseases were categorized under only HIV/AIDS, tuberculosis, or malaria.

**Box 2. EXAMPLE. Post-keyword search weighting**
A project in the CRS database had a value of $1,000 of DAH. A keyword search conducted on this project's title and description tagged five keywords: 3 keywords related to HIV/AIDs and 2 keywords related to tuberculosis. Therefore, $600, or 3/5 of total DAH, was allocated to HIV/AIDS, while $400, or 2/5 of total DAH, was allocated to tuberculosis.

In addition to keyword searches, funds were allocated to health focus areas based on characteristics of the channel or additional channel variables. For the bilateral agencies and the EC, purpose codes from the CRS were used to supplement keyword searches. For the World Bank-IDA and -IBRD, health focus areas were also determined by the project sector codes and theme codes, which included percentages of health funds that targeted each theme. All funds from Gavi were allocated to child and newborn vaccines, health system strengthening and non-communicable diseases and all funds from UNICEF to maternal, newborn, and child health, unspecified. Funds from GFATM were distributed to malaria, HIV/AIDS, TB, and health sector support based on disease components. Within each disease component, keyword searches on programmatic budget data and project descriptions were conducted to distribute among program areas. Funds from UNAIDS were allocated to HIV/AIDS, and specific program areas were determined by budget information. UNFPA, PAHO, and WHO funds were allocated to specific health focus areas based on project expenditure data from their annual reports and annual financial reports. For all channels, projects

listed as HIV/TB were distributed evenly among the two health focus categories. See eTable 6 below for more details on these categorizations.

eTable 5. Terms for keyword searches

Health focus area level	Program area	Keywords
HIV/AIDS	HIV envelope/unidentified	" HUMANIMMUNODEFVIRUS " " SIDA " " OVC " " H I V " " HIV " " AIDS " " HUMAN IMMUNODEFICIENCY " " REVERSE TRANSCRIPTASE INHIBITOR " " ACQUIRED IMMUNE DEFICIENCY SYNDROME " " ACQUIRED IMMUNNODEFICIENCY " " RETROVIRAL " " VCT " " MALE CIRCUMCISION " " ART " " ARV " " CD4 COUNT " " HAART " " PMTCT " " MOTHER TO CHILD TRANSMISSION" " MOTHER TO CHILD AIDS TRANSMISSION" " PARENT TO CHILD TRANSMISSION" " PRESIDENT S EMERGENCY PLAN FOR AIDS RELIEF " " PEPFAR " " THREE DISEASE FUND " " 3 DISEASE FUND "
	Care and Support	" CAREANDSUPPORT " " CARE ACTIVIT" " PAIN RELIEF " " SYMPTOM RELIEF " " SOCIAL SUPPORT " " CHRONICALLY ILL " " CLINICAL MONITORING " " CARE AND SUPPORT " " PSYCHOLOGICAL SERVICE" " PSYCHOLOGICAL SUPPORT " " PSYCHOSOCIAL SUPPORT " " PSYCHOSOCIAL SERVICE" " MATERIAL SUPPORT " " HEALTH CARE "
	Counseling and Testing	" COUNSELING " " TESTING " " VCT " " COUNSELLING " " COUNSELINGANDTESTING " " DIAGNOS"
	Orphans and Vulnerable Children	" VULNUERABLECHILD" " OVC " " ORPHAN" " VULNERABLE CHILD" " INFECTED CHILD" " VULNERABLE GROUP" " MOST AT RISK "
	Prevention of mother-to-child transmission (PMTCT)	" MOTHERTOCHILD" " MOTHER TO CHILD" " PARENT TO CHILD" " PMTCT " " EMTCT "
	Prevention	" CONDOM" " PREVENT" " HIV EDUCATION " " AIDS EDUCATION " " REDUCING THE TRANSMISSION OF HIV " " REDUCE THE TRANSMISSION OF HIV " " MALE CIRCUMCISION" " SAFE BLOOD SUPPL" " SAFE INJECTION" " ABSTINENCE " " AWARENESS " " BLOOD SAFETY " " MICROBICIDE"
	Treatment	" RETROVIRAL " " TREAT" " ART " " ARV " " CD4 COUNT " " HAART " " VIRAL LOAD " " VIRAL BURDEN " " VIRAL TITER " " ESSENTIAL SERVICE" " DRUG REGIMENS " " IMPACT REDUCTION " " REDUCE IMPACT "
Tuberculosis	Tuberculosis envelope/unidentified	" TUBERCULOSIS " " TB " " TBC " " TUBERCULAR" " DOTS " " DIRECTLY OBSERVED TREATMENT " " RIFAMPICIN " " ISONIAZID " " THREE DISEASE FUND " " 3 DISEASE FUND "
	Treatment	" TREATMENT " " TREATING" " DOTS " " DIRECTLY OBSERVED TREATMENT " " FIRST LINE " " DRUGS " " RIFAMPICIN " " RIFAMPIN " " RIF " " ISONIAZID " " INH " " PYRAZINAMIDE " " PZA " " ETHAMBUTOL " " EMB " " STREPTOMYCIN " " SM " " STM " " PATIENT KIT " " SECOND LINE " " INJECTABLE AGENT" " FLUOROQUINOLONES " " REGIMEN" " CASE MANAGEMENT " " ANTIMICROBIAL THERAPY " " DRUG SUSCEPTIBLE " " DRUG SENSITIVE " " SERVICE DELIVERY "

Health focus area level	Program area	Keywords
	Diagnosis	" DIAGNOSIS " " DIAGNOSTIC " " CASE DETECTION " " MICROSCOPY " " BLOOD SURVEY " " RAPID DIAGNOSTIC TESTING " " MOBILE MALARIA CLINIC " " BIOLOGICAL TESTING " " LABORATORY SERVICES " " EDT " " LAMP " " RDT "
Malaria	Malaria envelope/unidentified	" MALARIA " " FALCIPARUM " " ANOPHELES " " ARTEMISININ " " PRIMAQUINE " " INDOOR RESIDUAL SPRAY" " INDOORRESIDUALSPRAY" " IRS " " PLASMODIUM VIVAX " " BEDNETS " " BED NETS " " SMITN " " ITN " " LLIN " " INSECTICIDAL NET" " INSECTICIDE TREATED NET" " THREE DISEASES FUND " " 3 DISEASES FUND "
	Diagnosis	" DIAGNOSIS " " DIAGNOSTIC " " CASE DETECTION " " MICROSCOPY " " BLOOD SURVEY " " RAPID DIAGNOSTIC TESTING " " MOBILE MALARIA CLINIC " " BIOLOGICAL TESTING " " LABORATORY SERVICES " " EDT " " LAMP " " RDT "
	Community outreach	" COMMUNITYOUTREACH " " OUTREACH " " COMMUNITY MOBILIZATION" " AWARE" " COMMUNICATION STRATEGY " " SOCIAL COMMUNICATION " " HEALTH EDUCATION " " PARTNERSHIP" " PUBLIC SECTOR" " ACTIVITIES NEAR COMMUNITIES "
	Vector control: bednets	" BEDNET" " BED NET" " SMITN " " ITN " " LLIN " " INSECTICIDAL NETS " " INSECTICIDE TREATED NET" " INSECTICIDE TREAT"
	Vector control: irs	" INDOORRESIDUALSPRAY" " IRS " " REDUCE THE PARASITE RESERVOIR " " FOGGING " " COILS " " LARVICID" " LARVACID" " VECTOR CONTROL" "RESIDUAL SPRAY " " RESIDUALSSPRAY " "INDOOR SPRAY" " INDOORSPRAY "
	Vector control: other than bednets and irs	" PREVENT"
	Treatment	" ARTEMISININ " " PRIMAQUINE " " ACT " " DRUG ADMINISTRATION " " TREAT " " TREATMENT " " TREATING " " CASE MANAGEMENT " " COMBINATION THERAPY " " ANTI MALARIAL " " ANTIMALARIAL "
Maternal, newborn, and child health	envelope/unidentified	" FERTILITY " " FAMILY PLANNING " " FP " " BIRTH" " WOMEN HEALTH " " WOMEN S HEALTH " " WOMENS HEALTH " " CONTRACEP" " IPPF " " INTERNATIONAL PLANNED PARENTHOOD FOUNDATION " " ABORTION" " UNFPA " " POSTPARTUM " " POST PARTUM " " MATERNAL " " MATERNITY " " MOTHERS " " MOTHERHOOD " " SBA " " ANTENATAL " " PRENATAL " " NEONATAL " " PERINATAL " " POSTNATAL " " FETUS" " FETAL" " IPTP " " REPRODUCTIVE HEALTH " " OBSTETRIC" " PREGNANCY " " RH " " REPROD " " RHCS " " SEXUAL HEALTH " " SYPHILIS " " FISTULA " " SEPSIS " " ANEMI" " ANAEMI" " FOETUS" " FOETAL " " FGM " " FEMALE GENITAL MUTILATION " " FEMALE GENITAL CUTTING " " FEMALE CIRCUMCISION " " SBAS " " OBSTRUCTED LABOR " " NUTRITION " " MALNUTRITION " " VITAMIN A " " BREAST FE" " BREASTFE" " MICRONUTRIENT" " ZINC " " FORTIFICATION " " STUNT" " WASTING " " BABY FRIENDLY HOSPITAL INITIATIVE " " BREASTMILK " " BREAST MILK " " IODINE " " IODIZED " " IODIZATION " " VAD " " LACTAT" " FOLIC ACID " " FOLAT" "

Health focus area level	Program area	Keywords
		VACCINE" " VACCINATION" " IMMUNIZ" " POLIO " " DIPHTHERIA " " TETANUS " " PERTUSSIS " " DTP " " HIB " " ROTAVIRUS " " MEASLES " " IMMUNIS" " HEPB MONO " " INJECTION SAFETY " " RUBELLA " " MENINGITIS " " PENTA " " PENTAVALENT " " PNEUMONIA " " PNEUMOCOCC" " HAEMOPHILUS INFLUENZAE " " TETRA " " GAVI " " CHILDHEALTH " " CHILD HEALTH " " CHILDREN " " INFANT " " NEWBORN " " CHILD MORTALITY " " UNDER FIVE MORTALITY " " CHILD SURVIVAL " " CHILDHOOD ILLNESS" " LRI " " RESPIRATORY INFECTION" " DIARRHEA" " DIARRHOEA" " ORAL REHYDRATION " " ORT " " ORS " " UNICEF " " MNCH" " RNCH " " RCH " " RNH " " MNH " " MCH " " EMAS " " MCNH " " PMNCH " " WOMEN AND CHILDREN " " PRE ECLAMPSIA " " PRETERM " " POLIOVIRUS "
	Maternal health, family planning	" FERTILITY " " FAMILY PLANNING " " FP " " BIRTH SPACING " " CONTRACEPT" " FAMILY SIZE" " IPPF " " INTERNATIONAL PLANNED PARENTHOOD FOUNDATION " " ABORTION" " REDUCED FERTILITY " " UNFPA " " REDUCE FERTILITY " " BIRTH CONTROL "
	Maternal health, unspecified	" POSTPARTUM " " POST PARTUM " " MATERNAL HEALTH " " MATERNAL MORTALITY " " MATERNAL DEATH " " SAFE MOTHERHOOD " " BIRTH ATTENDANT" " SBA " " MATERNAL AND INFANT HEALTH " " ANTENATAL " " PRENATAL " " NEONATAL " " PERINATAL " " POSTNATAL " " FETUS" " FETAL" " IPTP " " REPRODUCTIVE HEALTH " " MATERNITY " " OBSTETRIC" " PREGNANCY " " RH " " REPROD " " RHCS " " STD " " STI " " SEXUAL HEALTH " " SEXUALLY TRANSMITTED " " SYPHILIS " " FISTULA " " WOMEN S HEALTH " " WOMENS HEALTH " " SEPSIS " " SEPTICEMIA " " ANEMI" " ANAEMI" " FOETUS" " FOETAL " " FGM " " FEMALE GENITAL MUTILATION " " FEMALE GENITAL CUTTING " " FEMALE CIRCUMCISION " " SBAS " " OBSTRUCTED LABOR " " DELIVERY ROOM" " CHILD DELIVERY " " MIDWIV" " MIDWIFE" " PRE ECLAMPSIA " " PRETERM "
	Child/newborn nutrition	" NUTRITION " " MALNUTRITION " " BIRTH WEIGHT " " BIRTHWEIGHT " " VITAMIN A " " BREAST FE" " BREASTFE" " FEEDING " " MICRONUTRIENT" " ZINC " " FORTIFICATION " " STUNT" " WASTING " " UNDERWEIGHT " " BABY FRIENDLY HOSPITAL INITIATIVE " " BREASTMILK " " BREAST MILK " " IODINE " " IODIZED " " IODIZATION " " VAD " " LACTAT" " FOLIC ACID " " FOLAT" " IRON "
	Child/newborn vaccines	" POLIO " " VACCINE" " VACCINATION" " IMMUNIZ" " DIPHTHERIA " " TETANUS " " PERTUSSIS " " DTP " " HIB " " ROTAVIRUS " " MEASLES " " IMMUNIS" " HEPB MONO " " INJECTION SAFETY " " RUBELLA " " MENINGITIS " " PENTA " " PENTAVALENT " " PNEUMONIA " " PNEUMOCOCC" " HAEMOPHILUS INFLUENZAE " " TETRA " " GAVI " " POLIOVIRUS "
	Child/newborn other	" CHILDHEALTH " " CHILDREN" " CHILD HEALTH " " INFANT HEALTH " " NEWBORN HEALTH " " CHILD MORTALITY " " INFANT MORTALITY " " UNDER FIVE MORTALITY " " CHILD SURVIVAL " " INFANT SURVIVAL " " CHILDHOOD

Health focus area level	Program area	Keywords
		ILLNESS" " LRI " " RESPIRATORY INFECTION" " DIARRHEA" " DIARRHOEA" " ORAL REHYDRATION " " ORT " " ORS " " UNICEF " " JAUNDICE "
Non-communicable diseases	Tobacco	" TOBACCO" " SMOK" " CIGAR"
	Mental health	" SCHIZOPHRENIA " " MENTAL HEALTH " " NEUROTIC " " NEUROSIS " " NEUROSES " " NEUROLOGICAL" " PSYCHOLOG" " PSYCHIATR" " EMOTIONAL DISORDER" " OBSESSIVE COMPULSIVE " " OCD " " PTSD " " POST TRAUMATIC " " POSTTRAUMATIC " " ALCOHOL " " ADDICTION " " DOWN SYNDROME " " DOWN S SYNDROME " " DOWNS SYNDROME " " BEHAVIORAL DISORDER" " DRUG ABUSE " " SUBSTANCE ABUSE " " OPIOID " " COCAINE " " AMPHETAMIN" " DEPRESSIVE DISORDER" " DEPRESSION " " DYSTHYMIA " " BIPOLAR " " ANXIETY " " EATING DISORDER " " AUTISM " " ASPERGER " " DEVELOPMENTAL DISORDER" " CONDUCT DISORDER" " INTELLECTUAL DISABILIT" " PHOBIA " " MENTAL DISAB" " MENTAL RETARDATION " " DRUG DEPENDENC" " ALZHEIMER" " DEMENTIA" " EPILEPSY " " MIGRAINE " " HEADACHE" " ATTENTION DEFICIT HYPERACTIVITY DISORDER " " ADHD " " PANIC DISORDER" " PARKINSON" " SELF HARM " " STRESS DISORDER" " SUBSTANCE USE DISORDER" " DRUG USE DISORDER" " MENTALLY DISAB" " NERVOUS SYSTEM" " SYNAPSE" " MENTAL ILLNESS" " MENTAL DISORDER" " PSYCHOSOCIAL " " PSYCHO SOCIAL "
	Non-communicable diseases, unspecified	" NON COMMUNICABLE " " NONCOMMUNICABLE " " CANCER" " CHEMOTHERAPY " " RADIATION " " NEOPLAS" " TUMOR " " LEUKEMIA " " LYMPHOMA " " MYELOMA " " HPV " " HUMAN PAPILLOMA VIRUS " " HEP C " " HEPATITIS C " " DIABET" " INSULIN " " ENDOCRINE " " RHEUMAT" " ISCHAEMIC " " ISCHEMIC " " CIRCULATORY " " CIRRHOSIS " " DIGESTIVE DISEASE" " OTHER DIGESTIVE " " PEPTIC " " APPENDICITIS " " GASTRITIS " " GENITOURINARY " " UROGENITAL " " MUSCULOSKELETAL " " GOUT " " BACK PAIN " " MACULAR " " HEARING " " AUDIOLOG" " PERIODONTAL " " CARIES " " CONGENITAL " " OBESITY " " OVERWEIGHT " " GLAUCOMA " " HYPERTENSI" " HERNIA " "ARTHRITIS " " CLEFT LIP" " CLEFT PALATE" " PHENYLKETONURIA " " SICKLE CELL" " DREPANOCYTOSIS " " HEMOPHILIA " " HAEMOPHILIA " " THALASSEMIA " " GENETIC DISORDER" " HEART DISEASE" " CHRONIC RESPIRATORY " " COPD " " STROKE " " CATARACT " " CATARACTS " " CHRONIC OBSTRUCTIVE PULMONARY DISEASE" " ASTHMA " " SKIN DISEASE" " DERMATITIS " " PSORIASIS " " SCABIES " " PHYSICAL DISAB" " DENTAL " " ORAL HEALTH " " CVD " " IHD " " CKD " " KIDNEY DISEASE" " MSK " " EYE " " CEREBROVASCULAR " " VASCULAR " " BLOOD PRESSURE " " ACUTE GLOMERULONEPHRITIS " " ALOPECIA AREATA " " ANEURYSM " " ANGINA " " ARTERY " " ATHEROSCLEROSIS " " ATRIAL FIBRILLATION" " ATRIAL FLUTTER " " BENIGN PROSTATIC HYPERPLASIA "

Health focus area level	Program area	Keywords
		"BLASTOMA" " BLIND " " PREVENTABLE BLINDNESS " " AVOIDABLE BLINDNESS " " BLOOD DISORDER" " BRONCHITI" " CARCINOMA " " CARDIAC " " CARDIO" " CELLULITIS " " CEREBRAL " " CORONARY " " DEAF" " DECUBITUS ULCER " "DIALYSIS" " DUODENITIS " " ECZEMA " " EKZEMA " " EDENTULISM " " ENDOCARDITIS " " FIBROSIS " " G6PD DEFICIENCY " " GALL BLADDER " " BILE DUCT " " GLYCEMI" " GLYCAEMI" " HEMOGLOBINOPATH" " HEMOLYTIC ANEMIA " " HODGKIN" " INSOMNIA " " INTERSTITIAL LUNG DISEASE" " INTESTINAL OBSTRUCTION" " LEUKAEMIA " " MELANOMA" " MULTIPLE SCLEROSIS " " MYOCARD" " NCD " " NECK PAIN " " NEPHRITIS " " NEPHROSIS " " NEURAL TUBE DEFECT" " NEURODEGENERATIVE " " INFLAMMATORY BOWEL" " ONCOLOG" " OPTICAL " " OSTEOMYELITIS " " OTITIS MEDIA " " PANCREATITIS " " PARALYTIC ILEUS " " PERITONEAL " " PNEUMOCONIOSIS " " PROSTATE " " PRURITUS " " SARCOIDOSIS " " PYELONEPHRITIS " " REFRACTIVE ERROR" " RENAL " " RETINA " " SARCOMA " " SUBCUTANEOUS DISEASE" " URINARY DISEASE" " URINARY TRACT INFECTION" " UROLITHIASIS " " URTICARIA " " VENTRICULAR " " VISION LOSS " " ACCOMODATION DISORDER" " SENSE ORGAN " " GUILLAIN BARRE SYNDROME" " IMPETIGO " " LOSE WEIGHT " " BIRTH DEFECT" "PAPILLOMAVIRUS" " GENE DEFECT" " PHYSICALLY DISAB" " TUMOUR" " BRAIN INJUR" " MAMMOGRA" " ANTITUMOR " " ANTITUMOUR " " BARIATRIC" " FATTY LIVER" " IMMUNOTHERAPY " " CHROMOSOMAL ABERRATION" " PERIODONTITIS " " OSTEOPOROSIS " " NEURON"
SWAps/ Health sector support		" SWAP" " TRAINING " " CAPACITY " " DATA SYSTEM" " SECTOR WIDE APPROACH" " HEALTH SYSTEM" " SECTOR PROGRAM" " BUDGET SUPPORT" " SECTOR SUPPORT " " HSS " " TRACKING PROGRESS " " SKILLED WORKERS " " HEALTH WORKERS " " SKILLED STAFF " " HEALTH PROFESSIONALS " " FACILITIES " " ESSENTIAL MEDICINES " " POLICY DEVELOPMENT" " MEDICAL EQUIPMENT" " SURGICAL EQUIPMENT" " HOSPITAL EQUIPMENT" " HOSPITAL EQMT " " HEALTH SECTOR PROGRAM" " HEALTH SECTOR SUPPORT " " SECTOR SUPPORT PROGRAM" " HEALTH INSTITUTIONAL STRENGTHENING " " HSPSP " " M&E " " MONITORING " " SURVEILLANCE " " GOVERNANCE " " HUMAN RESOURCE" " HUMAN CAPITAL " " IMPROVED CAPACITIES " " SCALING UP " " REALLOCATE RESOURCES " " STRATEGIES AND PROGRAM" " HIV STRATEG" " PROGRAM IN COUNTRY ACTIVITIES " " STRATEGIC INFORMATION " " PROCUREMENT " " EVIDENCE BASED " " CASE REPORTING " " OUTBREAK PREPAREDNESS " " RAPID RESPONSE STRATEG" " MEDICAL WORKER" " HEALTH CARE PERSONNEL " " OPERATIONAL RESEARCH " " SUPPORTIVE ENVIRONMENT " " INFORMATION SYSTEM" " INSECT " " WORKFORCE " " INFRASTRUCTUR" " ADMINISTRATIVE " " MEDICAL EDUCATION " " CASE NOTIFICATION " " CASE FINDING " " LABORATORY STRENGTHENING " "

Health focus area level	Program area	Keywords
		LABORATORY QUALITY " " LABORATORY NETWORK" " CONTROL SERVICES " " INFECTION CONTROL " " CONTROL PROGRAM" " SCALE UP" " STOP TB STRATEGY " " HEALTH EDUCATION " " CONTINUING EDUCATION " " SUPPLY " " HEALTH MANAGEMENT" " HEALTH POLICY " " MANAGEMENT AND COORDINATION " " ADMINISTRATIVE MANAGEMENT " " MANAGEMENT AND ADMINISTRATION " " STRENGTHENING NATIONAL HEALTH SYSTEM" " STRENGTHENING INSTITUTIONAL CAPACIT"
	Pandemic preparedness	" PANDEMIC PREPAREDNESS " " PANDEMIC RESPONSE" " PANDEMIC ALERT" " EPIDEMIC ALERT" " EPIDEMIC RESPONSE" " EPIDEMIC PREPAREDNESS " " OUTBREAK RESPONSE" " OUTBREAK ALERT" " OUTBREAK PREPAREDNESS " " PANDEMIC INFLUENZA " " EPIDEMIOLOGICAL INVESTIGATION" " CONTACT MANAGEMENT " " PREPAREDNESS AND RESPONSE PLAN" " PREPAREDNESS & RESPONSE PLAN" " BIOSAFETY MEASURE" "EARLY WARNING " " HEALTH SECURITY PREPAREDNESS " " HEALTH SECURITY RISK ASSESSMENT "
Other infectious diseases		" INFECTIOUS " " COMMUNICABLE " " TRICHURIASIS " " YELLOW FEVER " " WHIPWORM " " TRACHOMA " " SCHISTOSOMIASIS " " BILHARZIA " " SNAIL FEVER " " KAYAYAMA FEVER " " RABIES " " ONCHOCERCIASIS " " RIVER BLINDNESS " " ROBLES DISEASE" " LYMPHATIC FILARIASIS " " ELEPHANTIASIS " " LEISHMANIASIS " " LEISHMANIOSIS " " HOOKWORM " " FOOD BORNE TREMATOD" " FOODBORNE TREMATOD" " ECHINOCOCCOSIS " " HYDATID DISEASE" " HYDATIDOSIS " " DENGUE " " CYSTICERCOSIS " " CHAGAS " " TRYPANOSOMIASIS " " SLEEPING SICKNESS " " ASCARIASIS " " TROPICAL DISEASE" " AVIAN " " CHOLERA " " DYSENTERY " " PARASITE DISEASE" " AVIAN INFLUENZA " " AVIAN FLU " " FAO " " NEGLECTED TROPICAL DISEASE" "TYPHOID" " LEPROSY " " BURULI ULCER " " EBOLA" " EBOV " " EVD " " ZIKA " " ZIKV " " GUINEA WORM " " DRACUNCULIASIS " " FILARIASIS " " HEPATITIS E"

eTable 6. Additional health focus area categorizations

Channel	Allocation criteria	Health focus area
Bilaterals and the EC	CRS purpose code 13030, family planning	Family planning
	CRS purpose code 13020, reproductive health care	Maternal health, non-family planning
	CRS purpose code 12240, basic nutrition	Child and newborn nutrition
	CRS purpose code 12250, infectious disease control and the keywords “child” or “vaccine” present in descriptive variables	Child and newborn vaccines
	CRS purpose code 13040, STD control including HIV/AIDS	HIV/AIDS
	CRS purpose code 12262, malaria control	Malaria, unspecified

Channel	Allocation criteria	Health focus area
	CRS purpose code 12250, infectious disease control and no other keywords present in the descriptive variables	Other infectious diseases
	CRS purpose code 12263, tuberculosis control	Tuberculosis
	CRS purpose code 12230, basic health infrastructure	SWAPs/health system strengthening
	CRS purpose code 12281, health personnel development	SWAPs/health system strengthening
World Bank IDA and IBRD	Theme code population and reproductive health	Maternal, newborn, and child health, unspecified
	Theme code tuberculosis	Tuberculosis
	Theme code child health	Child and newborn health, unspecified
	Theme code HIV/AIDS	HIV/AIDS
	Theme code malaria	Malaria, unspecified
	Theme code injuries and non-communicable diseases	Non-communicable diseases, unspecified
	Theme code nutrition and food security	Child and newborn nutrition
	Theme code other communicable diseases	Other infectious diseases
	Theme code health system performance	SWAPs/health system strengthening
	Theme code social analysis and monitoring	SWAPs/health system strengthening
UNFPA	Family planning, population and development strategies, population and development, population dynamics	Family planning
	Reproductive health, maternal and newborn health, young people's SRH and sexuality education, HIV and STI prevention services, sexual and reproductive health, sexuality education	Maternal health
	Gender equality and women's empowerment, gender equality and reproductive rights, program coordination and assistance, adolescents and youth, civil society and rights for all, ending harmful practices, marginalized girls, protection rights, other	Family planning and Maternal health, unspecified, according to proportions between the two.
	HIV and STI prevention services, HIV and AIDS	HIV prevention
UNICEF	All DAH	HIV prevention
		Maternal, newborn, and child health (vaccines, maternal health, and health system strengthening)
UNAIDS	The keyword search was run on budget information for years 2008-2017<br>Program components in budget documents from 1998 to 2007	All program areas under HIV/AIDS and TB
UNITAID	Disease and type of project assigned in online database	HIV/AIDS (treatment, counseling and testing, prevention) , TB (treatment and diagnosis), Malaria (diagnosis and treatment), NCD other
GAVI	Vaccine DAH for HPV vaccine<br>All other vaccine DAH<br>HSS DAH	NCD other<br>Child and newborn vaccines<br>Maternal and child health HSS

Channel	Allocation criteria	Health focus area
GLOBAL FUND	Disease components for Malaria, HIV/AIDS, TB, TB/HIV, and Other (health systems strengthening)<br>Keyword search on program service delivery areas	All program areas under Malaria, TB, HIV and Swap/HSS
WHO	Reproductive, maternal, newborn, child, and adolescent health (divided by 2); Research in human reproduction	Maternal health, unspecified
	Nutrition	Child and newborn nutrition
	Vaccine-preventable diseases	Child and newborn vaccines
	Reproductive, maternal, newborn, child and adolescent health (divided by 2)	Child and newborn health, unspecified
	Aging and health; gender, equity and human rights mainstreaming	Maternal, newborn, and child health, unspecified
	HIV/AIDS	HIV/AIDS
	Malaria	Malaria
	Tuberculosis	Tuberculosis
	Mental health and substance abuse	Non-communicable diseases, mental health
	Disabilities and rehabilitation; Non-communicable diseases; Violence and injuries	Non-communicable diseases, unspecified
	Neglected tropical diseases; Tropical disease research; Infectious hazard management; Outbreak and crisis response (50%); Alert and response capacities (50%)	Other infectious diseases
	Health system information and evidence; Integrated people-centered health services; National health policies, strategies and plans; Access to medicines and health technologies and strengthening regulatory capacity; health emergency information and risk assessment (50%)	SWAps/health system strengthening
	Country health emergency preparedness and the International Health Regulations; health emergency information and risk assessment(50%); Emergency operations; Emergency core services; Outbreak and crisis response(50%); Epidemic- and pandemic-prone diseases; Alert and response capacities(50%)	SWAps/health system strengthening, pandemic preparedness
	Social determinants for health; Health and the environment; Food safety; Antimicrobial resistance	Other
PAHO	HIV/AIDS and STIs; HIV/AIDS, TB and malaria (33%)	HIV/AIDS, unspecified
	Tuberculosis; HIV/AIDS, TB and malaria (33%)	Tuberculosis, unspecified
	HIV/AIDS, TB and malaria (33%); Malaria and other Vector-Borne Diseases (50%);	Malaria, unspecified
	Communciable diseases; Malaria and other Vector-Borne Diseases (50%); Neglected Tropical and zoonotic diseases	Other infectious diseases
	Nutrition; Food Safety	Child and newborn nutrition
	Vaccíne-Preventable Diseases	Child and newborn vaccines

Channel	Allocation criteria	Health focus area
	Women, maternal, newborn, child, and adolescent and adult health	Maternal and child health, unspecified
	Mental Health and psychoactive substance use disorders	Non-communicable diseases, mental health
	Non Communicable Diseases and Risk Factors; Chronic noncommunicable diseases	Non-communicable diseases, unspecified
	Health systems leadership and governance; Human resources for health; Social protection and financing; Health systems information and evidence; Health services; People-centered integrated health services; Access to medical products and strengthening regulatory capacity; Health governance and financing, national health policies, strategies and plans	Swap/health system strengthening
	Violence and Injuries; Disabilities and Rehabilitation; Antimicrobial resistance; Aging and health; Gender, equity, human rights and ethnicity; Social determinants of health; Health and the environment; Strategic communications; Management and administration; Flexible and learning organization	Other

Disaggregating preliminary estimates by health focus area

Estimates by health focus area for years in which descriptive data were not available (usually 2017 and in many cases 2016 as well) were obtained by modeling channel-specific DAH per health focus area as a function of time. Out-of-sample validation was used to test the predictive accuracy of a large suite of models, estimating the models using 1990-2010 data and predicting 2011 and 2012. The potential models included fractional multinomial logit regression, OLS regression, autoregressive integrated moving average (ARIMA) models, epanechnikov kernel-weighted local polynomial smoothing, and multivariable fractional polynomial models. For each model, time was modeled linearly, with splines, and by including lag-dependent variables. Other methodologies considered included modeling health-focus-area-specific DAH as a dollar amount and as a fraction of the channel-specific total DAH. Lastly, models that involved transforming the dependent variable in natural log and logit transformed space were considered. In order to accommodate zero values in the logit transformation, the transformation described in Smithson and Verkuilen were applied.[71] Over 40 models and specifications were evaluated in total.

Each of the potential model and specification described above were estimated using data from 1990 through 2010, and then the estimated model was used to predict DAH by health focus area for 2011 and 2012. Since we have DAH estimates for 2011 and 2012, we compared the modeled estimates and the observed estimates and calculated average percent deviation and average total absolute deviation for each model and specification across all the channels and health focus areas. A variant of the Epanechnikov kernel-weighted local polynomial smoothing had the smallest average percent deviations and average total absolute error. In this model and specification, health focus area-specific DAH fractions were independently estimated at the channel level after they were logit transformed. Time was the only independent variable included in the model. The health focus area-specific DAH estimates were adjusted so the sum of the channel’s health focus area disbursements totaled channel-specific DAH envelope. Our preferred model, the Epanechnikov kernel-weighted local polynomial smoothing, minimized both the average percent deviation and the total absolute error out of sample, predicting two years ahead. See Dieleman et al. for a table that demonstrates the performance of four models, each with their optimal specification (as determined by the out-of-sample average percent deviation and total absolute error).[1]

Tracking development assistance for health from bilateral aid agencies and the European Commission

OECD-DAC maintains two databases on aid flows: 1) the DAC annual aggregates database, which provides summaries of the total volume of flows from different donor countries and institutions, and 2) the CRS, which contains project- or activity-level data.[3] This year, we used the DAC databases to track health ODA from 24 OECD-DAC members (Austria, Australia, Belgium, Canada, Denmark, Finland, France, Germany, Greece, Ireland, Italy, Japan, Luxembourg, the Netherlands, New Zealand, Norway, Portugal, South Korea, Spain, Sweden, Switzerland, the United Kingdom, the United States, and the EC), and one DAC Participant country, United Arab Emirates, for the years 1990 to 2017. Observed data for the DAC members was available from 1990 to 2016, and observed data for the United Arab Emirates was available from 2009 to 2016. United Arab Emirates bilateral health ODA from 1990 to 2008 was obtained through personal correspondence.

These two DAC databases track the following types of resource flows:

Official development assistance (ODA), defined as "flows of official financing administered with the promotion of the economic development and welfare of developing countries as the main objective"69 is tracked from its 30 members (Austria, Australia, Belgium, Canada, Czech Republic, Denmark, Finland, France, Germany, Greece, Hungary, Iceland, Ireland, Italy, Japan, Luxembourg, the Netherlands, New Zealand, Norway, Poland, Portugal, Slovakia, Slovenia, South Korea, Spain, Sweden, Switzerland, the United Kingdom, the United States, and the EC). The CRS also now includes some private ODA, such as that funded by BMGF and the Global Fund to Fight AIDS, Tuberculosis and Malaria (GFATM), as well as assistance from a number of non-DAC countries such as the United Arab Emirates and Kuwait.

ODA includes:

• Bilateral ODA, which is given directly by DAC members as aid to recipient governments, core contributions to NGOs and public-private partnerships, and earmarked funding to international organizations.

• Multilateral ODA, which includes core contributions to multilateral agencies such as WHO, UNFPA, GFATM, Gavi, UNAIDS, UNICEF, PAHO, the World Bank, and other regional development banks. Only regular budgetary contributions to these institutions can be reported to the OECD-DAC; hence, extrabudgetary funds, including earmarked contributions that donors can report as bilateral ODA, are not included as multilateral ODA. Only 70% of core contributions to WHO can be counted as multilateral ODA.

a. Official development finance (ODF), which includes grants and loans made by multilateral agencies.

b. Other official flows (OOF), which refers to transactions that "do not meet the conditions for eligibility as Official Development Assistance or Official Aid, either because they are not primarily aimed at development, or because they have a Grant Element of less than 25 percent."

The DAC aggregate tables include all multilateral development banks, GFATM, operational activities of UN agencies and funds, and a few other multilateral agencies. The project-level data in the CRS cover a smaller subset of multilateral institutions, including UNAIDS, UNFPA, UNICEF, public-private partnerships including Gavi and GFATM, some development banks, and BMGF, but do not reflect the core-funded operational activities of WHO prior to 2009, disbursements by Gavi prior to 2007 and BMGF prior to 2009, or all loans from the World Bank.

This research utilized the CRS as the principal source for tracking bilateral DAH. This is because the DAC aggregate tables do not report detailed project-level information about the recipient country and health focus area. The OECD sector codes for general health (121), basic health (122), and population programs (130) were used to identify health flows in the CRS. Only ODA related flows are used in our analysis, including OECD flow codes corresponding to ODA grants (11), ODA grant-like (12), ODA loans (13), and equity investment (19).

To avoid double-counting, all identifiable earmarked commitments and disbursements made by DAC members via Gavi, International Finance Facility for Immunisation (IFFIm), GFATM, WHO, UNICEF, UNAIDS, UNFPA,

, International drug purchase facility, PAHO, World Bank, and regional development banks. The channel of delivery fields as well as keyword searches in the descriptive project fields (project title, short description, and long description) were used to identify potential sources of double-counting. Channel codes in the CRS data were used to track DAH to international and donor-country-based non-governmental organizations. The names of NGOs that were captured in IHME's NGO data (as detailed in the section titled "Tracking non-governmental organizations") were searched for in the CRS descriptive variables and tagged as double-counting. Research funds for HIV/AIDS channeled by the US government through the National Institutes for Health (NIH) were also removed from the total since they do not meet the definition of DAH as contributions from institutions whose primary purpose is development assistance. Official development finance (ODF) from the CRS was not counted because these expenditures were included elsewhere, either in the analysis of multilateral institutions relevant to the study or in the assessment of health spending by BMGF, the data for which were obtained via correspondence and from their annual reports, audited financial statements, and project databases. To avoid double-counting, only health assistance flows from multilateral institutions to low- and middle-income countries were counted, and not transfers to multilateral institutions. Also, for regional projects the disbursements are split amongst all countries in the specified OECD region. For example, a project allocated to recipient "North of Sahara, regional" would have its disbursements split equally between all the countries in the corresponding OECD region: Algeria, Egypt, Libya, Morocco, and Tunisia.

Allocation of funding to health focus areas was assigned as described in the section "DISAGGREGATING BY HEALTH FOCUS AREA", based on a keyword search of five descriptive variables in the CRS: project title, short description, long description, channel name, and channel reported name. Additional adjustments were made based on CRS purpose codes, as detailed in eTable 6, in order to ensure that the specified purpose corresponded to the highest-weighted health focus area.

Estimating disbursements for the 24 bilateral channels and the EC

Both the DAC tables and the CRS rely on information reported by DAC members and other institutions to the OECD-DAC. Hence, the quality of the data varies considerably over time and across donors. Three variables were used to estimate yearly donor disbursements: CRS commitments, CRS disbursements, and DAC commitments. There were two main challenges in using the data from the CRS for this research:

1. underreporting of aid activity to the CRS compared to what is reported to the DAC, and

2. underreporting of disbursement data to the CRS compared to commitment data reported to the CRS.

These issues are highlighted in eFigure 1. Methods developed to account for both these challenges are discussed below. Details on how we estimated the cost of providing technical assistance and program support for these institutions are highlighted below in the section titled calculating the technical assistance and program support component of development assistance for health from loan-and grant-making channels of assistance.

To address these two challenges, we determined a cutoff point for each channel. We defined this channel-specific cutoff year as when the ratio of total CRS disbursements to commitments was greater than 50% and did not drop subsequently below 30%. eFigure 2 below shows each donor's CRS disbursement to commitment ratio in green, and the estimated cutoff year is marked with a vertical red line. For years after the cutoff year, DAH is measured using the unadjusted disbursement data. For the time prior to the cutoff year, it was determined that the disbursement data are not of high enough quality, and adjusted commitments were used instead.

Two adjustments were made to commitments to estimate disbursements before each donor-specific cutoff point:

I. The first adjustment addressed underreporting of aid activity to the CRS (relative to the DAC). To address this challenge, all CRS commitments for the health sector were adjusted upward using the DAC commitment to CRS commitment coverage ratio. The coverage ratio of the CRS was well below 10% before 1996 but has improved steadily over time.

II. The second adjustment addressed underreporting of disbursements data to the CRS (relative to commitments reported to the CRS). To address this challenge, we pooled completed projects in the CRS

that have disbursement data for each channel and computed yearly project disbursement rates (the fraction of total commitments disbursed for each year of a project) and overall project disbursement rates (the fraction of total commitments disbursed over the life of each project) by project length. Yearly disbursement schedules were calculated for projects with lengths of one, two, three, four, five, and six years. When an observed project length was more than six years, all expenditure after the sixth year was aggregated and assumed to be expended in the sixth year. This does not happen often. Yearly disbursement rates were the median of these shares, averaged across projects for every donor in each project year. The sum of these averages equals one, so that all the disbursements were expended over the lifetime of a project. The product of these donor-specific yearly disbursement rates and the donor-specific overall disbursement rates produced the donor-specific disbursement schedules. The donor-specific disbursement schedules were applied to project-level DAC-adjusted commitments reported in the CRS. eFigure 3 shows the yearly disbursement rates and overall disbursement rates for projects with one- to six-year lifespans for each of the 24 member countries and the EC.

Lastly, to address the challenge of underreporting of aid activity to the CRS compared to the DAC for all years, the difference between each donor's aggregate DAC health commitments and CRS health disbursements was added to each donor's yearly DAH. Since only aggregate commitments are reported to the DAC, several adjustments were made, based on more detailed CRS data:

I. First, each donor's yearly average project length was calculated by applying the donor-specific disbursement schedules described above to CRS projects that had disbursement in order to get adjusted DAC commitments.
II. Commitments for projects that have not opened yet were then subtracted, based on the open date reporting in the CRS. This ensured that future disbursements were not captured.
III. Lastly, these DAC-adjusted commitments were compared to CRS disbursements, inclusive of transfers that were later dropped as double-counting.

In addition to tracking disbursements from the EC, gross disbursements from the DAC were used to compile data on the sources of funding for the EC.

eFigure 1 Comparing CRS commitments, CRS disbursements, and DAC commitments

This figure compares commitments and disbursements from the Creditor Reporting System (CRS) and Development Assistance Committee (DAC) databases of the Development Assistance Committee of the Organisation for Economic Co-operation and Development (OECD-DAC) from 1990 to 2016. CRS disbursements are usually underreported when compared to both CRS and DAC commitments data, especially in earlier years. Because of this gap between CRS and DAC, CRS disbursements data were adjusted to fit DAC commitments data.

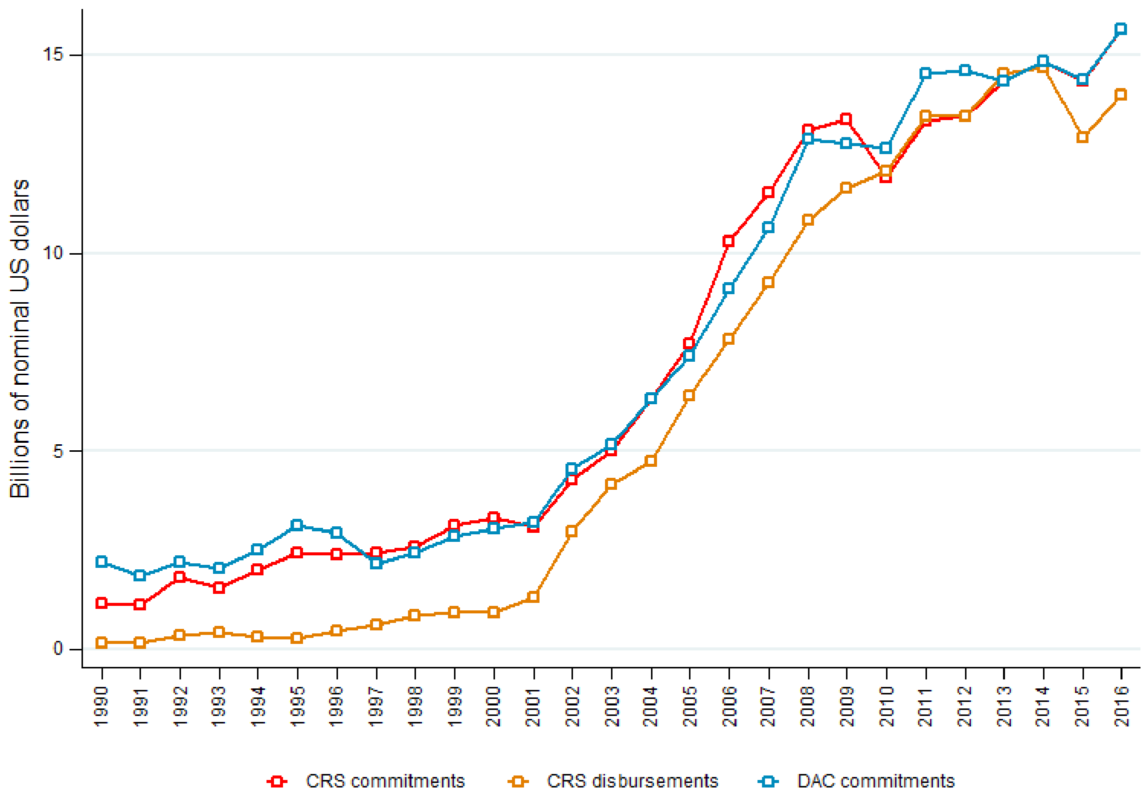

Source: OECD-DAC and OECD Creditor Reporting System

eFigure 2 CRS disbursement to commitment ratio and cutoff points by donor agency

This figure shows the channel-specific cutoff year. Before this year, we adjust CRS commitments using disbursement schedules. After this cutoff we rely on CRS-reported disbursements. The total CRS disbursements to commitments ratio is in green, and the cutoff year is marked with a vertical red line. The cutoff year is determined to be when the ratio goes above 50% and does not fall back below 30%. The vertical axis represents the CRS disbursement to commitment ratio as a percentage. ARE = United Arab Emirates, AUS = Australia, AUT = Austria, BEL = Belgium, CAN = Canada, CHE = Switzerland, DEU = Germany, DNK = Denmark, EC = European Commission, ESP = Spain, FIN = Finland, FRA = France, GBR = Great Britain, GRC = Greece, IRL = Ireland, ITA = Italy, JPN = Japan, KOR = South Korea, LUX = Luxembourg, NLD = the Netherlands, NOR = Norway, NZL = New Zealand, PRT = Portugal, SWE = Sweden, USA = United States of America

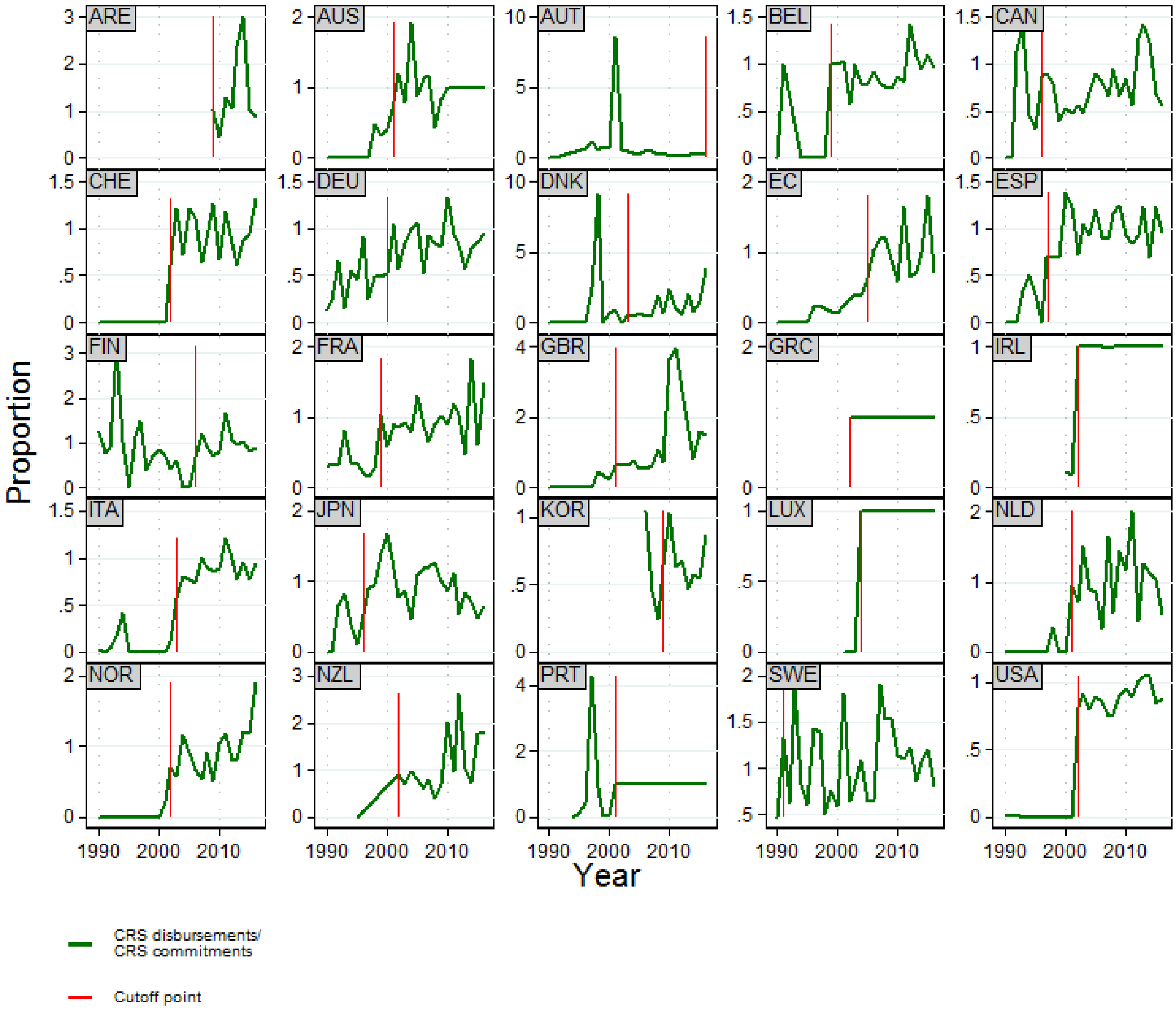

Source: OECD Creditor Reporting System

**Box 3. EXAMPLE. Australia's CRS disbursement to commitment ratio and cutoff year**
The green line shows the ratio of Australia's disbursements to commitments, as reported in the CRS. Prior to 2001, the ratio was always below 50%. In 2001, the ratio rose above 50%; it did not fall below 30% in subsequent years, thereby defining 2001 as the cutoff year. Thus, for Australia, before 2001 DAH is based on adjusted CRS commitment data. These data are adjusted using disbursements schedules (eFigure 3) and data from the DAC. After 2001, Australia's DAH is based on the disbursements reported in the CRS.

eFigure 3 One- to six-year disbursement schedules for bilateral channels

This figure shows the estimated disbursement schedules for bilateral channels. Before the channel-specific cutoff year, we rely on commitment data to inform our estimates of DAH. Commitment data are adjusted to reflect disbursements over time using schedules estimated from projects in the CRS that have both commitment and disbursement data. The vertical axis represents the percentage of the commitment disbursed. ARE = United Arab

Emirates, AUS = Australia, AUT = Austria, BEL = Belgium, CAN = Canada, CHE = Switzerland, DEU = Germany, DNK = Denmark, EC = European Commission, ESP = Spain, FIN = Finland, FRA = France, GBR = Great Britain, GRC = Greece, IRL = Ireland, ITA = Italy, JPN = Japan, KOR = South Korea, LUX = Luxembourg, NLD = the Netherlands, NOR = Norway, NZL = New Zealand, PRT = Portugal, SWE = Sweden, USA = United States of America

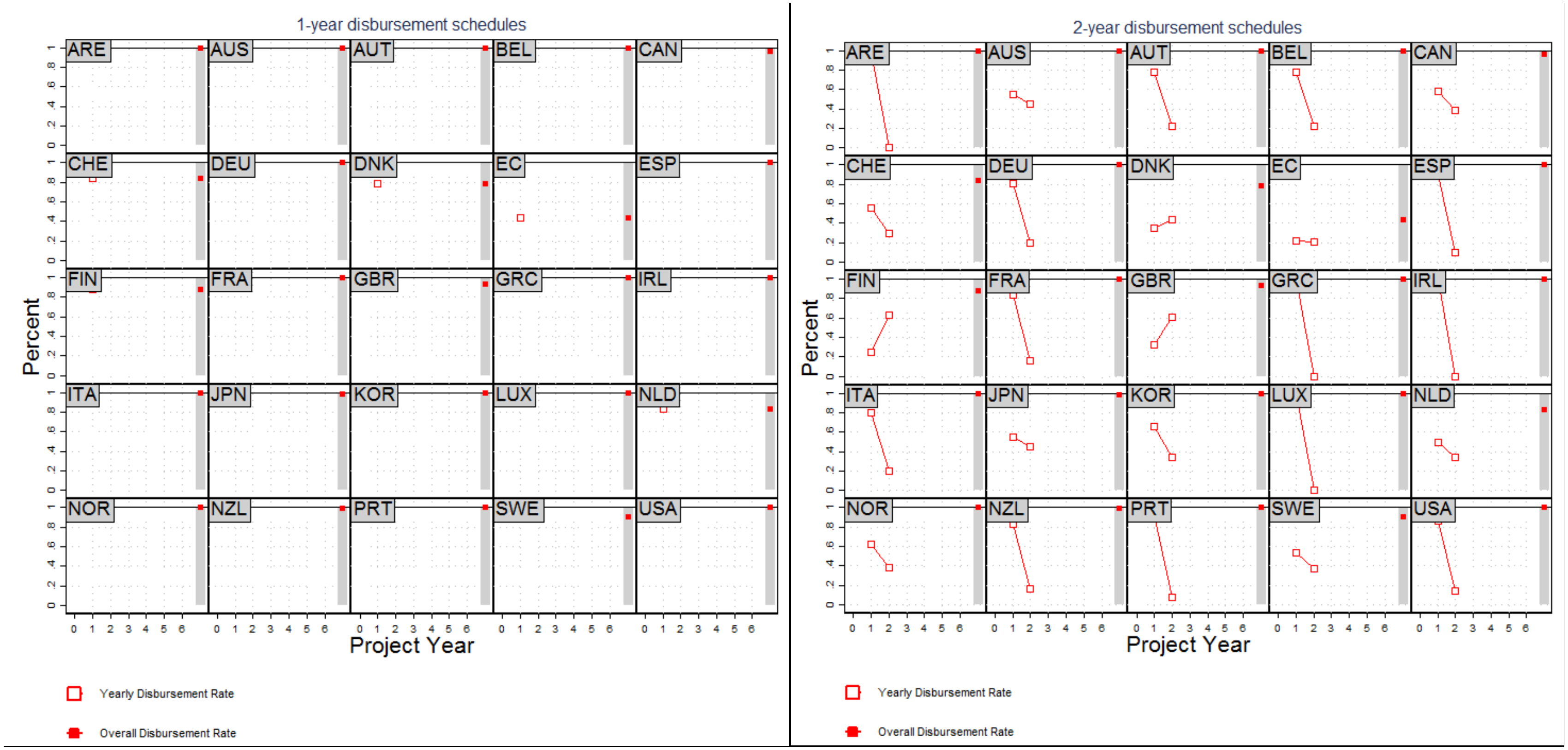
1-year disbursement schedules
2-year disbursement schedules
ARE
AUS
AUT
BEL
CAN
CHE
DEU
DNK
EC
ESP
FIN
FRA
GBR
GRC
IRL
ITA
JPN
KOR
LUX
NLD
NOR
NZL
PRT
SWE
USA
Percent
Project Year
Yearly Disbursement Rate
Overall Disbursement Rate

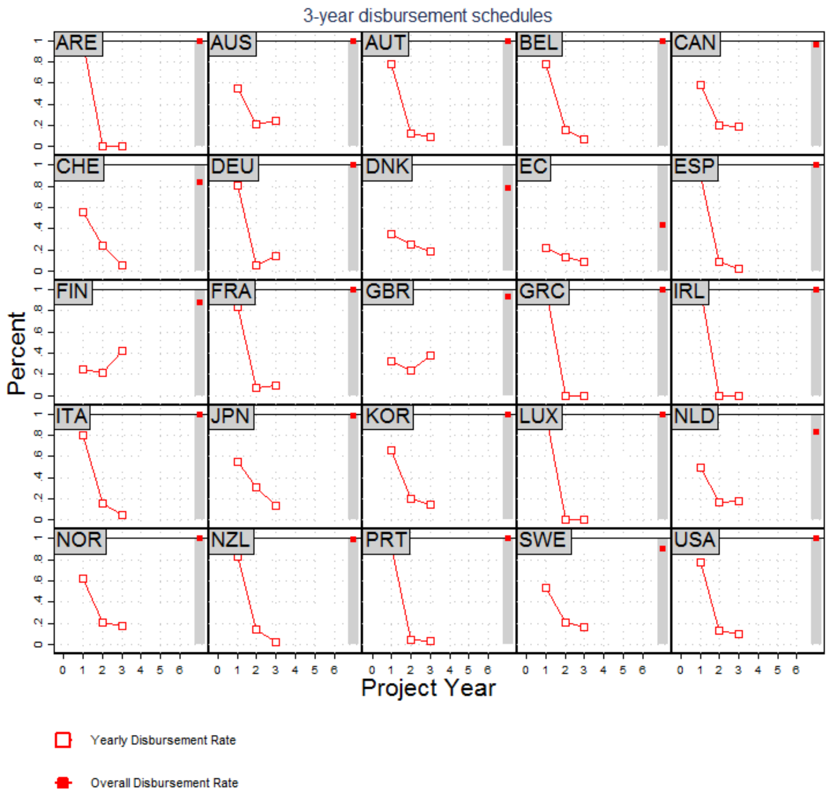
3-year disbursement schedules
ARE
AUS
AUT
BEL
CAN
CHE
DEU
DNK
EC
ESP
FIN
FRA
GBR
GRC
IRL
ITA
JPN
KOR
LUX
NLD
NOR
NZL
PRT
SWE
USA
Percent
Project Year
Yearly Disbursement Rate
Overall Disbursement Rate

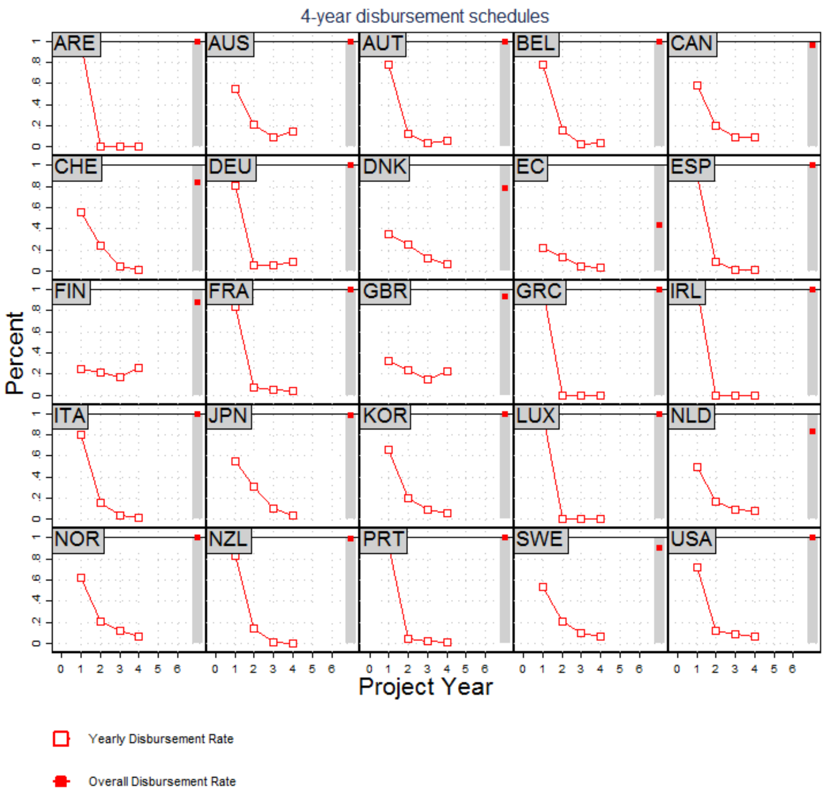
4-year disbursement schedules
ARE
AUS
AUT
BEL
CAN
CHE
DEU
DNK
EC
ESP
FIN
FRA
GBR
GRC
IRL
ITA
JPN
KOR
LUX
NLD
NOR
NZL
PRT
SWE
USA
Percent
Project Year
Yearly Disbursement Rate
Overall Disbursement Rate

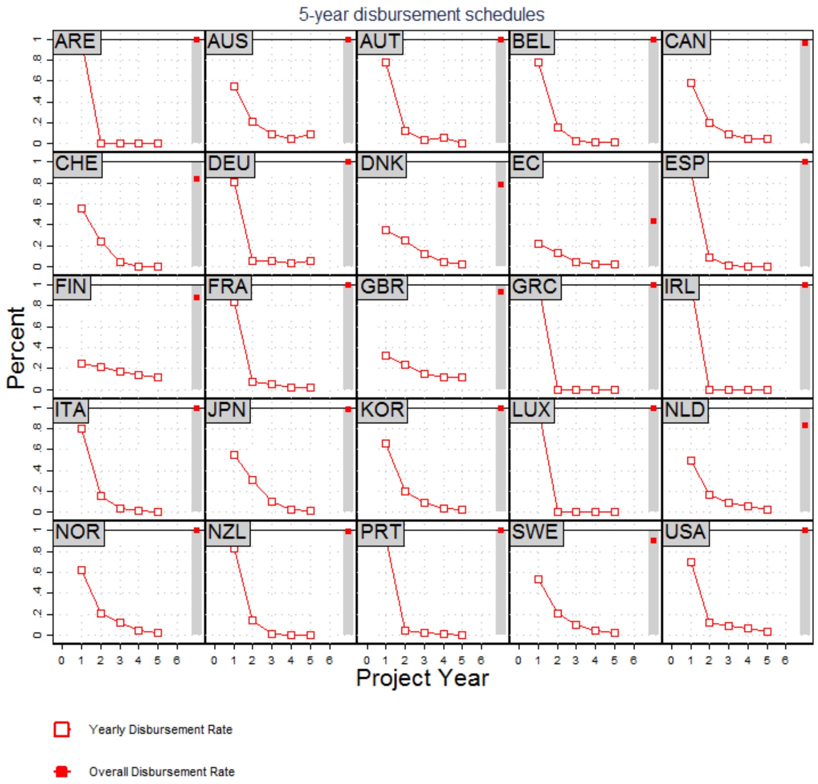
5-year disbursement schedules
ARE
AUS
AUT
BEL
CAN
CHE
DEU
DNK
EC
ESP
FIN
FRA
GBR
GRC
IRL
ITA
JPN
KOR
LUX
NLD
NOR
NZL
PRT
SWE
USA
Percent
Project Year
Yearly Disbursement Rate
Overall Disbursement Rate

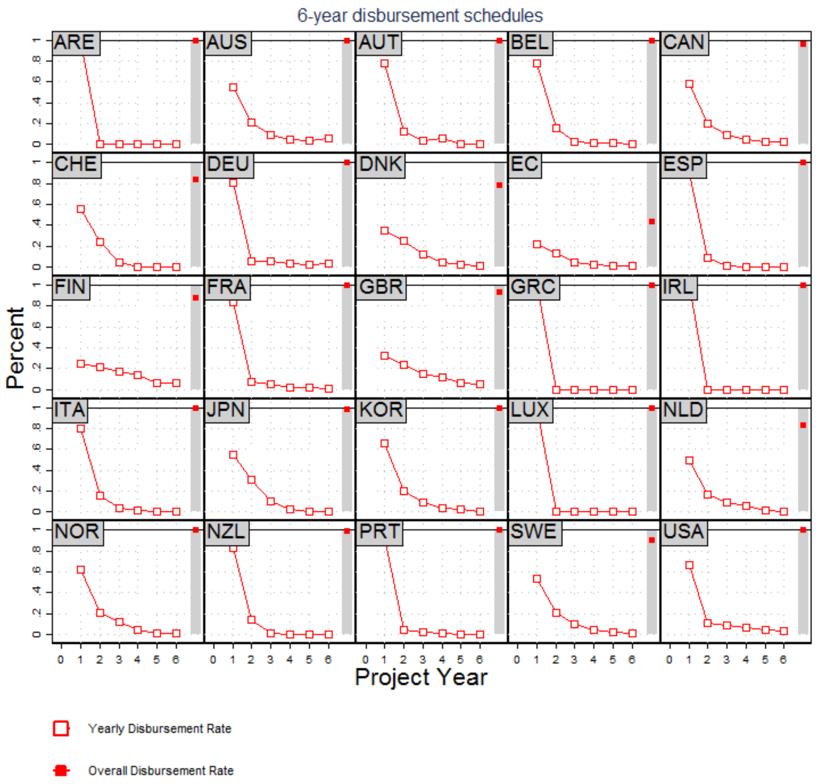
6-year disbursement schedules
ARE
AUS
AUT
BEL
CAN
CHE
DEU
DNK
EC
ESP
FIN
FRA
GBR
GRC
IRL
ITA
JPN
KOR
LUX
NLD
NOR
NZL
PRT
SWE
USA
Percent
Project Year
Yearly Disbursement Rate
Overall Disbursement Rate

**Box 4. EXAMPLE. Australia's one- to six-year disbursement schedules**
To estimate disbursements using commitment data, we rely on disbursement schedules derived from CRS data that include both commitments and disbursements. Disbursement schedules are specific for each channel and the length of a project. These schedules also take into consideration the average amount of commitments for each channel that lead to disbursements. Across all Australian projects in the CRS with complete disbursements data, Australia disbursed 100% of the funds that it committed, as shown by the solid red dot on the right-hand side of the Australia panel (upper left corner of the first panel of eFigure 3). In projects with a length of one year, Australia disbursed 100% of the funds that it committed in that year. For two-year projects, Australia disbursed 59% of total disbursements in year one and 41% of total disbursements in year two. In projects with lengths of three years, Australia disbursed about 59% of total disbursements in year one and 19% and 22% of total disbursements in years two and three, respectively. This is estimated for projects ranging from one to six years. The disbursement schedules were applied to commitment data from the CRS to estimate disbursements for years prior to the cutoff year, which is 2001 for Australia.

To predict DAH for the recent years not reported in the CRS, budget data were extracted from a variety of sources. These data are listed in eTable 4. Global health budgetary data were utilized whenever possible, but these detailed data were available as a complete time series only for Australia and the United States. For all other bilateral channels, general ODA budgets were used. In order to predict DAH for 2017 for 24 bilateral agencies, the budget ratio for each donor was calculated by dividing DAH estimates by the corresponding budget data (ODA or global health). Budget ratios for 2017 were projected using a weighted average of the previous three years (placing one-half weight on the one-year lagged ratio, one-third weight on the two-year lagged ratio, and one-sixth weight on the three-year lagged ratio), and this ratio was multiplied by the observed budgeted DAH for those same years. eFigure 4 plots the budget ratio for each bilateral channel. Budget data for the EC were inconsistent and did not match the disbursement series. Instead, DAH for 2017 was estimated based on trends in DAH for EC member countries. A weighted average was applied to the percent change in DAH from 2016-2017 for all EC member countries. The weighting was based on each country's total national contributions to the EC. These data were collected from the EC's 2016 financial statement.[72] The weighted average was then applied to the EC's 2016 DAH to forecast 2017.**eFigure 4 DAH as a percentage of corresponding budget data by bilateral agency**

This figure shows the trend of the ratio of DAH measured as a share of budget data. Green dots indicate that a donor provided global-health-specific budget data, so in these cases the denominator is all global-health-specific budgeted data. The numerator is estimated DAH. Red dots indicate that a donor did not have global-health-specific budget data, so overall ODA budget data were used in calculating the DAH to budget ratios. The vertical axis represents estimated DAH as a fraction of corresponding budget data.. ARE = United Arab Emirates, AUS = Australia, AUT = Austria, BEL = Belgium, CAN = Canada, CHE = Switzerland, DEU = Germany, DNK = Denmark, ESP = Spain, FIN = Finland, FRA = France, GBR = Great Britain, GRC = Greece, IRL = Ireland, ITA = Italy, JPN = Japan, KOR = South Korea, LUX = Luxembourg, NLD = the Netherlands, NOR = Norway, NZL = New Zealand, PRT = Portugal, SWE = Sweden, USA = United States of America

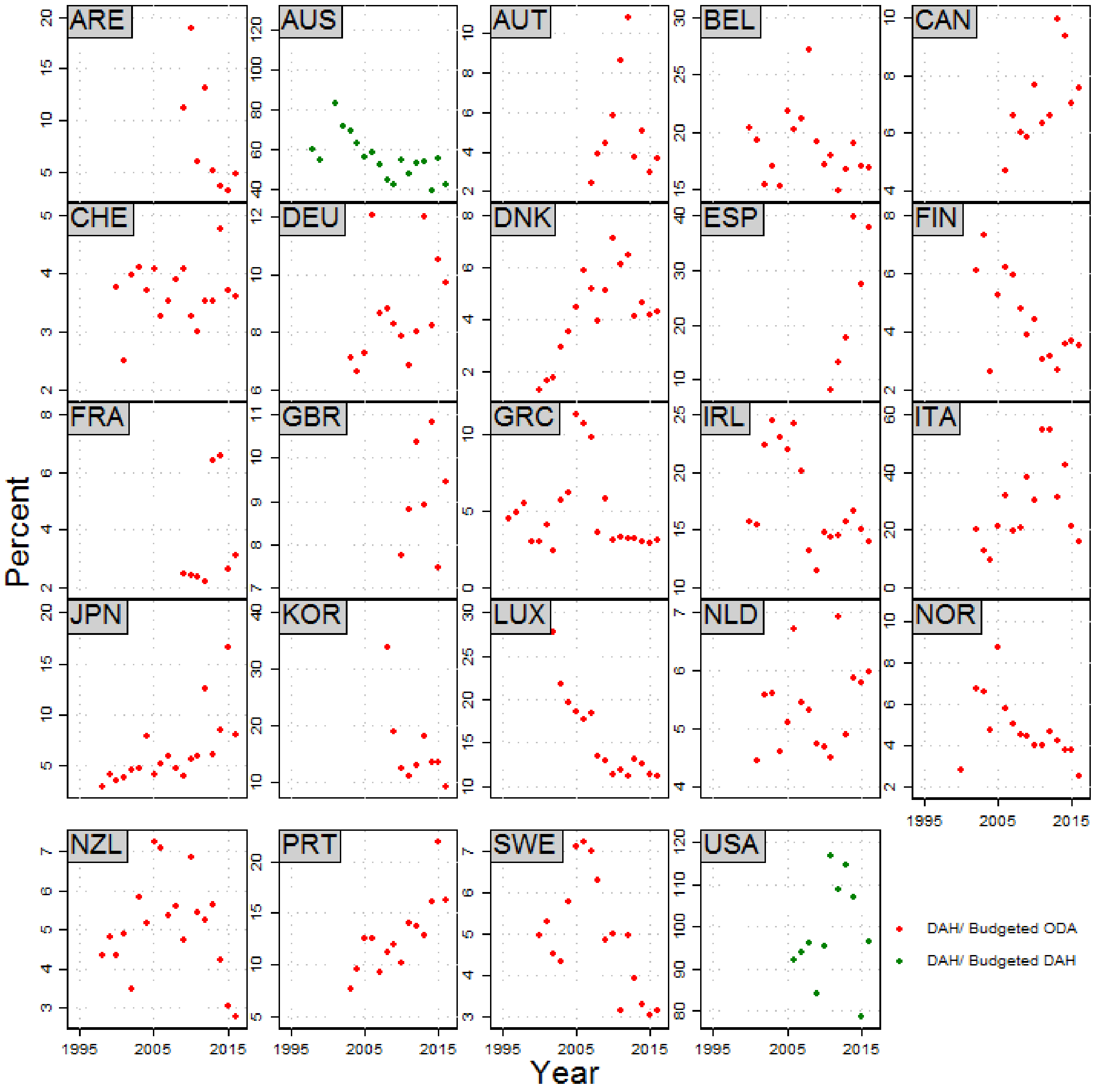

Source: IHME DAH Database (2017) and corresponding bilateral ODA/DAH budget documents outlined in eTable 2 and 4.

Box 5. EXAMPLE. Australia's DAH as a percentage of corresponding budget data

Australia provided global-health-specific budget data for 1998-2017 through its International Development Assistance and Overseas Aid Program budgets. For 1998-2016, health ODA and observed DAH were used to create DAH to budget ratios. These budget ratios were then applied to 2017 health ODA budget data to project DAH in 2017, using a weighted average:

$$(Total\ DAH_t) = \left(\frac{1}{2}\right)(\ Budget\ ratio_{t-1})\ (Budgeted\ GHE_t)\ + \left(\frac{1}{3}\right)(Budget\ ratio_{t-2})(Budgeted\ GHE_t) + \left(\frac{1}{6}\right)(Budget\ ratio_{t-3})(Budgeted\ GHE_t)$$

where t = year to be modeled (2017).

To supplement our estimates of development assistance for health to HIV/AIDS and malaria program areas for the United States, we used additional available data from the President's Emergency Plan for AIDS Relief (PEPFAR) and the President's Malaria Initiative (PMI). We downloaded data on all planned funding by PEPFAR by recipient country, year, and program area from 2004 to 2017.[73] All PEPFAR projects were assigned to our eight HIV/AIDS program areas using PEPFAR budget codes, splitting out overhead costs equally to all other program areas. We then created country-year specific HIV/AIDS program area fractions out of total annual HIV/AIDS DAH, which we applied to all United States HIV/AIDS projects in the CRS from 2004 to 2016 by country-year. To inform malaria funding by program areas, we downloaded the most recently available malaria funding tables from malaria operational plans for all countries and years.[74] We assigned each line item in these tables to our eight malaria program areas, and then created fractions for the malaria program areas out of the total annual malaria DAH specific to each country-year. These fractions were applied to all United States malaria projects in the CRS from 2006 to 2016 by country-year.

eFigure 5 Malaria DAH to program areas as assigned by keyword search and PMI reports

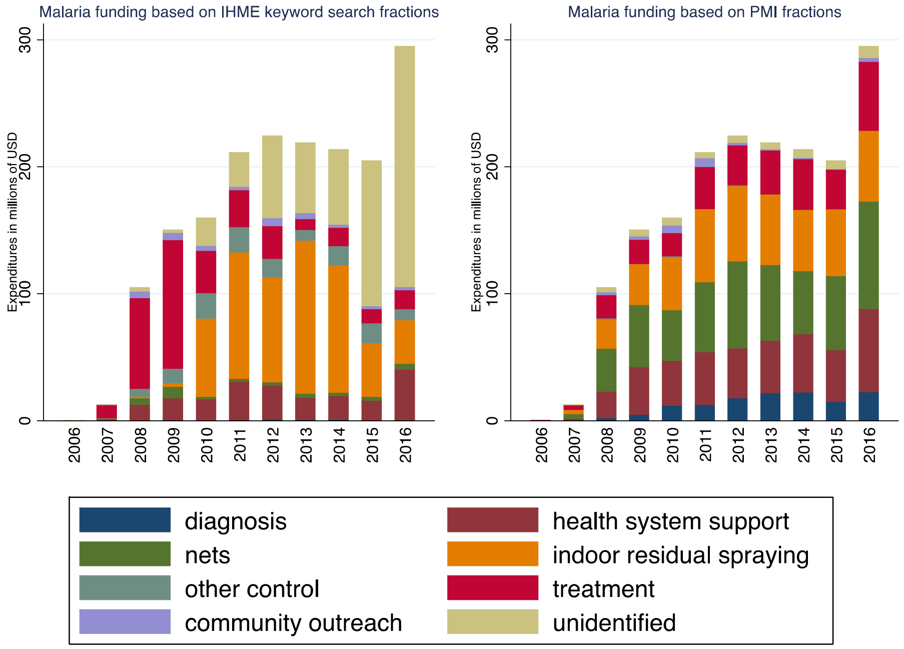

Source: IHME DAH Database (2017) and PMI malaria operational plans

This figure outlines the assignment of funding to malaria program areas for United States projects from the OECD CRS from 2006 onwards. The figure on the left shows how malaria funding is broken out based on keyword search. The figure on the right shows the breakdown of funding to malaria program areas based on PMI malaria funding tables. Using the data from PMI reduces the amount of unallocable funding. As such, in as often as more disaggregated information on project allocation is available, IHME uses such project information available in project budget documents or other project documents to disaggregate into program areas.

Tracking development assistance for health from the development banks

The World Bank project-level health disbursement data for 1990 through 2017 were obtained through correspondence with Miyuki Parris, Operations Analyst.[75] The World Bank recently underwent a recoding process for their disbursements. This recoding affected health disbursements, however the recoding was not completed for projects with disbursements prior to 2001. To create a comparable dataset adjustments had to be made. Regression analysis to predict health disbursements were explored, however, in the end the average percent change between project-level health disbursements before and after recoding was used to adjust health disbursements prior to 2001. It was observed that on average, between 2001 and 2005 (inclusive) the recoding process decreased health disbursements by 0.22%. This number was used to adjust all project-level health disbursements prior to 2001.[76] Health disbursements included all health projects as well as other sector projects with a health sector code. In addition, data were collected from the World Bank online loans database in order to fill in descriptive information for loans from the two arms of the World Bank: the International Development Association (IDA) and the International Bank for Reconstruction and Development (IBRD).[75] Along with keyword searches, health theme codes were used to allocate disbursements by health focus area. The online database contains up to five sector codes and five theme codes that can be assigned to each project. Sector codes represent economic, political, and social subdivisions, while theme codes represent the goals or objectives of World Bank activities. The codes are summarized in eTable 7. Emergency recovery loans were excluded since they do not fit the definition of DAH.

eTable 7 World Bank's health sector and theme codes

**Health sector codes**<br>Sector codes represent economic, political, or social subdivisions within society. World Bank projects are classified by up to five sectors.	**Health theme codes**<br>Theme codes represent the goals or objectives of World Bank activities.
Historic (prior to 2001):<br>(1) Basic health<br>(2) Other population health and nutrition<br>(3) Targeted health<br>(4) Primary health, including reproductive health, child health, and health promotion<br><br>Current (as of 2001):<br>(1) Health<br>(2) Compulsory health finance<br>(3) Public administration – health<br>(4) Noncompulsory health finance	Current:<br>(1) HIV/AIDS<br>(2) Malaria<br>(3) Tuberculosis<br>(4) Other communicable diseases<br>(5) Population and reproductive health<br>(6) Child health<br>(7) Nutrition and food security<br>(8) Injuries and non-communicable diseases<br>(9) Health system performance<br>(10) Social analysis and monitoring

Data on yearly government contributions were obtained from the DAC statistics in order to disaggregate IDA flows by source. Details on how we estimated the cost of providing technical assistance and program support for these institutions are highlighted below in the section titled "*Calculating the technical assistance and program support component of development assistance for health from loan-and grant-making channels of assistance*". The data received from the World Bank captured disbursements for only the first few months of 2017, so lending amounts by sectors, referred to below as budget data from 2013 through 2017 and historic disbursement data were used to predict 2017 health disbursements for IDA and IBRD separately.[77,78] Budget data is reported for fiscal years, while health disbursements were reported during the calendar year. To remedy this misalignment, budget data was averaged over two years to reflect half of the fiscal years falling within the calendar years. The 2017 estimate was based on a three-year weighted average of previous years (placing one-half weight on the one-year lagged ratio, one-third weight on the two-year lagged ratio, and one-sixth weight on the three-year lagged ratio). The predicted ratio was then multiplied by the observed program budget for 2017 to get the estimates of DAH.

$$(Predicted\ Ratio) = \left(\frac{1}{2}\right)(\ Observed\ DAH_{t-1})\ (Budgeted\ DAH_{t-1})\ + \left(\frac{1}{3}\right)(Observed\ DAH_{t-2})(Budgeted\ DAH_{t-2}) + \left(\frac{1}{6}\right)(Observed\ DAH_{t-3})(Budgeted\ DAH_{t-3})$$

$$(Total\ DAH_t) = (Predicted\ Ratio)(Budgeted\ HE_t)$$

eFigure 6 shows (a) total health budgets, referred to as lending amount from the World Bank website (green dashed line), (b) total health disbursements received from correspondence (orange line), and (c) predicted full-year disbursements (black dashed line). The database distinguishes between loans from IDA and IBRD, but the aggregates are shown in the figure.

eFigure 6 World Bank's annual health sector commitments and disbursements

This figure shows health sector commitments from the online database in green. The orange line shows annual health disbursements data received from the World Bank through 2017. The line for 2017 disbursements is lower because the 2017 data are incomplete due to reporting lag. The dashed black line shows predicted full-year disbursements based on the estimation method described above.

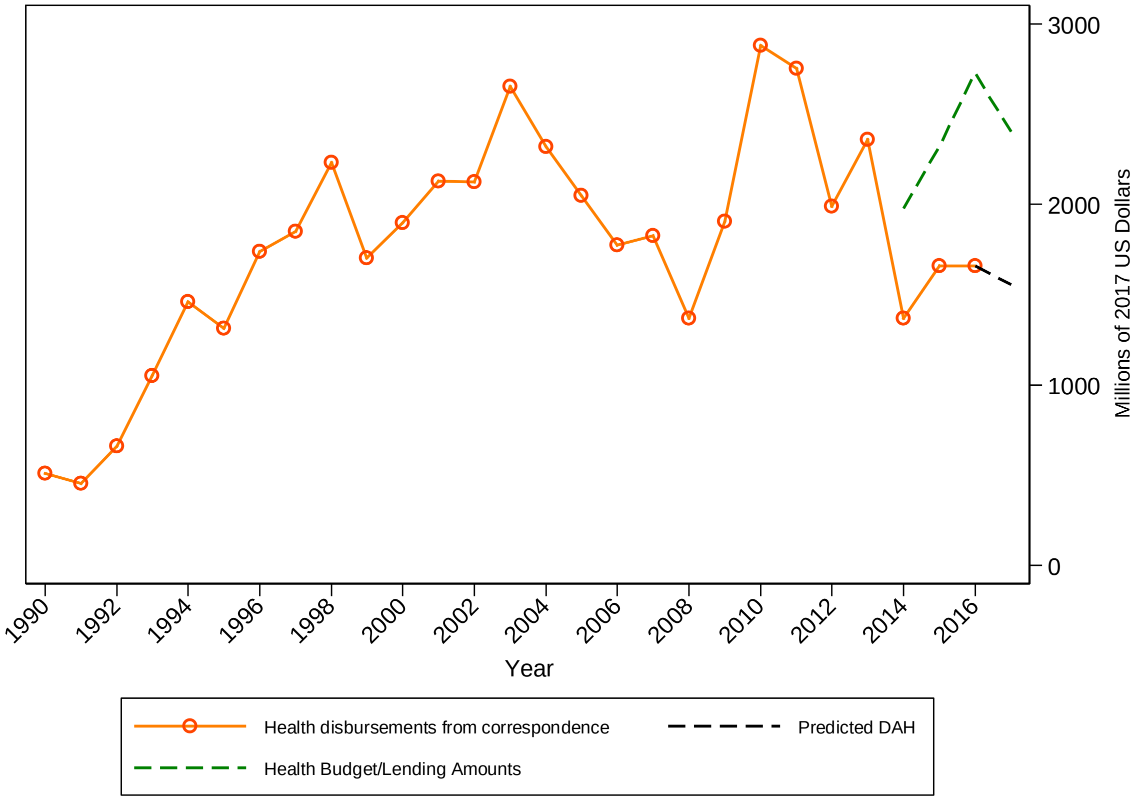

Source: IHME DAH Database (2017),World Bank website, and correspondence with World Bank

Regional development banks

The Asian Development Bank (ADB), and Inter-American Development Bank (IDB) all maintain their own loan databases, which were used to estimate disbursements.[14,16,17] To estimate health disbursements from the African Development Bank (AfDB), data was received via correspondence with Ms. Josselyne Ahogny (Manager, Loan accounting division)[33] eTable 8 provides a summary of the data sources used across the regional banks. Furthermore, eFigure 6 displays the disbursements for AfDB from 1990 to 2017 and eFigures 7, and 8 display commitments and disbursements from 1990 to 2017 for AsDB and IDB.

For AfDB, we received project level disbursement data from 2001 through October 2017. The final estimate for 2017 was rescaled based on the 10 months of complete data received for 2017. For pre-2001 estimates, data from the Compendium of Statistics were used for estimates pre-2001.

The ADB reported commitments and disbursements for all projects. Many of these projects were tagged as belonging to multiple sectors. For example, a project can be tagged for health, for education, and for public sector management. For projects with multiple sectors, disbursements and commitments were divided by the number of sectors a project was tagged for. If a project had multiple sectors, but it did not have the word "health" in its title or in its description, and if it also did not include any words associated with the health focus areas tracked in the *Financing Global Health* report in its title or in its description, it was excluded from the study. Once disbursements and commitments were adjusted for the presence of multiple sectors, annual disbursements were estimated by dividing the project length by total disbursements. For projects without a closing date, estimates were based on the average project length by project type (loan, grant, technical assistance). When no disbursement data were available, adjusted commitments were used, based on the average fraction of commitments that were disbursed by project type for projects with both commitments and disbursements data.

The IDB's project database also provided commitments and disbursements for all projects. The same methods were used for estimating annual disbursements from the IDB as were used for the ADB. Through correspondence, 2017 health loan disbursements were obtained. These numbers were used in the 2017 estimates. The dataset used to estimate disbursements for ADB was updated in October 2017 and the dataset used to estimate disbursement for IDB was updated in September 2017. Due to lags in reporting, preliminary estimates of DAH in 2017 may be incomplete. However, since these channels have so few new projects each year, it was assumed that smoothing disbursements over time for reported projects captured the majority of total disbursements for 2017.

eTable 8 Summary of data sources for the regional development banks

This figure indicates the data available and used to estimate DAH. (X) indicates that project-level data are present in the dataset. (-) indicates that project-level data are not present in the dataset.

Institution	Data source	Commitments	Cumulative disbursements	Yearly disbursement	Notes
**African Development Bank (AfDB)**	Compendium of Statistics	X		(Aggregate - not at the project level)	The Compendium of Statistics was not available for 1990-1993, 1995, and 1998-1999; we estimated yearly disbursements using the average of neighboring disbursements
	Correspondence			X	Annual loan disbursements from 2001 through October 2017 were provided.

Institution	Data source	Commitments	Cumulative disbursements	Yearly disbursement	Notes
**Asian Development Bank**	Online Projects Database	X	X		As yearly disbursement amounts are not provided in the online database, we estimated yearly disbursements by allocating cumulative disbursements over each year of the project.
	OECD-Creditor Reporting System	X			To maintain continuity with previous estimate, yearly disbursement amounts from the CRS were not used.
**InterAmerican Development Bank**	Online projects database	X	X		As yearly disbursement amounts are not provided in the online database, we estimated yearly disbursements by allocating cumulative disbursements over each year of the project.
	Correspondence			X	Loan disbursements from January through October 23, 2017 were provided, along with projected disbursements for October 24 through December 2017.

eFigure 7 Disbursements by the African Development Bank

The orange line with triangles shows estimated disbursements based on the Compendium of Statistics from 1990 through 2001 and actual disbursements received from 2001 onwards.

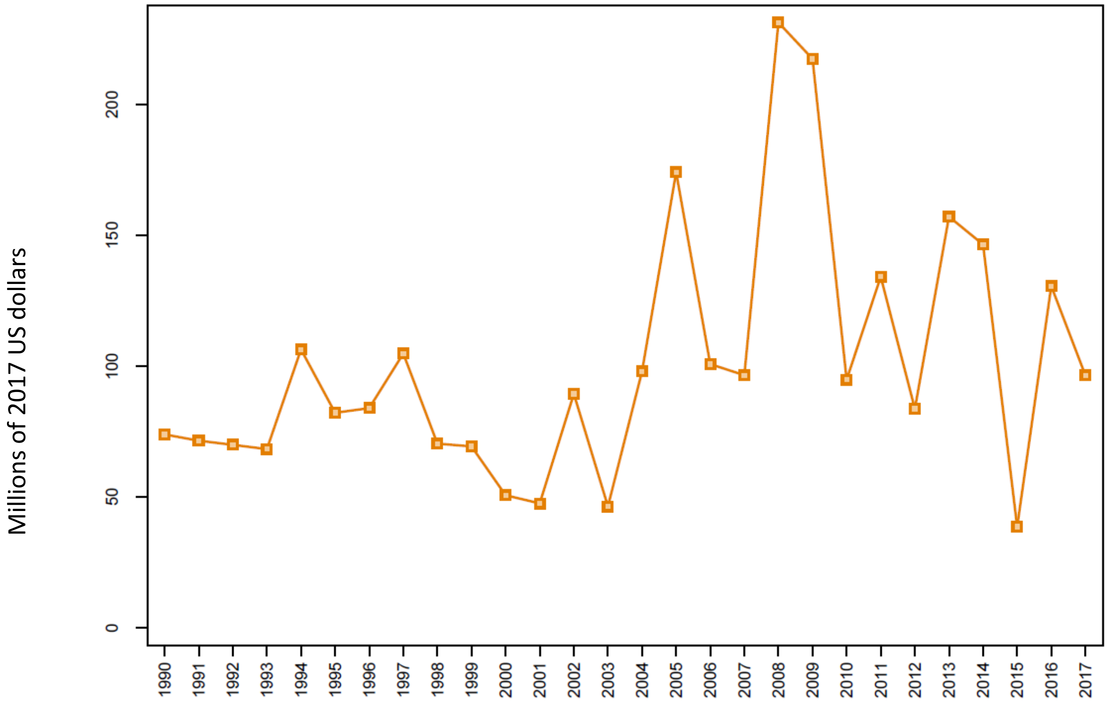

Source: IHME DAH Database (2017) and African Development Bank Compendium of Statistics.

eFigure 8 Commitments and disbursements by Asian Development Bank

The dashed green line shows commitments from the Asian Development Bank's (ADB) online projects database. The orange line shows smoothed disbursements from the online projects database.

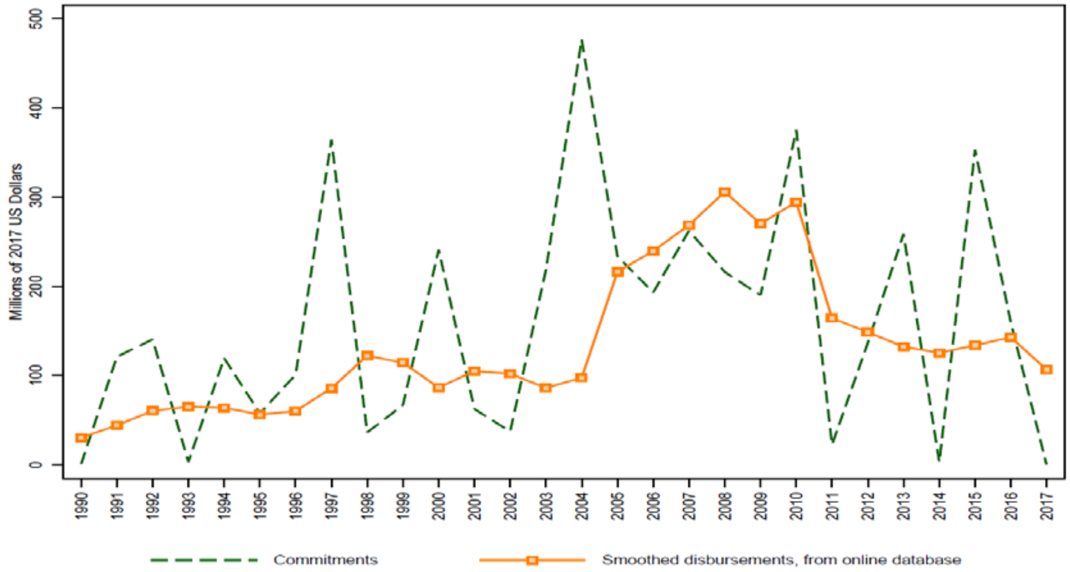

Source: IHME DAH Database (2017)

eFigure 9 Commitments and disbursements by Inter-American Development Bank

The dashed green line shows commitments from the Inter-American Development Bank's (IDB) online projects database. The orange line shows smoothed disbursements from the online projects database, and from correspondence for 2017.

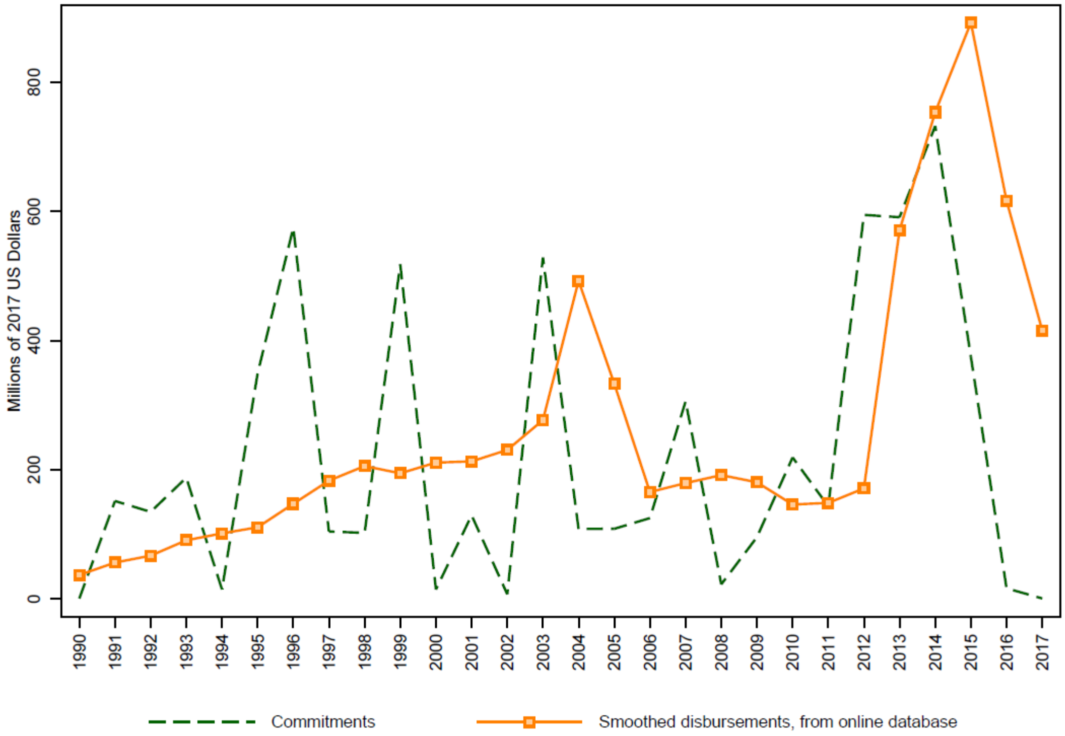

Source: IHME DAH Database (2017) and correspondence

Tracking contributions from GFATM and GAVI

The Global Fund to Fight AIDS, Tuberculosis and Malaria

The grants database made available online by the Global Fund to Fight AIDS, Tuberculosis and Malaria (GFATM) provides grant-level commitments and annual disbursements from its inception in 2002 to the present year.[23] In addition, sources of funding were compiled from the GFATM contributions dataset and annual reports, all downloaded from the GFATM website.[24,25] Regional grants were split evenly between all countries identified in the regional grant documents found on the GFATM website. eFigure 10 shows GFATM's annual contributions received from public and private sources. eFigure 10 shows GFATM's annual commitments and disbursements from its project database from 2002 through 2017.

eFigure 10 Contributions received by the Global Fund to Fight AIDS, Tuberculosis and Malaria

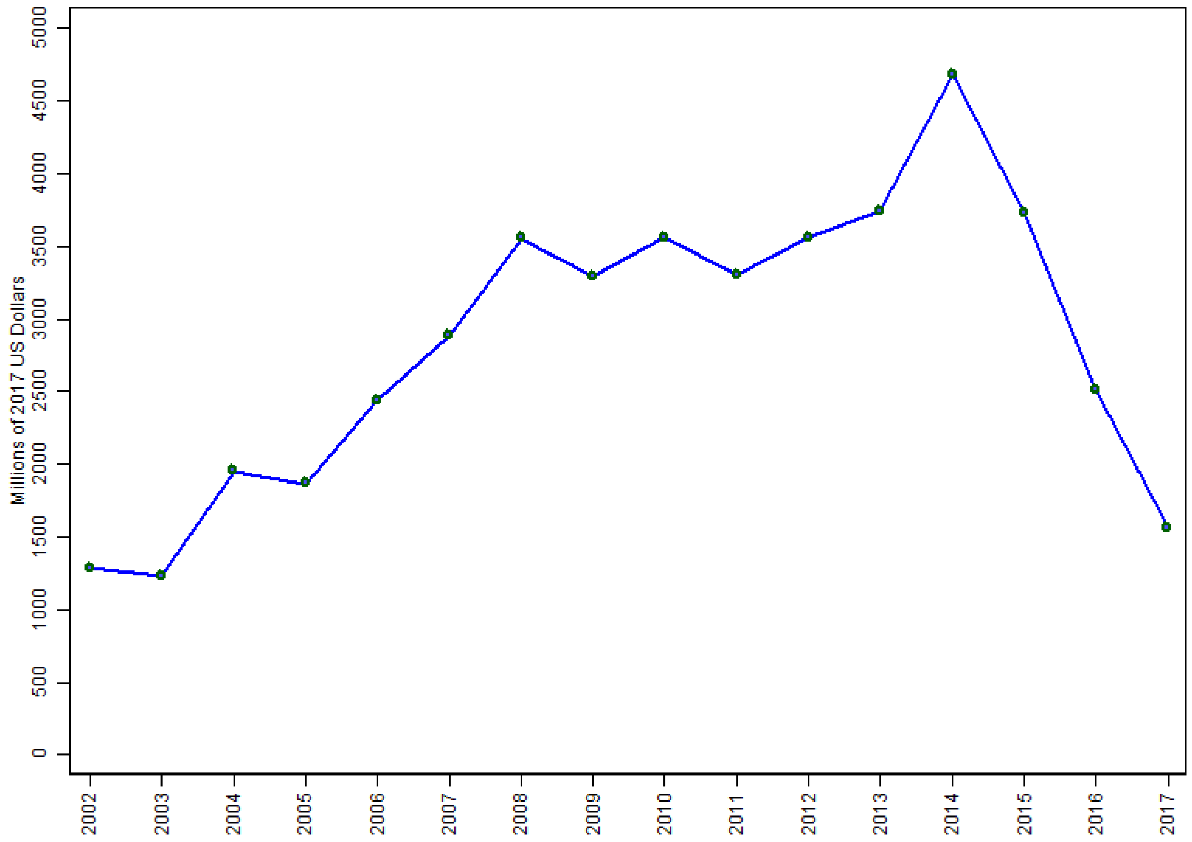

Source: GFATM pledges and contributions 2017

eFigure 11 The Global Fund to Fight AIDS, Tuberculosis and Malaria's commitments and disbursements

The dashed green line shows commitments from the Global Fund to Fight AIDS, Tuberculosis and Malaria's (GFATM) online grants database. The orange line shows disbursements from the online grants database.

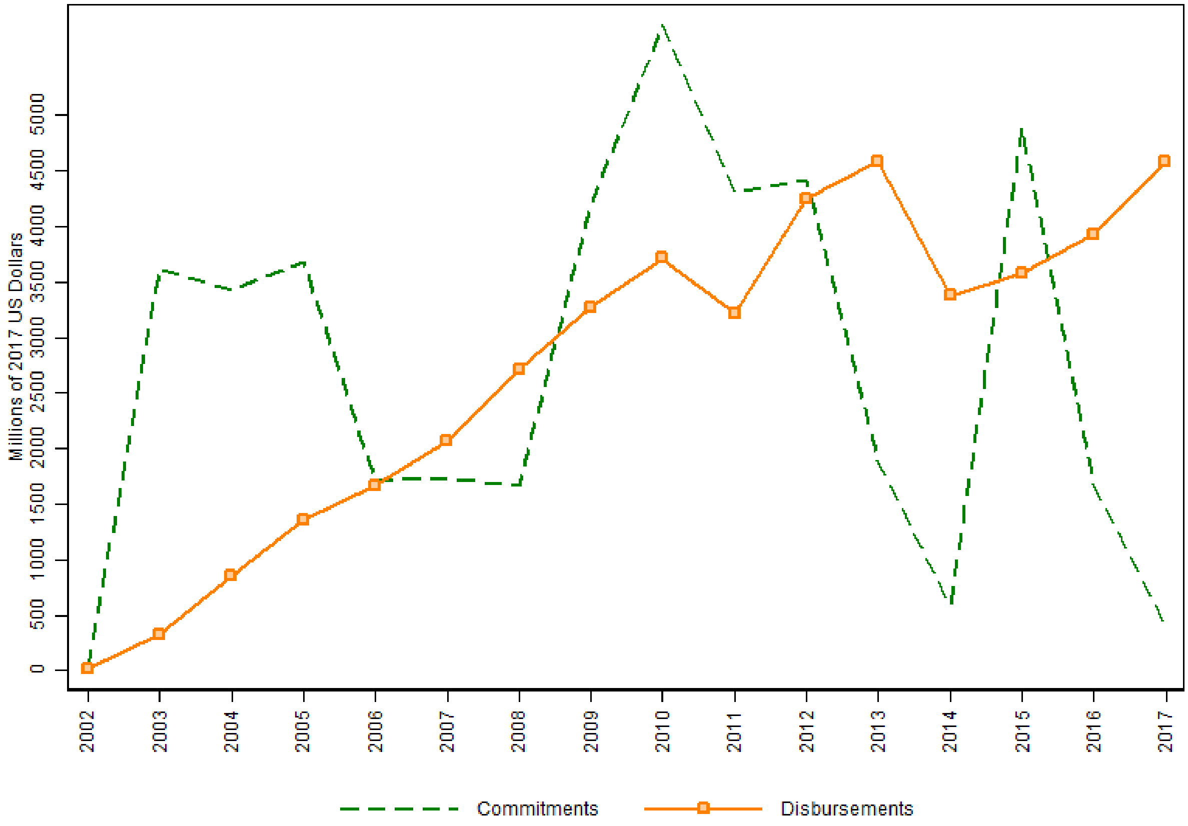

Source: IHME DAH Database (2017)

Gavi, the Vaccine Alliance

Gavi provided publicly available project-level data on commitments, disbursements, and investment cases from 2000 through the present.[19,22] Gavi's annual DAH was defined as the sum of (1) project-level disbursements by year paid; (2) investment cases (one-time investments in disease prevention and control); and (3) administrative and work plan costs. Data from Gavi's online databases include expenditure for (1) and (2), but not (3). However, project level data from the CRS for 2007-2012 did include administrative and work plan costs, so disbursements data from the online database were adjusted to match the CRS in those years. The average fraction of administrative and work plan costs was added to total disbursements in 2000-2006 and 2013-2015, the years in which the CRS did not include these data. Contributions data from Gavi's website as well as annual reports from the International Finance Facility for Immunisation (IFFIm) and Advance Market Commitment for Pneumococcal Vaccines were used to determine Gavi's annual income.[20,21,79]

All of the data sources used for Gavi estimates were complete through 2016. Donor contributions received and outstanding pledges data were available on Gavi's website. The unadjusted total pledges were used as total disbursements for 2017. . Gavi disbursements were assigned to health focus areas including child and newborn vaccines, HSS, and non-communicable disease as documented in eTable 6 above. Of note, we reclassified all GAVI health system strengthening projects as maternal, newborn, child health specific health system strengthening disbursements

eFigure 12 Gavi's income and disbursements

The dashed green line shows commitments from Gavi's online database. The dashed orange line shows the disbursements from Gavi's online database, which are the sum of project-level disbursements and investment cases. These data are adjusted using Gavi expenditure data reported to the Creditor Reporting System (CRS) to add administrative and work plan costs to the total. Adjusted disbursements are shown by the solid orange line.

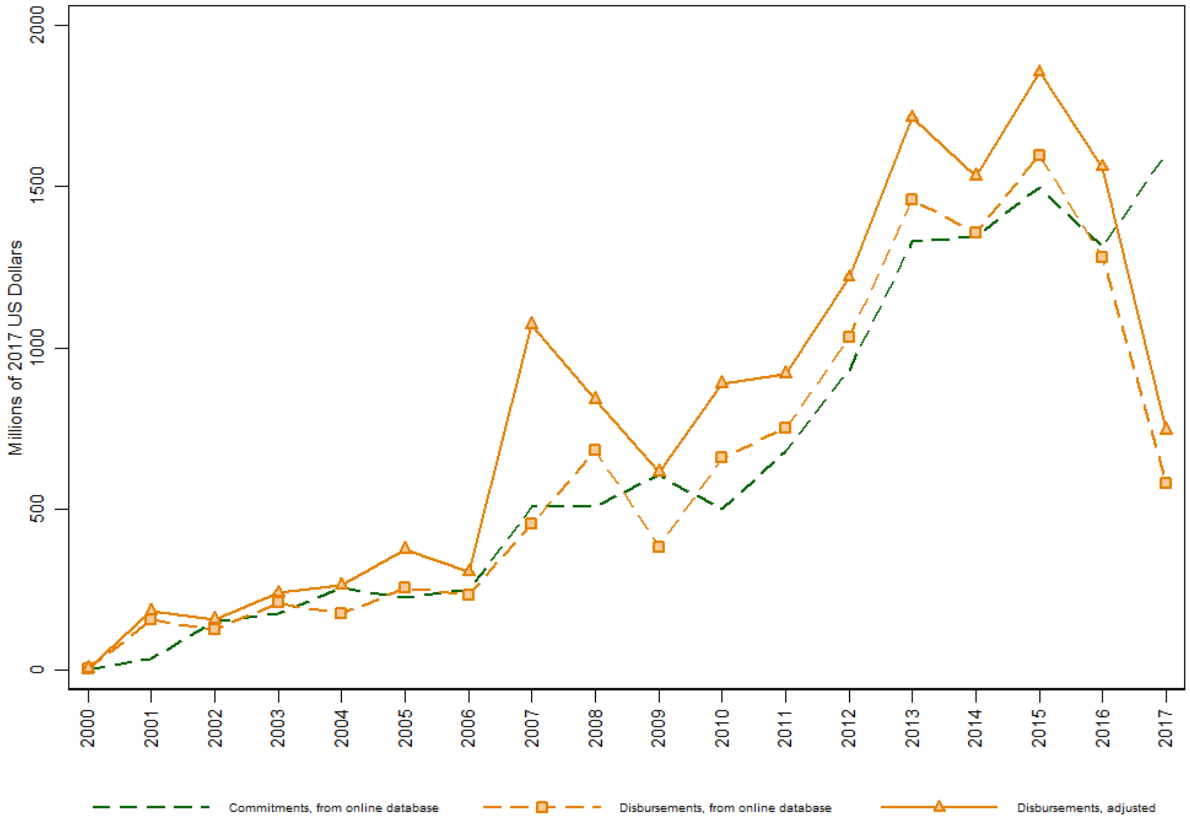

Source: IHME DAH Database (2017)

Tracking expenditure by United Nations Agencies active in the health domain

Data on income and expenditures were collected for six UN agencies: WHO, UNICEF, UNFPA, UNAIDS, UNITAID and PAHO. The data sources and calculations for each are described in detail below. Similar to the bilateral channels, we extracted budget data for the UN agencies to predict DAH for years for which we did not have health expenditure data. Model choices and budget measures for UN agencies are presented in eTable 4.

World Health Organization

Data on WHO's budgetary and extrabudgetary income and expenditure were compiled from annual reports and audited financial statements released by WHO.[80] Income data were extracted from WHO's assessed and voluntary

contributions, while expenditure data were extracted from both budgetary and extrabudgetary spending reports. As the financial statements represent activities over a two-year period, both income and expenditure data were divided by two, in order to approximate yearly amounts, and dollars were deflated using the US GDP deflator specific to the reporting year. Expenditures from trust funds, regional offices tracked separately, and associated entities not part of WHO's program of activities, such as UNAIDS and GFATM trust funds were excluded. Expenditures from supply services funds were also excluded, as these expenditures pertain to services provided by WHO but paid for by recipient countries. Additionally, WHO projects tracked as paid Ebola expenditure were extracted from the UNOCHA Financial Tracking System database and included as WHO health expenditure.

Disbursement data were not available for WHO in 2017. Much like the bilateral agencies, the ratio of DAH to the total program budget was estimated for 1990-2016 and then predicted for 2017 using the single-year average of previous year. The predicted ratio was then multiplied by the observed program budget for 2017 to get the estimates of DAH.

United Nations Population Fund

Data on income and expenditure were extracted for UNFPA from its audited financial statements.[70] As the 1990-2005 statements represent activities over a two-year period, income and expenditure data were divided by two in order to approximate yearly amounts.

Income and expenditures associated with procurement and cost-sharing activities were excluded from estimates of health assistance because UNFPA uses cost-sharing accounts when a donor contributes to UNFPA for a project to be conducted in the donor's own country. Since this money can be considered domestic spending that goes through UNFPA before being returned to the country in the form of a UNFPA program, it is not included in calculations of total DAH. UNFPA's additional expenditures for these projects come from trust funds or regular resources and are therefore, captured in our estimates. To estimate disbursements by health focus areas, UNFPA's total health expenditure was multiplied by the proportion of funding reported for each program area from annual reports from 1997 through 2013 and from the UNFPA transparency portal for 2014 through 2016. Maternal and child health spending classified as "other" was split equally between the maternal and child health program areas. Additionally, UNFPA projects tracked as paid Ebola expenditure were extracted from the UNOCHA Financial Tracking System database and added to UNFPA health expenditure.

The disbursement data for UNFPA were available through 2016. For year 2017, much like the bilateral agencies, the ratio of DAH and income was estimated for 1990-2016 and then predicted for 2017 using the three-year weighted average of previous years. The predicted ratio was multiplied by observed income to estimate DAH for 2017.

United Nations Children's Fund

Data on income and expenditure for UNICEF were extracted from its audited financial statements.[67] As these statements represent activities over a two-year period from 1990-2011, income and expenditure data were divided by two in order to approximate yearly amounts. The audited financial statements from 2012 onwards are produced on an annual basis.

Since UNICEF's activities are not limited to the health sector, the fraction of UNICEF's expenditure that was for health was estimated using either financial data from correspondence (2001-2013 observed data used to estimate 1990 through 2000 expenditure) or a combination of annual reports and annual results reports from 2014 through 2016. The annual results reports provide the proportion of funding for each program area, the average of 2014 through 2016 proportions was used to estimate the spending proportion for the years 1990 through 2013. In the annual results report, HIV/AIDS funding was reported separately from health funding so the percentages spent on each health program were proportioned based on total spending for Health. Furthermore, UNICEF projects tracked as paid Ebola expenditure were extracted from the UNOCHA Financial Tracking System and added to estimates for UNICEF's health expenditure.

The product of observed program budget and the weighted average of the DAH to budget ratio (placing one-half weight on the one-year lagged ratio, one-third weight on the two-year lagged ratio, and one-sixth weight on the three-year lagged ratio) was used to predict DAH in 2017.

Joint United Nations Programme on HIV/AIDS

UNAIDS income and expenditure data for both its core and noncore budgets were extracted from its audited financial statements.[65] As financial data are provided on a biennial basis in all years except for 2012 and 2013, the values were divided by two to obtain yearly amounts for all biennium data. Dollars were deflated using the US GDP deflator specific to the reporting year.

For UNAIDS, budget measures were available only for a subset of reported total disbursements. UNAIDS reported total expenditure, which combined Unified Budget and Workplan (UBW) and non-UWB components, but only UBW budget data were available.[66] To predict DAH for UNAIDS in 2017, disbursements in those years were calculated by multiplying the observed UBW budget by the three-year weighted average of the ratio of DAH to the UWB budget (placing one-half weight on the one-year lagged ratio, one-third weight on the two-year lagged ratio, and one-sixth weight on the three-year lagged ratio). UNAIDS disbursements were assigned to HIV/AIDS and TB program areas as documented in eTable 6 above.

UNITAID

Data on project level disbursement was obtain through correspondence with UNITAID. Income data was extracted from the annual financial statements downloaded from UNITAID's website. The project level data provided covered project disbursements from 2007 through 2016. To estimate the envelope for 2017, we used the weighted average of commitments from the last three years for which that data was available. Commitment data was extracted from the audited financial statements.

Pan American Health Organization

The Pan American Regional Office for WHO, or PAHO, reports its income and expenditure in its biennial financial report.[10,81] The funds transferred through the "Rotating Fund" were excluded because developing countries fund this procurement of health commodities which are then used within that funding country , and it therefore does not fit the definition of DAH.

As the financial data are provided on a biennial basis (with the exception of 2010 through 2016, where single-year financial reports were available), the quantities were divided by two to obtain yearly amounts. Dollars were deflated using the US GDP deflator specific to the reporting year.

Correspondence with PAHO revealed that data from the financial statements include both Program and nonProgram funds. The latter include funds that countries provide PAHO, so that PAHO can reinvest these funds into the countries' national health systems. These funds should not be included as development assistance for health, and PAHO provided corrected disbursement numbers for 2008 to 2013. The corresponding disbursement numbers for 2014 and 2015 were identified in the PAHO End-of-Biennium Assessment 2014-2015. These funds were provided as biennial disbursements, so they were divided by two to obtain yearly disbursements. The ratio of Program disbursements numbers provided by PAHO and the sum of Program and non-Program funds collected from financial statements was taken for the years 2008 to 2015. The average ratio was calculated, and this ratio was multiplied

through disbursement numbers collected from financial statements from earlier years. In this way, Program and non-Program funds collected from audited statements from earlier years were adjusted to estimate DAH.

For PAHO, disbursement data were not available for 2016 and 2017. PAHO provided budget information along with disbursements for 2008 to 2017. The average ratio between spending and budget was calculated over the years 2008 to 2015, and this ratio was used to estimate 2016 and 2017 disbursements.

Tracking development assistance for health from private foundations

Previous studies on foundations outside the US have documented the severe paucity of reliable time series data and lack of comparability across countries.[82] Hence, this research focused efforts on tracking only US foundations.

US Foundations

The Foundation Center maintains a database of all grants of $10,000 or more awarded by over 1,000 US foundations. The Foundation Center has coded each grant by sector and international focus and therefore is able to identify global health grants. IHME purchased a customized dataset with cross-border health grants and health grants to US-based international programs from 1992 to 2015 from the Foundation Center.[32] Grants from BMGF, which were tracked separately, were excluded. Additionally, grants to channels that this research already tracks were excluded.

The Foundation Center adopted a new classification methodology as of FGH 2016. The Foundation Center was able to provide historic data based on the new classification system from 2002 to 2012. In order to obtain the series from 1990 to 2001, we multiplied a weighted fraction calculated based on both old and new classification data values from 2002 through 2004 by the old data series (1992 to 2001) we had previously obtained.

$$
\begin{aligned}
(Weighted\ fraction) \\
&= \left(\frac{1}{2}\right)\left(DAH_{new\ classification}\right)/\left(DAH_{old\ classification}\right)2002 \\
&+ \left(\frac{1}{3}\right)\left(DAH_{new\ classification}\right)/\left(DAH_{old\ classification}\right)2003 \\
&+ \left(\frac{1}{6}\right)\left(DAH_{new\ classification}\right)/\left(DAH_{old\ classification}\right)2004
\end{aligned}
$$

$$(DAH\ Estimate_t) = (Weighted\ fraction)(DAH\ Observed_t)$$

where DAH Observed is the old data values for the series 1990 through 2001

To estimate total health grants in 1990-1991 and 2014-2017, natural log of US foundation DAH was regressed on the lagged natural log of US GDP per capita and year using ordinary least squares estimation. The missing years of data were predicted based on estimated regression coefficients from the equation. Exponents of the predicted values were used as final estimates

$$(ln\ Foundation_t) = \alpha + 1.\beta_1(\ln US\ GDP\ per\ capita_t) + \beta_2(year_t) + \varepsilon$$

Details on how we estimated the cost of providing technical assistance and program support for these US foundations are highlighted below in the section titled calculating the technical assistance and program support component of development assistance for health from loan-and grant-making channels of assistance.

Bill & Melinda Gates Foundation

BMGF has been the single largest grant-making institution in the health domain since 2000; hence, additional

research was undertaken to accurately capture its annual disbursements. BMGF's IRS 990PF filings for years 1999-2008, which report all global health grants disbursed per year, were downloaded from the BMGF website. Additionally, disbursement data for years 2009-2016 were collected from the BMGF online grants database, the OECD CRS and personal correspondence. The OECD CRS data was used to identify NGOs that are double-counted from other data sources.

An ordinary least squares linear regression model was used to predict the disbursement for BMGF for 2017. Since there is a strong correlation between market trends and BMGF annual disbursements, market data including lagged US GDP, lagged yearly average of Berkshire stock returns, lagged yearly average of the Russell Index, and lagged total assets of the BMGF Trust were utilized to predict the total disbursement for year 2017.

$$(BMGF\ total\ disbursement_t) = \alpha + \beta_1(US\ GDP\ per\ capita_{t-1}) + \beta_2(Berkshire\ stock\ returns_{t-1}) + \beta_3(Russell\ Index_{t-1}) + \beta_4(BMGF\ total\ asset_{t-1}) + \varepsilon$$

BMGF's predicted DAH was adjusted to account for in-kind DAH and double-counting. The difference between

BMGF's final DAH and DAH without in-kind added and double-counting removed from 2003-2016 was regressed using ordinary least squares on DAH without in-kind added and double-counting removed and year. The predicted difference was then subtracted from the predicted DAH from the previous regression for 2017.

Tracking non-governmental organizations

Currently, there are no centralized, easily accessible databases for tracking program expenses of the thousands of NGOs based in high-income countries that are active in providing development assistance and humanitarian relief worldwide. This study relied on CRS data and the only comprehensive data source identified for a large subset of these NGOs, namely the United States Agency for International Development's Report of Voluntary Agencies (USAID's VolAg report).[27] The report, which includes both US-based and international NGOs that received funding from the US government, provides data on domestic and overseas expenditures for these NGOs as well as their revenue from US and other public sources, private contributions, and in-kind. Total revenue and expenditure data obtained from the NGOs' IRS tax forms, accessed through the GuideStar online database, were also used in tracking NGOs incorporated in the US.[26]

First, in order to track disbursements from OECD donor countries to NGOs, we utilized channel codes present in the CRS database. The code 21000 identified international NGOs and the code 22000 identified donor-country-based NGOs. In order to remove double-counting, we conducted a keyword search on channels where the donor country was the United States to exclude NGOs present in the USAID VolAg report. Allocation of funding to health focus areas for NGOs tracked through the CRS was assigned as described in the section "DISAGGREGATING BY HEALTH FOCUS AREA", based on a keyword search of five descriptive variables in the CRS: project title, short description, long description, channel name, and channel reported name. For NGOs tracked in the USAID VolAg report, allocation of funding to health focus areas was assigned as described in the section "DISAGGREGATING BY HEALTH FOCUS AREA", based on a keyword search of the NGO's description given in the VolAg report.

In order to use the USAID VolAg data, several challenges were overcome. We outline these challenges here and discuss below the methods employed to estimate a consistent series of DAH channeled through NGOs despite these challenges. First, with the exception of BMGF, it was impossible to track the amount of funding from US

foundations routed through US NGOs, which may have led to double-counting in estimates of total health assistance. The second challenge relates to the incompleteness of the universe of NGOs captured through the USAID report. The report provides data on NGOs that received funding from the US government. While this covers many of the largest NGOs, it is not a comprehensive list. A related problem is that the VolAg report only includes NGOs that received funds in a given year. While many of the largest NGOs are consistently funded by the US government and are therefore in the report every year, not all NGOs are reported across all years. Third, health sector-specific expenditure is not reported in the VolAg or systematically reported in IRS tax forms. The VolAg does report overseas expenditure but does not disaggregate this expenditure by sector. Fourth, complete data are lacking in several time periods. The 2016 VolAg provided data through 2014. For NGOs incorporated in the US, IRS tax forms were obtained. Furthermore,
prior to 1998 the VolAg report did not include international NGOs. Attempts were made to compile other data on the health expenditures of the top international NGOs, in terms of overseas expenditure, by searching other websites for financial documents and contacting these organizations directly. Getting reliable time series data before 2000 proved to be extremely difficult for even this small sample of international NGOs.

Estimates of the share of overseas expenditure spent on health-related projects drew upon a sample of NGOs for which such data were available. Collecting financial data on health expenditures for each NGO would have been prohibitively time-consuming. Therefore, a sample of NGOs was drawn from the list for each year; the sample included the top 30 NGOs in terms of overseas expenditure and 20 randomly selected US-based NGOs from the remaining pool, with the probability of being selected set proportional to overseas expenditure. Next, health expenditure data were collected for each NGO in this sample by seeking out annual reports, audited financial statements, 990 tax forms, and data from NGO websites. Health expenditure was carefully reviewed to ensure that expenditures on food aid, food security, disaster relief, and water and sanitation projects were not included. eTable 9 summarizes the number of NGOs included each year in the USAID report, the number of NGOs in the sample by year, and the number of NGOs for which health expenditure data were successfully compiled in 2016. This table will be subsequently updated to reflect the 2017 sample.

eTable 9 Summary of US non-governmental organizations in the study

Year	Number of US NGOs in VolAG report	Number of international NGOs in VolAG report	Number of US NGOs in IHME sample	Number of US NGOs from sample for which data on health expenditure were found
1990	267	-	16	9
1991	334	-	19	14
1992	385	-	18	15
1993	411	-	17	12
1994	424	-	17	10
1995	416	-	16	12
1996	423	-	21	14
1997	425	-	23	18
1998	435	42	24	22
1999	438	-	33	28
2000	433	50	34	28
2001	442	51	33	26
2002	486	58	33	27
2003	507	54	42	32
2004	508	55	47	33

2005	494	59	45	36
2006	536	67	50	38
2007	556	68	50	40
2008	565	78	58	48
2009	580	90	57	45
2010	579	94	69	57
2011	595	112	73	63
2012	579	94	69	60
2013	519	113	69	52
**2014**	485	106	73	54

A random effects regression model was fit to predict health expenditure as a fraction of total expenditure using the data for the sampled NGOs. A random effects model was chosen because the sample included observations for several NGOs for multiple years. A random effects model allows for the effect of each type of NGO to be captured distinctly. This model was used to predict the fraction of expenditure spent on health for the remaining NGOs. To ensure that the predicted health fractions were bounded between zero and one, the regression utilized the logit-transformed health fraction as the dependent variable. Since several NGOs in the sample were observed for multiple years, the regression included a random effect that varied by NGO. Five of the nine variables used to predict the health fraction were drawn from the VolAg reports. They were (1) fraction of revenue from in-kind donations, (2) fraction of revenue from the US government, (3) fraction of revenue from private financial contributions, (4) overseas expenditure as a fraction of total expenditure, and (5) calendar year. The remaining four variables used to predict the health fraction were binary indicators that were constructed based on keyword searches on the NGO name and NGO description found in the VolAg. For both the NGO name and description, a keyword search was conducted to indicate whether the name or description was sufficiently health-related. Another keyword search was conducted independently on the NGO names and descriptions for keywords that indicated if the NGOs might focus on something other than health. These four indicators proved excellent predictors of health fractions.

$$
\begin{aligned}
logit(\,NGO - specific\ DAH_{it}) &= \alpha + \beta_1(Inkind\ contributions\ fraction_{it}) \\
&+ \beta_2(US\ government\ contributions\ fraction\ _{it}) \\
&+ \beta_3(Private\ financial\ contributions\ fractions_{it}) \\
&+ \beta_4(Overseas\ expenditure\ as\ a\ fraction\ of\ total\ expenditure_{it}) \\
&+ \beta_5(Health - related\ name_{it}) + \beta_6(Non - health - related\ name_{it}) \\
&+ \beta_7(Health - related\ description_{it}) + \beta_8(Non - health - related\ desciption_{it}) + U_i + \varepsilon
\end{aligned}
$$

Overseas health expenditure was calculated for individual NGOs in each year by multiplying the estimated health fraction and total overseas expenditure. For the NGOs that were sampled, the observed health fraction acquired through data collection was used. For the unsampled NGOs, the fitted fraction from the previously described random effects regression was used. Total overseas expenditure, reported in the VolAg, was not available for 2015-2017. For 2015 US-based NGOs, the 2015 NGO overseas fraction was calculated by regressing the logit transformed observed overseas fraction on a linear time trend using ordinary least squares, for each NGO independently. For these cases, the overseas health fraction was calculated as the product of estimated overseas fraction, estimated health fraction, and total expenditure found in the IRS 990 forms.

$$logit(Observed\ overseas\ health\ expenditure_i) = \alpha + \beta_i(year_t) + U_i + \varepsilon$$

At this point three reasons remained why the overseas health expenditure for some NGOs remained unknown. First, if an observation was non-US-based for 2015, then IRS tax forms were not available and total overseas expenditure could not be calculated. Second, for 2016 or 2017, no data were available. Finally, if an NGO was reported in the VolAg in multiple years but not for an intermittent year, no NGO-specific data were available for the gap year. This would be the case if an NGO received support from the US government one year and then again in a nonconsecutive year. For all three of these scenarios, a panel-based hierarchical linear regression model was used to fill in the overseas health expenditure gaps. Total overseas health expenditure (measured at the NGO-year level) was regressed on US GDP per capita and US bilateral DAH disbursed. Because the US government funds many of these NGOs, US bilateral DAH was an excellent predictor of NGO DAH. A flexible model was employed to allow both the GDP and US government DAH coefficients to vary randomly across NGOs, such that each NGO employed a unique (but not independent) relationship between overseas health expenditure, GDP, and US government DAH. A random intercept was also included to capture the significant unobserved heterogeneity present in our set of NGOs. Once fit, this model was used to predict overseas health expenditure for all remaining gaps.

$$(NGO\ DAH_{it}) = \alpha + \beta_{1i}(US\ GDP\ per\ capita_t) + \beta_{2i}(US\ bilateral\ DAH\ per\ capita_t) + U_i + \varepsilon$$

Expenditures financed from each revenue source were then calculated by multiplying overseas health expenditure by NGO-specific revenue fractions. Expenditures from in-kind sources were deflated by a constant fraction. This was determined by comparing the federal upper limit and average wholesale price valuations of drugs on the WHO’s Model List of Essential Medicines from the RED BOOK Expanded Database.[28,29] eFigure 13 and eFigure 14show the income and estimated overseas health expenditure, respectively, of the NGOs in the universe of US- and non-US-based NGOs that were tracked in this study from 1990 to 2014 in constant 2017 US dollars.

eFigure 13 Total revenue received by non-governmental organizations

The orange line shows total revenue for all sources, both public and private, received by NGOs. The green line shows estimates of private financial contributions to NGOs, while the blue line shows private in-kind donations to NGOs.

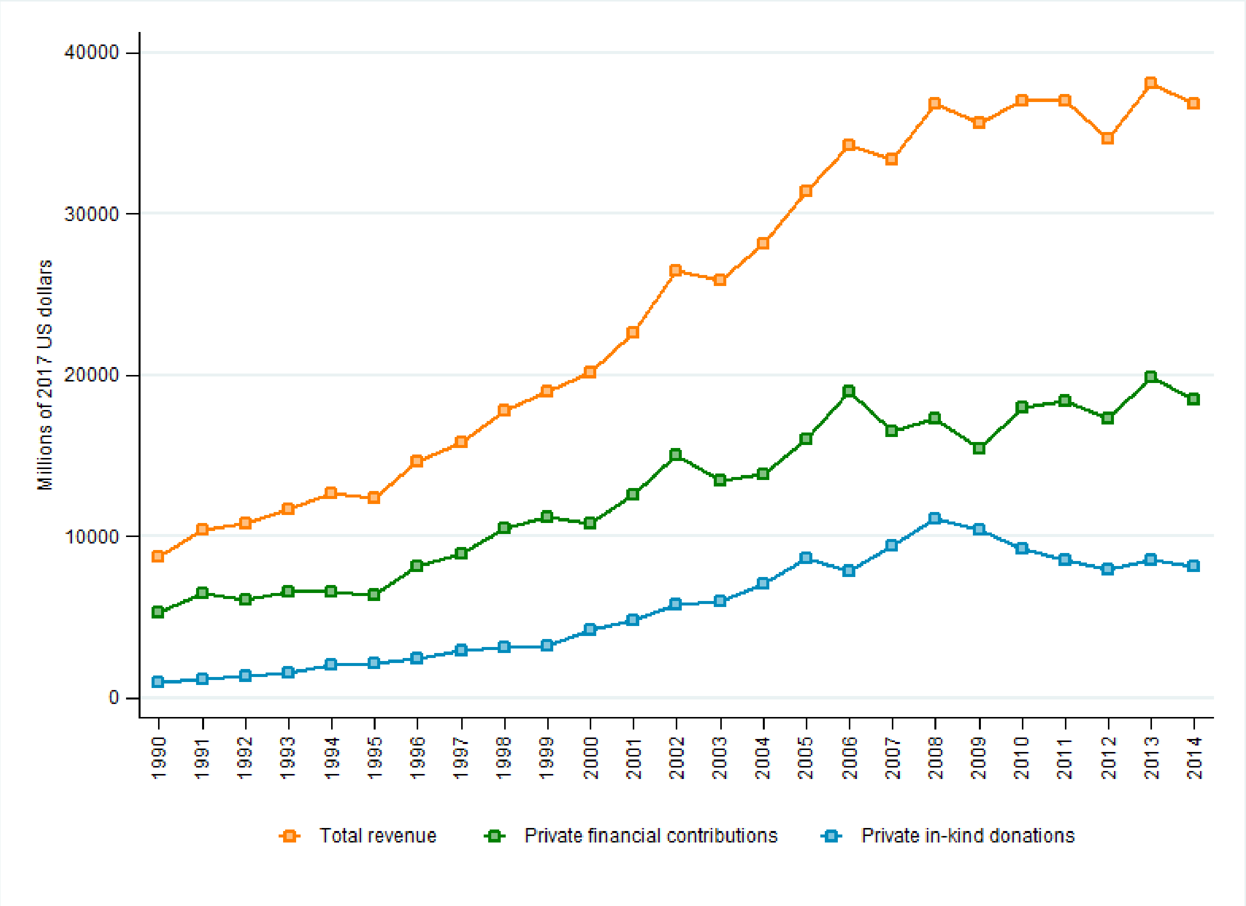

Source: IHME DAH Database (2017)

eFigure 14 Expenditure by non-governmental organizations

The orange line illustrates total overseas expenditure by NGOs, regardless of sector. The green line shows overseas expenditure by NGOs to health-specific recipients, or DAH.

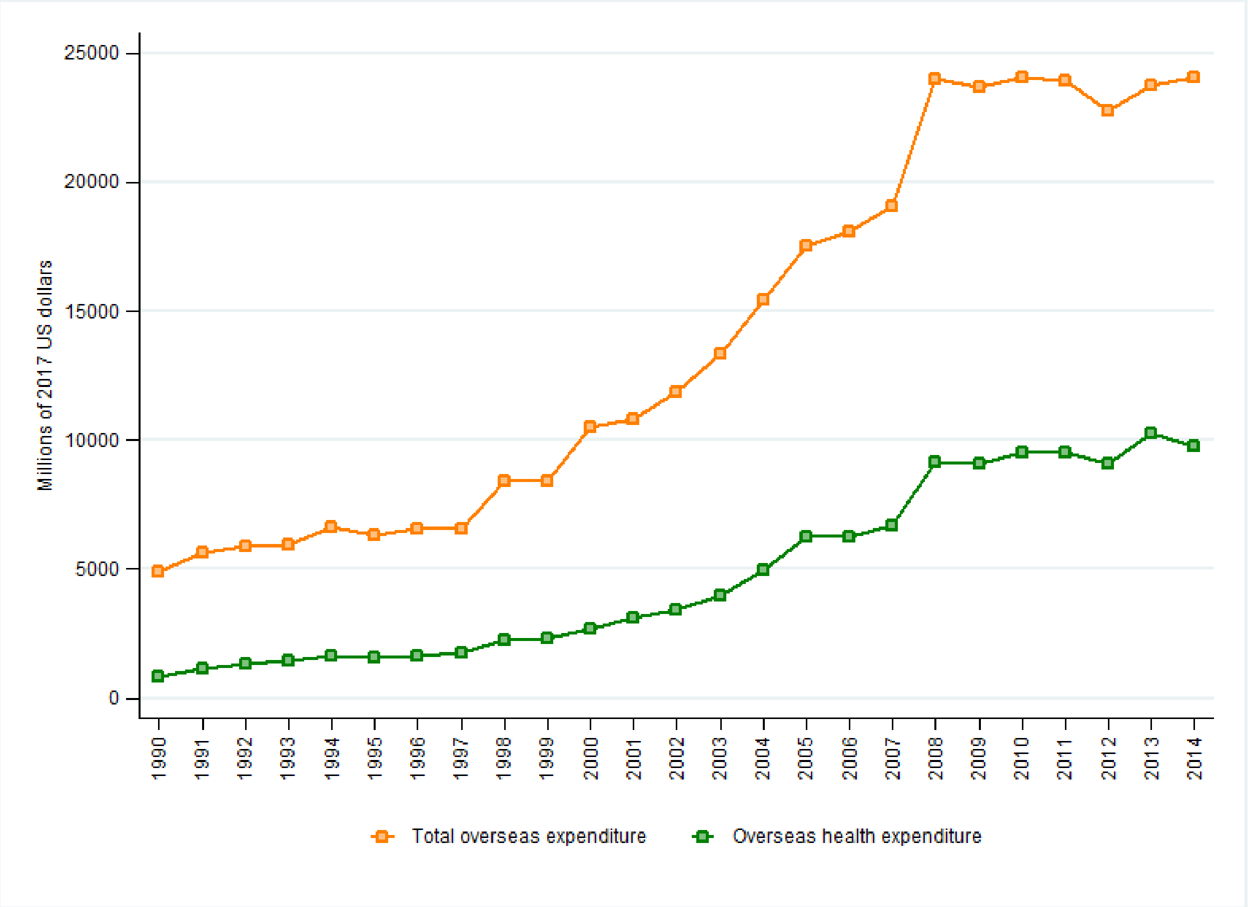

Source: IHME DAH Database (2017)

Calculating the technical assistance and program support component of development assistance for health from loan-and grant-making channels of assistance

The following methods were used to estimate the costs incurred by loan- and grant-making institutions for administering and supporting health sector loans and grants, which includes costs related to staffing and program management.

Data on the total administrative costs were compiled for a subset of institutions in our universe for which these data were readily available: IDA, IBRD, BMGF, GFATM, Gavi, USAID, and the UK Department for International Development (DFID). The sources of data for the institutions in this sample are summarized in eTable 10. The ratio of total administrative costs to total grants and loans was calculated for each source by year. It was assumed that the percentage of operating and administrative costs devoted to health would be equal to the percentage of grants and loans that were for health. In other words, if 20% of a foundation's grants were for health, the model assumed that 20% of administrative costs of the foundation were spent on facilitating these health grants. Given this assumption, the ratios of the observed administrative costs to grants/loans were used to estimate the in-kind contribution made by

each of these organizations toward maintaining their health grants and loans. For the institutions not in this sample, the ratio from the institution most similar to it was used to arrive at an estimate of in-kind contributions. For example, the average ratio observed for IDA and IBRD was used for all other development banks; the average of the ratios for BMGF for all other US foundations. Total in-kind contributions from all grant- and loan-making global health institutions are shown in eFigure 14. There was also considerable variation across channels in the ratio of in-kind contributions to financial contributions.

eTable 10 Summary of data sources for calculating in-kind contributions

Organization	Source	Notes
**BMGF**	990 tax returns (1999-2006)<br>BMGF Trust financial statements (2007-2016)	Used "cash basis" column to calculate ratio of total operating and administrative expenses to grants paid.<br>Used "grants expenditure" statement to calculate ratio of administrative expenditure to grants/program expenditure.
**GFATM**	Annual report financial statements	Calculated ratio of operating expenses to grants disbursed.
**Gavi**	Annual report financial statements	Calculated ratio of management, general, and fundraising expenses to program expenses.
**USAID**	US government budget database	Used outlays spreadsheet to calculate ratio of total outlays for USAID operating account to sum of outlays for bilateral accounts.
**DFID**	Annual report expense summary	Calculated ratio of DFID's administration expenses to DFID's bilateral program expenses from 2002 onward.
**IDA**	World Bank audited financial statements	Calculated ratio of management fee charged by IBRD to development credit disbursements.
**IBRD**	World Bank audited financial statements	Calculated ratio of administrative expenses to loan disbursements.

eFigure 15 In-kind contributions by loan- and grant-making DAH channels of assistance

This figure illustrates the proportions of financial and in-kind DAH disbursed by loan- and grant-making institutions. The proportion of in-kind DAH varies, based on the channel. The overall proportion of in-kind DAH received across all channels has grown over time.

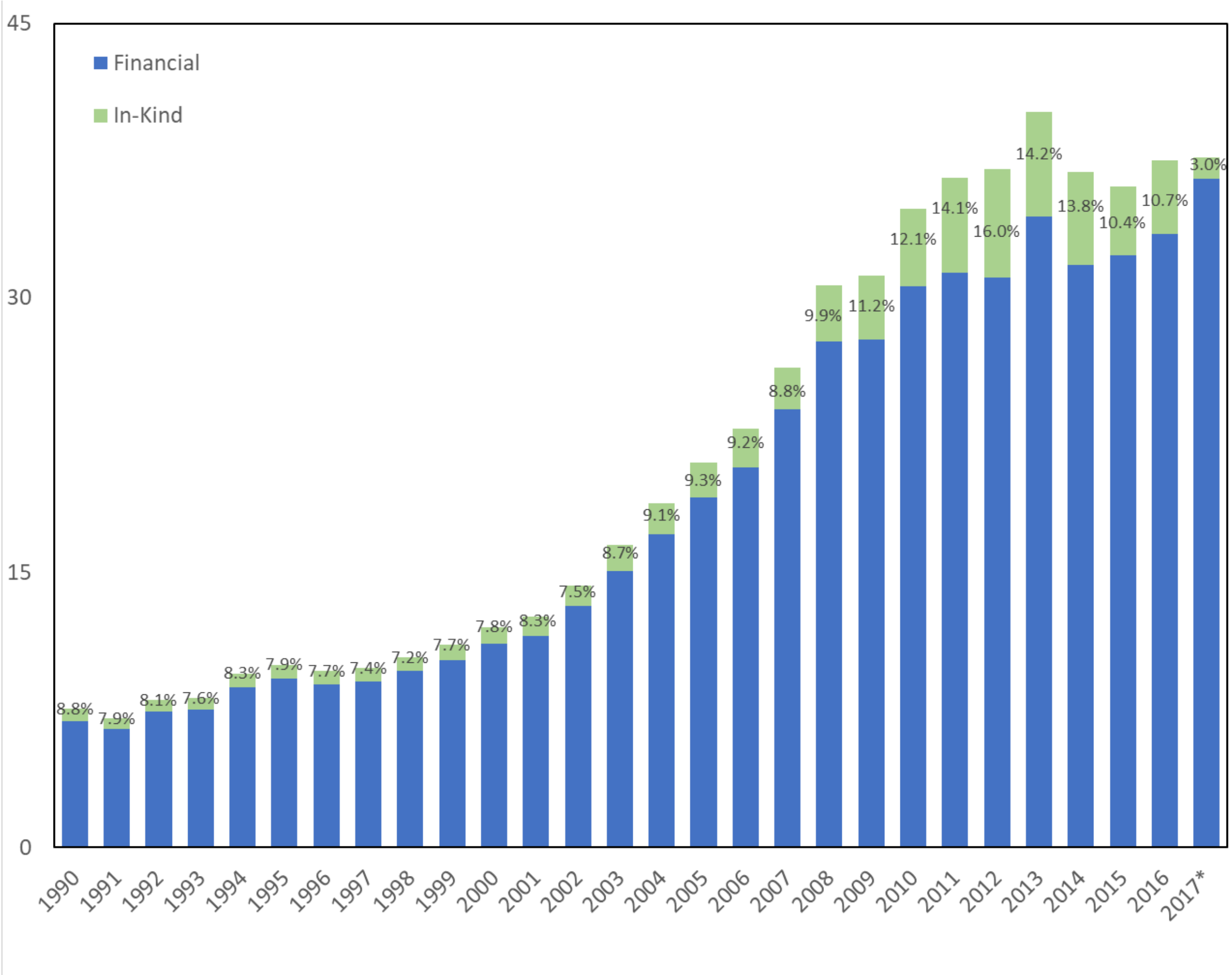

Source: IHME DAH Database (2017)

Comparing DAH by source and GDP

eFigure 16 DAH by source as a percentage of GDP, 2016

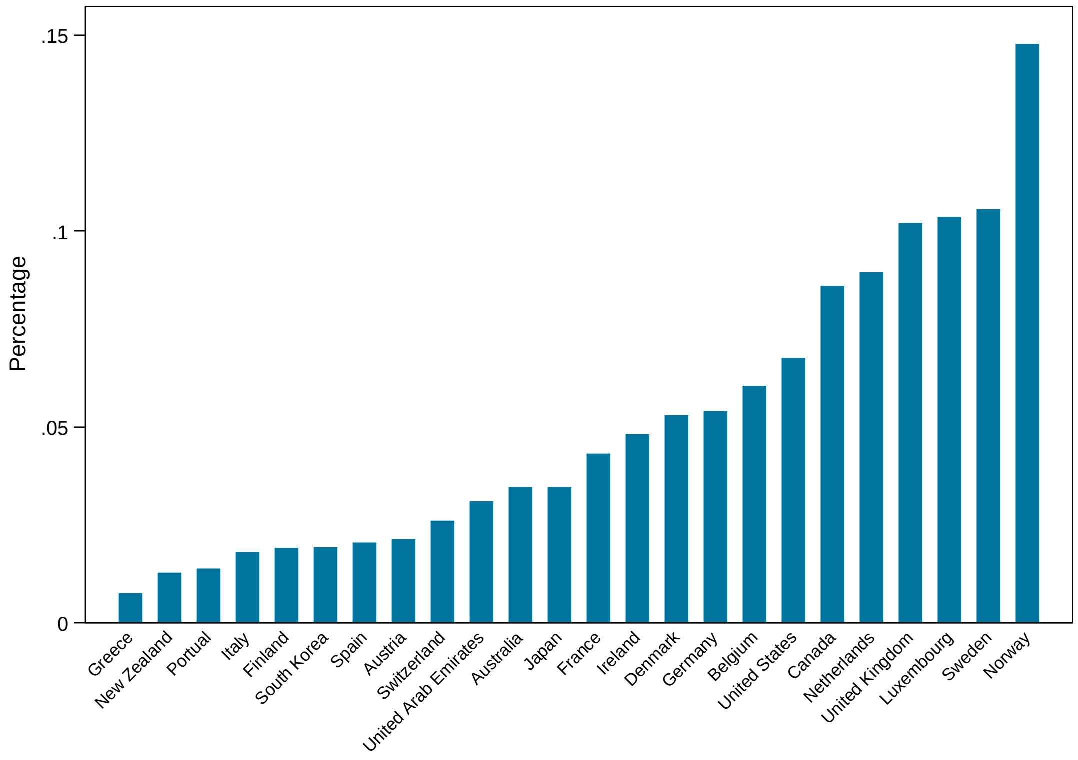

Source: IHME DAH Database (2017)

This figure illustrates DAH as percentage of GDP for each country as a source, across all channels. GDP data are constructed using methods developed by Spencer James and colleagues.[83]

SECTION 3. TRACKING TOTAL HEALTH SPENDING AND ITS COMPONENTS

Overview of GHED data cleaning process

We used Global Health Expenditure Database (GHED) data from the World Health Organization (WHO) to generate our estimates.[84] From the GHED, we extracted "Current health expenditure by revenues of health care financing schemes" for total health expenditure (THE), "Other revenues from households n.e.c" for out-of-pocket (OOP), "Gross Domestic Product" for GDP. We summed "Social insurance contributions", "Transfers from government domestic revenue (allocated to health purposes)", and "Compulsory prepayment (Other, and unspecified, than FS.3)" for government health spending (GHES). We summed "Voluntary prepayment", "Other revenues from corporations n.e.c." and "Other revenues from NPISH n.e.c." for pre-paid private (PPP).

To ensure we used the best possible data from the GHED, we evaluated the metadata also provided by GHED to establish the reliability of the data. To do so, we downloaded the metadata from the GHED website for each data point for the five indicators. We used the metadata to decide how each given data point should be weighted, from 0 to 5, with 0 meaning drop, and 1 through 5 meaning keep and treated these levels as linear weights.

To assign the weights, we established guidelines for the metadata that informed how the underlying data points should be weighted. We gave priority to factors such as complete, documented source information and penalized factors such as having been derived or estimated. eTable 11 describes the guidelines we created; any metadata that did not meet any of the disqualifying factors listed in eTable 11 were given a value of 5 to reflect highest reliability.

We used the four primary metadata variables from the GHED database: data type, method of estimation, comments, and sources. We applied the guidelines to each unique set of metadata across these four variables. In total, there were 2,598 unique sets of metadata, all of which can be found in eTable 12 PDF. We evaluated each of the 2,598 rows and assigned one of the levels 0 through 5, based on our guidelines. When two or more guidelines applied, we assigned that set of metadata the lower of the level values, unless our guidelines noted otherwise. In eTable 12, the following is the meaning for the color code used in the tables. The color code is assigned based on the level assigned to each row; Red – level 0, Orange – level 1, Yellow – level 2, Green – level 5, Blue – level to be decided based on levels of sub-components.

For a subset of data points, the metadata indicated that the data point was the sum of other data points (noted in eTable 12 with the level "TBD"). In these cases, if the indicator was a sub-indicator of GHES, PPP, or OOP, we assigned the data point a value of 2 to reflect that even though we could not determine if the sub-components existed, as they are not reported in GHED, we did not feel that being a sum warranted dropping the data. We assigned the summed GHES, PPP, and OOP indicators the lowest value of its sub-indicators. If the summed data point was THE, however, we assigned the data point the lowest value of its sub-components, the summed GHES, PPP, and OOP indicators.

After designating each of the 2,598 unique sets of metadata a value level, we applied these levels to the underlying data points. In total, we had 22,103 data points, as multiple data points shared the same unique set of metadata. Once the levels were applied to the data, we reassigned all high-income country data points that were a 0 based on the metadata to 3. We made this change to reflect that high-income countries have higher quality data and thus should not be dropped, but should also not be given the highest weight value. The high-income classification comes from the World Bank.[78]

The eTables 13-17 show the number of data points that we dropped based on the metadata globally and by country and region for each of the 6 indicators. In total for all indicators, we dropped 9,150 of 15,280 data points (59.9%). For each indicator, individually, we dropped 40 out of 3,056 data points (1.3%) for GDP; 1,717 of 3,056 data points (56.2%) for THE; 808 out of 3,056 (26.4%) data points for GGHE; 1,336 out of 3,056 data points (43.7%) for pre-paid private; and 1,291 out of 3,056 data points (42.2%) for OOP.

Currency exchange and deflation

To convert a metric (for example, DAH) from 2017 USD to 2017 PPP, the following steps were taken. First, we used the US deflator series to convert the series (DAH as example) from 2017 USD to nominal USD series for all country-years. Next, we converted the nominal USD series to nominal LCU (local currency unit) series by multiplying with country-year specific USD to LCU exchange rates. After which, we used country-year specific deflator series (based to year 2017) to convert from nominal LCU to 2017 LCU series. Finally, we converted from 2017 LCU to 2017 PPP (purchasing power parity) series using the country specific 2017 LCU to PPP conversion series.

eTable 11. Rules for assigning level values to metadata

Data type	Methods of estimation	Sources and/or Comments	Level
Blank			0
Estimated			0
	Derived by applying the sum of the components		Lowest level of the components
	Interpolated but with additional information		2
	Method description is unclear or provides very little information		0
	Time trend interpolation		1
	Uses data from other countries		0
		Abstract that's not from something documented	1 or method (whichever is bigger)
		Adjusted	0 or method (whichever is bigger)
		Adjusted using something	2 or method (whichever is bigger)
		Any suggestion that the WHO is unclear or unsure about some aspect of the data point's metadata	0 or method (whichever is bigger)
		Approximation	0 or method (whichever is bigger)
		Assumption	0 or method (whichever is bigger)
		Both blank	0 or method (whichever is bigger)
		Both with no intelligible information	0 or method (whichever is bigger)
		Budget address	1 or method (whichever is smaller)
		Calculation was used to generate the estimate	1 or method (whichever is bigger)
		Consultation/contact (without an additional documented source)	1 or method (whichever is smaller)
		Consultations with additional source, but no specifics and just consult is documented	1 or method (whichever is bigger)
		Currency conversion	1 or method (whichever is bigger)
		Data delivered/provided/reported by (a non-documented source)	1 or method (whichever is smaller)
		Data provided but not clear by whom, with an additional source if additional source is not documented	1 or method (whichever is smaller)
		Derived	0 or method (whichever is bigger)
		Estimated based on	1 or method (whichever is bigger)

Data type	Methods of estimation	Sources and/or Comments	Level
		Estimation	0 or method (whichever is bigger)
		Excludes (if it excludes what we do want)	0 (supersedes method)
		Extrapolated	0 or method (whichever is bigger)
		Forecasted	0 or method (whichever is bigger)
		Government department, no explicit documented source	1 or method (whichever is bigger)
		Government ministry, but no explicit documented source	1 or method (whichever is bigger)
		Includes (if it includes what we don't want)	0 (supersedes method)
		Inferred	0 or method (whichever is bigger)
		Interpolation	0 or method (whichever is bigger)
		Missing (if missing something that should be included)	0 (supersedes method)
		Modified	0 or method (whichever is bigger)
		Modified from something/modified using something	2 or method (whichever is bigger)
		Needs assessment	1 or method (whichever is smaller)
		Needs discussion	1 or method (whichever is smaller)
		Needs validation	1 or method (whichever is smaller)
		Needs verification	1 or method (whichever is smaller)
		Only provides hint of a source	1 or method (whichever is smaller)
		Projected	0 or method (whichever is bigger)
		Provides only a vague term that does not provide adequate information to infer or determine what the source is	0 or method (whichever is bigger)
		Reply	1 or method (whichever is smaller)
		Response	1 or method (whichever is smaller)
		Speech	1 or method (whichever is smaller)
		Sum of	2 (except for THE, which is lowest level of components)
		Total of	3 (except for THE, which is lowest level of components)
		Underestimated	0 (supersedes method)
		Unpublished	1 or method (whichever is smaller)
		Validated figures, but without specifics	2 or method (whichever is bigger)
		Weights	0 or method (whichever is bigger)

eTable 12. *This table is included at the end of the document due to the number of rows in the table.*

eTable 13. Gross Domestic Product (total number of observations: 3,056)

Location name	Number of dropped observations
Global	40
Upper Middle Income	11
Lower Middle Income	14
Low Income	15
North Africa and Middle East	21
Latin America and Caribbean	4
Sub-Saharan Africa	13
South Asia	2
Afghanistan	2
Algeria	3
Egypt	2
Iraq	2
Jordan	2
Libya	2
Morocco	2
Mexico	2
Mali	1
Pakistan	2
Sudan	2
South Sudan	12
Suriname	2
Syrian Arab Republic	2
Yemen	2

eTable 14. Total expenditure on health (total number of observations: 3,056)

Location name	Number of dropped observations
Global	1,717
High Income	51
Upper Middle Income	613
Lower Middle Income	682
Low Income	371
North Africa and Middle East	195
Sub-Saharan Africa	575
Central Europe, Eastern Europe, and Central Asia	247
High-income	20
Latin America and Caribbean	309
South Asia	62
Southeast Asia, East Asia, and Oceania	309
Afghanistan	16
Angola	16
Albania	15
Argentina	13
Armenia	8
Antigua and Barbuda	7
Azerbaijan	16
Burundi	14
Benin	11
Burkina Faso	5
Bangladesh	3
Bulgaria	6
Bahrain	1
Bosnia and Herzegovina	13
Belarus	12
Belize	16
Brazil	10
Barbados	4
Bhutan	14
Botswana	12
Central African Republic	14
China	7
Cote d'Ivoire	13
Cameroon	15

Location name	Number of dropped observations
Democratic Republic of the Congo	9
Congo	14
Colombia	16
Comoros	15
Cabo Verde Republic of	12
Costa Rica	6
Djibouti	16
Dominica	15
Dominican Republic	16
Algeria	16
Ecuador	15
Egypt	16
Eritrea	16
Ethiopia	15
Fiji	16
Micronesia (Federated States of)	16
Gabon	11
Georgia	5
Ghana	13
Guinea	16
Gambia	12
Guinea-Bissau	16
Equatorial Guinea	7
Grenada	16
Guatemala	3
Guyana	16
Honduras	16
Haiti	14
Hungary	2
Indonesia	9
India	16
Iraq	13
Jamaica	15
Jordan	16
Kazakhstan	16
Kenya	15
Kyrgyzstan	16
Cambodia	11
Kiribati	16
Lao People's Democratic Republic	15

Location name	Number of dropped observations
Lebanon	15
Liberia	16
Libya	12
Saint Lucia	15
Sri Lanka	15
Lesotho	16
Latvia	4
Morocco	16
Republic of Moldova	12
Madagascar	15
Maldives	16
Mexico	2
Marshall Islands	16
The former Yugoslav Republic of Macedonia	16
Mali	8
Myanmar	16
Montenegro	16
Mongolia	16
Mozambique	16
Mauritania	15
Mauritius	15
Malawi	7
Malaysia	2
Namibia	10
Niger	12
Nigeria	10
Nicaragua	7
Nepal	13
Oman	7
Pakistan	16
Panama	16
Peru	6
Philippines	16
Papua New Guinea	16
Paraguay	15
Romania	5
Russian Federation	3
Rwanda	12
Saudi Arabia	4
Sudan	16

Location name	Number of dropped observations
Senegal	11
Solomon Islands	16
Sierra Leone	12
El Salvador	13
Serbia	16
South Sudan	16
Sao Tome and Principe	16
Suriname	14
Swaziland	15
Seychelles	9
Syrian Arab Republic	16
Chad	15
Togo	15
Thailand	2
Tajikistan	10
Turkmenistan	16
Timor-Leste	16
Tonga	16
Trinidad and Tobago	6
Tunisia	15
United Republic of Tanzania	11
Uganda	3
Ukraine	8
Uruguay	7
Uzbekistan	16
Saint Vincent and the Grenadines	16
Venezuela (Bolivarian Republic of)	14
Viet Nam	16
Vanuatu	16
Samoa	16
Yemen	16
South Africa	9
Zambia	12
Zimbabwe	16

eTable 15. General Health Spending (total number of observations: 3,056)

Location name	Number of dropped observations
Global	808
High Income	16
Upper Middle Income	247
Lower Middle Income	342
Low Income	203
North Africa and Middle East	142
Sub-Saharan Africa	282
Central Europe, Eastern Europe, and Central Asia	50
High-income	2
Latin America and Caribbean	96
South Asia	26
Southeast Asia, East Asia, and Oceania	210
Afghanistan	14
Angola	2
Albania	2
Argentina	2
Armenia	3
Antigua and Barbuda	2
Burundi	11
Benin	7
Burkina Faso	5
Bangladesh	3
Bulgaria	3
Bahrain	1
Bosnia and Herzegovina	3
Belarus	2
Belize	4
Brazil	7
Central African Republic	7
Cote d'Ivoire	8
Cameroon	6
Democratic Republic of the Congo	1
Congo	9
Colombia	2
Comoros	7
Cabo Verde Republic of	3

Location name	Number of dropped observations
Costa Rica	2
Djibouti	16
Dominica	7
Dominican Republic	5
Algeria	1
Ecuador	3
Egypt	15
Eritrea	9
Ethiopia	12
Fiji	16
Micronesia (Federated States of)	16
Georgia	2
Ghana	5
Guinea	9
Gambia	10
Guinea-Bissau	9
Equatorial Guinea	7
Grenada	2
Guatemala	2
Guyana	3
Honduras	3
Haiti	5
Hungary	2
Indonesia	5
Iraq	12
Jamaica	2
Jordan	16
Kazakhstan	4
Kenya	11
Kyrgyzstan	4
Cambodia	3
Kiribati	13
Lao People's Democratic Republic	11
Lebanon	7
Liberia	1
Libya	13
Saint Lucia	2
Sri Lanka	3
Lesotho	7
Morocco	10

Location name	Number of dropped observations
Madagascar	3
Maldives	16
Mexico	2
Marshall Islands	16
Mali	8
Myanmar	1
Montenegro	7
Mozambique	3
Mauritania	2
Malawi	3
Malaysia	2
Namibia	8
Niger	2
Nigeria	2
Nicaragua	3
Nepal	10
Oman	7
Pakistan	13
Panama	8
Peru	5
Philippines	16
Papua New Guinea	16
Paraguay	6
Romania	2
Rwanda	9
Saudi Arabia	4
Sudan	16
Senegal	5
Solomon Islands	16
Sierra Leone	5
Serbia	4
South Sudan	13
Sao Tome and Principe	9
Suriname	14
Swaziland	1
Syrian Arab Republic	13
Chad	5
Togo	7
Turkmenistan	7
Timor-Leste	10

Location name	Number of dropped observations
Tonga	16
Tunisia	4
United Republic of Tanzania	4
Uganda	5
Ukraine	5
Saint Vincent and the Grenadines	5
Venezuela (Bolivarian Republic of)	2
Viet Nam	2
Vanuatu	16
Samoa	16
Yemen	9
Zambia	12
Zimbabwe	14

eTable 16. Out of pocket expenditures (total number of observations: 3,056)

Location name	Number of dropped observations
Global	1291
High Income	37
Upper Middle Income	421
Lower Middle Income	496
Low Income	337
North Africa and Middle East	173
Sub-Saharan Africa	511
Central Europe, Eastern Europe, and Central Asia	146
High-income	16
Latin America and Caribbean	178
South Asia	48
Southeast Asia, East Asia, and Oceania	219
Afghanistan	15
Angola	13
Albania	12
Argentina	13
Armenia	3
Antigua and Barbuda	5
Azerbaijan	12
Burundi	14

Location name	Number of dropped observations
Benin	10
Burkina Faso	5
Bangladesh	3
Bulgaria	7
Bahrain	1
Bosnia and Herzegovina	6
Belize	15
Brazil	2
Barbados	3
Bhutan	12
Botswana	10
Central African Republic	14
Cote d'Ivoire	10
Cameroon	15
Democratic Republic of the Congo	5
Congo	13
Comoros	14
Cabo Verde Republic of	11
Costa Rica	16
Djibouti	14
Dominica	14
Dominican Republic	12
Algeria	10
Ecuador	10
Egypt	16
Eritrea	15
Ethiopia	7
Fiji	10
Micronesia (Federated States of)	16
Gabon	10
Georgia	2
Ghana	13
Guinea	16
Gambia	12
Guinea-Bissau	16
Equatorial Guinea	7
Grenada	14
Guatemala	2
Guyana	16
Honduras	1

Location name	Number of dropped observations
Croatia	4
Haiti	12
Hungary	2
Indonesia	5
India	13
Iraq	14
Jamaica	6
Jordan	9
Kazakhstan	12
Kenya	14
Kyrgyzstan	5
Cambodia	11
Kiribati	13
Lao People's Democratic Republic	7
Lebanon	13
Liberia	15
Libya	16
Sri Lanka	2
Lesotho	15
Morocco	15
Republic of Moldova	3
Madagascar	14
Maldives	16
Mexico	2
Marshall Islands	16
Mali	6
Myanmar	4
Montenegro	13
Mongolia	10
Mozambique	11
Mauritania	12
Mauritius	10
Malawi	5
Malaysia	2
Namibia	7
Niger	12
Nigeria	10
Nicaragua	7
Nepal	8
Oman	7

Location name	Number of dropped observations
Pakistan	12
Panama	14
Peru	1
Philippines	4
Papua New Guinea	16
Paraguay	6
Romania	5
Rwanda	12
Saudi Arabia	4
Sudan	14
Senegal	10
Solomon Islands	16
Sierra Leone	15
El Salvador	2
Serbia	5
South Sudan	16
Sao Tome and Principe	9
Suriname	13
Swaziland	15
Seychelles	7
Syrian Arab Republic	15
Chad	13
Togo	15
Thailand	2
Tajikistan	8
Turkmenistan	16
Timor-Leste	16
Tonga	16
Trinidad and Tobago	5
Tunisia	9
United Republic of Tanzania	10
Uganda	5
Ukraine	7
Uruguay	3
Uzbekistan	14
Vanuatu	14
Samoa	16
Yemen	15
Zambia	11
Zimbabwe	15

eTable 17. Pre-paid private (total number of observations: 3,056)

Location name	Number of dropped observations
Global	1,336
High Income	51
Upper Middle Income	491
Lower Middle Income	486
Low Income	308
North Africa and Middle East	182
Sub-Saharan Africa	495
Central Europe, Eastern Europe, and Central Asia	186
High-income	15
Latin America and Caribbean	207
South Asia	33
Southeast Asia, East Asia, and Oceania	218
Afghanistan	14
Angola	13
Albania	7
Argentina	11
Armenia	5
Antigua and Barbuda	7
Azerbaijan	13
Burundi	13
Benin	10
Burkina Faso	5
Bangladesh	3
Bulgaria	6
Bosnia and Herzegovina	9
Belarus	7
Belize	16
Brazil	2
Barbados	4
Bhutan	8
Botswana	10
Central African Republic	14
Cote d'Ivoire	11
Cameroon	15
Congo	14
Colombia	2

Location name	Number of dropped observations
Comoros	15
Cabo Verde Republic of	12
Costa Rica	16
Czech Republic	3
Djibouti	16
Dominica	12
Dominican Republic	5
Algeria	11
Ecuador	7
Egypt	15
Eritrea	16
Ethiopia	8
Fiji	10
Micronesia (Federated States of)	16
Gabon	11
Georgia	2
Ghana	13
Guinea	15
Gambia	12
Guinea-Bissau	16
Equatorial Guinea	7
Grenada	11
Guyana	16
Honduras	1
Croatia	5
Haiti	11
Hungary	2
Indonesia	4
India	6
Iraq	16
Jamaica	6
Jordan	12
Kazakhstan	15
Kenya	11
Kyrgyzstan	15
Cambodia	11
Kiribati	11
Lao People's Democratic Republic	8
Lebanon	12
Liberia	13

Location name	Number of dropped observations
Libya	16
Saint Lucia	16
Sri Lanka	2
Lesotho	14
Morocco	13
Republic of Moldova	3
Madagascar	11
Maldives	15
Marshall Islands	16
The former Yugoslav Republic of Macedonia	14
Mali	6
Myanmar	5
Montenegro	16
Mongolia	2
Mozambique	15
Mauritania	12
Mauritius	10
Malawi	5
Malaysia	2
Namibia	7
Niger	6
Nigeria	10
Nicaragua	4
Nepal	4
Oman	7
Pakistan	12
Panama	14
Peru	1
Philippines	3
Papua New Guinea	16
Poland	2
Paraguay	7
Romania	5
Rwanda	12
Saudi Arabia	4
Sudan	15
Senegal	10
Solomon Islands	16
Sierra Leone	8
Serbia	8

Location name	Number of dropped observations
South Sudan	16
Sao Tome and Principe	14
Suriname	14
Slovakia	4
Swaziland	15
Seychelles	9
Syrian Arab Republic	16
Chad	14
Togo	14
Thailand	2
Tajikistan	9
Turkmenistan	16
Timor-Leste	16
Tonga	16
Trinidad and Tobago	5
Tunisia	10
Turkey	7
United Republic of Tanzania	11
Ukraine	6
Uruguay	4
Uzbekistan	12
Saint Vincent and the Grenadines	16
Venezuela (Bolivarian Republic of)	14
Vanuatu	14
Samoa	16
Yemen	14
Zambia	11
Zimbabwe	14

Statistical model to fill missingness in health expenditure variables

After the cleaning up of the data previously described above, we used Spatiotemporal Gaussian process regression (ST-GPR) in order to predict and fill out the missingness that existed in the resulting health expenditure dataset. ST-GPR is a stochastic modeling technique designed to detect signals amidst noisy data. It also serves as a powerful tool for interpolating non-linear trends. Unlike classical linear models that assume that the trend underlying data follows a definitive functional form, GPR assumes that the specific trend of interest follows a gaussian process, where each point can be estimated with a mean and covariance function.

The first step to implementing ST-GPR is to identify relevant covariates that would be helpful in predicting each health expenditure variable of interest. Using the following set of covariates, we estimated the first stage of the process (space-time) in order to predict and fill up the dependent variables. The covariates used are:

a) All-sector government expenditure per capita, logged
b) Healthcare Access and Quality Index, logged
c) Proportion of total population above the age of 65, logit transformed.

where the dependent variables were logs of GHE per capita, PPP per capita and OOP per capita.

Given the level of data, we were able to adjust the weight of each data point that contributed to the likelihood function of the Gaussian process, by inflating the pointwise variance for data points with lower weights. For missing data points, the resulting uncertainty was determined by region specific estimates. The final resulting dataset was a complete set of GHE, PPP and OOP per capita estimates for 188 countries from 1995 through 2015, where the uncertainty around each point was constructed by simulating from a normal distribution. Detailed description of the ST-GPR mechanism are described in the supplementary appendix of GBD 2016 Risk Factors Collaborators (2017).[85] This analysis was conducted in the following programs: Stata (version 13.1) and R (version 3.4.2).

eFigure 17 below contains four scatter plots of the indicators that were modeled through ST-GPR (THE being the sum of DAH, and modeled GHE, OOP and PPP) in 2017 PPP per capita space. Each year between 2000 through 2015 is represented by a different colors. The value of Pearson's correlation coefficient for each of four metrics in the graph are very strong, and therefore shows that the output from our modeling process were strongly correlated with the input data (0.9988 for GHE, 0.9988 for PPP, 0.9985 for OOP and 0.9953 for THE).

eFigure 17. Comparison of GHED Extracted data and ST-GPR Output

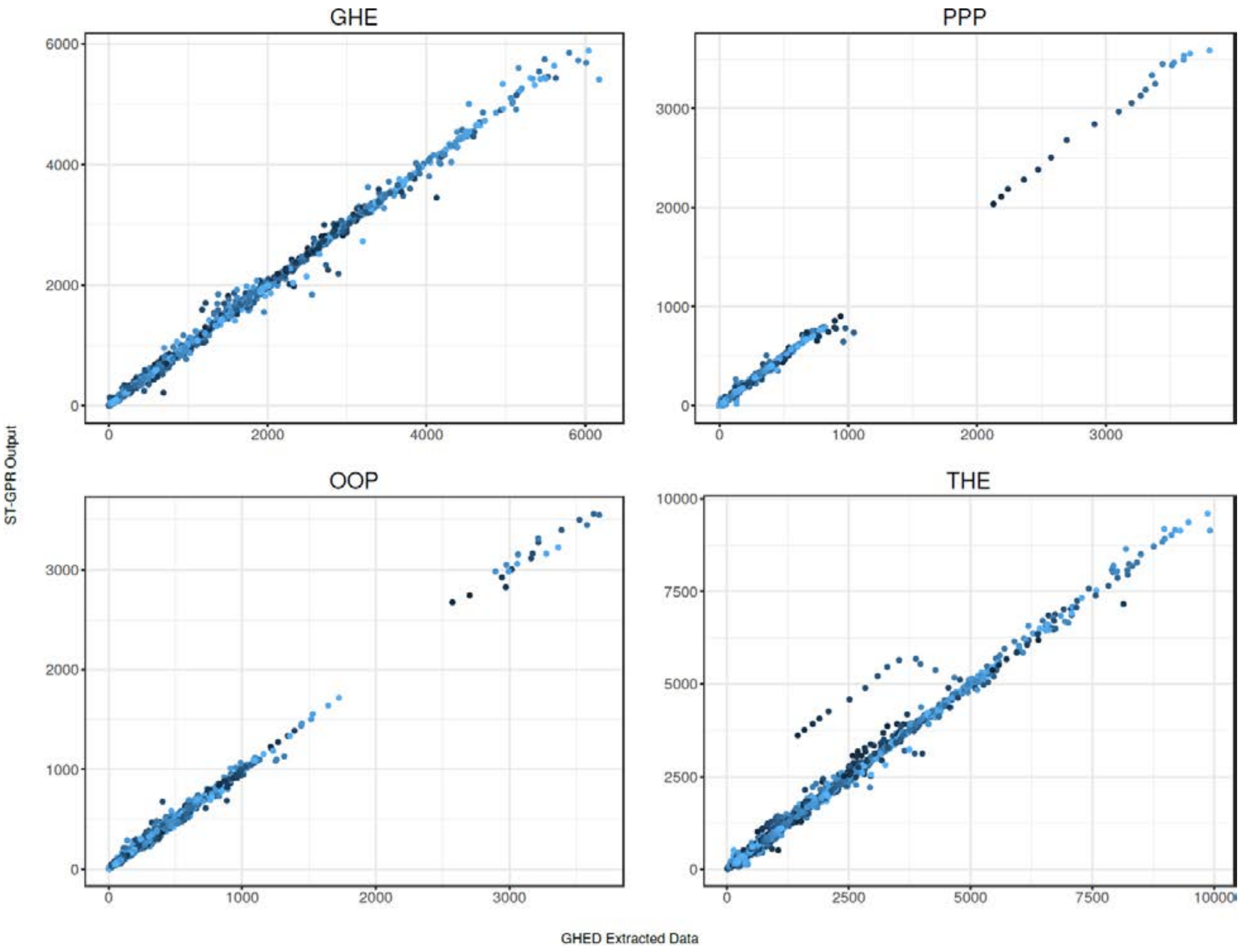

In eTable 18 we present the results from 10 fold out-of-sample root mean square error test performed in per cap space.

eTable 18: Out-of-sample root mean square error for overall health spending.

Model	Out-of-sample root mean square error
GHE	91.49
PPP	31.1
OOP	36.1

SECTION 4. TRACKING GLOBAL HIV SPENDING

Overview of data sources and data cleaning process

All data used for estimation of HIV/AIDS financing are publicly available through the websites of international institutions and public data aggregators. HIV/AIDS spending data were extracted from five sources:

- AIDSinfo database published by the Joint United Nations Programme on HIV/AIDS (UNAIDS)[86]
- Public and private spending data reported by countries in proposals and concept notes submitted to the Global Fund to Fight AIDS, Tuberculosis and Malaria (the Global Fund)[25]
- National Health Accounts that capture HIV/AIDS spending, including sub-accounts and accounts that adhere to the System of Health Accounts 2011 (SHA 2011) methodology[84]
- All National AIDS Spending Assessments (NASAs)[87]
- Asia Pacific region data downloaded from the AIDS data hub[88]

We leveraged the unique strengths across the different datasets, with the understanding that they were all generated to serve different purposes. The financing data collated by UNAIDS is sourced from annual reporting by countries to UNAIDS, in line with the 2000 Declaration on Commitment to HIV/AIDS. Similarly, countries report domestic spending in concept notes and proposals submitted to the Global Fund to secure funding. The Global Fund requires countries submit these estimates as part of a requirement that they contribute funds to the disease area of focus, in addition to Global Fund contributions. Staff at both the Global Fund and UNAIDS verify the data submitted to them, but in general do not publish data that has been altered from what countries themselves report.

Not all extracted data sources used the same definition of health expenditures. For example, National AIDS Spending Assessment's (NASAs) definition of health expenditure on HIV/AIDS followed a broader definition than the definition of health expenditure provided by National Health Accounts (NHAs). Specifically, NASAs included expenditure on non-health spending categories such as orphans and vulnerable children, creation of an enabling environment, and other social protection services.

To harmonize the definition of HIV/AIDS related health expenditure amongst data sources, when provided, we subtracted expenditure related to orphans and vulnerable children, creation of an enabling environment, and social protection services, from the respective sources and functions of health expenditure reported in the NASAs. When the reported data was not granular enough to make these adjustments, we down weighted the relevant data points. The three spending categories of orphans and vulnerable children, creation of an enabling environment, and social protection do not represent an exhaustive list of the deviations between NASAs and NHAs' HIV/AIDS spending definition, but do represent the vast majority of this deviation. Other spending categories that were included in NASAs but not included in NHAs were more granular and frequently not reported.

We extracted 5,385 unique data points. These data were sourced from a total of 61 National Health Accounts, 126 National AIDS Spending Assessments, 275 Global Fund for the Fight AIDS, Tuberculosis, and Malaria concept notes and proposals, and the AIDSinfo online database. Data for government health spending on HIV/AIDS was most substantial, with more than 1,987 data points. The fewest data points existed for HIV/AIDS care and treatment and prevention, respectively amounting to 783 and 748 data

points. eTable 21 and eTable 22 provide breakdown of the number of data points by year and quantity of interest and country and quantity of interest.

eFigure 16 captures the availability of HIV/AIDS spending data by country. Data density was highest in low- and middle-income countries with a large HIV/AIDS burden, notably Southern and Eastern sub-Saharan Africa; high-income countries, with the exception of the United States, had the fewest data points.

eFigure 16: Map of HIV/AIDS data availability

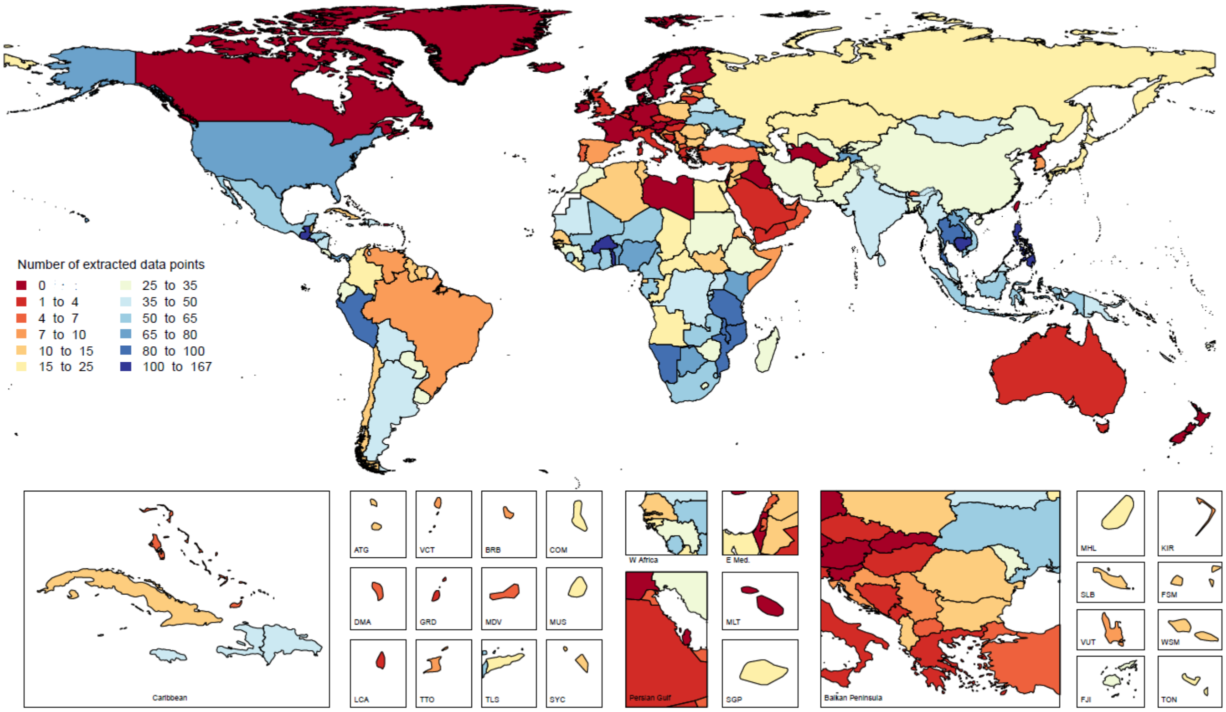

Currency exchange and deflation

Currency exchange and deflation

All HIV/AIDS expenditure estimates were made in 2017 purchasing power-adjusted dollars. Data source though reported expenditure in either nominal local currency units (LCUs) or nominal United States Dollars (USD). To convert nominal LCUs to purchasing power-adjusted dollars, we applied deflators to nominal LCU to inflate to 2017 LCUs. We then applied purchasing power parities to 2017 LCUs to produce 2017 purchasing power-adjusted dollars. When LCUs were not reported, we extracted reported expenditure in nominal USD, applied corresponding nominal exchange rates to produce nominal LCUs, inflated nominal LCUs to 2017 LCUs with deflators, and finally exchanged 2017 LCUs to purchasing power-adjusted dollars with purchasing power parities. All deflators, exchange rates, and purchasing power parities were extracted from the World Bank, International Monetary Fund, Penn World Tables, the United Nations National Accounts, and the World Health Organization, and were imputed to provide a complete series for each of the variables between 1950 and 2017. We then used several models including ordinary least-squares regression and mixed effects models, to complete each source series from 1950 to 2017. [83]

Modeling HIV/AIDS spending with ST-GPR

As previously mentioned in the manuscript, we used ST-GPR to model HIV/AIDS spending. The interested reader may view a complete description of ST-GPR here.[85] Briefly, ST-GPR has three primary steps. First, a linear mixed effects model is run with a given set of predictors. Predictions from the first step provide the general trend within the data. In the second step, spatiotemporal patterns were estimated by applying a series of spatiotemporal weights to average the residuals of the first step linear model. These spatiotemporal patterns were then added to the linear prediction to generate spatiotemproal predictions. Finally, the spatiotemporal predictions served as the mean function of a gaussian process regressions run across time on the data. Estimates of the Gaussian process regressions served as final ST-GPR predictions and generated a complete time-series of data from 2000 to 2015 in 188 countries, building from data when available and borrowing strength across time, geographic regions, and covariates' predictive power when data was not available.

For the first step of ST-GPR—the linear model—we used a linear mixed effects regression with random effects on super region, region, and country level. To select the fixed effect covariates that were most predictive, we performed 10 fold cross validation on every covariate combination of natural log of five-year lag distributed income per capita (LDI), natural log of ART prices, natural log of HIV/AIDS prevalence, natural log of HIV incidence, natural log of HIV/AIDS mortality, and ART coverage. All covariates were sourced from GBD 2016. We selected the model that minimized out-of-sample root mean square error; the selected covariates for each model and the out-of-sample root mean square error reported in logit space are displayed in eTable 19.

In the second step, we created spatiotemporal predictions by smoothing the predictions from the first step model based upon systematic deviations in the residuals of the first step model across time and geographic locations. The spatiotemporal predictions were passed as the mean function to a Gaussian process regression (with an amplitude of 1 and a scale parameter set to 7.5) along with the data to produce final ST-GPR predictions. Gaussian process regressions provide a measurement of variance that is largely influenced by the amplitude parameter, however, to further increase our uncertainty we added the variance of the residuals after the Gaussian process regressions to the estimated variance. For every country-year estimate, 1,000 draws were generated from the models' posterior distribution to propagate uncertainty in latter processes.

eTable 19: Covariates selected in for first step model in ST-GPR and out-of-sample root mean square error

ST-GPR model	Covariates	Out-of-sample root mean square error
Domestic	ART price, Art coverage, HIV/AIDS prevalence, HIV/AIDS mortality rate	0.79
Government	ART coverage, HIV incidence, HIV/AIDS mortality	0.82
Private	LDI, ART price, HIV/AIDS prevalence	2.34
OOP	ART coverage, HIV/AIDS prevalence, HIV incidence, HIV/AIDS mortality rate	1.78

PPP	ART price, ART coverage, HIV/AIDS prevalence, HIV incidence, HIV/AIDS mortality	3.16
Domestic HIV/AIDS spending on care and treatment	ART price	1.24
Domestic HIV/AIDS spending on prevention	ART price	1.47
Domestic HIV/AIDS spending on all other functions	ART price, ART coverage, HIV incidence	1.74

Enforcing internal consistency

To ensure internal consistency between the HIV/AIDS spending estimates and the all health spending estimates, HIV/AIDS spending by source was modeled as the logit transformed fraction of the respective, loess smoothed, all health spending by source estimate (e.g. domestic HIV/AIDS spending divided by all domestic health spending). As a consistency check, extracted data points were outliered if the fraction between HIV/AIDS spending by source and all health spending by source exceeded one.

While the above transformation helped ensure internal consistency between HIV/AIDS spending and all health spending, we were still required to ensure internal consistency within our estimates such that domestic HIV/AIDS spending did not exceed total HIV/AIDS spending and to take advantage of all the extracted data and implemented models. These objectives were accomplished by both aggregating and raking. Aggregating is the process of summing mutually exclusive and collectively exhaustive estimates of sub-components of health expenditure (e.g. OOP, PPP, GHES, DAH-HIV/AIDS) and using the sum as the estimate of total health expenditure. Raking is the exact opposite of aggregating. In raking, we used estimates of total health expenditure to evenly scale the estimated sub-components to ensure the sub-components sum to the estimated total health expenditure. Raking and aggregating are equally valid and widely used in health financing and in the Global Burden of Disease.[89,90]

In our extracted dataset, few data sources (NHAs and NASAs) reported OOP, prepaid private, or total HIV/AIDS spending (sum of OOP, prepaid private, public, DAH-HIV/AIDS), while nearly all data sources reported expenditure of either public, private (sum of OOP and prepaid private, but not disaggregated), and total domestic (sum of public and private, but not disaggregated) HIV/AIDS spending. Given this inconsistency, we modeled the five financing source spending variables in eTable 19 and raked and aggregated estimates to draw strength across areas with the highest data density. This process was implemented by averaging the domestic HIV/AIDS spending estimates with the aggregated domestic HIV/AIDS spending estimate formed by summing estimates of public and private HIV/AIDS spending. This averaged result represented our final estimate of domestic HIV/AIDS spending. We then raked estimates of public and private HIV/AIDS spending to the final domestic HIV/AIDS spending envelope to produced final private and public HIV/AIDS spending estimates. The final private HIV/AIDS spending estimates were then used as an envelope to rake OOP and prepaid private HIV/AIDS spending estimates. To propagate uncertainty, we conducted both aggregating and raking on the draw level. As final check for internal consistency with all health spending estimates, we replaced any draw where HIV/AIDS financing source exceeded 50% of the corresponding all health spending estimate. When these internal consistency issues arose, we replaced the HIV/AIDS spending estimate with 0.5 multiplied by the corresponding all health spending draw. This occurred in less than 0.05% of all country-year-source-draws.

To generate estimate of total HIV/AIDS spending by function (prevention, care and treatment, and other), we estimated domestic HIV/AIDS spending by function and deterministically added DAH spending by the analogous function. We first mapped DAH-HIV/AIDS by health focus areas into three spending function categories presented in eTable 20.

eTable 20: Aggregation of DAH-HIV/AIDS health focus areas into HIV/AIDS function spending

HIV/AIDS spending functions	DAH-HIV/AIDS health focus area
Prevention	Prevention, PMTCT
Care and treatment	Treatment, Care, Counseling and Testing
Other	Health system strengthening
	Unidentified

Unfortunately, a portion of DAH-HIV/AIDS spending could not be assigned to a health focus area—this was especially true in early years. To account for this and fully attribute all HIV/AIDS spending to a spending function, using the extracted data we modeled total HIV/AIDS spending by function (prevention, care and treatment, and other) in ST-GPR and used these estimates to proportionally split the unidentified DAH-HIV/AIDS expenditure into HIV/AIDS spending functions. This approach assumes relative proportions of total HIV/AIDS spending by function matched the unidentified portion of DAH-HIV/AIDS spending.

To estimate domestic spending by function we ran ST-GPR. To gather the necessary underlying data, we extracted domestic spending by function, but few data source (only NASAs, NHAs, GARPR reports) provided this information. Other data sources reported total spending by function but failed to further disaggregate spending on function by financing source. To leverage this data, we subtracted DAH spending by function from reported total HIV/AIDS spending on analogous functions (e.g. total HIV/AIDS spending on prevention less DAH –HIV/AIDS spending on prevention). In cases where this subtraction yielded values below zero, we dropped the data point. Final estimates of HIV/AIDS spending were made in the logit transformed space of fraction of HIV/AIDS spending over total domestic HIV/AIDS spending. Final estimates of domestic spending by function were scaled to total domestic spending at the draw level. eTable 21 and eTable 22 provide number of data source used in each model by time and location. location. A

eTable 21. Count of data sources over time by financing sources and spending functions. Data counts reflect counts of data that went into the model rather than extracted data points.

Year	Domestic spending	Public spending	Private spending	Out-of-pocket spending	Prepaid private spending	Domestic spending on care and treatment	Domestic spending on prevention	Domestic spending on all other areas
**2000**	7	9	5	4	1	7	2	4
**2001**	7	11	5	2	2	7	4	3
**2002**	9	15	8	7	2	10	3	9
**2003**	9	15	6	2	1	4	4	4
**2004**	15	21	10	3	4	7	8	4
**2005**	35	47	30	6	10	11	13	7
**2006**	52	104	48	13	13	25	21	18
**2007**	43	155	59	15	20	39	39	27
**2008**	41	200	89	16	23	64	64	46
**2009**	46	214	100	18	18	57	55	35
**2010**	53	215	105	18	18	53	48	36
**2011**	43	186	93	12	16	38	33	22
**2012**	52	175	114	21	26	35	35	25
**2013**	28	118	68	14	15	33	24	22
**2014**	104	172	77	10	12	14	15	7
**2015**	87	140	51	5	6	4	8	4

eTable 22. Count of data sources, by location. Data counts reflect counts of data that went into the model rather than extracted data points.

Country	Domestic spending	Public spending	Private spending	Out-of-pocket spending	Prepaid private spending	Domestic spending on care and treatment	Domestic spending on prevention	Domestic spending on all other areas
Afghanistan	0	10	0	0	0	0	1	0
Albania	4	5	0	0	0	0	0	0
Algeria	2	11	2	0	0	4	4	2
Angola	4	11	3	0	0	0	3	0
Antigua and Barbuda	0	8	2	0	0	0	0	0
Argentina	2	13	2	0	0	0	4	0
Armenia	2	13	5	0	0	0	0	0
Australia	0	1	0	0	0	0	0	0
Azerbaijan	2	12	0	0	0	1	1	0
Bangladesh	2	11	2	0	0	0	0	0
Barbados	0	7	2	0	0	5	9	10
Belarus	2	20	2	0	0	7	10	2
Belgium	0	3	0	0	0	0	0	0
Belize	2	8	3	0	0	2	3	2
Benin	10	19	13	6	3	3	3	1
Bhutan	2	4	0	0	0	0	0	0
Bolivia	5	14	7	3	3	5	6	2
Bosnia and Herzegovina	2	5	1	0	0	0	0	0
Botswana	11	24	16	2	5	6	6	6
Brazil	0	9	0	0	0	7	7	5
Bulgaria	0	13	0	0	0	0	0	0
Burkina Faso	26	33	30	14	14	7	12	9
Burundi	6	17	11	4	4	6	4	1
Cambodia	14	33	15	0	0	9	0	5
Cameroon	9	18	14	1	1	2	4	2
Cape Verde	4	13	9	0	0	0	0	0
Central African Republic	4	8	5	0	0	0	0	0
Chad	0	9	6	2	0	8	6	2
Chile	0	7	7	0	0	0	0	2
China	2	18	6	0	0	1	3	1
Colombia	4	13	10	0	0	4	8	6
Comoros	5	14	4	0	0	0	0	0
Congo	4	9	3	1	1	0	0	0

Costa Rica	4	9	6	0	0	4	3	4
Cote d'Ivoire	9	19	19	7	7	9	6	1
Croatia	0	8	0	0	0	0	0	0
Cuba	2	11	0	0	0	0	0	0
Czech Republic	0	3	3	0	0	0	0	0
Democratic Republic of the Congo	12	14	14	3	6	0	0	0
Djibouti	6	6	0	0	0	0	1	0
Dominica	0	5	0	0	0	0	0	0
Dominican Republic	6	10	8	0	2	2	2	2
Ecuador	2	12	1	0	3	3	3	2
Egypt	2	4	2	0	2	1	1	0
El Salvador	13	22	20	1	0	9	9	8
Equatorial Guinea	1	5	5	0	0	0	0	0
Eritrea	4	5	0	0	0	0	0	0
Estonia	0	2	0	0	0	0	0	0
Ethiopia	6	14	5	2	3	1	0	0
Federated States of Micronesia	0	0	0	0	0	5	0	2
Fiji	0	8	0	0	1	5	0	5
Gabon	0	15	11	5	5	4	5	4
Georgia	10	19	18	0	0	7	6	1
Ghana	10	18	14	1	4	3	4	1
Greece	0	1	0	0	0	0	0	0
Grenada	0	3	0	0	0	0	0	0
Guatemala	16	28	27	5	7	13	14	8
Guinea	5	16	13	1	1	1	4	1
Guinea-Bissau	6	10	5	0	0	0	1	0
Guyana	4	5	5	0	0	0	0	0
Haiti	5	19	3	0	0	0	2	1
Honduras	10	20	18	3	3	5	4	5
Hungary	0	3	0	0	0	0	0	0
India	10	18	6	0	0	0	3	0
Indonesia	4	23	4	0	0	7	1	3
Iran	3	10	5	0	0	3	1	2
Italy	0	1	0	0	0	0	0	0
Jamaica	8	13	10	0	0	2	4	3
Japan	0	4	0	0	0	4	4	3

**Jordan**	0	9	0	0	0	0	0	0
**Kazakhstan**	2	11	2	0	0	0	0	0
**Kenya**	11	20	10	5	7	7	6	2
**Kiribati**	0	1	0	0	0	1	1	0
**Kuwait**	0	7	0	0	0	0	0	0
**Kyrgyzstan**	3	18	1	0	0	2	0	0
**Laos**	7	19	2	0	0	1	1	2
**Latvia**	0	8	2	0	0	0	0	0
**Lebanon**	0	6	0	0	0	0	0	0
**Lesotho**	10	15	8	0	0	4	3	3
**Liberia**	3	8	2	0	2	0	0	0
**Lithuania**	0	2	0	0	0	0	0	0
**Macedonia**	0	6	1	0	0	0	0	0
**Madagascar**	3	14	12	0	0	1	2	2
**Malawi**	11	26	15	14	6	3	0	2
**Malaysia**	4	12	9	0	0	9	10	8
**Maldives**	0	2	0	0	0	0	0	0
**Mali**	10	16	13	3	3	7	7	3
**Marshall Islands**	0	8	0	0	0	8	1	4
**Mauritania**	9	12	6	3	3	3	0	3
**Mauritius**	4	8	4	0	0	2	2	0
**Mexico**	9	17	15	0	0	16	16	6
**Moldova**	2	15	9	0	0	10	2	3
**Mongolia**	4	15	8	0	2	3	0	0
**Montenegro**	4	2	2	0	0	0	0	0
**Morocco**	2	13	10	0	0	5	5	2
**Mozambique**	7	31	15	0	9	5	5	0
**Myanmar**	2	15	4	0	0	0	0	0
**Namibia**	9	18	13	7	9	8	5	9
**Nepal**	2	10	3	2	2	5	2	1
**Nicaragua**	6	11	6	4	4	2	4	3
**Niger**	9	20	16	7	7	2	2	0
**Nigeria**	4	22	13	0	7	7	8	4
**Oman**	0	6	0	0	0	2	2	2
**Pakistan**	2	11	2	0	0	3	4	3
**Palestine**	0	2	0	0	0	0	0	0
**Panama**	16	22	21	1	0	4	4	4
**Papua New Guinea**	6	10	8	0	0	3	0	3
**Paraguay**	2	16	7	0	0	0	0	0
**Peru**	12	21	14	6	6	8	13	11
**Philippines**	12	33	14	0	3	13	9	9

Poland	0	8	3	0	0	0	0	0
Portugal	0	5	0	0	0	0	0	0
Romania	0	10	1	0	0	0	0	0
Russian Federation	4	5	2	0	0	0	0	0
Rwanda	9	15	7	5	0	5	1	5
Saint Lucia	0	2	0	0	0	0	0	0
Saint Vincent and the Grenadines	0	5	3	0	0	2	1	1
Samoa	1	5	1	0	0	3	2	0
Sao Tome and Principe	2	10	1	0	0	0	0	0
Saudi Arabia	0	1	0	0	0	1	1	1
Senegal	2	10	2	0	0	0	0	0
Serbia	0	4	1	0	0	0	0	0
Seychelles	0	9	3	0	0	2	3	3
Sierra Leone	2	15	4	0	3	0	4	0
Singapore	0	8	0	0	0	5	5	0
Solomon Islands	0	4	0	0	0	2	0	1
Somalia	2	2	0	0	0	0	0	0
South Africa	8	14	11	0	0	3	2	2
South Korea	0	3	0	0	0	3	3	0
South Sudan	2	4	0	0	0	0	0	0
Spain	0	7	0	0	0	0	0	0
Sri Lanka	6	11	4	2	2	3	0	0
Sudan	2	13	5	0	0	0	0	0
Suriname	4	6	5	0	0	2	1	2
Swaziland	11	20	8	0	3	9	5	3
Switzerland	0	8	0	0	0	0	0	0
Syria	0	6	3	0	0	0	0	0
Tajikistan	8	19	11	4	0	6	7	0
Tanzania	11	16	11	6	7	4	3	6
Thailand	6	22	10	0	0	13	16	15
The Bahamas	0	3	3	0	0	0	0	0
The Gambia	2	7	2	0	0	0	0	0
Timor-Leste	4	4	0	0	0	0	0	0
Togo	16	27	25	14	7	7	6	8
Tonga	0	5	5	0	0	0	2	0
Trinidad and Tobago	0	4	4	0	0	0	0	0
Tunisia	2	6	2	0	0	5	5	0
Turkey	2	3	2	0	0	0	0	0

**Uganda**	8	15	6	2	0	3	0	0
**Ukraine**	6	19	17	2	2	2	2	2
**United Arab Emirates**	0	4	0	0	0	0	0	0
**United Kingdom**	0	3	0	0	0	0	0	0
**United States**	14	14	14	14	14	0	0	0
**Uruguay**	3	6	6	0	1	5	5	5
**Uzbekistan**	2	13	0	0	0	0	0	0
**Vanuatu**	0	5	0	0	0	1	0	0
**Venezuela**	0	9	0	0	0	9	10	9
**Vietnam**	5	20	5	3	0	4	4	7
**Yemen**	0	3	0	0	0	0	0	0
**Zambia**	6	14	6	1	3	0	2	0
**Zimbabwe**	6	12	6	0	0	0	0	0

REFERENCES

1 Dieleman JL, Schneider MT, Haakenstad A, *et al*. Development assistance for health: past trends, associations, and the future of international financial flows for health. *The Lancet* 2016; **387**: 2536–44.

2 OECD. International Development Statistics (IDS) online databases. https://www.oecd.org/development/stats/idsonline.htm (accessed Jan 7, 2017).

3 European Commission. Annual reports - international cooperation and development. Int. Coop. Dev. /europeaid/annual-reports_en (accessed Jan 26, 2017).

4 UNAIDS. PCB Archive. http://www.unaids.org/en/aboutunaids/unaidsprogrammecoordinatingboard/pcbmeetingarchive (accessed Jan 26, 2017).

5 UNICEF. UNICEF integrated budget, 2014-2017. 2013. https://www.unicef.org/about/execboard/files/2013-ABL4-UNICEF_integrated_budget-11Jul2013.pdf.

6 UNICEF. United Nations Children's Fund financial report and audited financial statements. New York: United Nations.

7 UNICEF. Annual Report 2015. UNICEF. https://www.unicef.org/publications/index_92018.html (accessed Jan 30, 2017).

8 United Nations Population Fund. Annual Report 2015. http://www.unfpa.org/annual-report (accessed Jan 30, 2017).

9 UNITAID. Audited Financial Statements (2007-2016). Unitaid. https://unitaid.eu/publications/ (accessed Dec 22, 2017).

10 Pan American Health Organization, World Health Organization. Executive Committee Session. 2016; published online Dec 20. http://www.paho.org/hq/index.php?option=com_content&view=category&id=1258&layout=blog&Itemid=1160&lang=en (accessed Jan 30, 2017).

11 World Health Organization. Financial report and audited financial statement, 2014. World Health Organization, 2015 http://apps.who.int/gb/ebwha/pdf_files/WHA68/A68_38-en.pdf.

12 The World Bank. Projects & Operations. http://projects.worldbank.org/ (accessed Jan 30, 2017).

13 Paris M. Project database 1990-2016 obtained through personal correspondence. 2016; published online Sept 29.

14 Asian Development Bank. Online project database. https://www.adb.org/projects (accessed Jan 30, 2017).

15 African Development Bank. Compendium of statistics on bank group operations. Tunis, Tunisia: Statistics Department, African Development Bank.

16 African Development Bank. Online project database. https://www.afdb.org/en/projects-and-operations/project-portfolio/ (accessed Jan 30, 2017).

17 Inter-American Development Bank. Projects database. http://www.iadb.org/en/projects/projects,1229.html (accessed Jan 30, 2017).

18 Neret M. health disbursement data. 2016; published online Dec 16.

19 Gavi, the Vaccine Alliance. Disbursements and commitments. http://www.gavi.org/results/disbursements/ (accessed Feb 27, 2017).

20 Gavi, the Vaccine Alliance. Pneumococcal AMC. http://www.gavi.org/funding/pneumococcal-amc/ (accessed Jan 30, 2017).

21 Gavi, the Vaccine Alliance. Financial reports. http://www.gavi.org/funding/financial-reports/ (accessed Feb 27, 2017).

22 Gavi, the Vaccine Alliance. Cash Received database. http://www.gavi.org/funding/donor-contributions-pledges/cash-receipts/ (accessed Feb 27, 2017).

23 The Global Fund to fight AIDS, Tuberculosis and Malaria. Grants in detail and Disbursements. http://www.theglobalfund.org/en/portfolio/ (accessed Jan 30, 2017).

24 The Global Fund to fight AIDS, Tuberculosis and Malaria. GFATM annual reports. 2017; published online Jan 30. http://www.theglobalfund.org/en/archive/annualreports/ (accessed Jan 30, 2017).

25 The Global Fund to fight AIDS, Tuberculosis and Malaria. GFATM pledges & contributions report. http://www.theglobalfund.org/en/ (accessed Jan 30, 2017).

26 GuideStar USA. Income tax filings. http://www2.guidestar.org/Home.aspx (accessed Jan 30, 2017).

27 United States Agency for International Development. VolAg report: report of voluntary agencies engaged in overseas relief and development. https://www.usaid.gov/pvo/volag-report (accessed Jan 30, 2017).

28 Thomson Reuters. Red book expanded database. New York: Thomson Reuters, 2009.

29 World Health Organization. WHO | Essential medicines. WHO. http://www.who.int/topics/essential_medicines/en/ (accessed Feb 15, 2017).

30 Bill & Melinda Gates Foundation. Audited Financial statements. Seattle, WA: Bill & Melinda Gates Foundation http://www.gatesfoundation.org/Who-We-Are/General-Information/Financials (accessed Jan 30, 2017).

31 Chan S. Foundation Awards and Payments. 2016; published online Aug 16.

32 Foundation Center. Grants database. Found. Cent. http://foundationcenter.org (accessed Jan 30, 2017).

33 Ahogny J. AfDB development support for health projects enquiry. 2017; published online Nov 16.

34 Rouzinova R. Incorporating UNITAID into Financing Global Health Landscape. 2017; published online Aug 21.

35 Al Anood Al Abdool., United Arab Emirates Ministry of Foreign Affairs and International Cooperation. UAE Foreign Assistance in Health 1990-2008 through personal correspondence. 2018; published online Jan 24.

36 Australian Government. Department of Foreign Affairs and Trade. Aid budget and statistical information. Dep. Foreign Aff. Trade. http://dfat.gov.au/aid/aid-budgets-statistics/Pages/default.aspx (accessed Feb 27, 2017).

37 Department of Foreign Affairs and Trade. Annual reports. Dep. Foreign Aff. Trade. http://dfat.gov.au/about-us/publications/corporate/annual-reports/pages/annual-reports.aspx (accessed Feb 15, 2017).

38 Austria Federal Ministry of Finance. Federal budget. https://www.bmf.gv.at/budget/das-budget/das-budget.html (accessed Jan 30, 2017).

39 Belgium House of Representatives. Project budgets. http://www.lachambre.be/kvvcr/showpage.cfm?section=/pri/budget&language=fr&rightmenu=right_pri&story=2017-budget.xml (accessed Feb 13, 2018).

40 Government of Canada. Planning and performance. GAC. http://international.gc.ca/gac-amc/publications/plans/index.aspx?lang=eng#rpp (accessed Jan 30, 2017).

41 Danish Ministry of Foreign Affairs. Foreign affairs budget. http://www.oes-cs.dk/bevillingslove/ (accessed Jan 30, 2017).

42 European Commission. General Budget - budget on-line. http://eur-lex.europa.eu/budget/www/index-en.htm (accessed Jan 30, 2017).

43 Ministry of Finance Finland. State budget bills [in Finnish]. http://budjetti.vm.fi/indox/index.jsp (accessed Jan 31, 2017).

44 Ministry of Foreign Affairs and International Development. Politique francaise en faveur du developpement. Ministry of Foreign Affairs and International Development, 2017 https://www.performance-publique.budget.gouv.fr/sites/performance_publique/files/farandole/ressources/2017/pap/pdf/DPT/DPT2017_politique_developpement.pdf (accessed Feb 12, 2018).

45 Legifrance. Republique Francaise. Budget and financial documents. https://www.legifrance.gouv.fr/initRechTexte.do (accessed Feb 15, 2018).

46 German Federal Ministry of Economic Cooperation and Development. Plan of the federal budget. Fed. Minist. Econ. Coop. Dev. http://www.bmz.de/en/ministry/InDetail/budget/index.html (accessed Feb 13, 2018).

47 Greece Standing Committee on Economic Affairs. The state budget and budgets for certain special funds and services, 2013 and 2014 [in Greek]. 2014. http://www.hellenicparliament.gr/UserFiles/7b24652e-78eb-4807-9d68-e9a5d4576eff/Proyp2014-prak.pdf.

48 Greece Standing Committee on Economic Affairs. Ratification of the State Budget for the financial year 2017. http://www.hellenicparliament.gr/Nomothetiko-Ergo/Anazitisi-Nomothetikou-Ergou?law_id=76e87dd3-8cae-433f-963f-a6c50141de97 (accessed Feb 13, 2018).

49 Department of Finance, Government of Ireland. The Budget. http://www.budget.gov.ie/Budgets/2017/2017.aspx (accessed Feb 15, 2018).

50 Ministry of Foreign Affairs and International Cooperation. Stato di previsione del ministero degli affari esteri e della cooperazione internazionale. 2017. http://www.rgs.mef.gov.it/_Documenti/VERSIONE-I/Attivit--i/Bilancio_di_previsione/Bilancio_finanziario/2017/Allegato-t-17-19/2017-DLB-04-AT-060-Esteri.pdf (accessed Feb 15, 2018).

51 Ministry of Finance Japan. Budget. http://www.mof.go.jp/english/budget/budget/index.html (accessed Jan 31, 2017).

52 Korea Official Development Assistance. Comprehensive Implementation plan for international development cooperation by year. http://www.odakorea.go.kr/hz.blltn2.YearPlanSlPL.do?brd_seq=3&blltn_div=oda (accessed Feb 15, 2018).

53 Ministry of Finance Luxembourg. State Budget[in French]. http://www.igf.etat.lu/.

54 New Zealand Treasury. Vote budget data - budgets of the New Zealand Government. 2017; published online Jan 31. http://www.treasury.govt.nz/budget (accessed Jan 31, 2017).

55 Ministry of Foreign Affairs Norway. Email correspondences. April 18, 2011, February 13, 2012, and August 14, 2013. .

56 Norwegian Ministry of Finance. National Budget. https://www.statsbudsjettet.no/english/ (accessed Feb 15, 2018).

57 Ministry of Finance Portugal. State budget report. http://www.dgo.pt/Paginas/default.aspx (accessed Jan 31, 2017).

58 Ministry of Finance and Public Function Spain. Annual plan of cooperation (PACI). http://www.aecid.es/EN/cultura/Paginas/Publicaciones/Coop_Espanola/PACI/PACI.aspx (accessed Feb 15, 2018).

59 Ministry of Foreign Affairs Sweden. International aid budget. http://www.regeringen.se/ (accessed Jan 31, 2017).

60 UK - Department for International Development. IATI Dashboard. http://dashboard.iatistandard.org/publisher/dfid.html (accessed Feb 22, 2017).

61 Her Majesty's Treasury United Kingdom. Budget. https://www.gov.uk/government/publications/spring-budget-2017-documents (accessed Feb 15, 2018).

62 Executive Office of the President of the United States. Budget of the United States Government. https://www.gpo.gov/fdsys/browse/collection.action?collectionCode=BUDGET&browsePath=Fiscal+Year+2014&isCollapsed=true&leafLevelBrowse=false&isDocumentResults=true&ycord=0 (accessed Jan 31, 2017).

63 US Foreign Assistance Dashboard. Foreign assistance by category, health-planned stage. https://www.foreignassistance.gov/explore (accessed Feb 15, 2018).

64 World Health Organization. Proposed programme budget. http://www.who.int/about/finances-accountability/budget/en/ (accessed Jan 31, 2017).

65 Joint United Nations Programme on HIV/AIDS. Financial report and audited financial statement. http://www.unaids.org/en/aboutunaids/unaidsprogrammecoordinatingboard/pcbmeetingarchive/ (accessed Jan 31, 2017).

66 Joint United Nations Programme on HIV/AIDS. Unified budget and workplan. http://www.unaids.org/en/aboutunaids/unaidsprogrammecoordinatingboard/pcbmeetingarchive/ (accessed Jan 31, 2017).

67 United Nations Children's Fund. Financial Report and Audited Financial Statement - Executive Board documents. https://www.unicef.org/about/execboard/index_25993.html (accessed Jan 31, 2017).

68 Sabbah L. Health expenditure data - UNICEF. 2015; published online Sept 29.

69 UNFPA. Report on contributions by member states and others to UNFPA and revenue projections. https://executiveboard.unfpa.org/ (accessed Jan 31, 2017).

70 UNFPA. Statistical and Financial review - Audited Financial report. https://executiveboard.unfpa.org/ (accessed Jan 31, 2017).

71 Smithson M, Verkuilen J. A better lemon squeezer? Maximum-likelihood regression with beta-distributed dependent variables. *Psychol Methods* 2006; **11**: 54–71.

72 Office of the European Union. EU budget 2014 Financial report. Luxembourg: Office of the European Union, 2015 http://ec.europa.eu/budget/financialreport/2014/lib/financial_report_2014_en.pdf (accessed April 17, 2017).

73 President's Emergency Plan for AIDS Relief/ US Government. PEPFAR Dashboards-Country and regional program results, FY 2016. 2017. https://data.pepfar.net/global (accessed Feb 14, 2018).

74 President's Malaria Initiative/US Government. Malaria Operational Plans (MOPs). 2017. https://www.pmi.gov/resource-library/mops (accessed Feb 14, 2018).

75 Miyuki Parris. Project database 1990-2016 obtained through personal correspondence. Washington D.C.: The World Bank, 2016.

76 Projects & Operations - Sectors. http://projects.worldbank.org/sector (accessed Jan 26, 2018).

77 The World Bank. World Bank Lending by Sector, Fiscal Years 2012-16. http://www.worldbank.org/en/about/annual-report/fiscalyeardata#4 (accessed April 17, 2017).

78 The World Bank. World Bank Country and Lending Groups – World Bank Data Help Desk. https://datahelpdesk.worldbank.org/knowledgebase/articles/906519-world-bank-country-and-lending-groups (accessed Dec 23, 2017).

79 Gavi, the Vaccine Alliance. Annual financial reports. http://www.gavialliance.org/funding/financial-reports/ (accessed Jan 30, 2017).

80 World Health Organization. WHO Programmatic and Financial Report (including audited financial statements). Geneva, Switzerland: World Health Organization, 2016 http://apps.who.int/gb/ebwha/pdf_files/WHA69/A69_45-en.pdf?ua=1 (accessed April 17, 2017).

81 Pan American Health Organization. Financial Report of the Director and Report of the External Auditor. 1 January 2015 - 31 December 2015. Washington D.C.: Pan American Health Organization, 2016 http://iris.paho.org/xmlui/handle/123456789/33710.

82 Schlutter A, Volker T, Walkenhorst P. Foundations in Europe: International Reference Book on Society, Management and Law. Gutersloh, Germany; Washington, DC: Bertelsmann Stiftung; Brookings Institution Press, 2001.

83 James SL, Gubbins P, Murray CJ, Gakidou E. Developing a comprehensive time series of GDP per capita for 210 countries from 1950 to 2015. *Popul Health Metr* 2012; **10**: 12.

84 World Health Organization. Global Health Expenditure Database. http://apps.who.int/nha/database/ViewData/Indicators/en (accessed Dec 23, 2017).

85 Gakidou E, Afshin A, Abajobir AA, *et al.* Global, regional, and national comparative risk assessment of 84 behavioural, environmental and occupational, and metabolic risks or clusters of risks, 1990–2016: a systematic analysis for the Global Burden of Disease Study 2016. *The Lancet* 2017; **390**: 1345–422.

86 GAM - Global AIDS Monitoring. http://www.aidsinfoonline.org/gam/libraries/aspx/Home.aspx (accessed Dec 19, 2017).

87 NASA country reports. http://www.unaids.org/en/dataanalysis/knowyourresponse/nasacountryreports (accessed Dec 19, 2017).

88 Key facts on HIV in Asia and the Pacific (2016). http://www.aidsdatahub.org/ (accessed Dec 19, 2017).

89 Foreman KJ, Lozano R, Lopez AD, Murray CJ. Modeling causes of death: an integrated approach using CODEm. *Popul Health Metr* 2012; **10**: 1.

90 Dieleman JL, Campbell M, Chapin A, *et al.* Future and potential spending on health 2015–40: development assistance for health, and government, prepaid private, and out-of-pocket health spending in 184 countries. *The Lancet* 2017; **389**: 2005–30.

91 Iglewicz B, Hoaglin DC. How to detect and handle outliers. Milwaukee, WI: ASQC Quality Press, 1993.
